# Quinones as Multifunctional Scaffolds for Oxidative, Reductive, and HAT Photocatalysis

**DOI:** 10.1002/chem.202404707

**Published:** 2025-03-02

**Authors:** Lea Müller, Jonas Poll, Patrick Nuernberger, Indrajit Ghosh, Burkhard König

**Affiliations:** ^1^ Fakultät für Chemie und Pharmazie Universität Regensburg 93053 Regensburg Germany; ^2^ Institut für Physikalische und Theoretische Chemie Universität Regensburg 93053 Regensburg Germany; ^3^ Nanotechnology Centre Centre for Energy and Environmental Technologies VSB - Technical University of Ostrava 708 00 Ostrava-Poruba Czech Republic

**Keywords:** Photocatalysis, Quinone, Reductive, Oxidative, HAT catalysis, large redox window

## Abstract

Photoredox catalysis, which enables both electron and hydrogen atom transfer, has become a powerful tool for activating chemical bonds and synthesizing complex molecules under mild conditions. Typically, photocatalysts are optimized either for oxidative or reductive reactions within a limited redox window (less than 3.1 V) and for hydrogen atom transfer (HAT) reactions, with few frameworks capable of mediating both pathways for high redox‐demanding reactions (covering more than a 5 V redox window) without requiring special conditions. Herein, we report the use of quinones as multifunctional scaffolds in light‐driven redox transformations, offering access to a redox window of approximately 5 V using visible light. The quinone scaffold's versatility facilitates a wide range of radical and ionic processes under both oxidative and reductive conditions, in addition to enabling HAT reactions. By keeping the parameters, i. e. the reaction partners, constant, such transformations can be carried out under just two reaction conditions. Oxidative transformations and HAT reactions occur under ambient air, while activation of the chromophore for reductive transformations can be achieved using an inorganic base (Cs_2_CO_3_) via a simple acid‐base deprotonation event. This dual capability highlights the potential of quinones as scaffolds to extend their utility in photoredox catalysis.

## Graphical abstract

Quinone scaffolds facilitate light‐driven redox transformations across a redox window of approximately 5 V, supporting oxidative, reductive, and HAT reactions. Oxidative and HAT processes occur under ambient air, while reductive transformations require an inorganic base. Their adaptability to facilitate a broad range of radical and ionic processes under just two conditions highlights the potential of quinone scaffolds in photoredox catalysis.

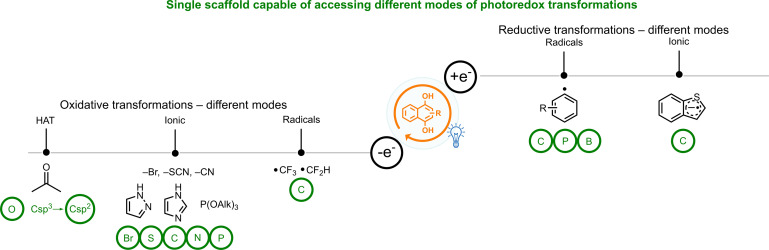

## Introduction

Over the past couple of decades, photoredox catalysis has emerged as a cornerstone of chemical synthesis,[^1^, ^2^, ^3^, ^4^, ^5^] providing a versatile platform for oxidation, reduction, and hydrogen atom transfer (HAT) reactions under mild conditions using visible light. Traditionally, this field has been dominated by heavy transition metal‐based catalysts, particularly ruthenium and iridium complexes,[^6^, ^7^] which are renowned for their efficiency across a broad range of photochemical transformations. However, increasing environmental and economic concerns have prompted a shift toward more sustainable alternatives, such as 3d‐transition metal complexes[^8^, ^9^, ^10^] and organic photocatalysts,[^11^, ^12^, ^13^, ^14^, ^15^, ^16^, ^17^] which offer greater abundance, cost‐effectiveness, and a lower environmental impact. Moreover, recent innovations have shown that organic photocatalysis not only complements rare‐earth metal‐based catalysts but also significantly broadens the scope of photoredox transformations. For instance, innovative strategies such as consecutive photoinduced electron transfer (conPET)[^12^, ^14^, ^18^] in both reductive[^12^, ^14^, ^18^] and oxidative pathways,^[19]^ electro‐photochemistry,[^20^, ^21^, ^22^] and anion excitation mediated by organic dyes[^23^, ^24^, ^25^, ^26^, ^27^, ^28^] have enabled transformations that were previously difficult to achieve, all under very mild reaction conditions.

Despite these advancements, a significant challenge persists: selecting the appropriate photocatalyst scaffold for specific redox transformations. This is due to the distinct chemical reactivities, photoredox capabilities (particularly redox windows), and compatibility with reaction conditions that different photocatalysts exhibit. For example, the photophysical properties of organic photocatalysts like eosin Y can vary dramatically with pH,^[29]^ while quinolinium and triarylpyrylium dyes may be deactivated by nucleophiles such as amines, acetates, phosphates, or cyanide ions.[^11^, ^30^, ^31^] This complicates the selection of suitable photocatalysts, often leading to case‐by‐case optimization for efficient catalysis. In some cases, it even necessitates tailoring reaction partners or additives to meet the specific requirements of the catalyst, a crucial step for achieving successful reaction outcomes.^[31]^

Recent strategies have begun to address this limitation by demonstrating that a single scaffold can facilitate both oxidative and reductive transformations under a wide range of redox conditions. For example, Fukuzumi's acridinium‐based dyes,^[32]^ known for their strong oxidation potential, have facilitated challenging transformations, such as the photooxidation of the nitrate anion to generate the reactive nitrate radical (NO_3_^⋅^).[^11^, ^33^] Furthermore, research by Nicewicz and coworkers has shown that these same acridinium‐based dyes can also drive challenging reductive processes,^[18]^ effectively extending the redox window of a single scaffold beyond 5 V.[^18^, ^33^] Similarly, flavins, which were initially employed for oxidative transformations,^[34]^ have proven effective in reductive reactions,[^35^, ^36^] further underscoring the potential of dual‐function scaffolds in photoredox catalysis.

**Scheme 1:**
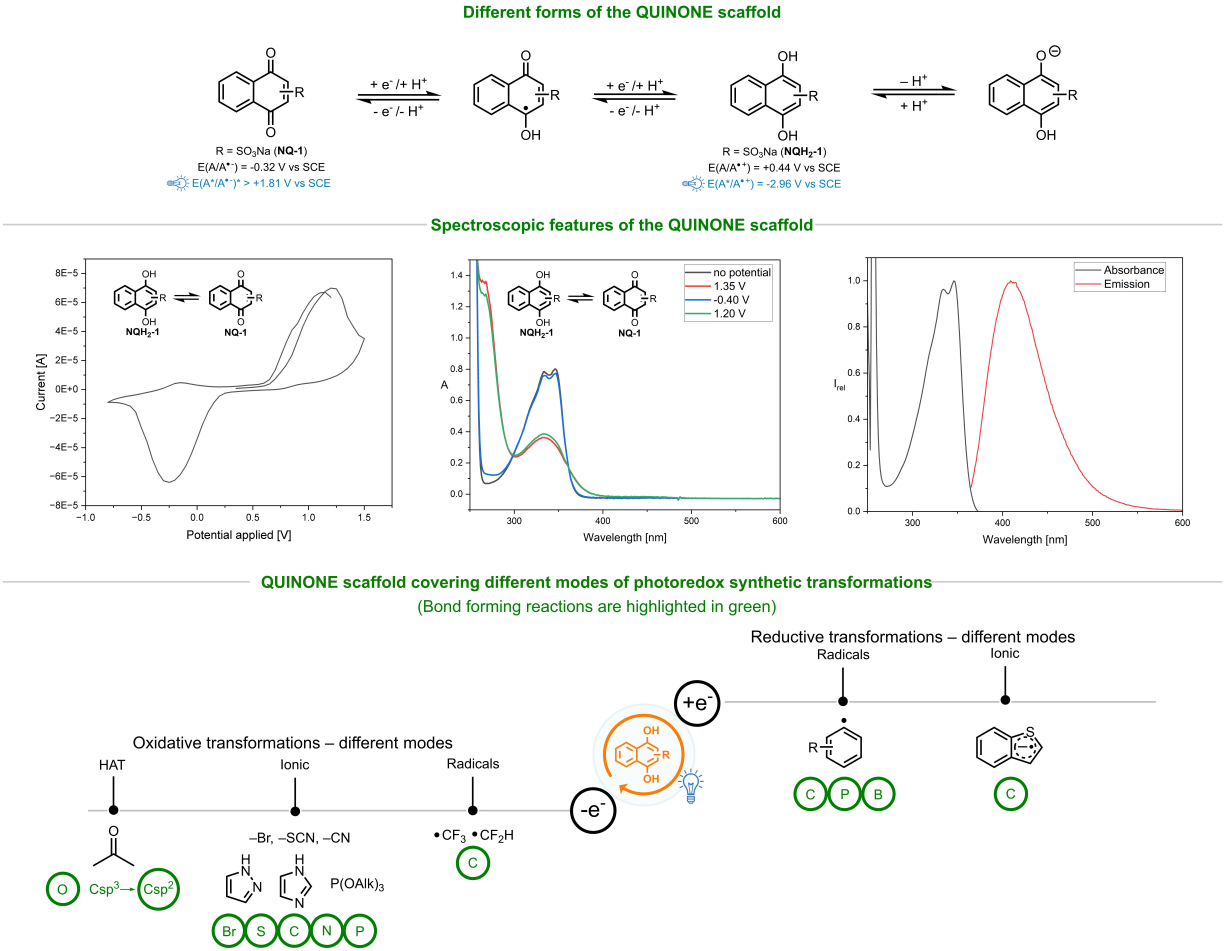
Schematic representation of the quinone scaffold in different forms (top). Cyclic voltammogram, spectroelectrochemical measurements, and absorption and emission spectra of **NQH_2_–1** (middle). Below are the possible synthetic routes covering different modes under photoredox reaction conditions. Reductive transformations are possible both in radical and ionic pathways, oxidative transformations are possible in radical, ionic, and hydrogen atom transfer pathways.

Quinones, a class of naturally occurring chromophores, present another promising scaffold due to their unique properties (Scheme 1). Their inherent redox activity, coupled with facile reversibility between hydroquinone and quinone forms, enables participation in various redox processes.[^37^, ^38^, ^39^, ^40^, ^41^, ^42^, ^43^, ^44^] Quinones also offer extensive opportunities for functionalization, allowing precise tuning of both electronic and steric properties. Additionally, their ability to act as HAT reagent and their compatibility with a broad range of solvents, including aqueous media, further enhance their versatility in diverse chemical transformations.

Given these attributes, we wondered whether quinones could seamlessly adapt to a variety of reaction conditions, facilitating both radical and ionic processes under oxidative and reductive settings while spanning a broad range of redox potentials. Here, we report the potential of quinones as versatile multifunctional scaffolds for photoredox catalysis, capable of mediating radical, ionic, and hydrogen atom transfer processes. By keeping the parameters, i. e. the reaction partners, constant, these transformations can be accomplished under just two reaction conditions: oxidative transformations and HAT reactions proceed in ambient air, while reductive transformations are activated using an inorganic base (Cs_2_CO_3_) through a simple acid‐base deprotonation event.

## Results and Discussion

**Figure 1:**
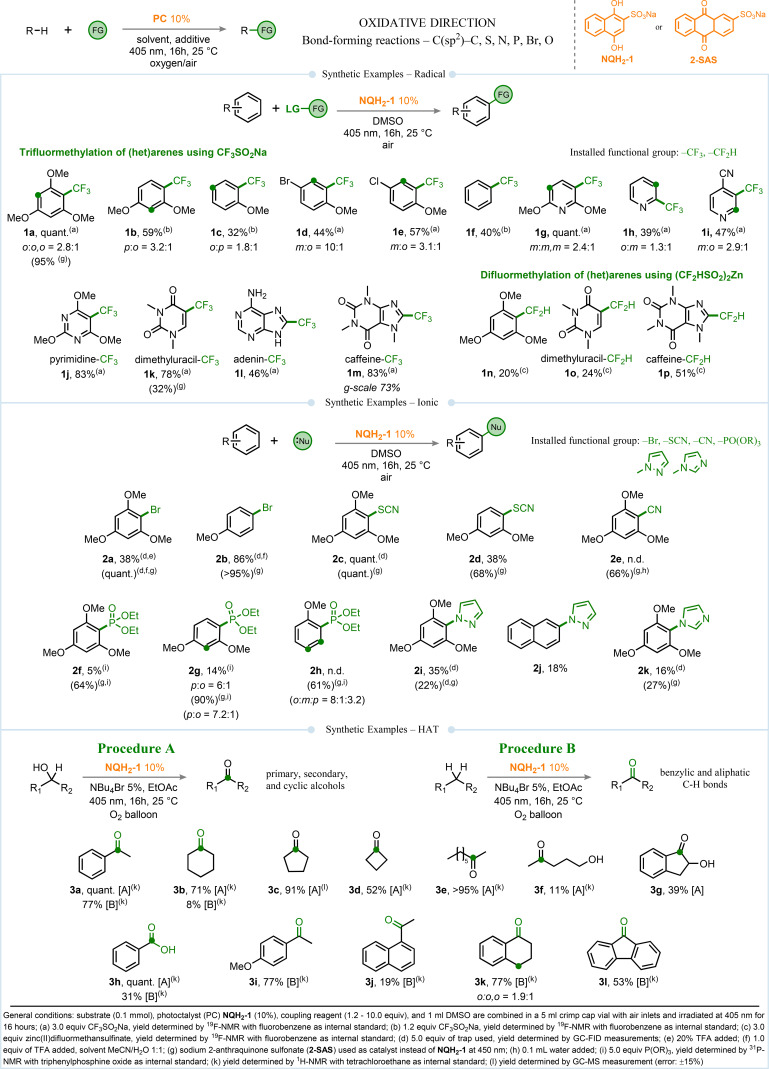
Synthetic examples in oxidative direction using quinones. The transformations include radical and ionic reactions. Synthetic scope for HAT transformations using quinones is also demonstrated.

We began our synthetic investigations under oxidative photoredox reaction conditions, encompassing both radical and ionic transformations (see Figure 1). For the radical pathway, we explored the trifluoromethylation of arenes, particularly focusing on heteroarenes due to the increasing prevalence of the trifluoromethyl group in high‐value pharmaceuticals, agrochemicals, dyes, polymers, and liquid crystals. Employing 1,4‐naphthohydroquinone‐2‐sodium sulfonate (**NQH_2_–1**) as a photocatalyst allowed efficient trifluoromethylation of arenes using commercially available sodium trifluoromethanesulfinate (*E*_ox_=1.05 V vs SCE),^[45]^ yielding the desired products (**1 a**–**1 m**) in good to excellent yields. Noteworthy are the remarkably straightforward reaction conditions requiring only mixing the reactants and irradiating them under ambient air. Control experiments confirmed the necessity of each component (in this case, photocatalyst and light) for successful transformation (see Tables S1–2 in the supporting information for further details). Furthermore, trifluoromethylation was not limited to arenes; heteroarenes were similarly functionalized, achieving the desired products in good yields. Beyond trifluoromethanesulfinate salts, the use of zinc difluoromethanesulfinate salts^[46]^ (*E*_ox_=1.35 V vs SCE^[47]^) facilitated the incorporation of CF_2_H groups into arenes and (het)arenes. Notably, the CF_2_H moiety, known for its lipophilic hydrogen‐bond donor properties, is valuable in bioisostere design, mimicking functional groups such as thiols, hydroxamic acids, hydroxyls, and amides.[^46^, ^48^] Additionally, applying this methodology to biologically relevant pyridines and pyrimidines^[49]^ with various substitution patterns resulted in synthetically useful to good yields of the desired products. Biologically important molecules such as RNA base analog dimethyluracil, adenine, and caffeine, all showed excellent reactivity toward trifluoromethylation, yielding the respective products, in good to excellent yields. To our delight, very similar reactivity and product yields were obtained when the reactions were performed on gram scales (product **1 m**, Figure 1). The simplicity of the reaction conditions allowed us to perform the gram scale reaction just in a crystallizing dish (see Figure 4 and supporting information for further details).

The versatility of naphthoquinone as a scaffold in oxidative transformations extends beyond radical reactions. Under almost identical reaction conditions, it also facilitates the utilization of simple nucleophilic molecules or common inorganic salts for ionic reactions, enabling the introduction of valuable functional groups onto arenes. The use of neutral molecules such as pyrazole and imidazole enables synthetically important C(sp^2^)−N bond‐forming reactions. Similarly, anionic nucleophiles prove effective in functionalizing (het)arenes; for instance, using KBr facilitates bromination (**2 a**), while NaSCN leads to the formation of the desired product (**2 c**–**2 d**) in excellent yields.^[50]^ Additionally, utilizing NaCN as the salt results in the installation of the –CN group onto arenes,[^51^, ^52^] which can be readily transformed into other functional groups under diverse reaction conditions. Control experiments confirmed the necessity of all components for efficient photochemical reactions (cf. Table S3 in the supporting information for additional details).

Recognizing the effectiveness of the substituted quinone, we next explored another key aspect of its catalytic capability: hydrogen atom transfer (HAT) reactions. It is important to note that hydrogen atom abstraction is fundamentally a combined one‐electron oxidation and deprotonation process. Our investigations into this facet led to the formation of products **3 a**–**3 l** up to near‐quantitative yields, as illustrated in Figure 1. Notably, the photochemical conditions enabled the synthesis of various four‐ to six‐membered cyclic ketones, highlighting the versatility of the quinone‐based photocatalyst. Furthermore, the reaction conditions facilitated the synthesis of key molecules, such as compound **3 g** from indane‐1,2‐diol. Additionally, the reaction efficiently converted benzyl alcohol and toluene into benzoic acid, achieving a quantitative yield from the alcohol starting material and 31 % from toluene. To our delight, the quinone scaffold demonstrated the ability also to activate starting materials with a wide range of bond dissociation energies for the respective C−H bonds. Benzylic (**3 a**, **3 h**–**3 l**), aliphatic (**3 b**), and *α*‐OH hydrogen atoms (**3 a**–**3 h**) were successfully abstracted to yield the corresponding photooxidized ketone or acid compound.

Given the inherent characteristics of the quinone moiety, we next explored reduction pathways, which only required slight modifications to the reaction conditions – i. e., the use of an inorganic base, Cs_2_CO_3_ – enabling access to photochemical transformations in both radical and ionic pathways. In the radical direction, we focused on the activation of (het)aryl halides, particularly aryl chlorides, due to their abundance, cost‐effectiveness, more negative reduction potentials, and slower two‐step kinetics^[53]^ compared to their iodide or bromide analogs, making them challenging for synthetic transformations. Using **NQH_2_–1** as a catalyst in DMSO with Cs_2_CO_3_ enabled the use of chloroarenes for various bond‐forming transformations. For example, using 2‐chlorobenzotrifluoride as the chloride substrate with biologically relevant (het)arenes *N*‐methylpyrrole and a benchmark trapping reagent for (het)aryl radicals yielded the desired C−H arylated product **4 a** with a satisfactory 55 % isolated yield from the CF_3_‐substituted aryl chloride. Additionally, electron‐neutral aryl chlorides like chlorobenzene, and more challenging substrates such as p‐methoxy aryl bromide, also afforded the desired products in synthetically useful yields. The formation of product **4 d**, when 1,1‐diphenylethylene was used as the coupling reagent, confirms the radical nature of the reaction. To our delight, these reductive transformations are not limited to C(sp^2^)−C(sp^2^) bond‐forming reactions but also enable synthetically important C(sp^2^)−B and C(sp^2^)−P bond‐forming reactions, using simple reagents such as B₂Pin₂ or trialkyl phosphites. In these transformations, chloroarenes serve as cost‐effective starting materials, further enhancing the practicality of the methodology. Notably, the photochemical reduction reactions were also found to be effective for ortho‐substituted chlorinated electrophiles, which are often problematic in traditional cross‐coupling reactions. These challenging substrates yielded the desired products in good yields, highlighting the versatility and broad applicability of this approach.

Similarly, while the activation of C(sp^2^)−Cl bonds via a radical pathway enables the regioselective installation of arene or heteroarene moieties, the reductive ionic transformation facilitates innate functionalization of C−H bonds in (het)arenes. For our initial evaluation of the photocatalytic system, we selected the reductive activation of C(sp^2^)−H bonds in benzothiophene (*E*_red_=ca. −2.84 V vs SCE)^[54]^ as the test substrate, followed by trapping with simple acetone (as solvent) to form synthetically important C−C bonds. Traditionally, such reactions require Grignard reagents and organometallics. However, utilizing quinone under reductive photoredox conditions allows these transformations to proceed under very mild conditions. This was exemplified by the formation of the desired C−H functionalized product using acetone as the test electrophile in a redox‐neutral fashion, yielding a commendable 81 % isolated yield. The use of other carbonyl compounds, including various cyclic ketones, also facilitated the formation of C−C bonds in synthetically useful yields. Notably, employing cyclic ketones such as cyclohexanone and cyclobutanone in C(sp^2^)−H coupling reactions resulted in the introduction of six‐ and four‐membered OH‐functionalized rings, respectively. Furthermore, the use of CO_2_ as an electrophile yielded the desired carboxylic acid (product **5 b**) in a synthetically useful yield.

The application of the quinone scaffold is particularly noteworthy for its complementarity with photoredox transformations, which manifests in two distinct directions: (1) It enables the installation of diverse functional groups in innate or regioselective transformations. For instance, the use of trialkylphosphite under oxidative reaction conditions enables the selective installation of the desired moiety at the most reactive position via C−H functionalization reactions. Conversely, employing aryl halides under reductive reaction conditions grants access to aryl phosphites through regiospecific transformations (compare products **2 h** and **4 p**). (2) It allows the installation of functional groups that can be subsequently modified through additional functionalization. More significant are the examples of the second scenario: The application of the quinone scaffold covering a wide redox range, combined with the simplicity of the reaction conditions – using air for oxidative transformations and an inorganic base for reductive transformations – facilitated synthetically important bifunctionalization reactions that forge one or two different chemical bonds, enabling the rapid construction of molecular complexity from simple starting materials. In this comprehensive synthetic approach, starting from readily available precursors, the quinone scaffold allows both oxidative and reductive transformations (see Figure 3). As illustrated, oxidative reactions conducted under ambient air conditions enable the straightforward installation of functional groups onto (het)arenes, such as bromination using KBr as an inorganic salt.^[52]^ Conversely, the use of Cs_2_CO_3_ under a nitrogen atmosphere triggers the activation of C(sp^2^)−Br bonds, facilitating the introduction of diverse functional groups, including C−C and C−P bonds.

While the functionalized quinones are easily synthesized, naturally occurring quinones, such as Menadione (**NQ‐2**), Lawson (**NQ‐3**), Juglone (**NQ‐4**), and their respective hydroquinone forms, are also appropriate for both oxidative and reductive transformations. This is demonstrated by the trifluoromethylations of (het)arenes and C−H functionalizations of (het)arenes using aryl chlorides, which result in the formation of the desired products in synthetically useful to high yields, further demonstrating the simplicity and efficiency of using quinone‐based catalytic systems (Figure 4, top). Additionally, the simplicity of the photochemical transformations allows for easy scalability, consistently yielding the desired product. For example, the trifluoromethylation of caffeine can be performed under air, resulting in the formation of the desired products in a 73 % isolated yield (Figure 4, bottom). Furthermore, the adaptability of the quinone scaffold extends beyond substituted naphthoquinone to include substituted anthraquinone (sodium 2‐anthraquinone sulfonate (**2‐SAS**)),^[51]^ as exemplified by synthetic transformations yielding trifluormethylated (**1 a, 1 k**) and phosphonated (**2 a–i, 2 k**) products under oxidative reaction conditions.

**Figure 2:**
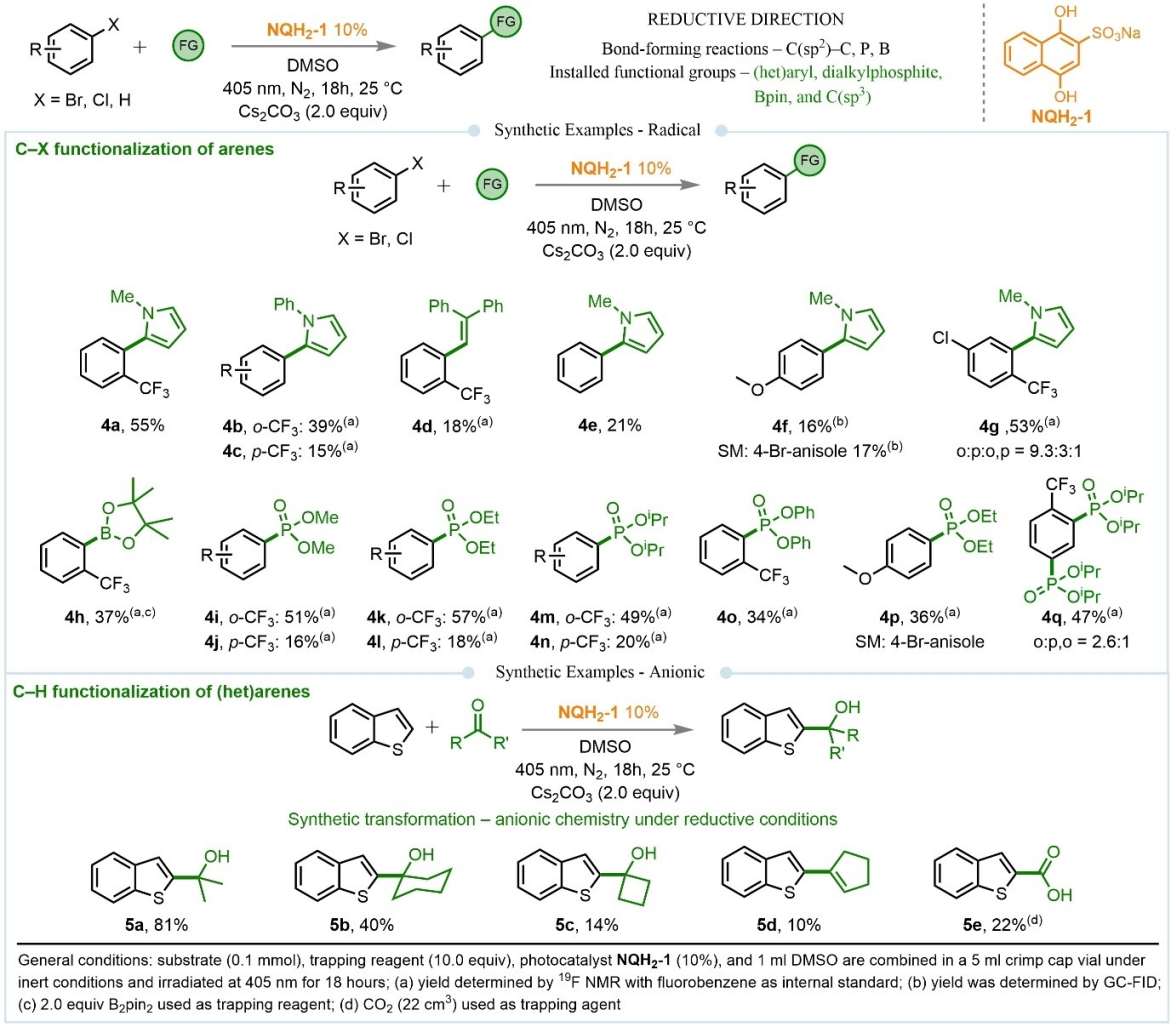
Synthetic examples in reductive direction using quinones. The transformation includes radical and ionic reactions.

**Figure 3:**
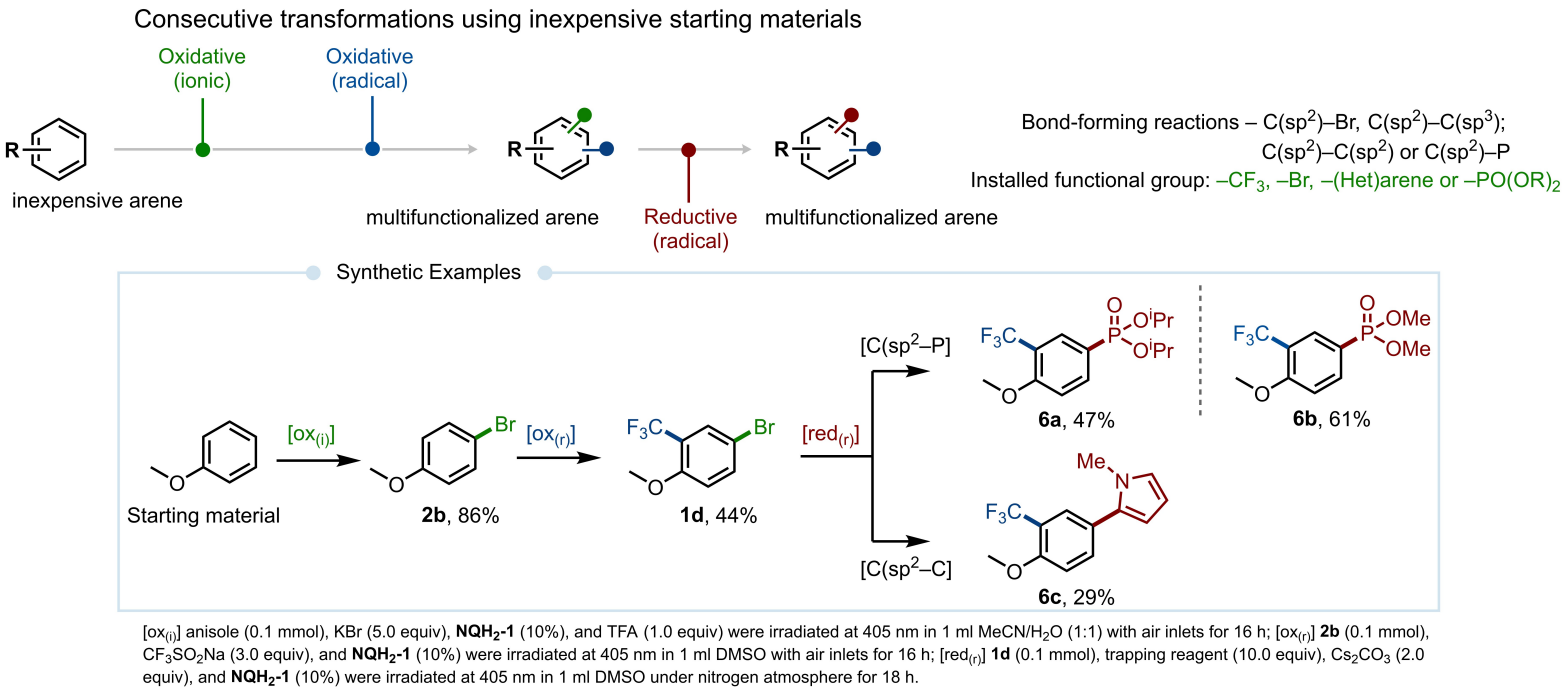
The ability of the quinone scaffold to undergo successive transformations covering different modes of photoredox transformations with visible light.

**Figure 4:**
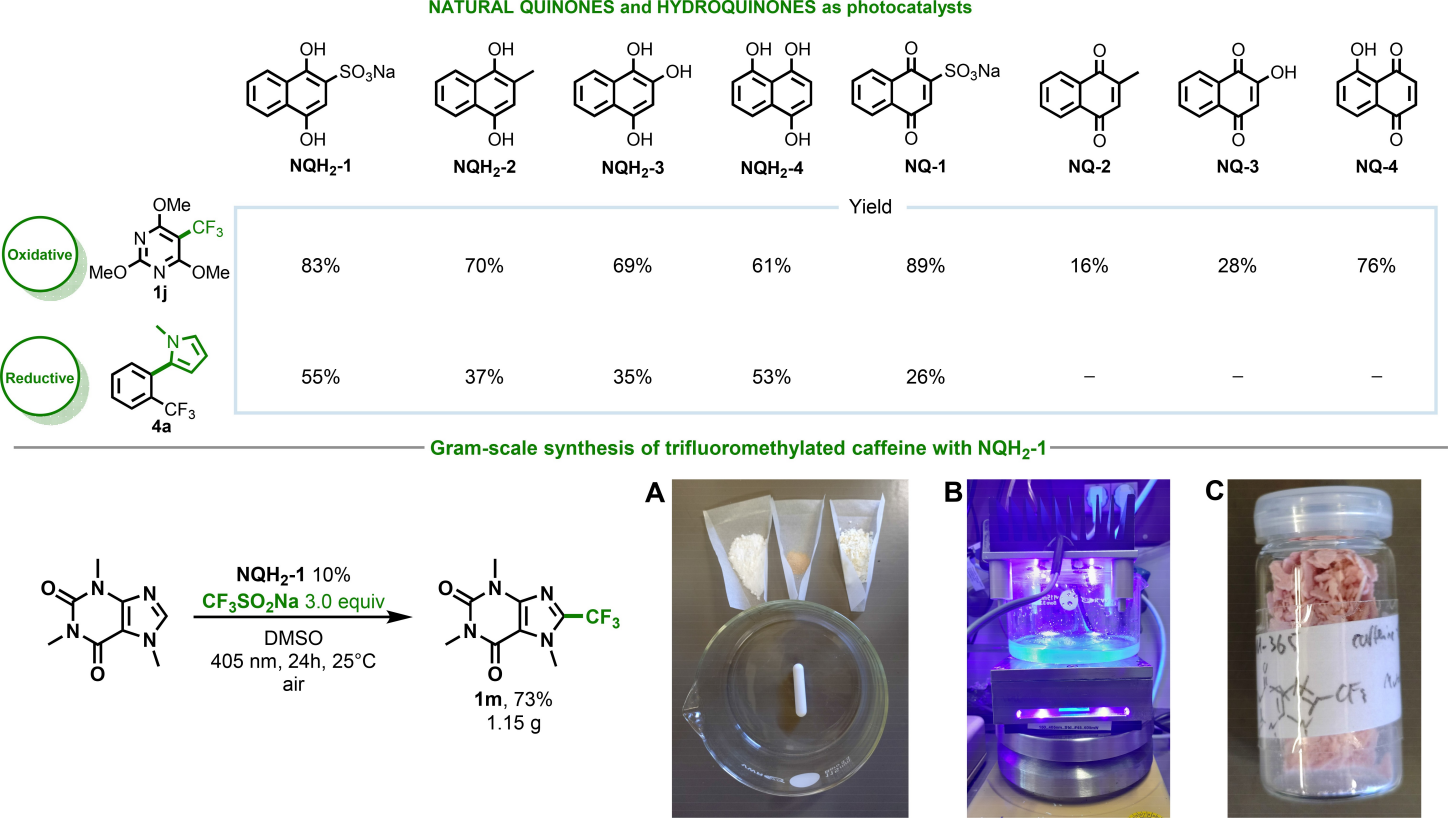
The use of naturally occurring quinones and hydroquinones in photoredox trifluoromethylation and reductive activation of aryl halides for C(sp^2^)−C(sp^2^) bond formation reactions and gram‐scale trifluoromethylation of caffeine under air (depicted in **A**, **B**, and **C**): (A) required reagents and reaction vessel, (B) photochemical setup, (C) isolated product.

**Figure 5:**
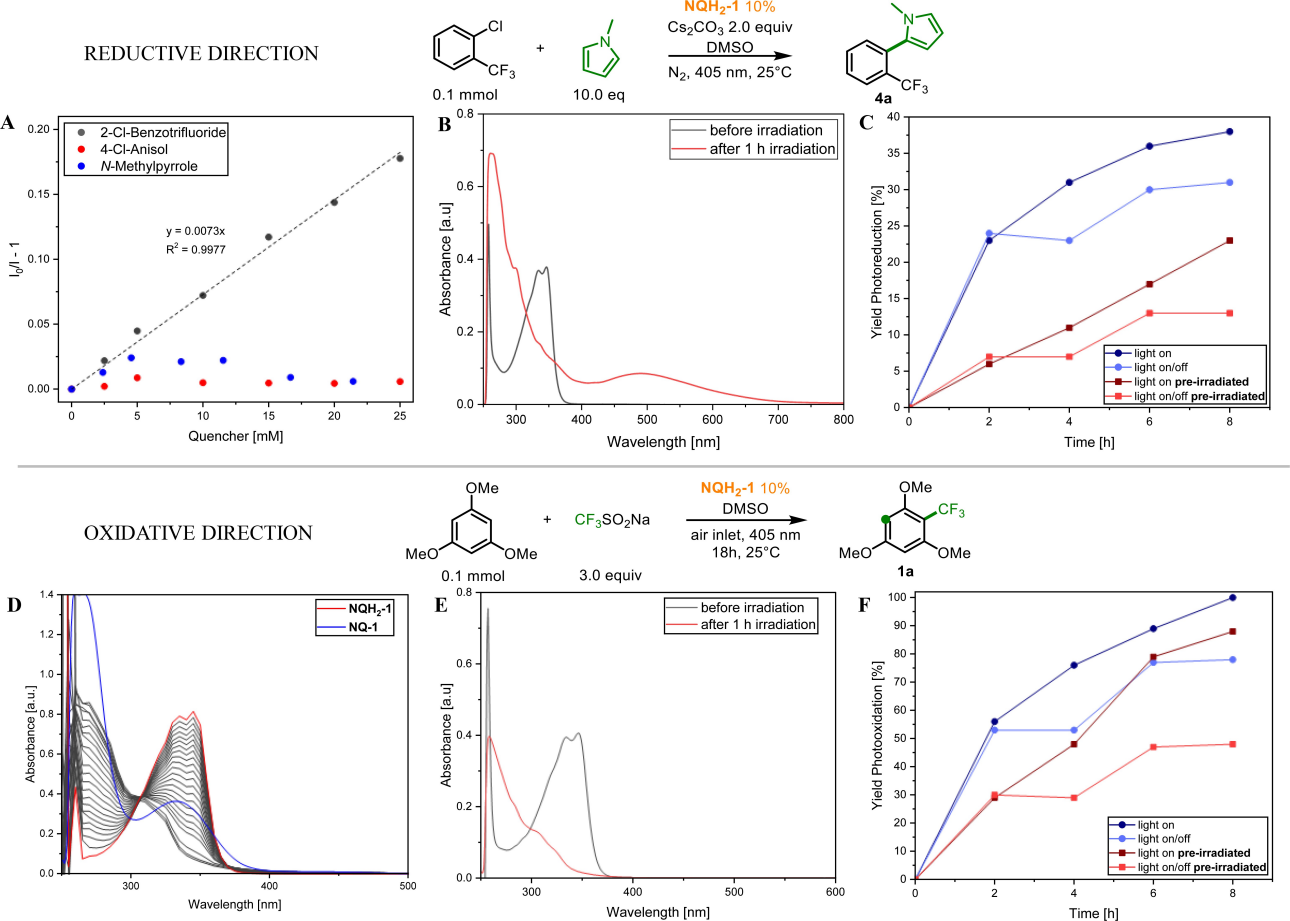
Mechanistic investigations on the reductive (top) and oxidative (down) reaction pathways. Luminescence quenching of **NQH_2_–1** with aryl halides without the presence of base (A). Transformed photocatalytic mixture upon photoirradiation (B) and light on/off experiments in the reductive pathway (C). Photoirradiation experiment of **NQH_2_–1** under oxygen in DMSO (D). Transformed photocatalytic mixture upon photoirradiation (E) and light on/off experiments in the oxidative pathway (F).

Quinones, owing to their inherent redox properties, are capable of facilitating a broad range of chemical transformations (*cf*., Figures 2, 3, 4). However, the presence of various photo‐ and redox‐active species, including all intermediates in the reaction mixture under synthetic conditions, combined with the challenges of performing spectroscopic and electrochemical experiments in non‐idealized environments, hinders the development of a comprehensive mechanistic proposal for these transformations. This includes processes involving oxidation, reduction, and hydrogen atom transfer (HAT), encompassing both radical and ionic pathways. That said, the experimental data provide strong evidence for the photocatalytic nature of the reaction, as supported by control experiments (see Section 5 in the supporting information) and the kinetics of product formation under light on/off reaction conditions (*cf*., Figure 5C and F for reductive and oxidative transformations respectively and Section 6 in the supporting information for further details). The overall quantum yield of both processes was also found to be on the order of 1 % or less (see Section 6.4 in the supporting information), ruling out the possibility of radical chain mechanisms.

In the reductive pathway, spectroscopic investigations revealed that the addition of 2‐Cl‐benzotrifluoride to a solution containing **NQH_2_–1** did not result in significant changes to the absorption spectra. However, a notable decrease in luminescence lifetime and intensity was observed, indicating the possibility of electron transfer from the excited state of **NQH_2_–1** to the substrate. In contrast, no comparable changes in luminescence were detected when substrates with more negative reduction potentials, such as 4‐Cl‐anisole, were introduced. These findings suggest that, in the latter case, a deprotonated photocatalyst, formed in the presence of Cs₂CO₃, or a phototransformed catalyst (discussed below), plays a critical role in facilitating the single‐electron transfer (SET) required for substrate activation. It is worth noting that direct characterization of the luminescence spectra of the deprotonated catalyst proved difficult, likely due to a dynamic equilibrium between protonated and deprotonated species or the inherently low luminescence quantum yield of the deprotonated form.

In the oxidative pathway, **NQ‐1**, **NQ‐2**, and related quinones were evaluated as photocatalysts, yielding the desired product in up to 89 % yield (see Figure 4). Spectroelectrochemical measurements revealed a reversible redox peak corresponding to the interconversion between hydroquinone and quinone forms, with near‐complete reversibility. Surprisingly, under photochemical conditions with **NQH_2_–1** as the initial photocatalyst in the presence of oxygen, the quinone form–predicted from the absorption spectra (see Figure 5)–was not observed. This unexpected result led to the hypothesis that, while the reaction may initially proceed via the identified photocatalyst, prolonged photoirradiation generates multiple photocatalytically active species that collectively drive the transformation. Kinetic experiments and product formation studies demonstrated that photoirradiation of the initial photocatalyst led to the formation of distinct species that continued to facilitate product formation (*cf*., Figure 5F and Section 6.1 in the supporting information), albeit at a lower reaction rate. However, the exact identity of these species and their relative contributions to the transformation remain unresolved.

Overall, photoirradiation of **NQH_2_–1** under both oxidative and reductive conditions generates multiple photocatalytically active species. The interplay of redox transformations, protonation‐deprotonation equilibria, and photochemical reactions likely gives rise to a dynamic ensemble of reactive intermediates that function either independently or synergistically. While this complexity complicates a detailed mechanistic interpretation, it underscores the broad applicability of the system, as distinct species can operate effectively under a wide range of redox conditions.

The oxidative HAT reactions involving photoexcited quinone scaffold in the presence of oxygen likely proceed via a mechanism reported in the literature,[^55^, ^56^] initiated by hydrogen atom abstraction (see section 6.2 in the supporting information for further details). This step generates a semi‐hydroquinone species and a carbon‐centered radical. The carbon‐centered radical subsequently reacts with oxygen to form a peroxy radical, which oxidizes the semi‐hydroquinone back to its original form via HAT. The process culminates in the formation of an organic peroxide, which upon decomposition yields the desired oxidized product.

## Conclusions

We report the applicability of quinone scaffolds in light‐driven redox transformations, offering broad coverage across a wide range of redox potentials. The versatility of the quinone scaffold allows for seamless adaptation to diverse reaction conditions, enabling both radical and ionic processes under oxidative and reductive environments. By maintaining simple, consistent parameters–oxidative transformations performed under ambient air and reductive transformations facilitated by chromophore activation in the presence of Cs_2_CO_3_–this approach provides a streamlined methodology for achieving complex chemical transformations. The ease of synthesis, natural abundance, scalability, and remarkable versatility of quinones make them an invaluable addition to the toolbox of chromophores that also allows easy access to a wide range of redox potentials for synthetic transformations, which not only enhances the use of quinones in existing transformations but also encourages systematic exploration of other chromophores with similar redox versatility for future applications in synthetic chemistry.

## Conflict of Interests

The authors declare that they have no competing interests.

1

## Supporting information

As a service to our authors and readers, this journal provides supporting information supplied by the authors. Such materials are peer reviewed and may be re‐organized for online delivery, but are not copy‐edited or typeset. Technical support issues arising from supporting information (other than missing files) should be addressed to the authors.

Supporting Information

## Data Availability

All data are available in the main text or the ESI.^†^ The primary research data is provided under DOI: 10.22000/pmprxn5p2syh0rrq.
