# Supplementary material for: Quinones as Multifunctional Scaffolds for Oxidative, Reductive, and HAT Photocatalysis
Source: Chemistry. 2025 Mar 2;31(20):e202404707. doi: 10.1002/chem.202404707 (PMC11973854; doi:10.1002/chem.202404707)
Supplement: Supplementary file 1 — Supporting Information [file CHEM-31-e202404707-s001.pdf]

Chemistry–A European Journal

Supporting Information

Quinones as Multifunctional Scaffolds for Oxidative, Reductive, and HAT Photocatalysis

Lea Müller, Jonas Poll, Patrick Nuernberger, Indrajit Ghosh,* and Burkhard König*

Electronic Supplementary Information

Quinones as Multifunctional Scaffolds for Oxidative, Reductive, and HAT Photocatalysis

Lea Müller[1], Jonas Poll[2], Patrick Nuernberger[2], Indrajit Ghosh[1,3,*], and Burkhard König[1,*]
[1]Fakultät für Chemie und Pharmazie, Universität Regensburg, 93053 Regensburg, Germany
[2]Institut für Physikalische und Theoretische Chemie, Universität Regensburg, 93053 Regensburg, Germany
[3]Nanotechnology Centre, Centre for Energy and Environmental Technologies, VSB –Technical University of Ostrava, 708 00 Ostrava-Poruba, Czech Republic
The correspondence may be addressed to: Indrajit1.ghosh@ur.de (I.G.) and burkhard.koenig@ur.de (B.K.)

The primary research data is provided under DOI: 10.22000/pmprxn5p2syh0rrq

Table of contents

1. General Information 1
2. Photochemical reactor setups 3
3. Synthesis of photocatalysts 5
4. General photocatalytic procedures 7
5. Reaction monitoring and optimization 10
   5.1 Oxidative transformation 10
   5.2 HAT-based oxidative transformations 13
   5.3 Reductive transformations 14
6. Mechanistic discussion 16
   6.1 Oxidative direction 16
   6.2 HAT direction 17
   6.3 Reductive direction 17
   6.4 Quantum yield measurement 20
7. Additional spectroscopic investigations 22
   7.1 UV-Vis absorption 22
   7.2 Emission spectra 23
   7.3 Stern-Volmer Quenching 23
   7.4 Cyclic voltammetry of catalysts 29
   7.5 Cyclic voltammetry of starting materials 35
   7.6 Spectroelectrochemistry of catalyst NQH_2-1 36
   7.7 Photostability measurements 37
8. Characterization of the products 39
   8.1 Photooxidative transformations 39
   8.2 Photocatalytic HAT reactions 51
   8.3 Photoreductive transformations 55
9. NMR spectra 67
10. References 159

1. General Information

Solvents and Chemicals: Commercially available chemicals were purchased at the highest available quality and used without further purification unless noted otherwise. Quinones were synthesized following reported procedures or modified versions thereof (see Section 3). Commercially purchased discolored N-methylpyrrole was filtered through basic alumina oxide until clear to remove oxidized byproducts before use. The photochemical reactions were conducted in dimethyl sulfoxide (DMSO) and acetonitrile (MeCN), which were dried over 4 Å molecular sieves according to established protocols.[1]

NMR Spectroscopy: All NMR spectra were measured using either a Bruker Avance 300 MHz (^{1}H: 300 MHz, ^{13}C: 75 MHz) or a Bruker Avance III HD 400 MHz spectrometer (^{1}H: 400 MHz, ^{13}C: 101 MHz, ^{19}F: 376 MHz, ^{31}P: 162 MHz). Chemical shifts are reported in δ [ppm], relative to an internal standard or the residual solvent peak, with coupling constants (J) provided in Hertz [Hz]. Signal multiplicities are abbreviated as follows: s = singlet, d = doublet, t = triplet, m = multiplet, dd = doublet of doublets, td = triplet of doublets, ddd = doublet of doublet of doublets, b = broad singlet.

Gas Chromatography (GC) and GC-MS: Gas chromatography with a flame-ionization detector (GC-FID) and gas chromatography coupled to low-resolution mass spectrometry (GC-MS) were performed using a capillary column (length: 30 m; diameter: 0.25 mm; film thickness: 0.25 µm) with helium as the carrier gas. GC-MS analysis was conducted on a 5975 MSD single quadrupole detector. The standard heating procedure began with an initial temperature of 40 °C, held for 3 minutes. The temperature was then ramped up to 280 °C at 15 °C/min and maintained for 5 minutes, followed by a final increase to 300 °C at 25 °C/min.

High-resolution mass spectrometry: High-resolution mass spectra (HRMS) were acquired at the central analytical mass spectrometry facilities of the Faculty of Chemistry and Pharmacy, University of Regensburg, using either a JEOL AccuTOF GCX or an Agilent Q-TOF 6540 UHD instrument.

UV-Vis Absorption Spectroscopy: Absorption spectra were measured at room temperature using either an Agilent Cary 60 UV-Vis spectrophotometer or an Agilent 8453 UV-Vis spectrometer with a 10 mm Hellma quartz fluorescence cuvette or a 10 mm Starna Spectrosil Far UV Quartz cuvette.

Fluorescence Spectroscopy: Fluorescence absorption and emission measurements were conducted using a HORIBA Fluoromax-4 spectrofluorometer or a Horiba Fluorolog-3 spectrofluorometer with FluorEssence v3.9 software.

For time-resolved emission spectra, a home-built TCSPC-setup was used.[2] The excitation source was a Horiba NanoLED-370 centered around 367 nm and the emission was recorded at 410 nm. Measurements were performed in a 10 mm Hellma quartz fluorescence cuvette or a 10 mm Starna Spectrosil Far UV Quartz cuvette.

Cyclic voltammetry: Cyclic voltammetry (CV) measurements were conducted using a Metrohm Autolab PGSTAT302N three-electrode potentiostat/galvanostat. A glassy carbon electrode was used as the working electrode, a platinum wire as the counter electrode, and a silver wire as the reference electrode, with 0.1 M tetrabutylammonium tetrafluoroborate (Fluka) as the supporting electrolyte. The instrument control, data acquisition, and processing were performed using Metrohm Autolab NOVA 1.10.4 software. Prior to measurement, the solvent was degassed with argon, and all experiments were carried out under an argon atmosphere at a scan rate of 0.05 $V \cdot s^{-1}$. Ferrocene was used as an internal reference to determine the reduction and oxidation potentials, and all potentials are reported relative to the saturated calomel electrode (SCE).

Spectroelectrochemistry: The measurements followed the procedure for cyclic voltammetry. A platinum minigrid electrode (32 wires/cm) was used as working electrode and counter electrode, and a silver microwire as reference electrode. The UV-Vis spectra were in parallel measured using an Agilent 8453 UV-Vis spectrometer with a 0.2 mm Ottle Cell (optically transparent thin-layer electrochemical cell) from Frantisek Hartl (University of Reading) with optically transparent CaF_2 windows.

LEDs: For the photochemical reactions 405 nm Mouser/LEDTECH LST1-01G01-UV04-00 LEDs (405 nm, λ_{max} = 408 (±30) nm, I_{max} = 700 mA, radiant power ~ 605 mW), 405 nm LEDs Nichia - NVSU233B UV (405 nm, λ_{max} = 405 (±30) nm, I_{max} = 700 mA, radiant power ~ 413 mW), and 450 nm Oslon SSL 80 LDCQ7P-2U3U LT-1960 royal-blue (451, λ_{max} = 442 (±30) nm, I_{max} = 700 mA, radiant power ~ 658 mW) were used.

2. Photochemical reactor setups

Small-scale room temperature photoreactions

Photoreactions were performed in the reactor depicted in Figure S1. Reagents were placed in 5 ml crimp-capped vials. The vials fit into a cooling block kept at 25 °C by a thermostat. The vials were irradiated from below *via* Mouser/LEDTECH LST1-01G01-UV04-00 LEDs (λ_{max} = 408 nm) or OSRAM Oslon SSL 80 LT-1960 royal-blue LEDs (λ_{max} = 442 nm). Stirring was achieved *via* a magnetic stirrer placed below the reactor. Although the emission maximum is reported at 400 – 405 nm by the supplier, it was determined to be at 408 nm with a range of approximately 370 – 460 nm. Similarly, the emission of the 450 nm LED is reported at 451 nm by the supplier but was determined to be at 441 nm with a range of approximately 410 – 500 nm (Figure S1, bottom).

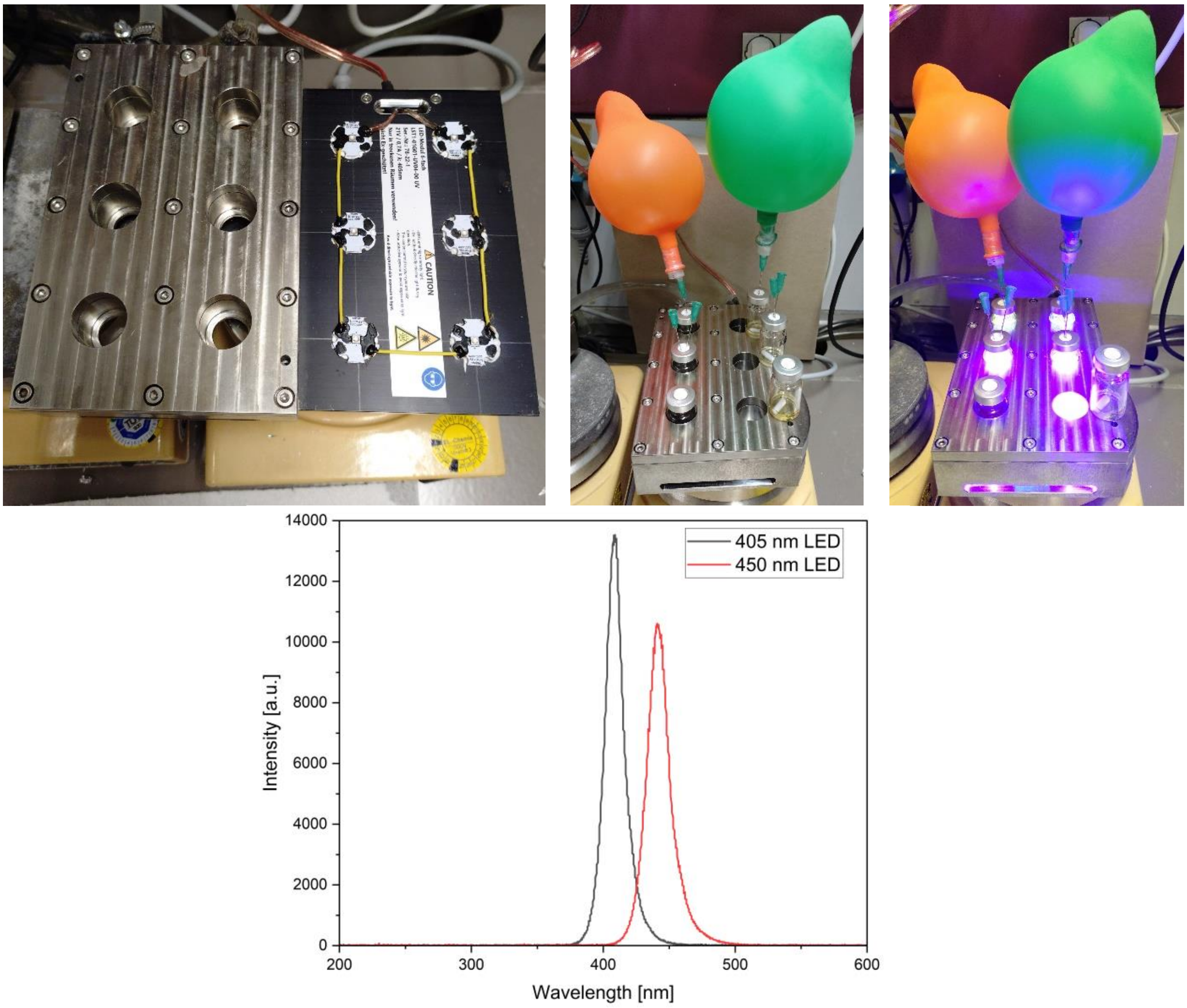

Figure S1. **Photographs of the photochemical reaction set-ups**. Top: Photograph of the typical and LED module (left), photoreactor setup (middle), and photoreactor setup in operation (right). Five ml crimp cap vials were illuminated from the bottom side with LEDs (in this case with 405 nm LEDs: λ = 408(± 30) nm, ~650 mW) and the temperatures were maintained at 25 °C unless noted otherwise from the bottom and side using custom-made aluminum cooling blocks connected to a thermostat. Bottom: **Emission spectra of LEDs**. Emission spectra of the 405 nm LEDs (black) and the 450 nm LEDs (red).

Large-scale room temperature photoreactions

Large-scale room temperature photoreactions were performed in the setup depicted in Figure S2. All reagents and the solvent were put into the crystallizing dish (~140 mm diameter) together with a stirring bar. Reactions were irradiated by 4 LEDs from the top (Mouser/LEDTECH LST1-01G01-UV04-00, λ_{max} = 408 nm, 0.65 Watt) and 4 LEDs from the bottom. The temperature was controlled via a cooling block kept at 25°C by a thermostat.

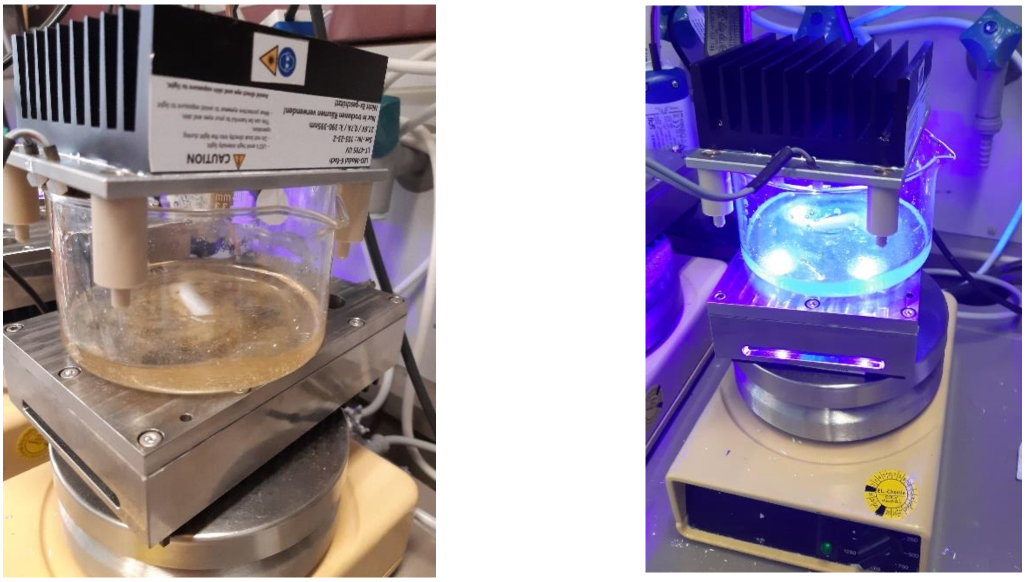

Figure S2. Photograph of the large-scale photoreaction setup without (left) and with illumination (right). The setup was irradiated by a set of 4 LEDs from the top and 4 LEDs from the bottom and temperatures were maintained at 25 °C unless noted otherwise from the bottom using a custom-made aluminum cooling block connected to a thermostat.

3. Synthesis of photocatalysts

General procedure for the synthesis of naphthohydroquinone photocatalysts (Procedure A)

The catalysts were synthesized following established literature procedures.[3] The naphthoquinone compound was placed in a separatory funnel, dissolved in 15 ml of ethyl acetate, and purged with nitrogen gas. Sodium dithionite ($Na_2S_2O_4$, 5.0 equiv) was dissolved in 5 ml of water and added to the organic solution. The mixture was stirred vigorously for 30 minutes, and the reaction progress was monitored by thin-layer chromatography (TLC). If necessary, an additional 5.0 equiv of $Na_2S_2O_4$ were added, and stirring was continued for an additional hour. Upon completion of the reaction, the organic phase was separated, and the aqueous phase was extracted with ethyl acetate (3 × 15 ml). The combined organic phases were washed with water and brine, dried over $MgSO_4$, and concentrated under reduced pressure. The product was characterized by NMR spectroscopy.

Sodium 1,4-dihydroxynaphthalene-2-sulfonate (NQH_2-1)

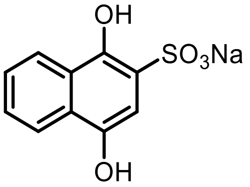

The title compound was synthesized according to a modified literature procedure.[4] 1,4-naphthoquinone (1.50 g, 9.48 mmol) and sodium bisulfite (1.97 g, 19.22 mmol, 2.0 equiv) were placed in a Schlenk flask and backfilled two times with nitrogen. Water (8 ml), degassed by sparging with nitrogen for 20 minutes, was added and the mixture stirred for 90 minutes. Then, activated charcoal (40 mg) was added and the mixture stirred for an additional 30 minutes. The mixture was filtered over celite and concentrated *in vacuo*. The crude mixture was sonicated with 30 ml ethanol and filtered over celite. The filtrate was collected and concentrated *in vacuo*, yielding **NQH_2-1** as off-white powder (1.52 g, 5.79 mmol, 61%).

^{1}H NMR (400 MHz, DMSO-d_6) δ [ppm] = 10.92 (s, 1H), 9.50 (s, 1H), 8.10 (dt, *J* = 7.8, 2.6 Hz, 1H), 8.06 – 7.98 (m, 1H), 7.54 – 7.43 (m, 2H), 6.94 (s, 1H).

^{13}C NMR (101 MHz, DMSO) δ [ppm] = 144.41, 141.77, 126.26, 125.93, 125.45, 125.17, 122.73, 122.68, 121.87, 105.42.

HRMS (ESI): calculated *m/z* for $C_{10}H_7O_5S^-$ [M^-] 239.0020, found 239.0023.

2-methylnaphthalene-1,4-diol (NQH_2-2)

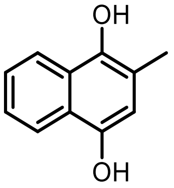

The title compound was synthesized using 2-methyl-1,4-naphthoquinone (0.50 g, 2.90 mmol) as starting material, yielding **NQH_2-2** as green powder (0.39 g, 2.22 mmol, 77%).

^{1}H NMR (300 MHz, DMSO-d_6) δ [ppm] = 9.35 (s, 1H), 8.24 (s, 1H), 8.11 – 8.03 (m, 1H), 8.03 – 7.97 (m, 1H), 7.46 – 7.28 (m, 2H), 6.62 (s, 1H), 2.27 (s, 3H).

^{13}C NMR (101 MHz, DMSO-d_6) δ [ppm] = 145.81, 141.71, 126.51, 124.90, 123.78, 123.52, 121.85, 121.68, 118.72, 111.08, 16.56.

The spectral data was compared to literature values.[3]

Naphthalene-1,2,4-triol (NQH$_2$-3)

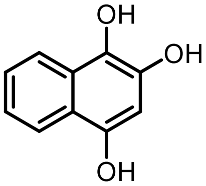

The title compound was synthesized using 2-hydroxy-1,4-naphthoquinone (0.50 g, 2.87 mmol) as starting material, yielding **NQH$_2$-3** as green powder (0.39 g, 2.21 mmol, 88%).
^{1}H NMR (300 MHz, DMSO-d$_6$) δ [ppm] = 9.41 (s, 1H), 9.02 (s, 1H), 7.96 – 7.82 (m, 2H), 7.42 – 7.25 (m, 1H), 7.21 – 7.09 (m, 1H).
^{13}C NMR (101 MHz, DMSO-d$_6$) δ [ppm] = 146.22, 140.57, 129.53, 126.66, 125.00, 121.89, 121.10, 120.63, 119.28, 101.49.
The spectral data was compared to literature values.[5]

Naphthalene-1,4,5-triol (NQH$_2$-4)

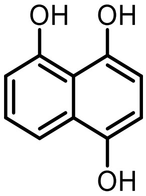

The title compound was synthesized using 5-hydroxy-1,4-naphthoquinone (0.50 g, 2.87 mmol) as starting material, yielding **NQH$_2$-4** as green powder (0.44 g, 2.50 mmol, 87%).
^{1}H NMR (300 MHz, DMSO-d$_6$) δ [ppm] = 10.71 (s, 1H), 10.35 (s, 1H), 9.36 (s, 1H), 7.51 (dd, *J*=8.5, 1.1, 1H), 7.22 (dd, *J*=8.4, 7.5, 1H), 6.71 (dd, *J*=7.5, 1.1, 1H), 6.66 (d, *J*=8.2, 1H), 6.55 (d, *J*=8.1, 1H).
^{13}C NMR (101 MHz, DMSO-d$_6$) δ [ppm] = 154.04, 145.86, 145.72, 127.18, 125.66, 115.11, 113.28, 108.84, 108.45, 108.03.
The spectral data was compared to literature values.[6]

Sodium 1,4-naphthoquinone-2-sulfonate (NQ-1)

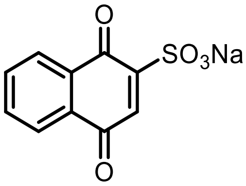

Synthesized according to a modified literature procedure.[4] 1,4-naphthoquinone (1.50 g, 9.48 mmol) and sodium bisulfite (1.97 g, 19.22 mmol, 2.0 equiv) were placed in a Schlenk flask and backfilled two times with nitrogen. Water (8 ml), degassed by sparging with nitrogen for 20 minutes, was added and the mixture stirred for 90 minutes. Then, activated charcoal (40 mg) was added and the mixture stirred for an additional 30 minutes. The mixture was filtered over celite, and the filtrate heated to 60°C. Nitric acid (65%, 2.92 ml, 45.49 mmol, 4.8 equiv) was added dropwise under stirring. Yellow precipitate began to form. The mixture was stirred for an additional 10 minutes and filtrated. The yellow residue was washed with deionized water until neutral and dried to obtain **NQ-1** as yellow powder (0.79 g, 3.04 mmol, 32%).
^{1}H-NMR (400 MHz, DMSO-d$_6$) δ [ppm] = 8.04 – 7.94 (m, 2H), 7.92 – 7.82 (m, 2H), 7.20 (s, 1H).
^{13}C-NMR (101 MHz, DMSO-d$_6$) δ [ppm] = 185.79, 180.89, 150.99, 134.52, 133.94, 133.60, 132.09, 131.43, 126.20, 125.44.
HRMS (ESI): calculated *m/z* for $C_{10}H_5O_5S^-$ [M$^-$] 236.9863, found 236.9868.

4. General photocatalytic procedures

General procedure for the photocatalytic oxidative coupling reactions (Procedure B)

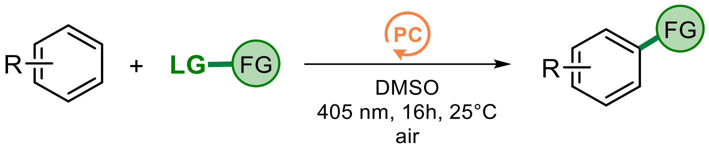

A 5 ml crimp vial, equipped with a stirring bar, was charged with the (hetero)aromatic starting material (0.1 mmol, 1.0 equiv), photocatalyst **NQH$_2$-1** (0.01 mmol, 0.1 equiv), coupling reagent (0.13 – 1.0 mmol, 1.3 – 10.0 equiv), and 1 ml of DMSO. The vial was sealed and fitted with two syringe needles to serve as air inlets. The reaction mixture was irradiated with a 405 nm LED through the flat bottom side of the vial at 25 °C, unless noted otherwise. Following irradiation, the crude reaction mixture was analyzed by ^{19}F NMR and GC-MS. Fluorobenzene (10 µL, 1.0 equiv) was added directly to the vial as an internal standard. A 50 µL aliquot of the reaction mixture was then combined with 0.55 ml of CDCl3 in an NMR tube for analysis.

Note: It is to be noted here that due to the high concentration of DMSO in the crude NMR sample, the ^{19}F NMR peaks were slightly shifted from their literature values. The internal standard is indicated with an asterisk in the NMR spectra.

To isolate the desired product, the reaction mixtures from four vials were combined and transferred to a separatory funnel containing 30 ml of water and 10 ml of brine. The mixture was extracted with ethyl acetate (3 × 15 ml). The combined organic phase was dried over $MgSO_4$ and concentrated under reduced pressure. The crude material was purified by flash column chromatography using petrol ether/ethyl acetate or other suitable solvent mixtures (PE/DCM, DCM/MeOH, EtOAc/MeOH, wherever applicable) as eluents on silica gel.

Large-scale transformations:

The large-scale reactions were performed using the reaction setup depicted in Figure S2. The starting material (6.0 mmol, 1.0 equiv), photocatalyst **NQH$_2$-1** (0.6 mmol, 0.1 equiv), coupling reagent (18.0 mmol, 3.0 equiv), and 60 ml of DMSO were placed in a crystallizing dish equipped with a magnetic stirring bar. The setup was placed on a cooling block and irradiated from top and bottom with sets of four 405 nm LEDs at 25°C for approximately 24 hours.

To isolate the desired product, the reaction mixture was transferred to a separatory funnel containing 100 ml of water and 250 ml of brine. The mixture was extracted with ethyl acetate (4 × 100 ml). The combined organic phase was dried over $MgSO_4$ and concentrated under reduced pressure. The crude material was purified by flash column chromatography using petrol ether/ethyl acetate as eluents on silica gel.

The analysis of the crude reaction mixture by ^{19}F-NMR and GC-MS is showcased by the trifluormethylation of 1,3,5-trimethoxypyrimidine to 2,4,6-trimethoxy-5-(trifluoromethyl)-pyrimidine **1j**. The yield of the desired product was determined by ^{19}F NMR of the crude reaction mixture using fluorobenzene (25 µl, 0.265 mmol) as an internal standard. NMR-yield: 83% (product).

^{19}F NMR (377 MHz, $CDCl_3$) δ [ppm] = -55.78 (product **1j**), -113.15 (fluorobenzene).

The spectral data was compared to literature values.[7]

GC-MS retention time [min] = 2.00 (MeCN, m/z = 41), 2.61 (EtOAc, m/z = 89), 3.03 (standard: fluorobenzene, m/z = 96), 5.60 (DMSO, m/z = 78), 10.36 (1,3,5-trimethoxypyrimidine, m/z = 170), 10.73 (product **1j**, m/z = 238).

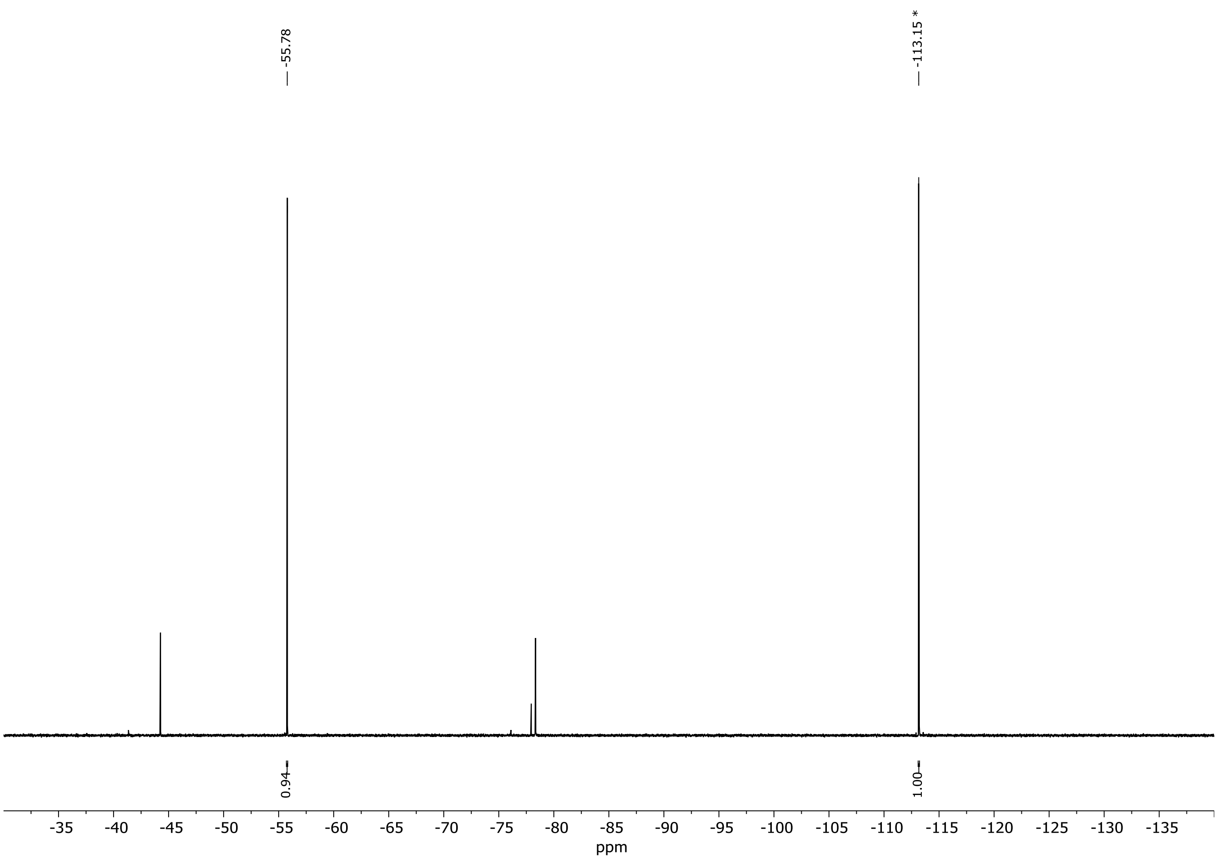

-55.78
-113.15 *
0.94
1.00
-35 -40 -45 -50 -55 -60 -65 -70 -75 -80 -85 -90 -95 -100 -105 -110 -115 -120 -125 -130 -135
ppm

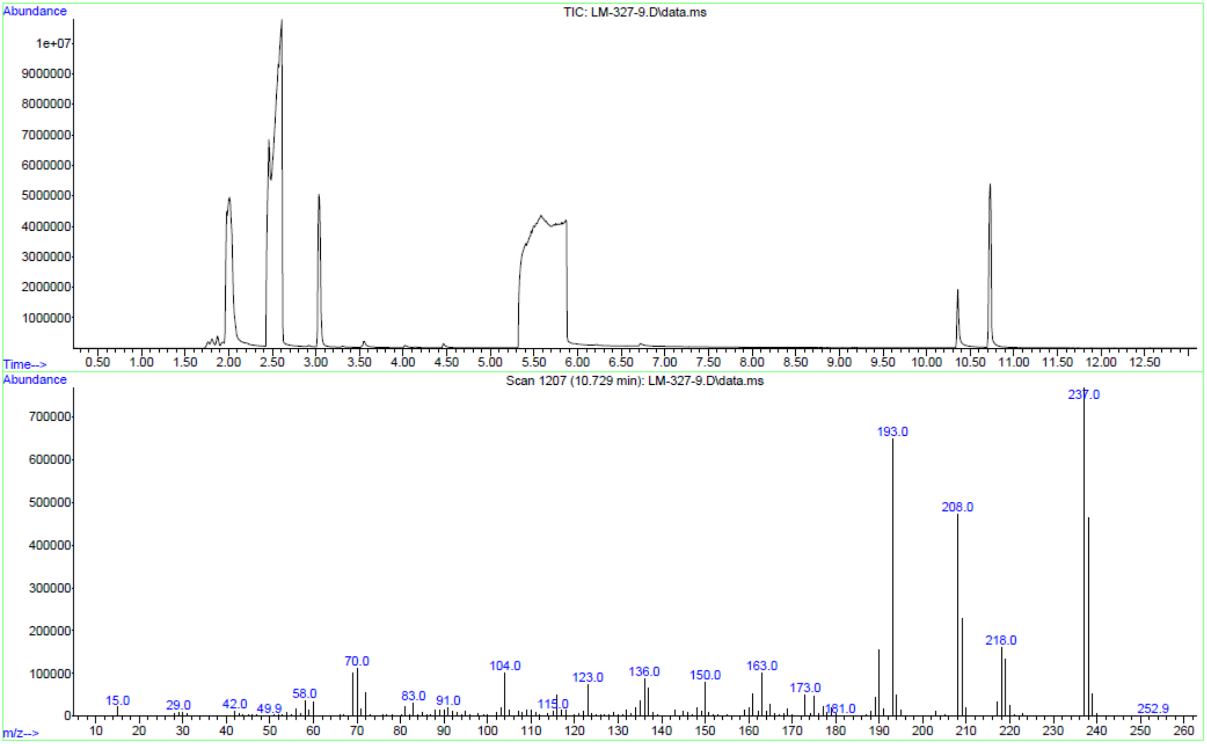

Abundance
TIC: LM-327-9.D\data.ms
Time-->
Abundance
Scan 1207 (10.729 min): LM-327-9.D\data.ms
237.0
193.0
208.0
218.0
70.0
104.0
123.0
136.0
150.0
163.0
173.0
m/z-->

General procedure for the photocatalytic HAT reactions (Procedure C)

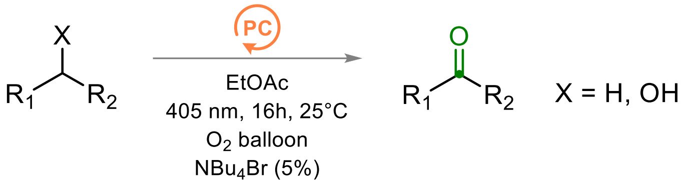

A 5 ml crimp vial, equipped with a stirring bar, was charged with the starting material (0.1 mmol, 1.0 equiv), photocatalyst **NQH$_2$-1** (0.01 mmol, 0.1 equiv), tetrabutylammonium bromide (0.005 mmol, 0.05 equiv) and 1 ml of EtOAc. The vial was sealed and fitted with an oxygen balloon attached to a syringe needle. The reaction mixture was irradiated with a 405 nm LED through the flat bottom side of the vial at 25 °C, unless noted otherwise. Following irradiation, the crude reaction mixture was analyzed by ^{1}H NMR. 1,1,2,2-tetrachlorethane (10 µL, 0.95 equiv) was added directly to the vial as an internal standard. A 50 µL aliquot of the reaction mixture was then combined with 0.55 ml of $CDCl_3$ in an NMR tube for analysis.

Note: The internal standard is indicated with an asterisk in the NMR spectra.

To isolate the desired product, the reaction mixtures from four vials were combined and transferred to a separatory funnel containing 30 ml of water and 10 ml of brine. The mixture was extracted with ethyl acetate (3 × 15 ml). The combined organic phase was dried over $MgSO_4$ and concentrated under reduced pressure. The crude material was purified by flash column chromatography using petrol ether/ethyl acetate or other suitable solvent mixtures (PE/DCM, DCM/MeOH, EtOAc/MeOH, PE/acetone wherever applicable) as eluents on silica gel.

General procedure for the photocatalytic dehalogenative coupling reactions (Procedure D)

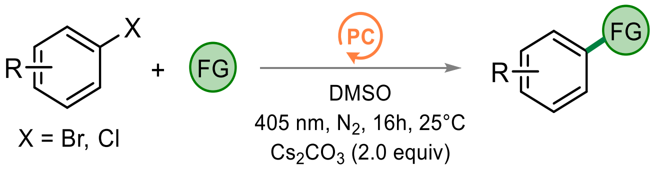

A 5 ml crimp vial equipped with a stirring bar was charged with the (hetero)aryl halide (0.1 mmol, 1.0 equiv), Cs_2CO_3 (65 mg, 2.0 equiv), and photocatalyst **NQH$_2$-1** (2.6 mg, 0.1 equiv). The vial was sealed, evacuated, and backfilled with nitrogen three times. Dry DMSO (1 ml), degassed by the freeze-pump-thaw method, was added via syringe. *N*-Methylpyrrole, purified by filtration over basic aluminum oxide until colorless, was then introduced into the reaction vessel via syringe (89 µL, 10 equiv). The reaction mixture was irradiated with a 405 nm LED through the flat bottom side of the vial at 25 °C for approximately 16 hours. Following irradiation, the crude reaction mixture was analyzed by ^{19}F NMR. Fluorobenzene (10 µl, 1.0 equiv) was added directly to the vial as an internal standard. A 50 µl aliquot of the reaction mixture was then combined with 0.55 ml of $CDCl_3$ in an NMR tube for analysis.

Note: It is to be noted here that due to the high concentration of DMSO in the crude NMR sample, the ^{19}F NMR peaks were slightly shifted from their literature values. The internal standard is indicated with an asterisk in the NMR spectra.

To isolate the desired product, the reaction mixtures from four vials were combined and transferred to a separatory funnel containing 30 ml of water and 10 ml of brine. The reaction mixture was extracted with diethyl ether (3 × 15 ml), and the combined organic phase was dried over $MgSO_4$ and concentrated under reduced pressure. The crude material was purified by flash column chromatography using petrol ether/ethyl acetate or other suitable solvent mixtures (PE/DCM, DCM/MeOH, EtOAc/MeOH, PE/acetone wherever applicable) as eluents on silica gel.

General procedure for the photocatalytic C-H activation (Procedure E)

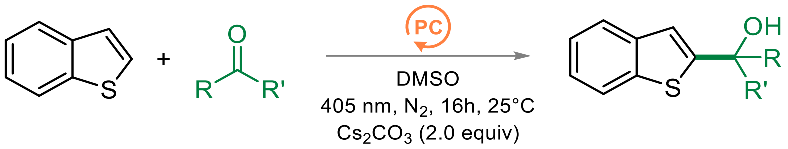

A 5 ml crimp vial, equipped with a stirring bar, was charged with benzothiophene (0.1 mmol, 13.4 mg), Cs_2CO_3 (65 mg, 2.0 equiv), and photocatalyst **NQH_2-1** (2.6 mg, 0.1 equiv). The vial was sealed, evacuated, and backfilled with nitrogen three times. Dry DMSO (1 ml), degassed by three freeze-pump-thaw cycles, and acetone (148 µl, 20 equiv) were then added via syringe. The reaction mixture was irradiated with a 405 nm LED through the flat bottom side of the vial at 25 °C for approximately 16 hours. The crude reaction mixture was analyzed directly by GC or GC-MS using 4-methylanisole (10 µl, 0.79 equiv) as an internal standard.

To isolate the desired product, the reaction mixtures from four vials were combined and transferred to a separatory funnel containing 30 ml of water and 10 ml of brine. The mixture was extracted with diethyl ether (3 × 15 ml). The combined organic phases were dried over $MgSO_4$ and concentrated under reduced pressure. The crude material was purified by flash column chromatography using petrol ether/ethyl acetate or other suitable solvent mixtures (PE/DCM, DCM/MeOH, EtOAc/MeOH, wherever applicable) as eluents on silica gel.

5. Reaction monitoring and optimization

5.1 Oxidative transformation

Control reactions and optimization reactions were carried out according to general procedure B using 1,3,5-trimethoxy-pyrimidine (Table S1). The yield of the reaction was determined by crude ^{19}F NMR with fluorobenzene as internal standard.

Table S1. Control reactions and optimization reactions for the photooxidative trifluormethylation of 1,3,5-trimethoxypyrimidine.

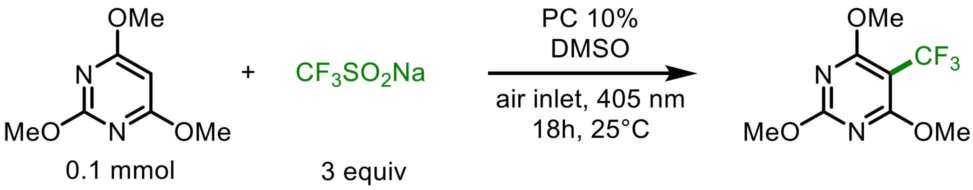

Entry	Photocatalyst	Light [nm]	Atmosphere	Yield
1	**NQH_2-1**	405	air inlet	83%
Different photocatalysts				
2	**NQH_2-2**	405	air inlet	70%
3	**NQH_2-3**	405	air inlet	69%
4	**NQH_2-4**	405	air inlet	61%
5	**NQ-1**	405	air inlet	89%
6	**NQ-2**	405	air inlet	16%
7	**NQ-3**	405	air inlet	28%
8	**NQ-4**	405	air inlet	76%
9	Lawsonia inermis powder extract	405	air inlet	2%
Control reactions				
10	**NQH_2-1**	405	O_2 balloon	40%
11	-	405	air inlet	18%
12	**NQH_2-1**	405	N_2	5%

Control reactions and optimization reactions were carried out according to general procedure B using 1,3,5-trimethoxybenzene (Table S2). The yield of the reaction was determined by calibrated GC-FID measurements with 4-methyl-anisole as internal standard. The yields in parentheses indicate the yield of di-trifluormethylated trimethoxybenzene.

Table S2. Control reactions and optimization reactions for the photooxidative trifluormethylation of 1,3,5-trimethoxybenzene. The yield of double trifluormethylated trimethoxybenzene is shown in parentheses.

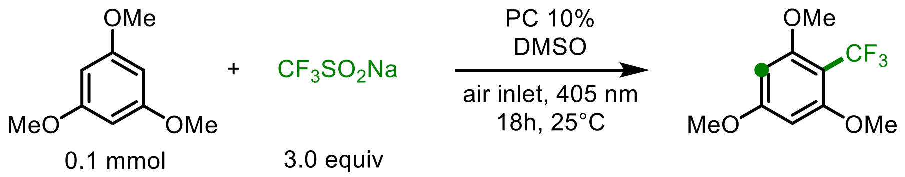

Entry	Photocatalyst	Light [nm]	Atmosphere	CF_3SO_2Na [equiv]	Yield
1	**NQH_2-1**	405	O_2 balloon	3	34%
2	**NQH_2-1**	405	air inlet	3	74% (26%)
3	**NQH_2-1**	450	air inlet	3	70% (1%)
4	**NQH_2-1**	405	air inlet	1.2	75% (27%)
Different photocatalysts					
5	**NQH_2-2**	405	air inlet	3	68% (12%)
6	**NQH_2-3**	405	air inlet	3	48% (21%)
7	**NQH_2-4**	405	air inlet	3	79% (9%)
8	**NQ-1**	405	air inlet	3	85% (5%)
9	**NQ-2**	405	air inlet	3	81% (10%)
10	**NQ-3**	405	air inlet	3	34%
11	**NQ-4**	405	air inlet	3	77% (2%)
12	**2-SAS**	450	O_2 balloon	3	95%
Control reactions					
13	-	405	air inlet	3	11%
14	**NQH_2-1**	dark	air inlet	3	n.d.
15	-	450	O_2 balloon	3	16%

Control reactions and optimization reactions were carried out according to general procedure B using 1,3,5-trimethoxybenzene (Table S3). The yield of the reaction was determined by calibrated GC-FID measurements with 4-methyl-anisole as internal standard or crude ^{1}H-NMR with 1,1,2,2-tetrachlorethane as internal standard or ^{31}P-NMR with triphenylphosphine oxide as internal standard.

Table S3: Control reactions and optimization reactions for the photooxidative coupling of 1,3,5-trimethoxybenzene with different nucleophiles.

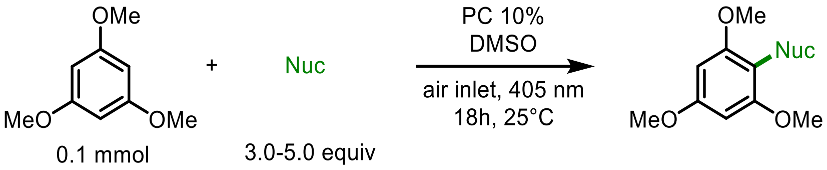

Entry	Photocatalyst	Wavelength [nm]	Nucleophile [equiv]	Additive	Time [h]	Yield
1	**NQH$_2$-1**	405	KBr [5]	-	45	35%
2	-	405	KBr [5]	-	45	n.d.
3	**2-SAS**	450	KBr [5]	-	167	>95%
4	-	450	KBr [5]	-	167	n.d.
5	**NQH$_2$-1**	405	KBr [5]	TFA (20%)	45	38%
6	**NQH$_2$-1**	dark	KBr [5]	TFA (20%)	45	4%
NaSCN as nucleophile						
7	**NQH$_2$-1**	405	NaSCN [5]	-	18	>95%
8	-	405	NaSCN [5]	-	18	>95%
9	**2-SAS**	450	NaSCN [5]	-	18	>95%
10	-	450	NaSCN [5]	-	18	11%
KCN as nucleophile						
11	**NQH$_2$-1**	405	KCN [3]	-	68	1%
12	**NQH$_2$-1**	405	KCN [3]	0.2 ml H_2O	93	4%
13	**2-SAS**	450	KCN [3]	-	93	51%
14	**2-SAS**	450	KCN [3]	0.2 ml H_2O	93	66%
15	-	450	KCN [3]	0.2 ml H_2O	93	n.d.
$P(OEt)_3$ as nucleophile						
16	**NQH$_2$-1**	405	$P(OEt)_3$ [5]	-	18	traces
17	**NQH$_2$-1**	405	$P(OEt)_3$ [5]	$(NH_4)_2S_2O_8$ (2.2 equiv)	18	traces
18[a]	**NQH$_2$-1**	405	$P(OEt)_3$ [5]	-	18	traces
19[a,b]	**NQH$_2$-1**	405	$P(OEt)_3$ [5]	$(NH_4)_2S_2O_8$ (2.2 equiv)	18	5%
20[a,b]	-	405	$P(OEt)_3$ [5]	$(NH_4)_2S_2O_8$ (2.2 equiv)	18	n.d.
21[a,b]	**2-SAS**	450	$P(OEt)_3$ [5]	$(NH_4)_2S_2O_8$ (2.2 equiv)	18	64%
22[a,b]	-	450	$P(OEt)_3$ [5]	$(NH_4)_2S_2O_8$ (2.2 equiv)	18	n.d.
Pyrazole as nucleophile						
23	**NQH$_2$-1**	405	Pyrazole [5]	-	45	35%
24	-	405	Pyrazole [5]	-	45	n.d.
25	**2-SAS**	450	Pyrazole [5]	-	45	22%
26	-	450	Pyrazole [5]	-	45	n.d.
Imidazole as nucleophile						
27	**NQH$_2$-1**	405	Imidazole [5]	-	45	16
28	-	405	Imidazole [5]	-	45	n.d
29	**2-SAS**	450	Imidazole [5]	-	45	83%
30	-	450	Imidazole [5]	-	45	n.d.

[a] MeCN instead or DMSO used as solvent; [b] nitrogen atmosphere instead of oxygen balloon

5.2 HAT-based oxidative transformations

Control reactions and optimization reactions were carried out according to general procedure C using 1-phenylethanol (0.3 mmol, 1.0 equiv) (Table S4). The yield of the reaction was determined by crude ^{1}H-NMR with 1,1,2,2-tetrachlorethane as internal standard.

Table S4. Control reactions and optimization reactions for the HAT-based photooxidation of 1-phenylethanol.

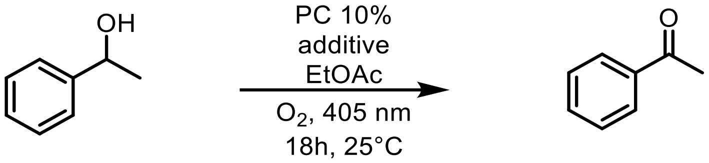

Entry	Photocatalyst	Light [nm]	Atmosphere	Solvent	Additive	Yield
1	**NQH_2-1**	405	air sealed	EtOAc	-	15%
2	**NQH_2-1**	405	air inlet	EtOAc	-	17%
3	**NQH_2-1**	450	air inlet	EtOAc	-	27%
Solvent screening						
4	**NQH_2-1**	450	air inlet	MeCN	-	n.d.
5	**NQH_2-1**	450	air inlet	MeOH	-	3%
6	**NQH_2-1**	450	air inlet	acetone	-	16%
7	**NQH_2-1**	450	air inlet	DMSO	-	18%
8	**NQH_2-1**	450	air inlet	DCM	-	12%
9	**NQH_2-1**	450	air inlet	MeCN/H_2O 1:1	-	13%
10	**NQ-1**	450	air inlet	EtOAc	-	32%
11	**NQH_2-1**	450	O_2 balloon	EtOAc	-	37%
NBr_4Br as additive						
12	**NQH_2-1**	450	O_2 balloon	EtOAc	NBu_4Br 5%	81%
13	**NQ-1**	450	O_2 balloon	EtOAc	NBu_4Br 5%	>95%
14	**NQH_2-1**	405	O_2 balloon	EtOAc	NBu_4Br 5%	>95%
Control reactions						
15	**NQH_2-1**	405	N_2	EtOAc	NBu_4Br 5%	3%
16	**NQ-1**	450	N_2	EtOAc	NBu_4Br 5%	18%
17	**NQH_2-1**	-	O_2 balloon	EtOAc	NBu_4Br 5%	1%
18	-	405	O_2 balloon	EtOAc	NBu_4Br 5%	5%

5.3 Reductive transformations

Control reactions and optimization reactions were carried out according to general procedure D using 1-chloro-2-(trifluoromethyl)benzene (0.1 mmol, 1.0 equiv) and *N*-methylpyrrole (1.0 mmol, 10 equiv) (Table S5). The yield of the reaction was determined by crude ^{19}F NMR with fluorobenzene as internal standard.

Table S5. Control reactions and optimization reactions for the photoreductive dehalogenative coupling of 2-chlorobenzotrifluoride and *N*-methylpyrrole.

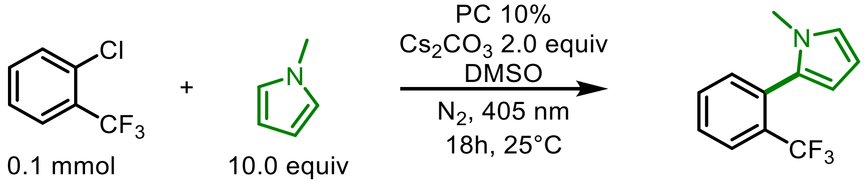

Entry	Photocatalyst	Light [nm]	Additive [equiv]	Yield
1	**NQH$_2$-1**	405	Cs_2CO_3 [2]	56%
2	**NQH$_2$-1**	450	Cs_2CO_3 [2]	43%
Base screening				
3	**NQH$_2$-1**	405	Cs_2CO_3 [1]	31%
4	**NQH$_2$-1**	405	TMG [2]	42%
Different photocatalysts				
5	**NQ-1**	405	Cs_2CO_3 [2]	26%
6	**NQH$_2$-2**	405	Cs_2CO_3 [2]	37%
7	**NQH$_2$-3**	405	Cs_2CO_3 [2]	35%
8	**NQH$_2$-4**	405	Cs_2CO_3 [2]	53%
Control reactions				
9	-	405	Cs_2CO_3 [2]	9%
10	**NQH$_2$-1**	405	-	11%
11	**NQH$_2$-1** 40%	405	-	17%
12	**NQH$_2$-1**	dark	Cs_2CO_3 [2]	n.d.

Control reactions and optimization reactions were carried out according to general procedure E using benzothiophene (0.1 mmol, 1.0 equiv) and acetone (1.0 mmol, 10 equiv). The yield of the reaction was determined by calibrated GC-FID with 4-methyl-anisole as internal standard.

Table S6. Control reactions and optimization reactions for the photoreductive coupling of benzothiophene and acetone.

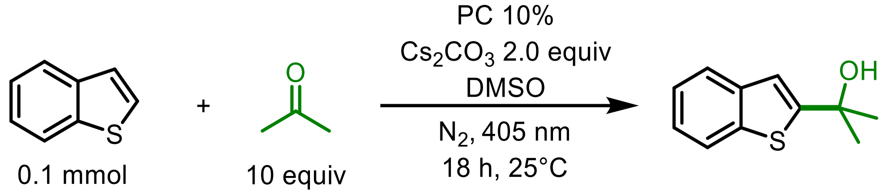

Entry	PC	solvent	additive	Yield
1	**NQH_2-1**	DMSO	-	70
Solvent screening				
2	**NQH_2-1**	THF	-	n.d.
3	**NQH_2-1**	MeOH	-	n.d.
4	**NQH_2-1**	DCM	-	n.d.
5	**NQH_2-1**	DMF	-	52
Different photocatalysts				
6	**NQH_2-4**	DMSO	-	21
7	**NQH_2-1+NQ-1** (1:1)	DMSO	-	10
8	**NQ-1** (20%)	DMSO	-	43
Different additives				
9	**NQH_2-1**	DMSO	$NaHSO_3$ (0.5 equiv)	80
10	**NQH_2-1**	DMSO	H_2O (5.0 equiv)	56
11	**NQH_2-1**	DMSO	O_2 2 cm^3	n.d.
12	**NQ-1**	DMSO	$NaHSO_3$ (0.5 equiv)	49
13	**-**	DMSO	$NaHSO_3$ (1.0 equiv)	9
14	**NQH_2-1**	DMSO	TMG instead of Cs_2CO_3	19
Control reactions				
15	**-**	DMSO	-	n.d.
16	**NQH_2-1**	DMSO	no Cs_2CO_3	n.d.

6. Mechanistic discussion

Quinones, due to their inherent redox properties, are capable of facilitating a wide range of chemical transformations. Similar to other photocatalytic processes, particularly those involving organic dye molecules under photoirradiation, the complete mechanistic picture of these photochemical reactions encompassing oxidative, reductive, and hydrogen atom transfer (HAT) processes, in conjunction with radical and nucleophilic ionic species remains elusive. Moreover, light-absorbing materials tend to decompose under photoirradiation. In the following, we present our initial findings on the mechanistic aspects of these reactions.

6.1 Oxidative direction

Photochemical reactions necessitate both the presence of a photocatalyst and light irradiation (cf. Tables S1-S2), underscoring the critical role these factors play in the process. Notably, while the photochemical reaction begins with the identified photocatalyst, light irradiation of the reaction mixture leads to the formation of multiple photocatalytic species, part of which in combination appear capable of driving the photochemical reaction (see also photostability measurements, section S7.7). Our initial investigation indicates that the original photocatalyst undergoes a transformation into other species, yet the photochemical reaction persists. The most obvious explanation would be the transformation of the naphthohydroquinone derivate to the naphthoquinone derivate under oxidative conditions. This however does not match the absorbance of the photocatalyst solution after irradiation (see Figure S19). While the naphthoquinone derivate **NQ-1** can be employed successfully as photocatalyst, photostability measurements indicate that also this quinone form undergoes transformation into multiple photocatalytic species, that seem capable of driving the reaction. Mechanistic quenching studies in the oxidative direction with the naphthoquinone derivate **NQ-1** or its photophysics could not be performed conclusively, considering the limited photostability of the quinone form and its rather weak luminescence signal.

Additional experiments, including a series of reactions and monitoring the kinetics of product formation, revealed that when the original photocatalyst was subjected to photoirradiation to induce photochemical transformation (Figure S3, left) the photocatalytic reaction kinetic was slightly slower (Figure S3, right). At present, it is unclear which specific species are actively involved, or the extent of their contribution to the photochemical reactions and the formation of the desired product. We are continuing to investigate these mechanisms in detail, with the aim of advancing the understanding of quinones as photocatalysts and guiding the development of new photocatalysts in this field.

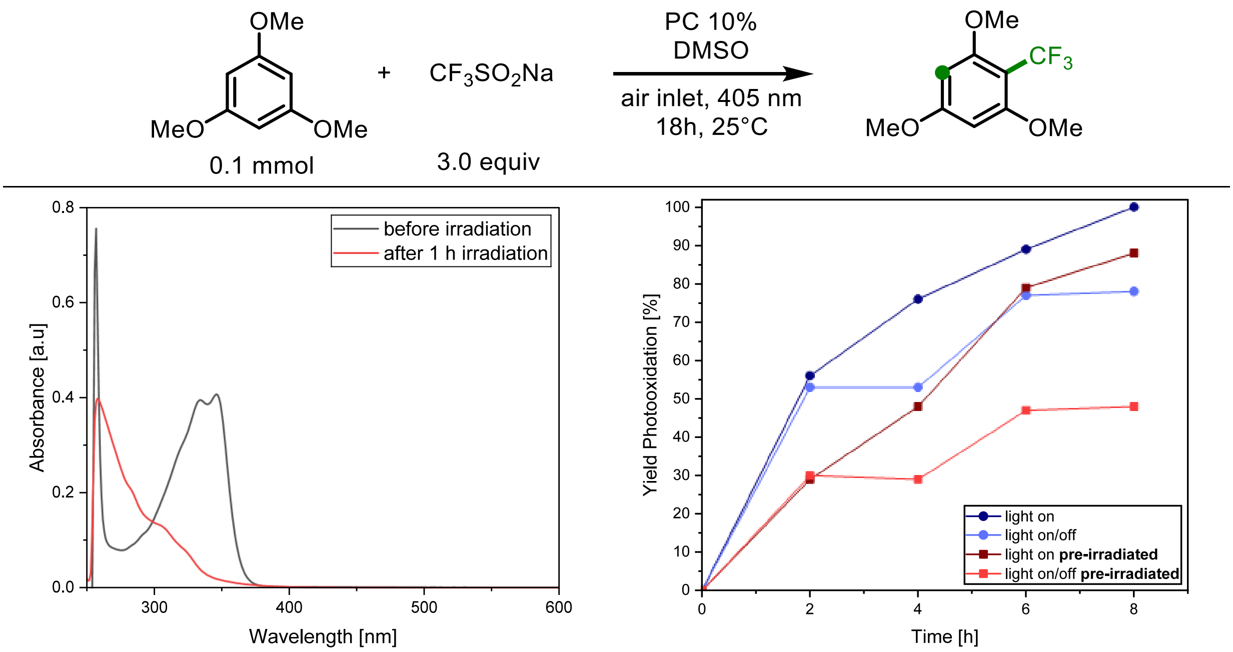

Figure S3. Left panel: UV-Vis absorbance of **NQH_2-1** in DMSO before (black) and after (red) irradiation. Right panel: Yield of the photooxidative trifluoromethylation reaction with 1,3,5-trimethoxybenzene with continuous and interrupted irradiation at 405 nm under normal conditions (blue) and with pre-irradiated photocatalyst (red).

To investigate whether the transformed photocatalyst was driving the reaction by a radical chain mechanism, a light on/off experiment was designed. First, the photocatalyst (0.01 mmol, 0.1 equiv) was subjected to 1 h of irradiation at 405 nm wavelength in DMSO. To monitor whether the transformation to other species was taking place, the mixture was investigated by UV-Vis spectroscopy (Figure S3, left). Indeed, the UV-Vis signature of the photocatalyst changed, indicating that a transformation had taken place. To this mixture, 1,3,5-trimethoxybenzene and sodium triflate were added and the reaction yield was monitored over time with one batch being irradiated continuously with 405 nm, while a second batch was subjected to interrupted (2h irradiated, 2h dark) irradiation conditions (Figure S3, right). Aliquot samples (50 µl) were drawn at indicated times and measured via ^{19}F NMR after addition of 0.55 ml $CDCl_3$ and 2 µl fluorobenzene as internal standard. Indeed, no rise in yield could be observed under dark conditions, so the progress of the reaction *via* radical-chain mechanism can be ruled out.
Additionally, the quantum yield of the photooxidative transformation was found to be on the order of 1% or less (see Section 6.4).

6.2 HAT direction

The potential mechanism for the HAT catalytic transformations has already been discussed in the main manuscript. The reaction is likely mediated by the in-situ generated active photocatalytic species (ACS) that is able to abstract a proton from its photoexcited state and regenerated with oxygen as terminal oxidant.

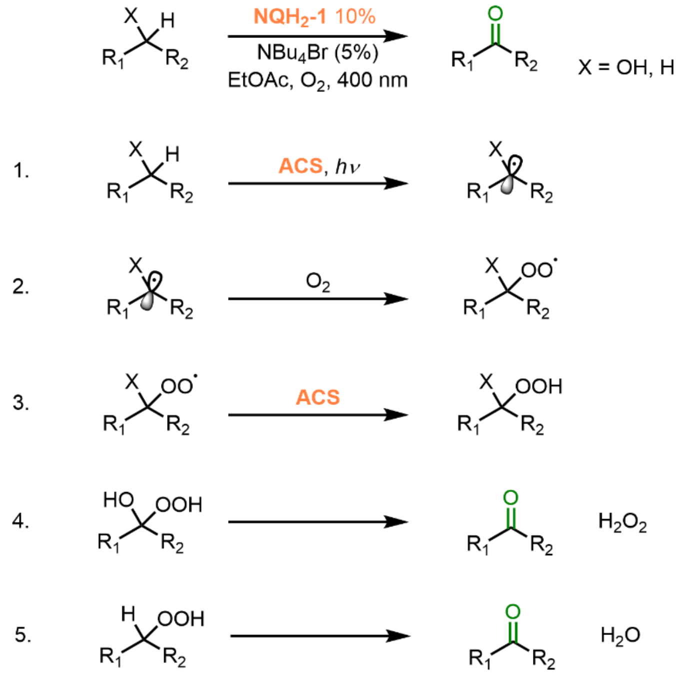

Figure S4. Possible mechanistic pathway for HAT catalytic transformations using excited state quinones under oxygen, ACS = active catalytic species.

6.3 Reductive direction

Similar to oxidative processes, photochemical reductive transformations also require the presence of both a photocatalyst and light irradiation (see Tables S4-6). The absorption and fluorescence spectra of **NQH_2-1** are presented in Figure S5. The addition of 2-Cl-benzotrifluoride to a solution containing **NQH_2-1** did not result in a significant change in the absorption spectra. However, a substantial change in fluorescence intensity and lifetime was observed, indicating a potential electron transfer from the excited state of **NQH_2-1** to the substrate (Figure S6). This fluorescence change did not occur when 4-Cl-anisole, *N*-methylpyrrole, acetone, or benzothiophene were added to the solution, suggesting that the deprotonated photocatalyst, formed in the presence of Cs_2CO_3, or a transformed photocatalyst, plays a critical role in facilitating the single-electron transfer step. Additionally, **NQH_2-1** underwent photochemical transformation upon photoirradiation under a nitrogen atmosphere, and the transformed photocatalyst or photocatalysts in combination may play a crucial role in the single electron transfer step yielding the desired product (see section S7.7). Capturing the luminescence of the deprotonated photocatalyst proved challenging due to the dynam-

ic equilibrium between the protonated and deprotonated species. However, the light on-off experiments confirmed that illumination is essential for the photochemical transformation, whether it involves single-electron transfer, depending on the substrate, from the excited state of **NQH$_2$-1** or of the deprotonated catalyst or of a photo-transformed catalyst.

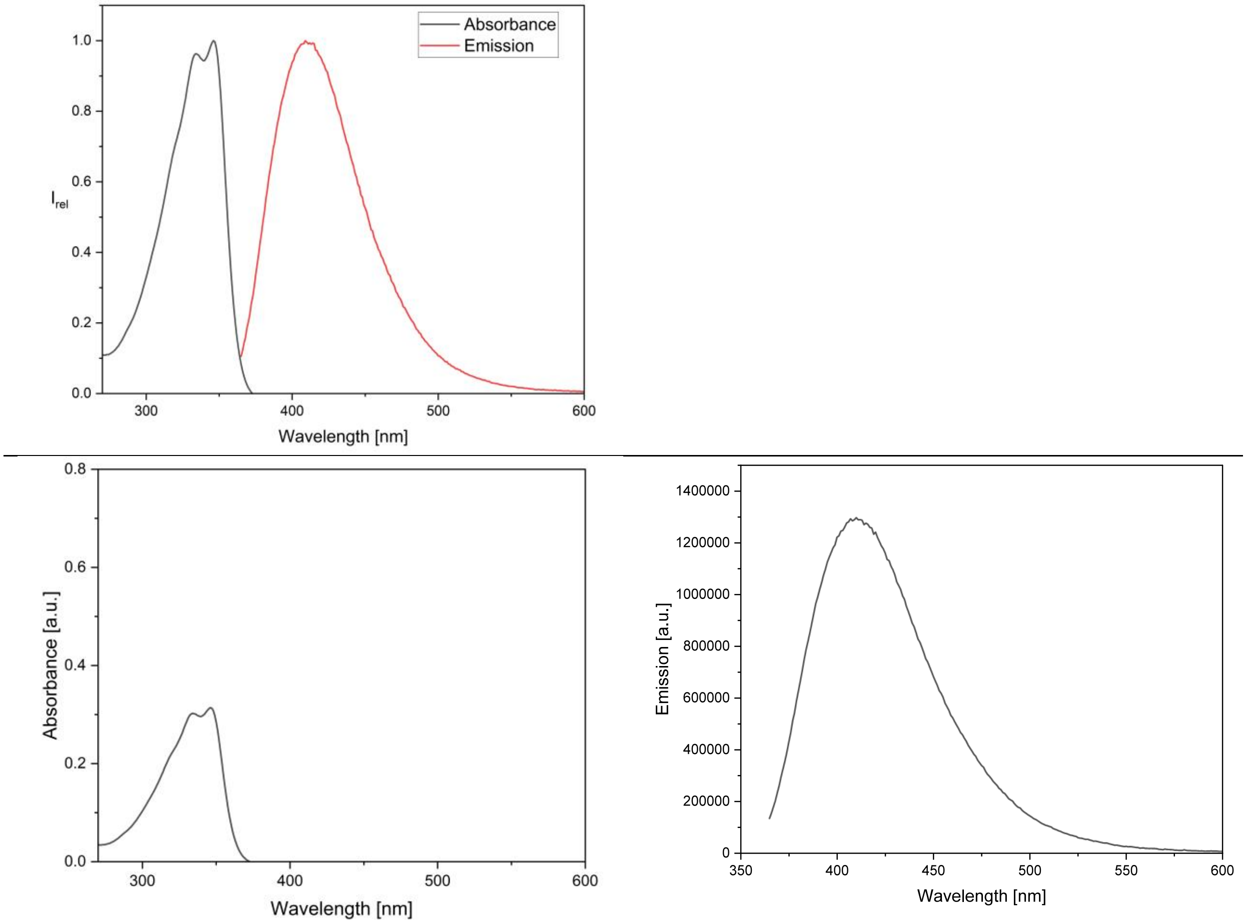

Figure S5. Absorption and fluorescence spectra (λ_{exc} = 350 nm) of **NQH$_2$-1** in DMSO

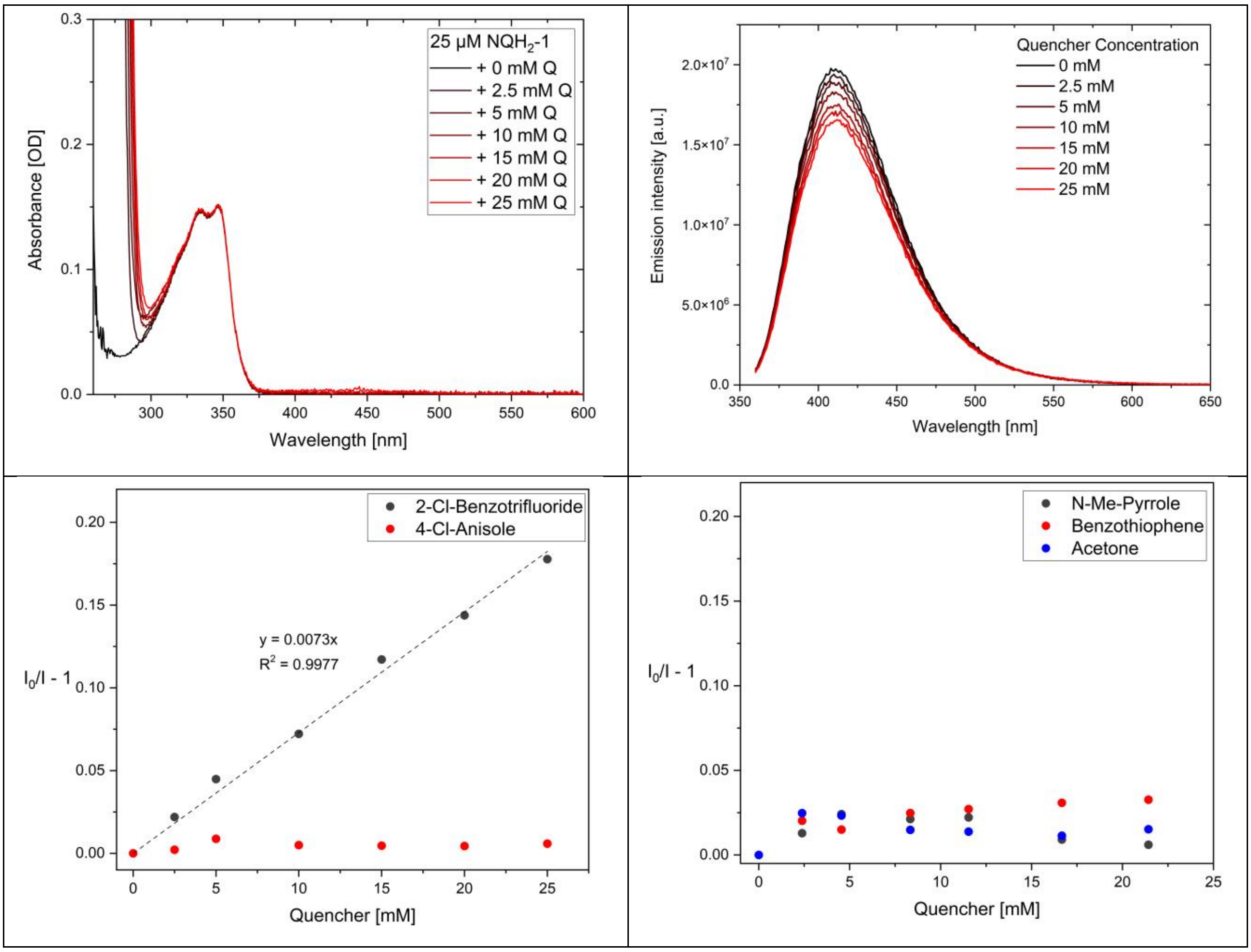

Figure S6. Stern-Volmer fluorescence quenching studies with **NQH$_2$-1** in DMSO (25 µM) without added base. The lack of changes in UV-Vis absorbance (top left) indicates that the decrease in fluorescence intensity (top right) upon addition of 2-chlorobenzotrifluoride is due to fluorescence quenching and not caused by a change in concentration. Fluorescence quenching was only observed with 2-chlorobenzotrifluoride and not with other quenchers (bottom).

To investigate whether the transformed photocatalyst was eventually driving the photoreductive reactions by a radical chain mechanism, a light on/off experiment similar to Figure S3 was conducted. First, the photocatalyst (0.01 mmol, 0.1 equiv) was subjected to 1 hour of irradiation at 405 nm wavelength in DMSO in the presence of Cs_2CO_3 (0.2 mmol, 2.0 equiv). To monitor whether the transformation to other species was taking place, the mixture was investigated by UV-Vis spectroscopy (Figure S7, see also photostability measurements section S7.7). The UV-Vis signature of the photocatalyst changed as well, indicating that a transformation had taken place. Notably, the UV-Vis signature before irradiation is that of the protonated photocatalyst. This is due to the dilution effect under spectroscopic concentration, as a clear colour change can be observed upon mixing photocatalyst with base under reaction concentration (Figure S7 bottom). To this irradiated mixture 2-chlorobenzotrifluoride (0.1 mmol, 1.0 equiv) and *N*-methylpyrrole (1.0 mmol, 10 equiv) were added via syringe and the reaction yield was monitored over time with one batch being irradiated continuously with 405 nm, while a second batch was subjected to interrupted irradiation conditions (Figure S7). Aliquot samples (50 µl) were drawn at indicated times and measured via ^{19}F NMR after addition of 0.55 ml $CDCl_3$ and 2 µl fluorobenzene as internal standard. Indeed, no rise in yield could be observed under dark conditions, so the progress of the reaction *via* radical-chain mechanism can be ruled out. Additionally, the quantum yield of the photooxidative transformation was found to be on the order of 1% or less (see Section 6.4).

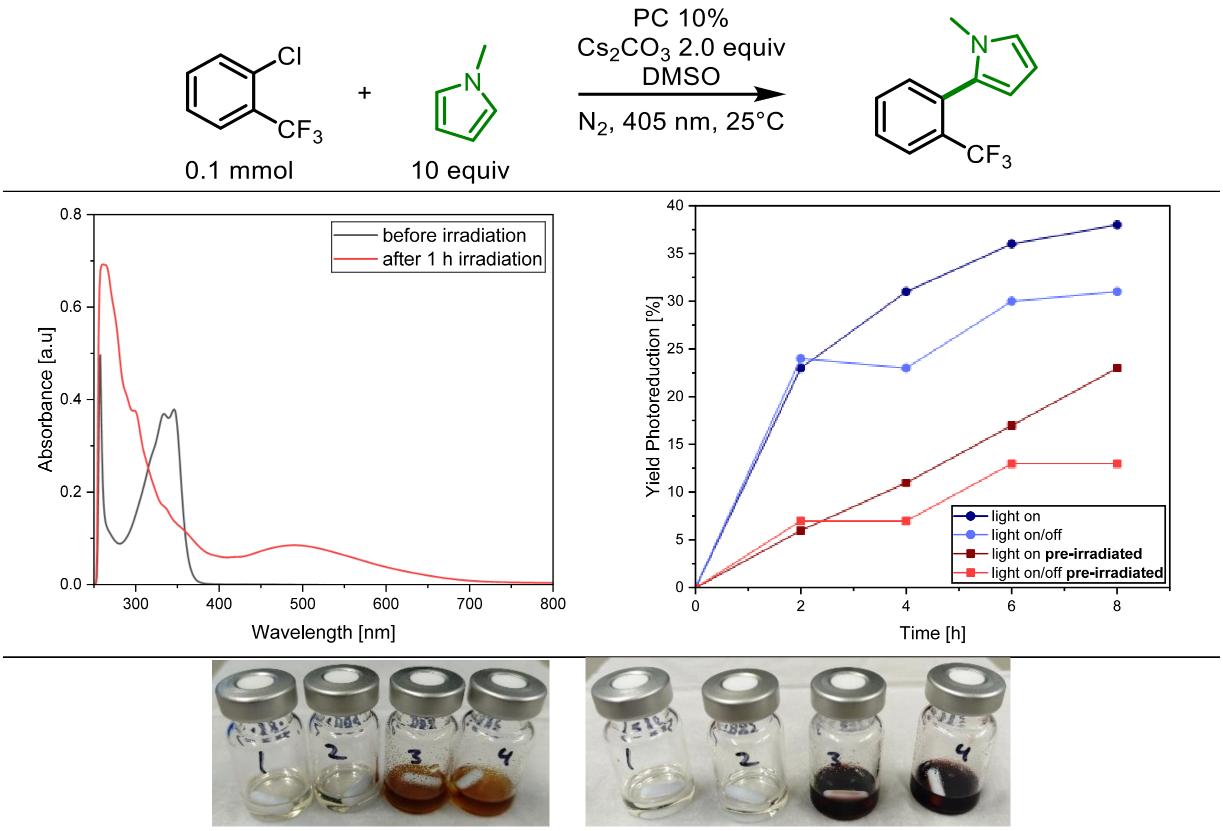

Figure S7. UV-Vis absorbance of the **NQH$_2$-1** in DMSO before (black) and after (red) irradiation (middle left). Yield of the photooxidative trifluoromethylation reaction with 1,3,5-trimethoxybenzene with continuous and interrupted irradiation at 405 nm (middle right) under normal reaction conditions (blue) and with pre-irradiated photocatalyst (red). Visible change in color upon mixing photocatalyst with base (batches 3,4, bottom left) compared to only photocatalyst in DMSO (batches 1+2, bottom left), and difference in color after 1 hour of 405 nm irradiation (bottom right).

6.4 Quantum yield measurement

The quantum yield was measured with a slightly modified **Q**uantum **Y**ield **D**etermination **S**etup (**QYDS**).[8] The samples were illuminated with a 400 nm LED (Luxeon LHUV-0400-0450, 1 A, 248 mW optical power after reference cuvette). The remaining power after the sample cuvette was measured every 5 minutes with a PowerMax-USB PM10 power meter. The difference in power between the pure solvent reference cuvette and the reading of the power meter is equivalent to the optical power absorbed by the sample. The samples for the oxidative and reductive pathway (conditions from S3 and S7) were used in their original synthetic concentrations in a volume of 2.2 mL, placed in a 10 × 10 mm cuvette with continuous stirring. For the reductive pathway, TMG (2.0 equiv) instead of Cs_2CO_3 was used to eliminate scattering of light during the measurements. For the oxidative pathway, aliquots of 100 µL were taken from the solution after 30 min, 1 h, 2 h and 3 h (2 h, 4 h, 6h and 8 h respectively for the reductive pathway) and the product yield was quantified by ^{19}F NMR. The further analysis was done according to the procedure outlined in Reference [8] (Table S7-8).

Table S7. Quantum yield determination of the oxidative pathway

**interval** *i*	**time** *t* **[h]**	**product concentration** *c* **[mM]**	**average absorbed power** P_{abs} **[mW]**	**illuminated volume** *V* **[mL]**	**quantum yield [%]**
0	0	0	0	2.2	--
1	0.5	0.7	173	2.2	0.15
2	1	2.0	168	2.1	0.25
3	2	3.1	163	2.0	0.11
4	3	4.7	167	1.9	0.15
**Averaged Quantum Yield Oxidative Pathway** $\frac{\sum_{i=1}^{4}(c_i - c_{i-1})V_i \mathrm{N_A}}{\sum_{i=1}^{4} P_{\mathrm{abs},i}(t_i - t_{i-1})/E_{\mathrm{photon}}}$ = 0.15%					

Table S8. Quantum yield determination of the reductive pathway

**interval** *i*	**time** *t* **[h]**	**product concentration** *c* **[mM]**	**average absorbed power** P_{abs} **[mW]**	**illuminated volume** *V* **[mL]**	**quantum yield [%]**
0	0	0	0	2.2	--
1	2	16	247	2.2	0.58
2	4	18	247	2.1	0.09
3	6	20	247	2.0	0.05
4	8	21	247	1.9	0.05
**Averaged Quantum Yield Reductive Pathway** $\frac{\sum_{i=1}^{4}(c_i - c_{i-1})V_i \mathrm{N_A}}{\sum_{i=1}^{4} P_{\mathrm{abs},i}(t_i - t_{i-1})/E_{\mathrm{photon}}}$ = 0.19%					

Note that several error sources may contribute, e.g. for the concentrations from NMR and the number of absorbed photons from the power meter measurements. Nonetheless, the measurements indicate that the quantum yields in both the reductive and oxidative pathways are on the order of 1% or less, thereby ruling out the possibility of radical chain mechanisms for the photooxidative and photoreductive transformations.

7. Additional spectroscopic investigations

7.1 UV-Vis absorption

Spectroscopic investigations were carried out with 1,1,3,3-tetramethylguanidin (TMG) instead of Cs_2CO_3 due to the diminished solubility of Cs_2CO_3 in DMSO and other organic solvents. As shown in Figure S8 top left, the absorbance of deprotonated **NQH_2-1** is almost identical if TMG or Cs_2CO_3 is used as base in DMSO. Upon addition of a base to the hydroquinone scaffolds a change in the absorption spectra was observed.

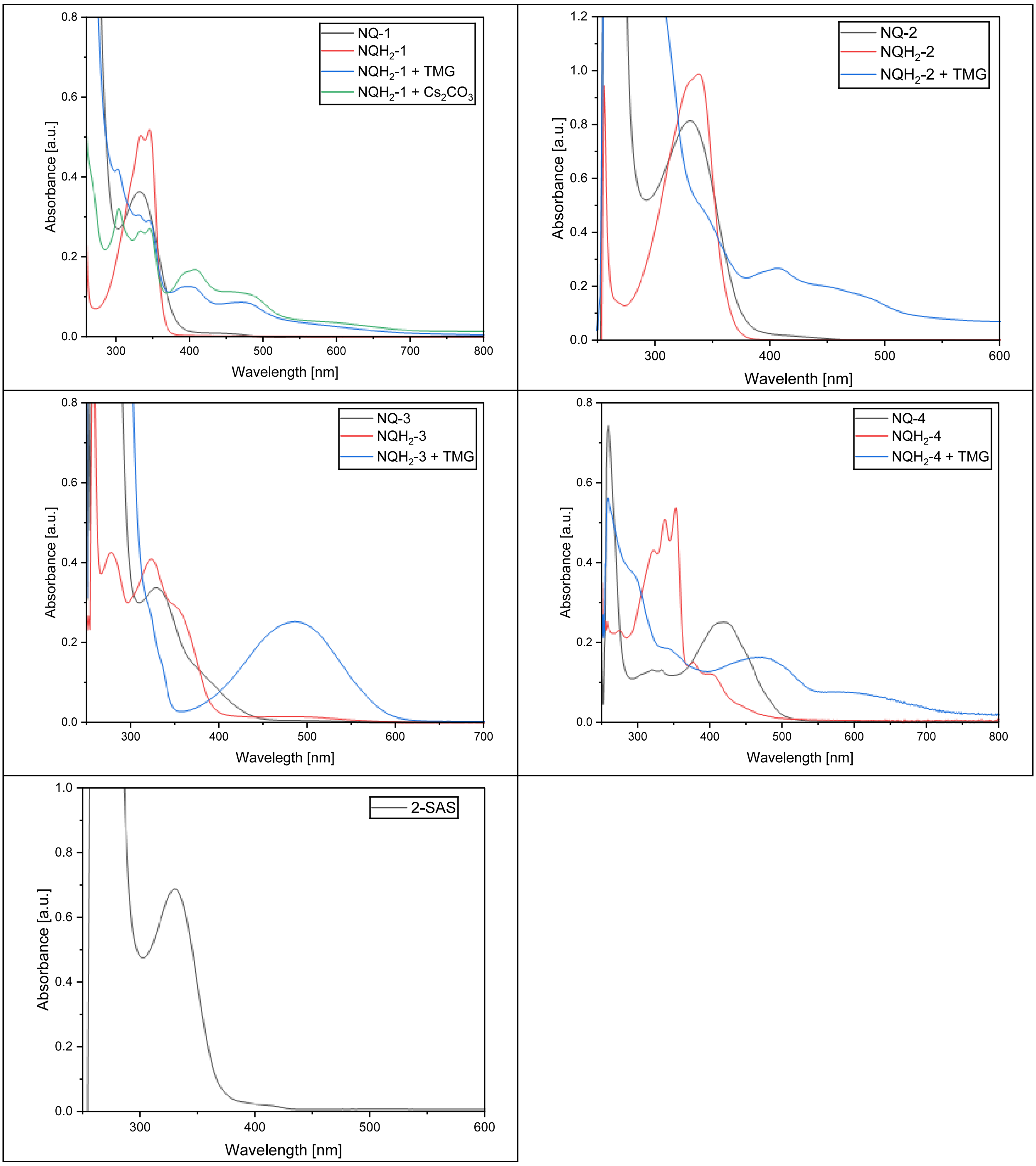

Figure S8: UV-Vis absorption spectra of the quinone photocatalysts in absence and presence of base (1,1,3,3-tetramethylguanidine (TMG)) in dried, degassed DMSO.

7.2 Emission spectra

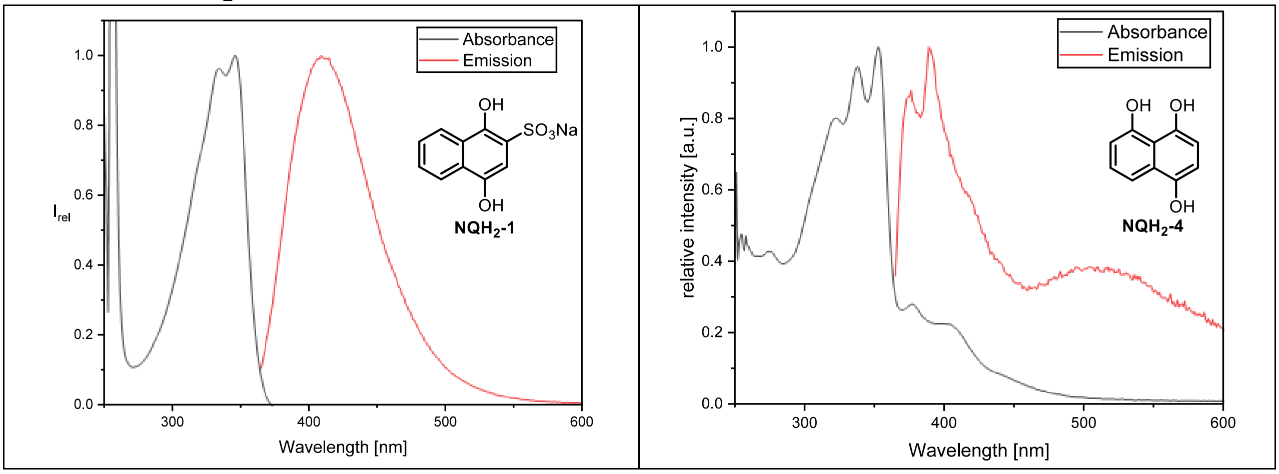

Figure S9. UV-Vis absorbance (black) and fluorescence emission (red) spectra (λ_{exc} = 350 nm) of quinone photocatalysts. The maximum intensity of both spectra was set to equal value.

7.3 Stern-Volmer Quenching

Each sample was prepared separately. The **NQH_2-1** concentration was chosen to result in approximately 0.1 OD at the absorption maximum (25 µM). All measurements were conducted in dry DMSO. Each sample was degassed in the 10 mm Starna Spectrosil® Far UV Quartz cuvette with N_2 for four minutes. For the steady-state emission spectra, a Fluorolog-3 spectrofluorometer from Horiba with the FluorEssence v3.9 software was used. The irradiation source was centered around 347 nm using a monochromator. The emission was recorded between 360 and 650 nm. The fluorescence intensities for Stern-Volmer evaluation were obtained by integration over the whole emission peak. For time-resolved emission spectra, a home-built TCSPC-setup was used.[2] The excitation source was a Horiba NanoLED-370 centered around 367 nm and the emission was recorded at 410 nm. Before and after the emission measurements, an absorption spectrum was measured. The corresponding absorption spectra were recorded using an Agilent Cary 60 spectrophotometer using the WinUV software. The wavelength range measured was set between 190 and 1100 nm.

Quenching studies with 2-Cl-benzotrifluoride

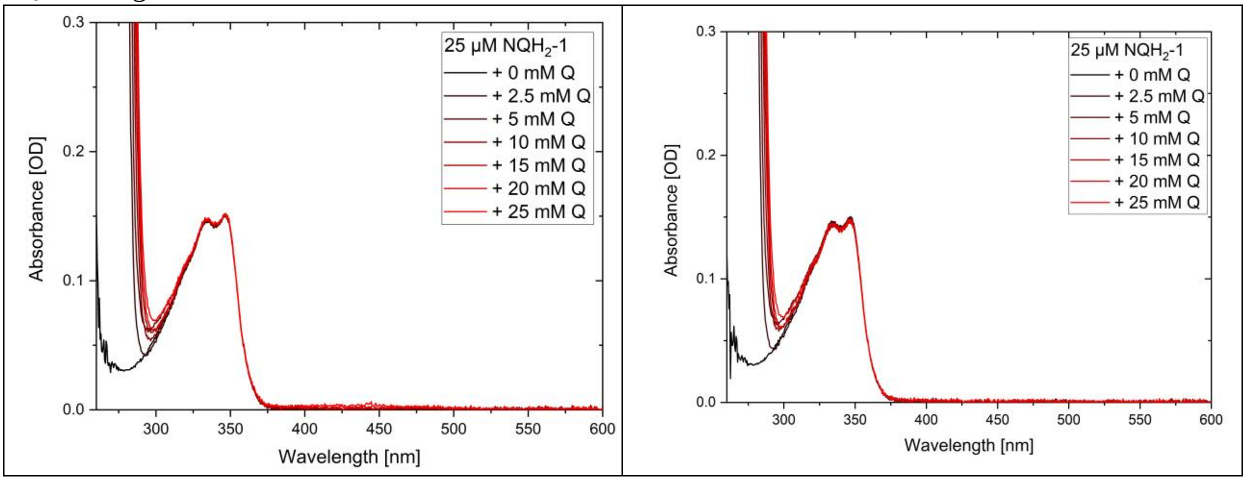

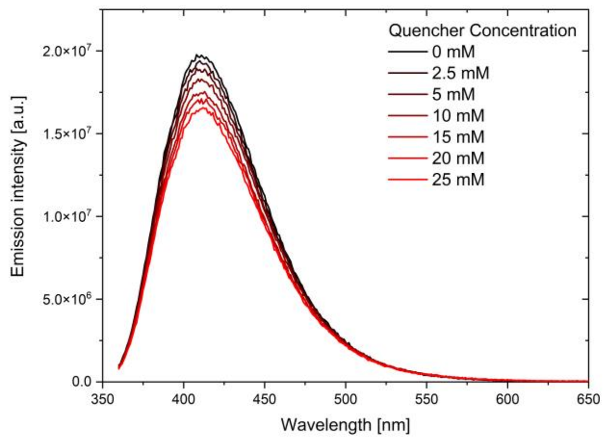

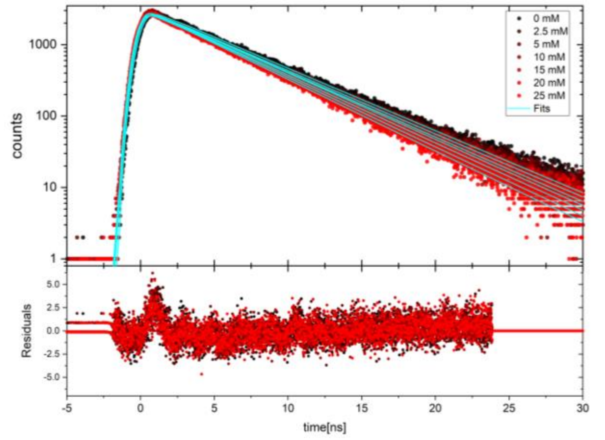

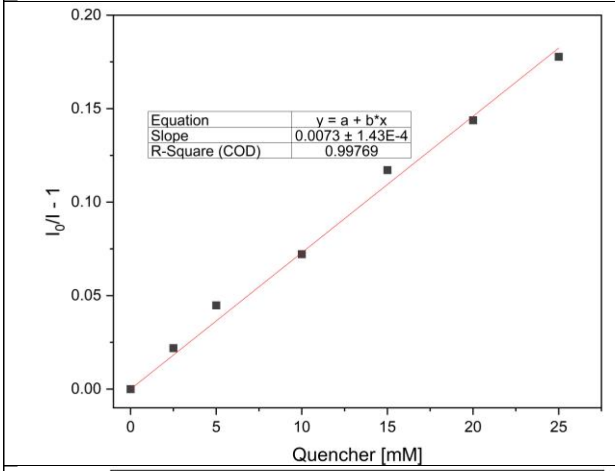

[Q] [mM]	I	I_0/I -1
0	1.58E+09	0
2.5	1.54E+09	0.022
5	1.51E+09	0.045
10	1.47E+09	0.072
15	1.41E+09	0.117
20	1.38E+09	0.144
25	1.34E+09	0.178
K_{SV} = 0.0073 ± 1.43E-4		

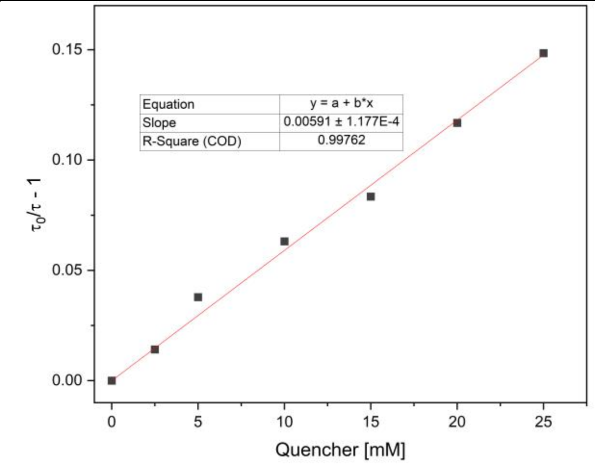

[Q] [mM]	τ	τ $_0$/ τ -1
0	4.96	0
2.5	4.89	0.014
5	4.78	0.038
10	4.67	0.063
15	4.58	0.083
20	4.44	0.117
25	4.32	0.145
K_{SV} = 0.0059 ± 1.18E-4		

Figure S10. Fluorescence quenching studies of the photocatalyst **NQH$_2$-1** with 2-Cl-benzotrifluoride. Changes in UV-Vis absorbance spectra upon quencher addition before (top left) and after the measurements (top right). Changes in the fluorescence emission spectra (top left) and fluorescence lifetime (top right) upon quencher addition, Stern-Volmer plot (bottom left) and underlying data (bottom right) of the quenching studies.

Quenching studies with 4-Cl-anisole

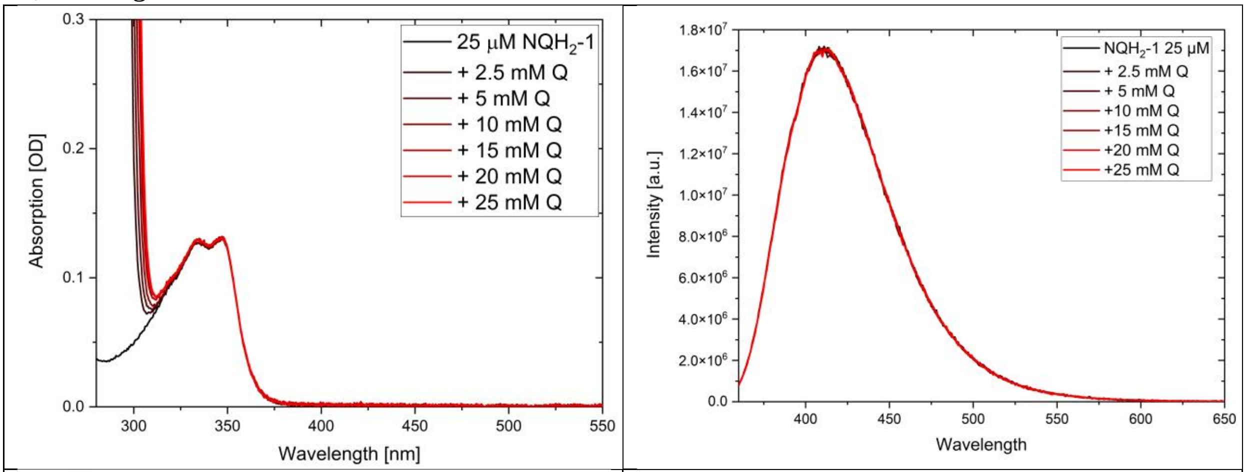

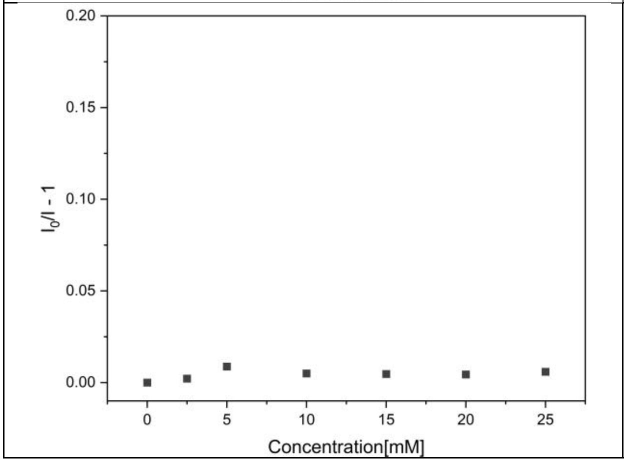

[Q] [mM]	I	I_0/I-1
0	1.370E9	0
2.5	1.367E9	2.17E-3
5	1.358E9	8.77E-3
10	1.363E9	4.99E-3
15	1.363E9	4.68E-3
20	1.363E9	4.45E-3
25	1.362E9	5.87E-3

Figure S11. Fluorescence quenching studies of the photocatalyst **NQH$_2$-1** with 4-Cl-anisole. Changes in UV-Vis absorbance spectra upon quencher addition (top left) and changes in the fluorescence emission spectra (top right). Stern-Volmer plot (bottom left) and underlying data (bottom right) of the quenching studies.

To investigate if an electron transfer from the excited photocatalyst to the coupling reagents or benzothiophene was possible, further fluorescence studies were conducted with **NQH$_2$-1**, N-methylpyrrole, benzothiophene and acetone. The samples were prepared by titrating a stock solution of quencher (50 mM) and **NQH$_2$-1** (50 µM) to a stock solution of **NQH$_2$-1** (50 µM) so that the concentration of the photocatalyst remained constant. All measurements were conducted in dry degassed DMSO in a 10 mm Hellma® quartz fluorescence cuvette. The fluorescence emission measurements were conducted on a HORIBA Fluoromax-4 spectrofluorometer with FluorEssence v3.9 software. Emission spectra were measured after being excited at 350 nm. For Stern-Volmer quenching evaluation, the emission intensity at 440 nm was selected. For each titration step, absorption, then emission, then lifetime was measured.

Quenching studies with *N*-methylpyrrole

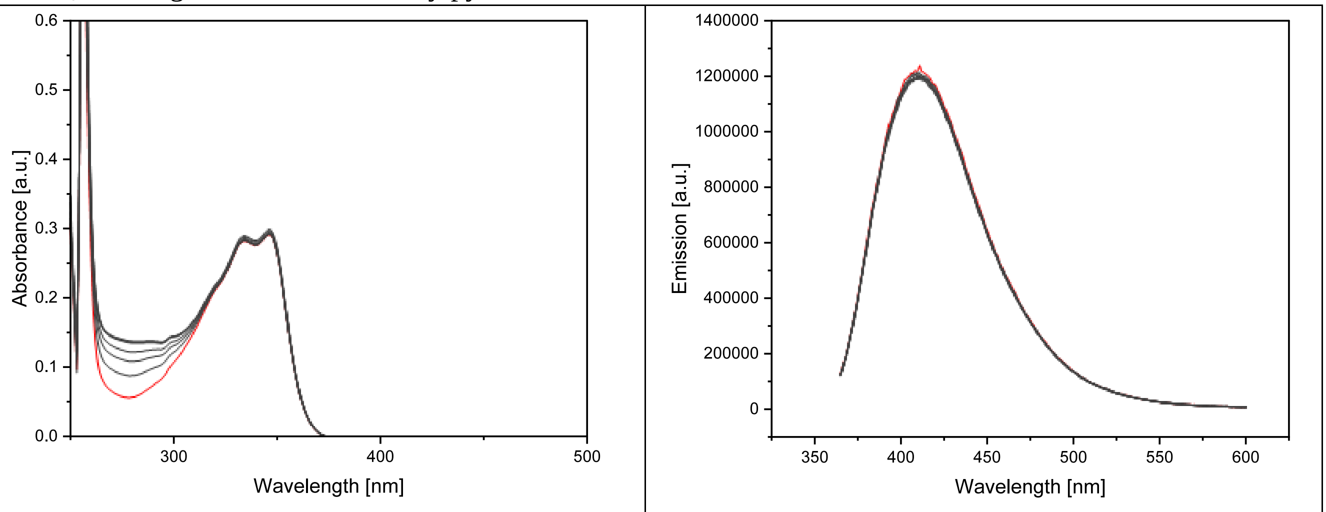

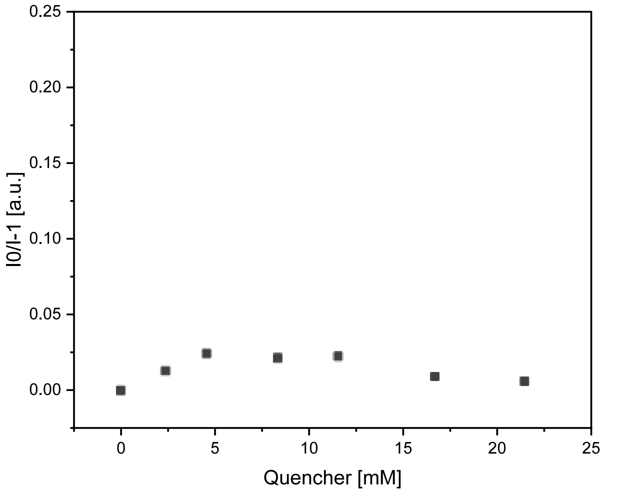

[Q] [mM]	[Q] [equiv]	I (440 nm)	I_0/I - 1
0.0	0	821949	0.000
2.4	48	811495	0.013
4.5	91	802570	0.024
8.3	167	804879	0.021
11.5	231	804089	0.022
16.7	333	814543	0.009
21.4	429	817022	0.006

Figure S12. Fluorescence quenching studies of the photocatalyst **NQH_2-1** with *N*-methylpyrrole. Changes in UV-Vis absorbance spectra upon quencher addition (top left), Changes in the fluorescence emission spectra upon quencher addition (top right), Stern-Volmer plot (bottom left) and underlying data (bottom right) of the quenching study.

Quenching studies with benzothiophene

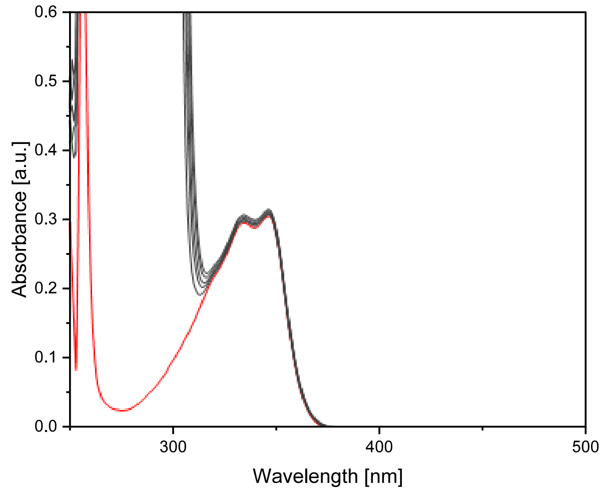

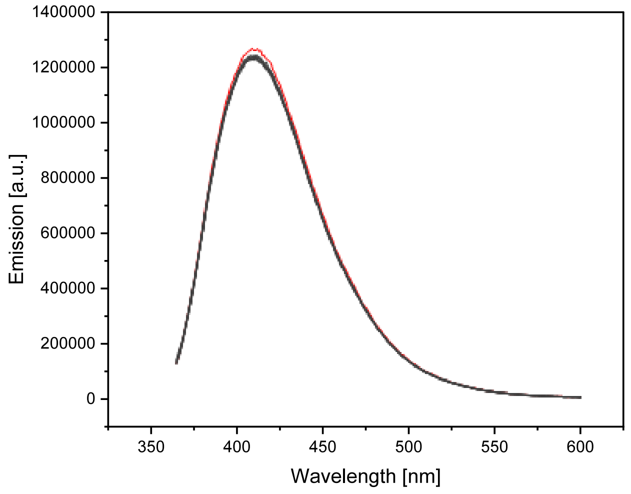

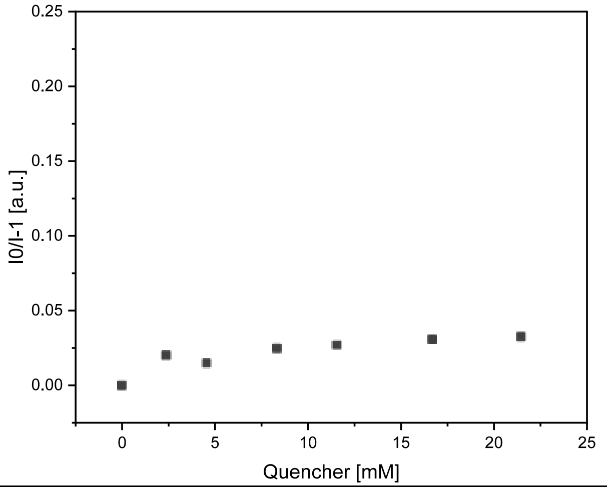

[Q] [mM]	[Q] [equiv]	I (440 nm)	I_0/I - 1
0.0	0	854070	0.000
2.4	48	837150	0.020
4.5	91	841468	0.015
8.3	167	833412	0.025
11.5	231	831513	0.027
16.7	333	828535	0.031
21.4	429	827066	0.033

Figure S13. Fluorescence quenching studies of the photocatalyst **NQH$_2$-1** with benzothiophene. Changes in UV-Vis absorbance spectra upon quencher addition (top left), Changes in the fluorescence emission spectra upon quencher addition (top right), Stern-Volmer plot (bottom left) and underlying data (bottom right) of the quenching study.

Quenching studies with acetone

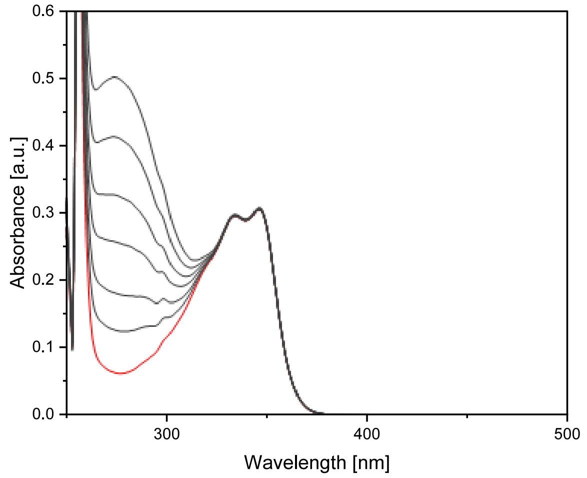

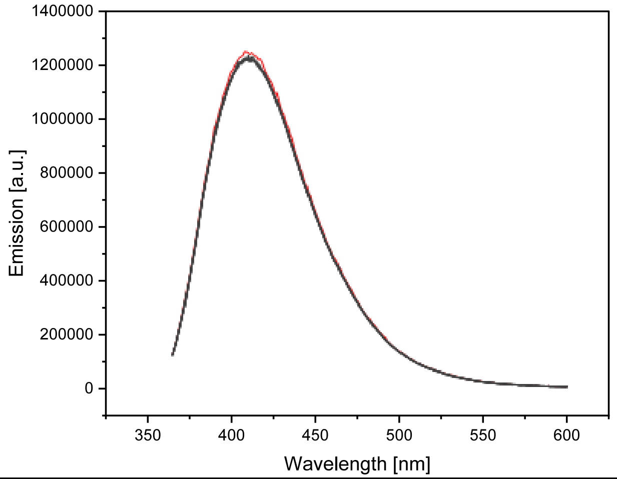

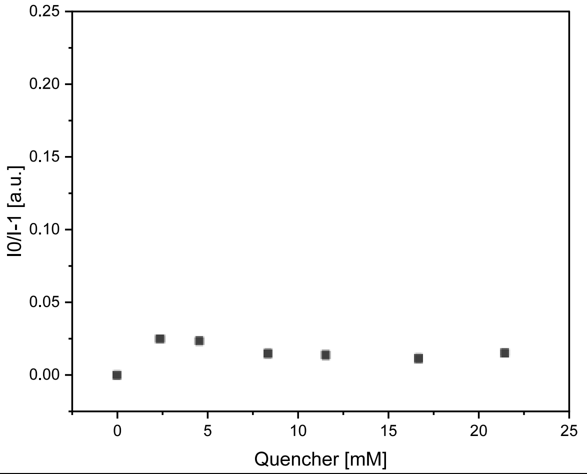

[Q] [mM]	[Q] [equiv]	I (440 nm)	$I_0/I - 1$
0.0	0	839819	0.000
2.4	48	819560	0.025
4.5	91	820709	0.023
8.3	167	827566	0.015
11.5	231	828395	0.014
16.7	333	830384	0.011
21.4	429	827236	0.015

Figure S14. Fluorescence quenching studies of the photocatalyst **NQH$_2$-1** with acetone. Changes in UV-Vis absorbance spectra upon quencher addition (top left), Changes in the fluorescence emission spectra upon quencher addition (top right), Stern-Volmer plot (bottom left) and underlying data (bottom right) of the quenching study.

7.4 Cyclic voltammetry of catalysts

NQH$_2$-1

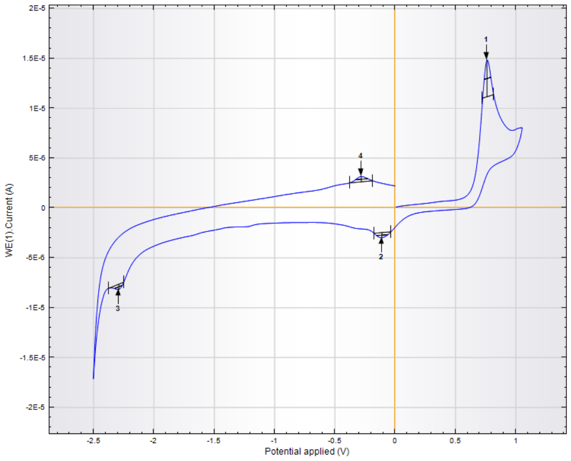

Index	Peak position
1	0.76035
2	-0.11078
3	-2.2911
4	-0.28198

NQH$_2$-1 + ferrocene

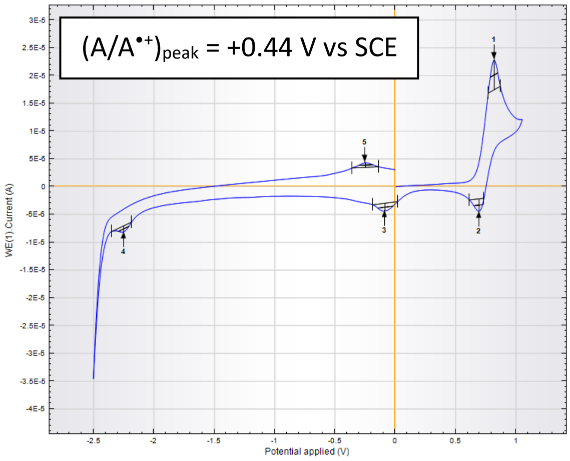

Index	Peak position
1	**0.82077** (Peak + Fc)
2	0.69489 (Fc)
3	-0.085602
4	-2.2508
5	-0.24673

NQH$_2$-1 + TMG

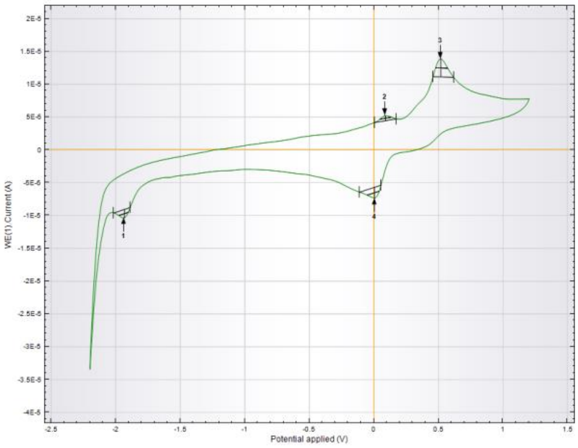

Index	Peak position
1	-1.9336
2	0.090637
3	0.51865
4	0.0050354

NQH$_2$-1 + TMG + ferrocene

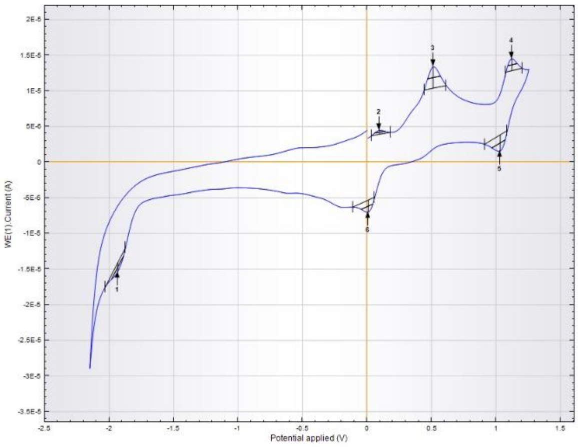

Index	Peak position
1	-1.9336
2	**0.095673**
3	0.51361
4	1.1229 (Fc)
5	1.0323 (Fc)
6	**0.010071**

NQH_2-2

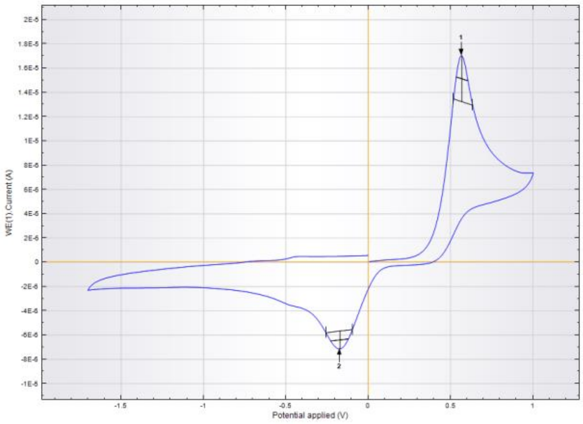

**Index**	**Peak position**
1	0.569
2	-0.1712

NQH_2-2 + ferrocene

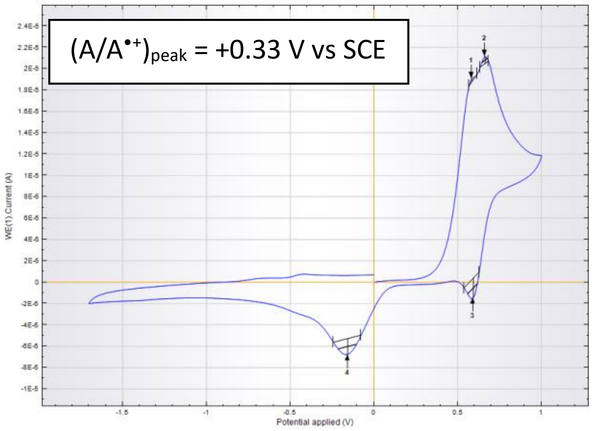

**Index**	**Peak position**
1	**0.58411**
2	0.66467 (Fc)
3	0.59418 (Fc)
4	-0.1561

NQH_2-2 + TMG

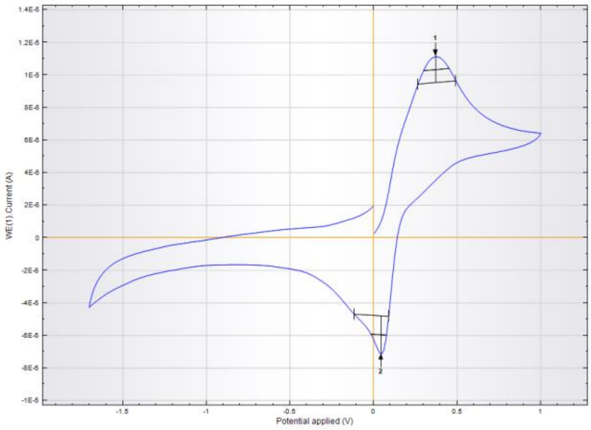

**Index**	**Peak position**
1	0.37262
2	0.045319

NQH_2-2 + TMG + ferrocene

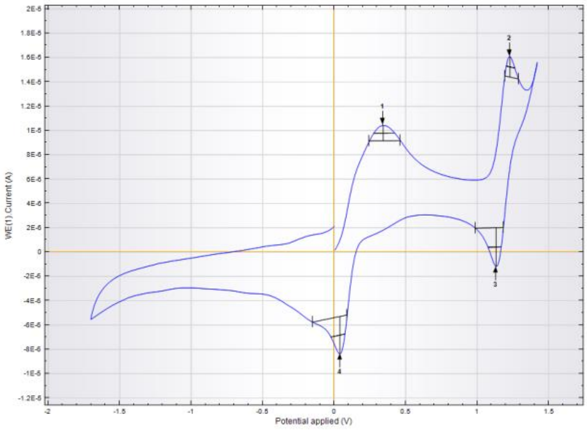

**Index**	**Peak position**
1	0.34241
2	1.2286 (Fc)
3	1.133 (Fc)
4	0.040283

NQH_2-3

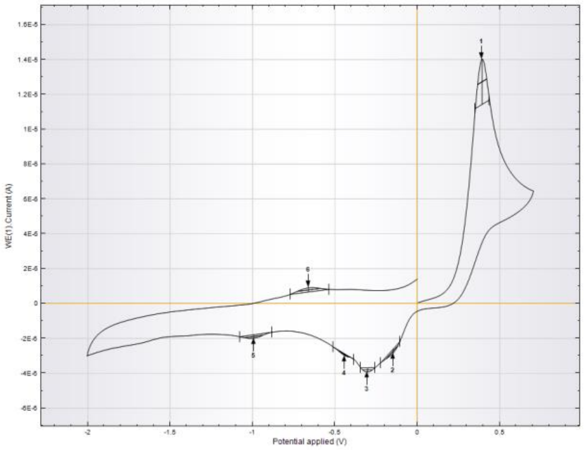

**Index**	**Peak position**
1	0.39276
2	-0.14603
3	-0.30212
4	-0.44312
5	-0.99197
6	-0.65964

NQH_2-3 + ferrocene

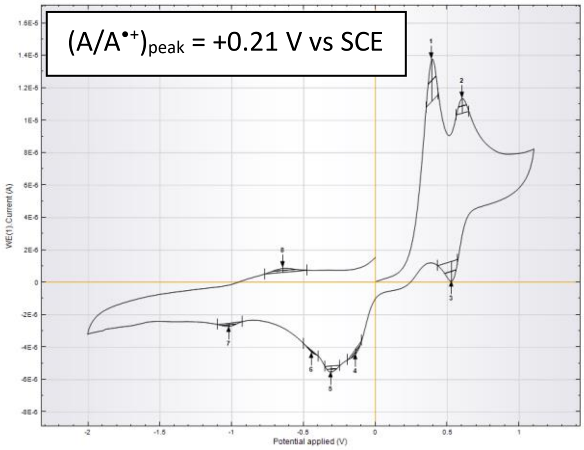

**Index**	**Peak position**
1	**0.39276**
2	0.60425 (Fc)
3	0.52872 (Fc)
4	-0.13596
5	-0.30716
6	-0.44312
7	-1.0172
8	-0.64453

NQH_2-3 + TMG

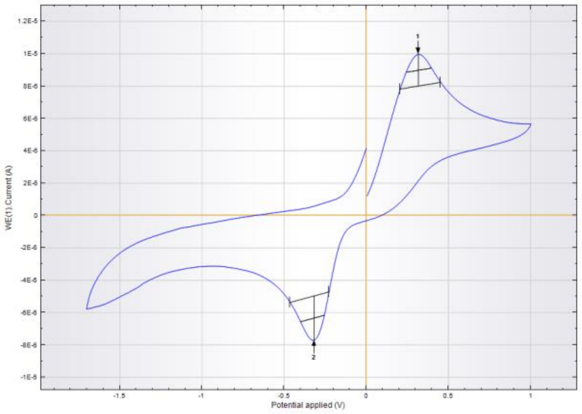

**Index**	**Peak position**
1	0.31723
2	-0.31723

NQH_2-3 + TMG + ferrocene

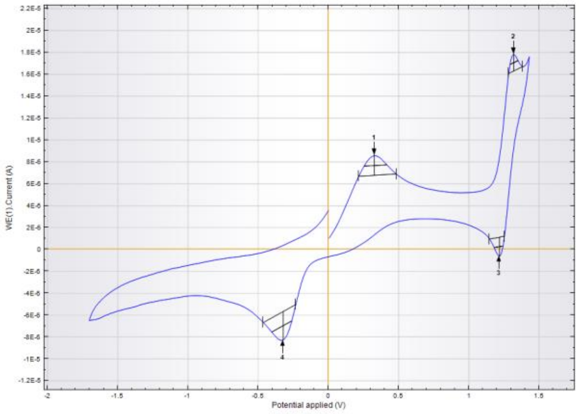

**Index**	**Peak position**
1	0.3273
2	1.3193 (Fc)
3	1.2186 (Fc)
4	-0.32227

NQH_2-4

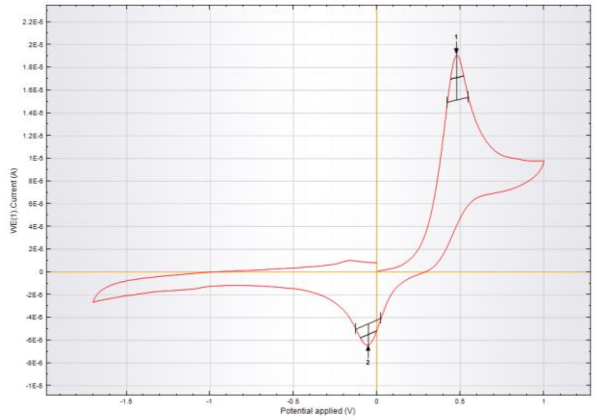

**Index**	**Peak position**
1	0.47836
2	-0.050354

NQH_2-4 + ferrocene

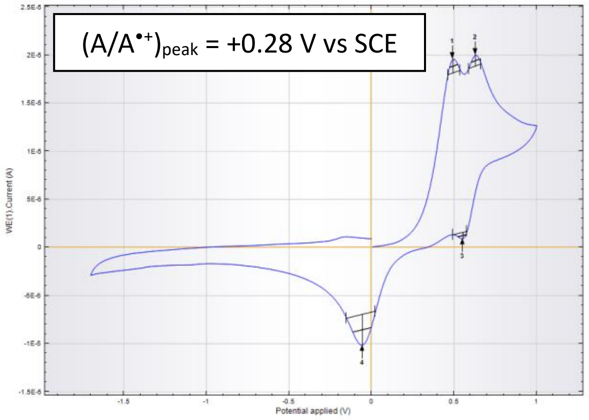

**Index**	**Peak position**
1	**0.49347**
2	0.62943 (Fc)
3	0.55389 (Fc)
4	-0.055389

NQH_2-4 + TMG

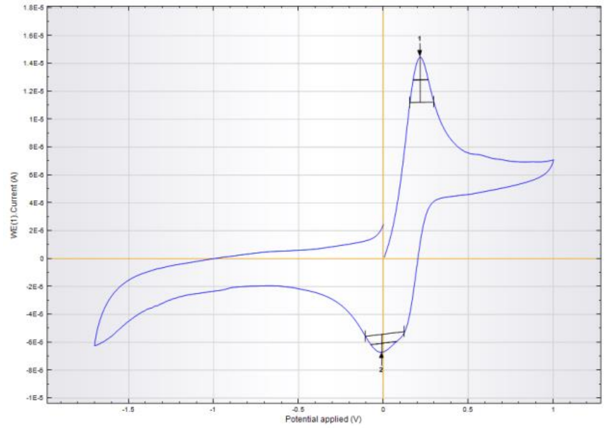

**Index**	**Peak position**
1	0.21652
2	-0.010071

NQH_2-4 + TMG + ferrocene

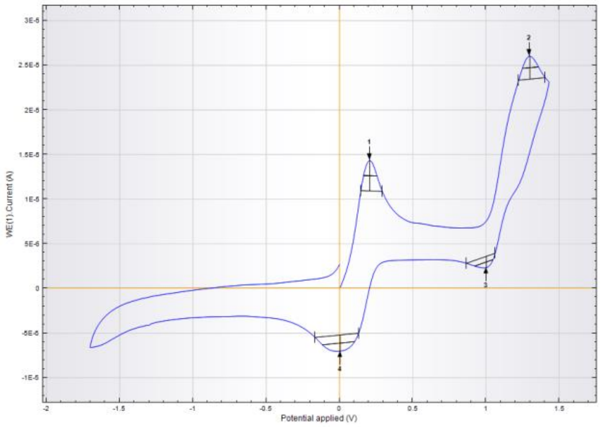

**Index**	**Peak position**
1	**0.20645**
2	1.2991 (Fc)
3	1.002 (Fc)
4	**0.0050354**

NQ-1

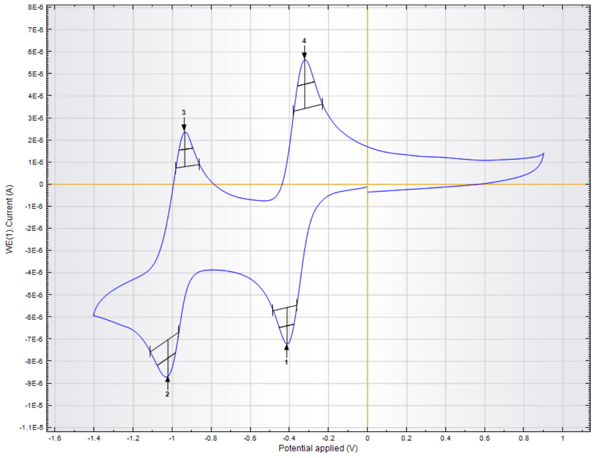

Index	Peak position
1	-0.4129
2	-1.0222
3	-0.93658
4	-0.32227

NQ-1 + ferrocene

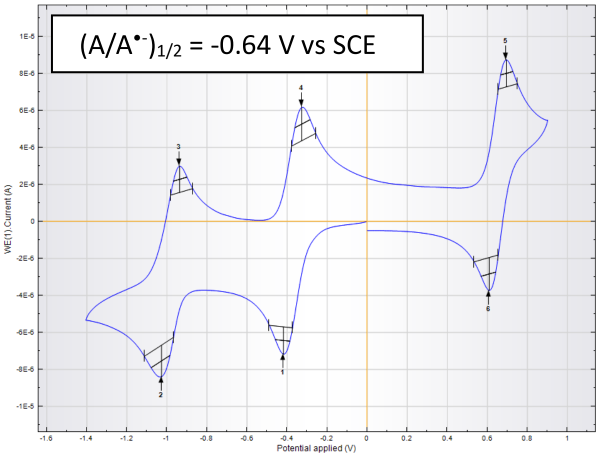

Index	Peak position
1	**-0.41794**
2	-1.0272
3	-0.93658
4	**-0.3273**
5	0.69489 (Fc)
6	0.60928 (Fc)

NQ-2

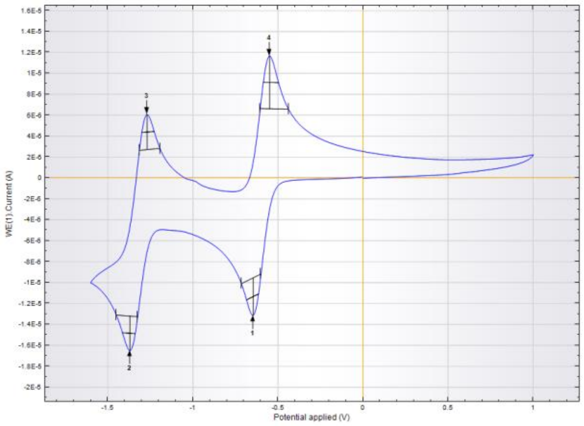

Index	Peak position
1	-0.64453
2	-1.3696
3	-1.2689
4	-0.54886

NQ-2 + ferrocene

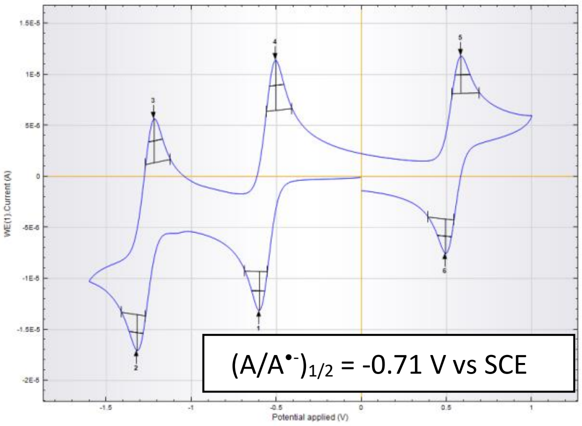

Index	Peak position
1	**-0.59921**
2	-1.3193
3	-1.2186
4	**-0.50354**
5	0.58411 (Fc)
6	0.49347 (Fc)

NQ-3

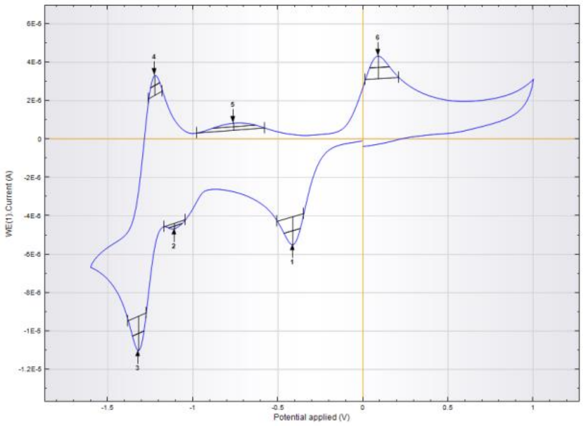

Index	Peak position
1	-0.4129
2	-1.1078
3	-1.3193
4	-1.2236
5	-0.76035
6	0.090637

NQ-3 + ferrocene

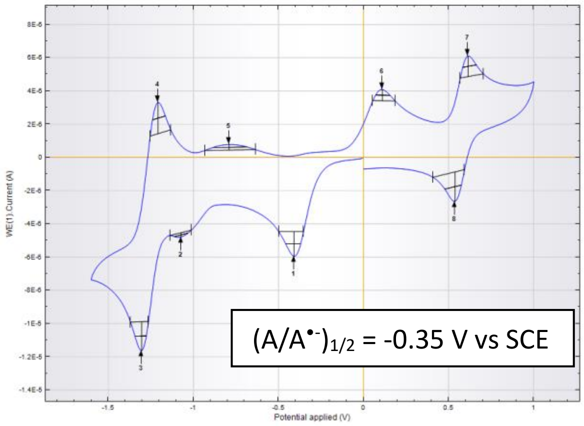

Index	Peak position
1	**-0.40787**
2	-1.0725
3	-1.3042
4	-1.2085
5	-0.79056
6	**0.11078**
7	0.61432 (Fc)
8	0.53879 (Fc)

NQ-4

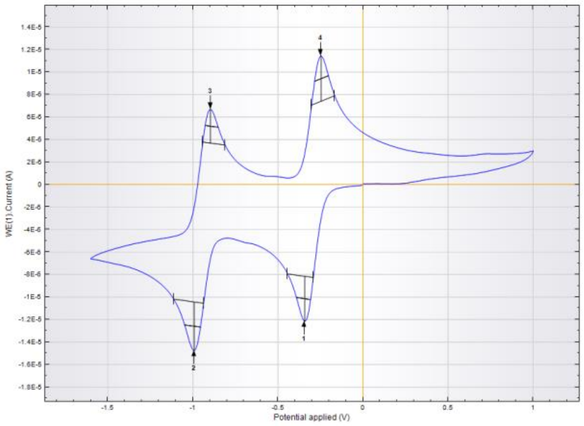

Index	Peak position
1	-0.34241
2	-0.99197
3	-0.8963
4	-0.24673

NQ-4 + ferrocene

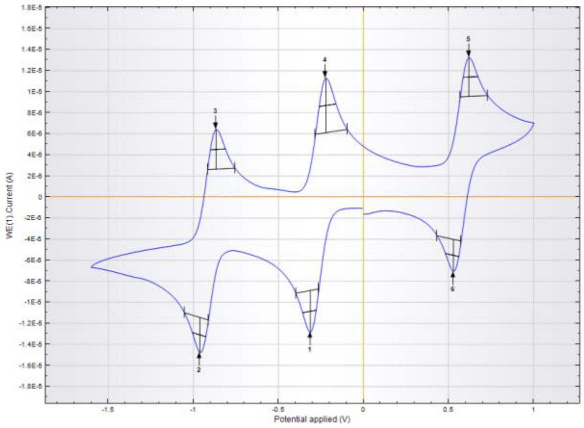

Index	Peak position
1	**-0.31219**
2	-0.96176
3	-0.86609
4	**-0.22156**
5	0.61935 (Fc)
6	0.52872 (Fc)

$(A/A^{\bullet -})_{1/2}$ = -0.46 V vs SCE

2-SAS

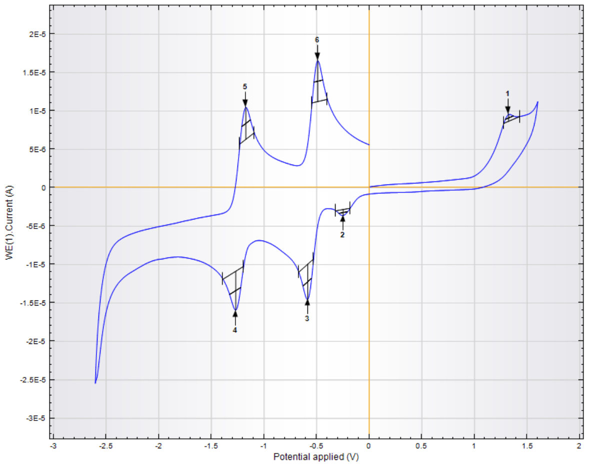

Index	Peak position
1	1.3243
2	-0.24673
3	-0.58411
4	-1.2689
5	-1.1732
6	-0.48843

2-SAS + ferrocene

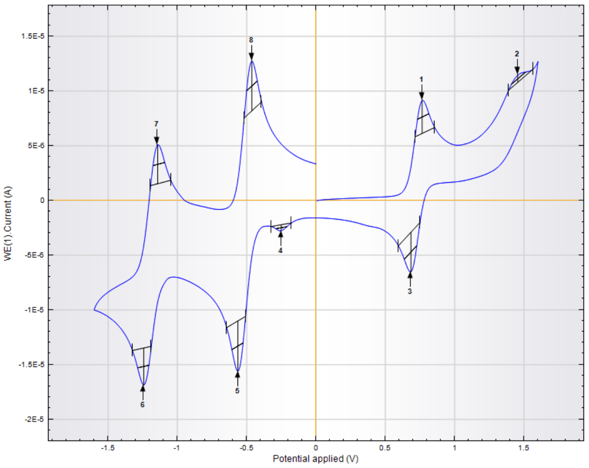

Index	Peak position
1	0.76538 (Fc)
2	1.4552
3	0.68481 (Fc)
4	-0.25177
5	**-0.56396**
6	-1.2437
7	-1.143
8	**-0.46326**

$(A/A^{\bullet-})_{1/2}$ = -0.86 V vs SCE

7.5 Cyclic voltammetry of starting materials

The reduction potentials of employed starting materials were determined by CV or are reported in literature. Ferrocene was added as internal reference. Obtained potentials vs. Fc/Fc+ were converted to yield approximated potentials against saturated calomel electrode.[9]

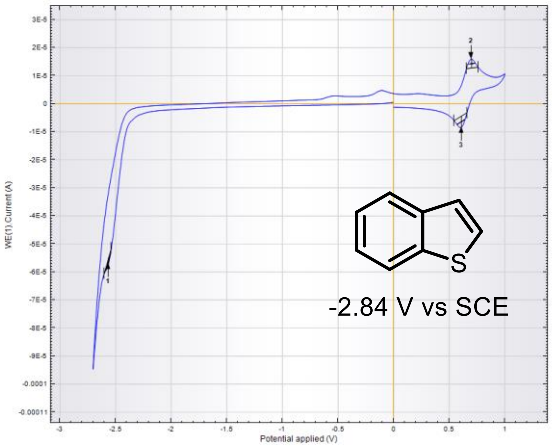

Index	Peak position
1	**-2.563**
2	0.69992 (Fc)
3	0.60928 (Fc)

7.6 Spectroelectrochemistry of catalyst NQH2-1

Spectroelectrochemical measurements were conducted on a solution of photocatalyst **NQH2-1** in degassed DMSO without base (Figure S15). A potential cycle was performed starting from +0.3 V with turning points at +1.5 V in positive direction and -0.8 V in negative direction. The UV-Vis spectra at indicated potential values show a clean oxidation from the hydroquinone to the quinone form at positive potentials and a clean reduction from the quinone form back to the hydroquinone form at negative potentials.

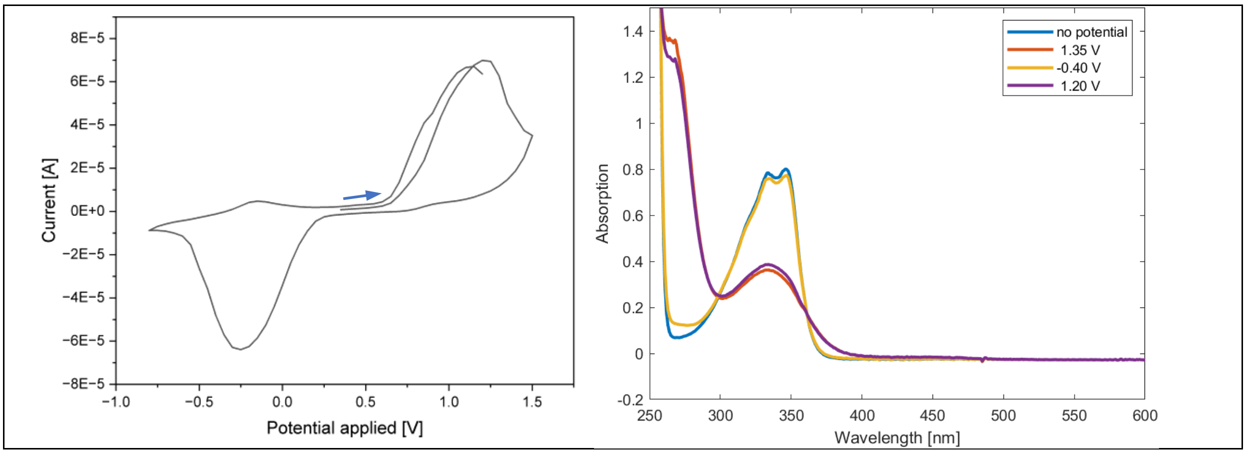

Figure S15. Cyclic voltammogram (left) and UV-Vis spectra at indicated potentials (right) of the spectroelectroscopy measurement of **NQH2-1**.

A mixture of photocatalyst **NQH2-1** with TMG (5 equiv) as a base was measured in degassed DMSO (Figure S16). A potential cycle was performed starting from -0.3 V with turning points at +0.8 V in positive direction and -0.8 V in negative direction. The UV-Vis spectra at indicated potential values show the formation of a species with a red-shifted absorption maximum at approximately 480 nm upon oxidation. At negative potentials, the species is reduced and shows a different UV-Vis signature. A possible explanation for this could be the formation of a stabilized anionic form of the photocatalyst.

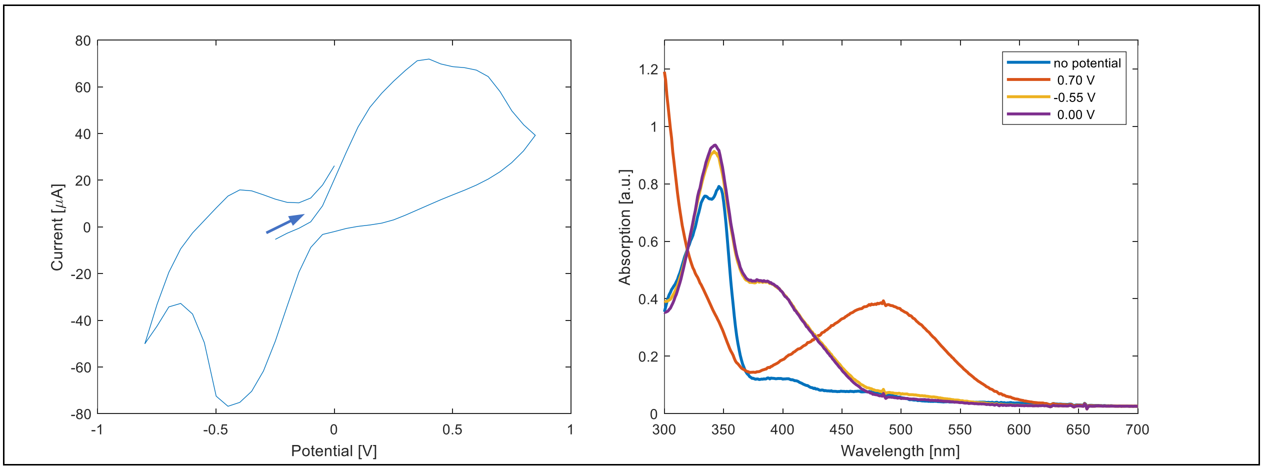

Figure S16. Cyclic voltammogram (left) and UV-Vis spectra at indicated potentials (right) of the spectroelectroscopy measurement of **NQH2-1** with TMG as added base.

A mixture of photocatalyst **NQ-1** was measured in degassed DMSO (Figure S17). A potential cycle was performed starting from 0.0 V with turning points at -0.75 V in negative direction and 0.15 V in positive direction. Starting from the quinone form, the photocatalyst is oxidized to its semiquinone radical form with a red-shifted UV-Vis absorption spectra at negative potentials. The semiquinone can be oxidized again to its quinone form when positive potentials are applied. When the same mixture was measured at more negative potentials with a turning point at -1.5 V another species with a UV-Vis absorption maximum at approximately 405 nm formed. It is currently believed that this is a dianionic

form of the photocatalyst. The second reduction was not reversible under spectroelectroscopy conditions but was shown to be reversible under normal cyclic voltammetry measurements. This can be explained by the altered diffusion of the solute in the OTTLE cell.

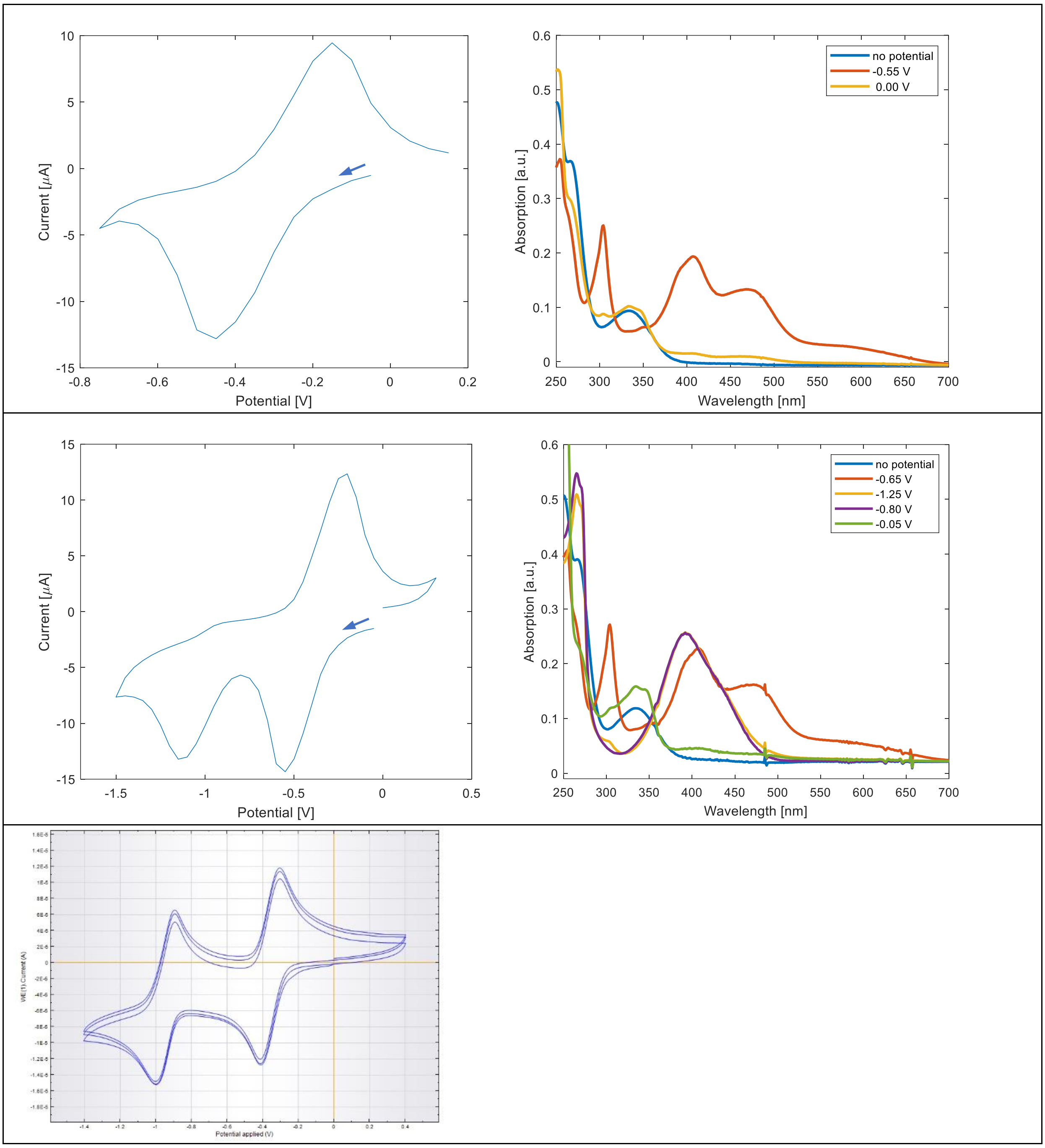

Figure S17. Cyclic voltammogram (left) and UV-Vis spectra at indicated potentials (right) of the spectroelectroscopy measurement of **NQ-1** in DMSO with in negative potential direction to -0.75 V (top) and further to -1.5 V (bottom). The cyclic voltammogram was retained even after three cycles (bottom).

7.7 Photostability measurements

The photostability of the catalyst in solution was monitored upon irradiation with 405 nm light (Figure S18). A fluorescence cuvette with 67 μM **NQH$_2$-1** in dry DMSO and subjected to irradiation under stirring while the UV-Vis absorbance was continuously measured. **NQH$_2$-1** was tested under nitrogen atmosphere and irradiation with (top left) and without presence of base (top right). To investigate whether the catalyst would go from the hydroquinone form to the quinone form under oxygen, the photocatalyst was measured under oxygen atmosphere with (bottom left) and without irradiation (bot-

tom right). The absorbance was measured every 30 minutes. The catalyst is not stable under irradiation or air and transforms into a mixture of species with similar absorbance under different conditions.

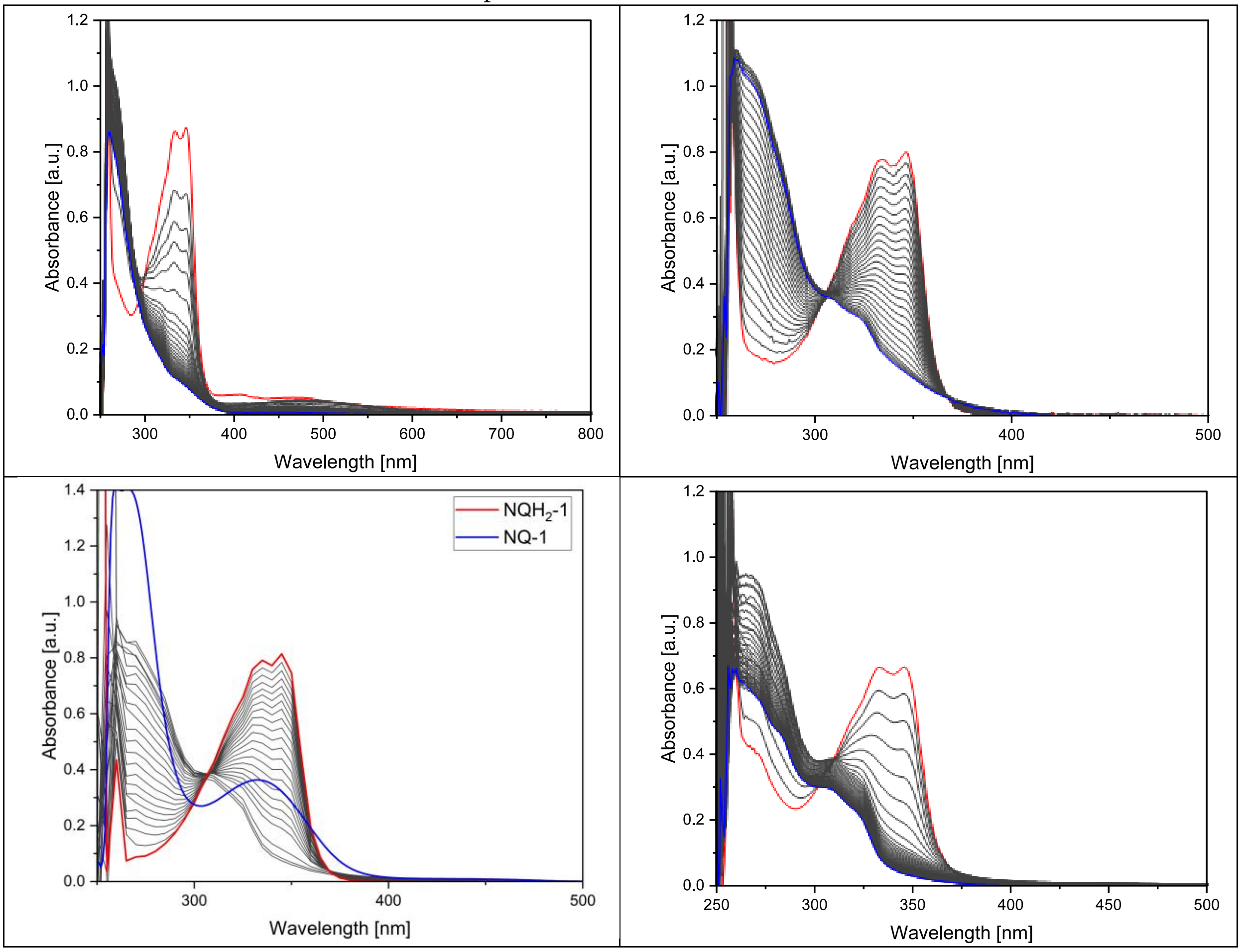

Figure S18. Photostability measurements of photocatalyst **NQH₂-1** under nitrogen atmosphere with (top left) and without presence of base (TMG) (top right), and under oxygen atmosphere with (bottom left) and without irradiation at 405 nm (bottom right).

If the naphthoquinone form of the catalyst **NQ-1** is irradiated at 405 nm with an electron donor (DIPEA 5.0 equiv) under nitrogen atmosphere in DMSO, the hydroquinone derivate is being formed by reduction (Figure S19, left). If irradiated only under oxygen atmosphere in DMSO, photoirradiation products with a similar absorbance as photo-transformed NQH_2-1 are formed (Figure S19, right)

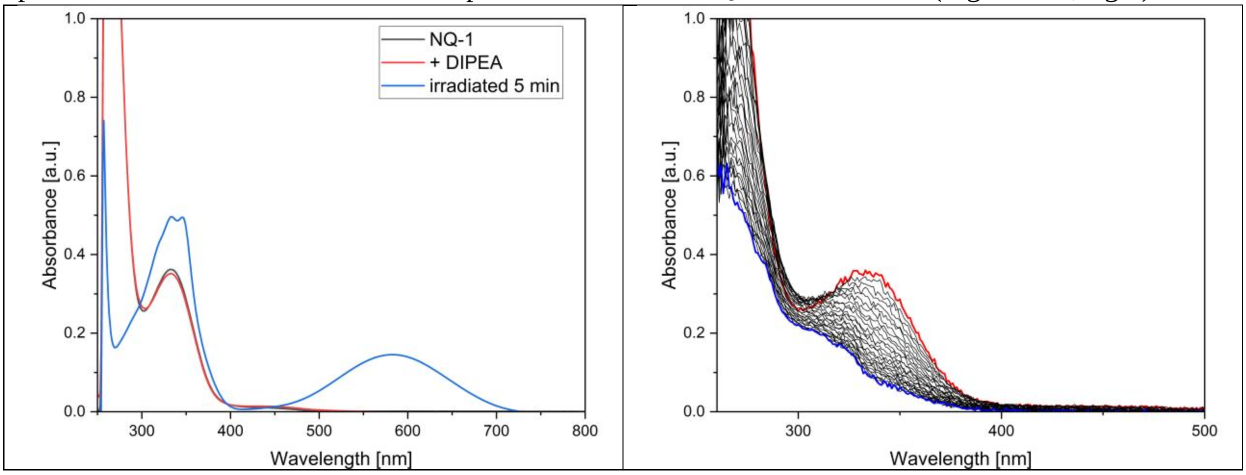

Figure S19. Formation of the naphthohydroquinone **NQH_2-1** from **NQ-1** upon photoirradiation in DMSO with added electron donor (DIPEA). The mixture (67 µM **NQ-1** in DMSO) was irradiated with a 405 nm LED in a 5 ml crimp vial and aliquot samples were drawn and measured in DMSO. The absorbance at 580 nm likely stems from an anionic naphthohydroquinone species forming upon reduction with DIPEA.

8. Characterization of the products

8.1 Photooxidative transformations

1,3,5-trimethoxy-2-(trifluoromethyl)benzene 1a

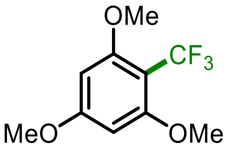

The title compound was synthesized following General Procedure B using 1,3,5-trimethoxybenzene (20.0 mg, 0.12 mmol, 1.0 equiv) and sodium triflinate (47.0 mg, 0.30 mmol, 2.5 equiv).
The yield of the desired product was determined by ^{19}F NMR of the crude reaction mixture using fluorobenzene (10 µl, 0.10 mmol) as an internal standard. NMR-yield: 74% (2-CF_3) + 26% (2,4-di-CF_3).
^{19}F NMR (377 MHz, $CDCl_3$) δ [ppm] = -53.93 (2-CF_3), -55.37 (2,4-di-CF_3), -113.15 (standard).
The spectral data was compared to literature values.[10]
With 2-SAS was used as photocatalyst irradiated at 450 nm and an oxygen filled balloon instead of air inlets. The yield of the desired product was determined by calibrated GC-FID measurements with 4-methylanisole (10 µl, 0.08 mmol) as internal standard: GC-yield: >95%.
GC-FID retention time [min] = 9.13 (standard), 12.94 (starting material), 13.24 (TMB-$(CF_3)_2$), 13.63 (product).
GC-MS retention time [min] = 11.57 (starting material, m/z = 168, calcd = 168), 11.88 (TMB-$(CF_3)_2$, m/z = 304, calcd = 304), 12.21 (product, m/z = 236, calcd = 236).
To isolate the compound, the combined contents of four reactions run in parallel with 2-SAS as catalyst were transferred with water and brine to an extraction funnel, extracted with ethyl acetate (3 × 15 ml), the combined organic phases dried over $MgSO_4$ and concentrated. The crude was purified by flash column chromatography (PE/EA 30%) to obtain **1a** as white solid (59 mg, 0.25 mmol, 62%)
^{1}H NMR (400 MHz, $CDCl_3$) δ [ppm] = 6.13 (s, 2H), 3.84 (s, 9H).
^{13}C NMR (101 MHz, $CDCl_3$) δ [ppm] = 163.64, 160.57 (q, *J*=1.2), 124.47 (q, *J*=272.4), 100.57 (q, *J*=30.0), 91.40, 56.40, 55.52.
^{19}F NMR (376 MHz, $CDCl_3$) δ [ppm] = -54.68.
The spectral data was compared to literature values.[10]

1,3-dimethoxy-4-(trifluoromethyl)benzene 1b

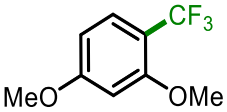

The title compound was synthesized following General Procedure B using 1,3-dimethoxybenzene (14 µl, 0.10 mmol, 1.0 equiv) and sodium triflinate (20.3 mg, 0.13 mmol, 1.3 equiv). The yield of the desired product was determined by ^{19}F NMR of the crude reaction mixture with fluorobenzene (25 µl, 0.265 mmol) used as an internal standard. NMR-yield: 59% (45% 4-CF_3 + 14% 2-CF_3).
^{19}F NMR (377 MHz, $CDCl_3$) δ [ppm] = -54.73 (2-CF_3), -61.11 (4-CF_3), -113.15 (standard).
The spectral data was compared to reported values.[11]
GC-MS: product 4-CF_3 (m/z = 206, calcd = 206), product 2-CF_3 (m/z = 206, calcd = 206).

1-methoxy-2-(trifluoromethyl)benzene 1c

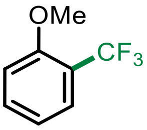

The title compound was synthesized following General Procedure B using anisole (11 µl, 0.10 mmol, 1.0 equiv) and sodium triflinate (20.3 mg, 0.13 mmol, 1.3 equiv). The yield of the desired product was

determined by ^{19}F NMR of the crude reaction mixture with fluorobenzene (25 µl, 0.265 mmol) used as an internal standard. NMR-yield: 32% (11% *p*-CF_3-anisole + 21% *o*-CF_3-anisole).
^{19}F NMR (377 MHz, $CDCl_3$) δ [ppm] = -61.32 (*p*-CF_3-anisole), -62.30 (*o*-CF_3-anisole), -113.15 (standard).
The spectral data was compared to reported values.[12]
GC-MS: *p*-CF_3-anisole (m/z = 176, calcd = 176), *o*-CF_3-anisole (m/z = 176, calcd = 176), anisole-$(CF_3)_2$ (m/z = 244, calcd = 244).

1-bromo-4-methoxy-2-(trifluoromethyl)benzene 1d

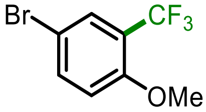

The title compound was synthesized following General Procedure B using 1-bromo-4-methoxybenzene (14 µl, 0.11 mmol, 1.0 equiv) and sodium triflinate (47 mg, 0.3 mmol, 2.7 equiv). The yield of the desired product was determined by ^{19}F NMR of the crude reaction mixture with fluorobenzene (25 µl, 0.265 mmol) as an internal standard. NMR-yield: 40% (2-CF_3) + 4% (3-CF_3).
^{19}F NMR (377 MHz, $CDCl_3$) δ [ppm] = -62.70 (2-CF_3, product), -62.80 (3-CF_3, product), -113.15 (standard).
The spectral data was compared to literature values.[13,14]
To isolate the compound, the combined contents of three reactions run in parallel were transferred with water and brine to a separatory funnel, extracted with diethyl ether (3 × 15 ml), dried over $MgSO_4$, filtered, and concentrated. The crude was purified by flash column chromatography (pentane/acetone 0-30%) to yield **1d** as colorless oil (34%, 26 mg, 0.10 mmol).
^{1}H NMR (400 MHz, $CDCl_3$) δ [ppm] = 7.67 (d, *J*=2.5, 1H), 7.59 (dd, *J*=8.8, 2.5, 1H), 6.89 (d, *J*=8.8, 1H), 3.89 (s, 3H).
^{19}F NMR (376 MHz, $CDCl_3$) δ [ppm] = -63.34.
^{13}C NMR (101 MHz, $CDCl_3$) δ [ppm] = 156.80 (q, *J*=0.1), 136.11, 130.21 (q, *J*=5.5), 122.83 (q, *J*=272.9), 120.65 (d, *J*=31.6), 113.93, 112.19, 56.34.
GC-MS: product (m/z = 254, calcd = 254).

1-chloro-4-methoxy-2-(trifluoromethyl)benzene 1e

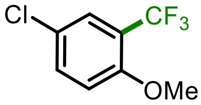

The title compound was synthesized following General Procedure B using 1-chloro-4-methoxybenzene (14 µl, 0.11 mmol, 1.0 equiv) and sodium triflinate (47 mg, 0.3 mmol, 2.7 equiv). The yield of the desired product was determined by ^{19}F NMR of the crude reaction mixture with an internal standard (fluorobenzene, 25 µl, 0.265 mmol). NMR-yield: 43% (2-CF_3, product) + 14% (3-CF_3, product).
^{19}F NMR (377 MHz, $CDCl_3$) δ [ppm] = -61.00 ($(CF_3)_2$-sideproduct), -62.73 (2-CF_3, product), -62.77 (3-CF_3, product), -113.15 (standard).
The spectral data was compared to literature values.[14]
GC-MS: $(CF_3)_2$-sideproduct (m/z = 278 calcd = 278), product (m/z = 210, calcd = 210).

Trifluortoluene 1f

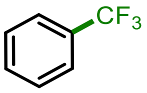

The title compound was synthesized following General Procedure B using benzene (9 µl, 0.10 mmol, 1.0 equiv) and sodium triflinate (20.3 mg, 0.13 mmol, 1.3 equiv). The yield of the desired product was

determined by ^{19}F NMR of the crude reaction mixture with fluorobenzene (25 µl, 0.265 mmol) used as an internal standard. NMR-yield: 40%.
^{19}F NMR (377 MHz, $CDCl_3$) δ [ppm] = -62.60 (product), -113.15 (standard).
The spectral data was compared to reported values.[12]
GC-MS: product (m/z = 146, calcd = 146), benzene-$(CF_3)_2$ (m/z = 214, calcd = 214).

2,6-dimethoxy-3-(trifluoromethyl)pyridine 1g

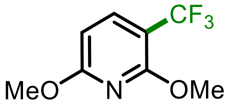

The title compound was synthesized following General Procedure B using 2,6-dimethoxypyridine (13.5 µl, 0.10 mmol, 1.0 equiv) and sodium triflinate (47 mg, 0.3 mmol, 3.0 equiv). The yield of the desired product was determined by ^{19}F NMR of the crude reaction mixture using fluorobenzene (25 µl, 0.265 mmol) as an internal standard. NMR-yield: 71% (3-CF_3, product) + 29% (3,3-$(CF_3)_2$, side-product).
^{19}F NMR (377 MHz, $CDCl_3$) δ [ppm] = -61.73 (3-CF_3, product), -62.16 (3,3-$(CF_3)_2$, sideproduct), -113.15 (standard).
The spectral data was compared to literature values.[15]
GC-MS: 3,3-$(CF_3)_2$ (m/z = 275, calcd = 275), product (m/z = 207, calcd = 207).

2-(trifluoromethyl)pyridine 1h

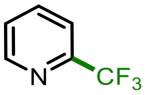

The title compound was synthesized following General Procedure B using pyridine (8 µl, 0.10 mmol, 1.0 equiv) and sodium triflinate (47 mg, 0.3 mmol, 3.0 equiv). The yield of the desired product was determined by ^{19}F NMR of the crude reaction mixture with fluorobenzene (25 µl, 0.265 mmol) as an internal standard. NMR-yield: 22 % (2-CF_3, product) + 17 % (3-CF_3, product).
^{19}F NMR (377 MHz, $CDCl_3$) δ [ppm] = -62.56 (3-CF_3, product), -68.02 (2-CF_3, product), -113.15 (standard).
The spectral data was compared to literature values.[15]
GC-MS: product 3-CF_3 (m/z = 147, calcd = 147), product 2-CF_3 (m/z = 147, calcd = 147).

3-(trifluoromethyl)isonicotinonitrile 1i

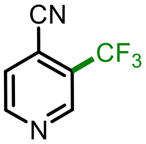

The title compound was synthesized following General Procedure B using isonicotinonitrile (12.8 mg, 0.12 mmol, 1.0 equiv) and sodium triflinate (47 mg, 0.3 mmol, 2.5 equiv). The yield of the desired product was determined by ^{19}F NMR of the crude reaction mixture using fluorobenzene (25 µl, 0.265 mmol) as an internal standard. NMR-yield: 35 % (3-CF_3, product) + 12 % (2-CF_3, product).
^{19}F NMR (377 MHz, $CDCl_3$) δ [ppm] = -61.64 (3-CF_3, product), -68.32 (2-CF_3, product), -113.15 (standard).
The spectral data was compared to literature values.[16]
GC-MS: product (m/z = 172, calcd = 172), product m/z = 172, calcd = 172).

2,4,6-trimethoxy-5-(trifluoromethyl)pyrimidine 1j

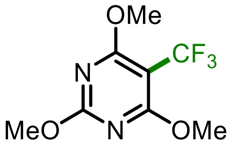

The title compound was synthesized following General Procedure B using 2,4,6-trimethoxypyrimidine (17.6 mg, 0.10 mmol, 1.0 equiv) and sodium triflinate (47 mg, 0.3 mmol, 3.0 equiv). The yield of the desired product was determined by ^{19}F NMR of the crude reaction mixture using fluorobenzene (25 µl, 0.265 mmol) as an internal standard. NMR-yield: 83% (product).
^{19}F NMR (377 MHz, $CDCl_3$) δ [ppm] = -55.78 (product), -113.15 (standard).
The spectral data was compared to literature values.[7]
GC-MS: product (m/z = 238, calcd = 238).

1,3-dimethyl-5-(trifluoromethyl)pyrimidine-2,4(1H,3H)-dione 1k

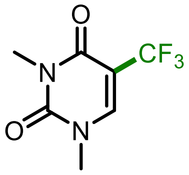

The title compound was synthesized following General Procedure B using 1,3-dimethylpyrimidine-2,4(1H,3H)-dione (14.2 mg, 0.10 mmol, 1.0 equiv) and sodium triflinate (47 mg, 0.3 mmol, 3.0 equiv). The yield of the desired product was determined by ^{19}F NMR of the crude reaction mixture with fluorobenzene (10 µl, 0.106 mmol) as an internal standard. NMR-yield: 78% (product).
^{19}F NMR (377 MHz, $CDCl_3$) δ [ppm] = -63.53 (product), -113.15 (standard).
The spectral data was compared to literature values.[7]
GC-MS: product (m/z = 208, calcd = 208).

8-(trifluoromethyl)-9H-purin-6-amine 1l

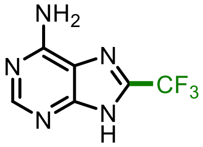

The title compound was synthesized following General Procedure B using adenine (14.6 mg, 0.11 mmol, 1.0 equiv) and zinc(II) difluoromethanesulfinate (48 mg, 0.16 mmol, 1.5 equiv). The yield of the desired product was determined by ^{19}F NMR of the crude reaction mixture with fluorobenzene (25 µl, 0.265 mmol) as an internal standard. NMR-yield: 46% (product).
^{19}F NMR (377 MHz, $CDCl_3$) δ [ppm] = -64.31 (product), 113.15 (standard).
The spectral data was compared to literature values.[17]
GC-MS: product (m/z = 203, calcd = 203).

1,3,7-trimethyl-8-(trifluoromethyl)-3,7-dihydro-1H-purine-2,6-dione 1m

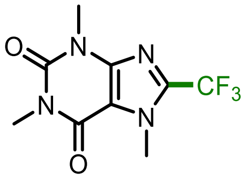

The title compound was synthesized following General Procedure B using caffeine (24.6 mg, 0.13 mmol, 1.0 equiv) and sodium triflinate (61 mg, 0.39 mmol, 3.0 equiv). The yield of the desired product was determined by ^{19}F NMR of the crude reaction mixture using fluorobenzene (25 µl, 0.265 mmol) as an internal standard. NMR-yield: 83% (product).
^{19}F NMR (377 MHz, $CDCl_3$) δ [ppm] = -62.34 (product), -113.15 (standard).

The spectral data was compared to literature values.[7]
GC-MS: product (m/z = 262, calcd = 262).
For gram scale synthesis the title compound was prepared following the large scale General Procedure B using caffeine (1.164 g, 6.0 mmol, 1.0 equiv), sodium triflinate (2.80 g, 18.0 mmol, 3.0 equiv), and photocatalyst **NQH$_2$-1** (156 mg, 0.6 mmol, 0.1 equiv). Following irradiation, the crude purified by column chromatography (PE/EA 70:30) to obtain the desired product **1m** as white powder (1.15 g, 4.37 mmol, 73%).
^{1}H NMR (400 MHz, CDCl$_3$) δ [ppm] = 4.15 (q, *J*=1.2, 1H), 3.58 (s, 1H), 3.41 (s, 1H).
^{13}C NMR (101 MHz, CDCl$_3$) δ [ppm] = 155.60, 151.47, 146.66, 139.05 (d, *J*=40.1), 118.33 (q, *J*=271.3), 109.78, 33.32 (q, *J*=2.1), 30.03, 28.33.
^{19}F NMR (377 MHz, CDCl$_3$) δ [ppm] = -62.92.
HRMS (EI+): exact mass calcd for $C_9H_9F_3N_4O_2^+$ [M$^+$]: m/z = 262.06741, found: 262.06756.

2-(difluoromethyl)-1,3,5-trimethoxybenzene 1n

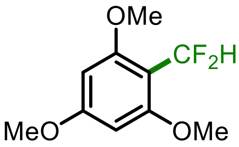

The title compound was synthesized following General Procedure B using 1,3,5-trimethoxybenzene (17.7 mg, 0.11 mmol, 1.0 equiv) and zinc(II) difluoromethanesulfinate (48 mg, 0.16 mmol, 1.5 equiv). The yield of the desired product was determined by ^{19}F NMR of the crude reaction mixture with fluorobenzene (25 µl, 0.265 mmol) as an internal standard. NMR-yield: 20% (product).
^{19}F NMR (377 MHz, CDCl$_3$) δ [ppm] = -113.15 (standard), -114.66 (product).
The spectral data was compared to literature values.[18]
GC-MS: product (m/z = 218, calcd = 218).

5-(difluoromethyl)-1,3-dimethylpyrimidine-2,4(1H,3H)-dione 1o

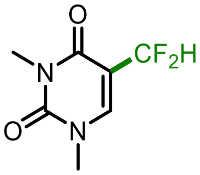

The title compound was synthesized following General Procedure B using 1,3-dimethylpyrimidine-2,4(1H,3H)-dione (17.1 mg, 0.12 mmol, 1.0 equiv) and zinc(II) difluoromethanesulfinate (54 mg, 0.18 mmol, 1.5 equiv). The yield of the desired product was determined by ^{19}F NMR of the crude reaction mixture with fluorobenzene (10 µl, 0.106 mmol) as an internal standard. NMR-yield: 24% (product).
^{19}F NMR (377 MHz, CDCl$_3$) δ [ppm] = -113.15 (standard), -117.13 (product).
The spectral data was compared to literature values.[19]
GC-MS: product (m/z = 190, calcd = 190).

8-(difluoromethyl)-1,3,7-trimethyl-3,7-dihydro-1H-purine-2,6-dione 1p

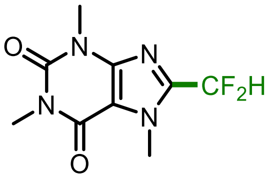

The title compound was synthesized following General Procedure B using caffeine (19.7 mg, 0.10 mmol, 1.0 equiv) and zinc(II) difluoromethanesulfinate (44 mg, 0.15 mmol, 1.5 equiv). The yield of the desired product was determined by ^{19}F NMR of the crude reaction mixture with an internal standard (fluorobenzene, 25 µl, 0.265 mmol): NMR-yield: 51% (product).
^{19}F NMR (377 MHz, CDCl$_3$) δ [ppm] = -113.15 (standard), -115.27 (product).

The spectral data was compared to literature values.[7]
GC-MS: product (m/z = 244, calcd = 244).

2-bromo-1,3,5-trimethoxybenzene 2a

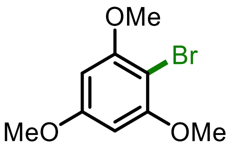

The title compound was synthesized following General Procedure B using 1,3,5-trimethoxybenzene (16.8 mg, 0.10 mmol, 1.0 equiv) and potassium bromide (60 mg, 0.50 mmol, 5.0 equiv). Additionally, trifluoracetic acid (1.5 µl, 0.02 mmol, 0.2 equiv) was added to the reaction mixture via syringe. The yield of the desired product was determined by calibrated GC-FID with 4-methylanisole (10 µl, 0.079 mmol) as an internal standard. GC-yield: 39% (product).
With sodium anthraquinone-2-sulfonate as photocatalyst irradiated at 450 nm, no trifluoracetic acid and a reaction time of 167 h : GC-yield: >95%.
GC-FID retention time [min] = 9.01 (standard), 12.87 (starting material), 15.33 (product).
GC-MS retention time [min] = 11.60 (starting material, m/z = 168, calcd = 168), 13.97 (product, m/z = 246, calcd = 246).
To isolate the compound the combined contents of four reactions run in parallel with 2-SAS as catalyst, were transferred with water and brine to an extraction funnel, extracted with ethyl acetate (3 × 15 ml), the combined organic phases dried over $MgSO_4$, filtered and concentrated. The crude was purified by flash column chromatography (PE/EA, 15-30%) to obtain **2a** as white solid (75 mg, 0.30 mmol, 77%).
^{1}H NMR (400 MHz, $CDCl_3$) δ [ppm] = 6.17 (s, 2H), 3.87 (s, 6H), 3.81 (s, 3H).
^{13}C NMR (101 MHz, $CDCl_3$) δ [ppm] = 160.60, 157.60, 92.14, 91.78, 56.49, 55.65.
The spectral data was compared to literature values.[7]

1-bromo-4-methoxybenzene 2b

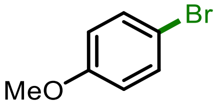

The title compound was synthesized following General Procedure B using anisole (11 µl, 0.10 mmol, 1.0 equiv) and potassium bromide (60 mg, 0.50 mmol, 5.0 equiv) in 1 ml of MeCN/H_2O (1:1). Additionally, trifluoracetic acid (7.7 µl, 0.1 mmol, 1.0 equiv) was added to the reaction mixture via syringe. The yield of the desired product was determined by GC-FID with an added internal standard (4-methyl-anisole, 10 µl, 0.079 mmol). For GC analysis, 1 ml of MeCN was added to the reaction mixture to prevent phase separation. GC-yield: 86% (product).
With 2-SAS as photocatalyst and irradiation at 450 nm under otherwise identical reaction conditions: GC-yield: >95%.
GC-FID retention time [min] = 7.63 (starting material), 8.95 (standard), 10.90 (product).
To isolate the product, the combined contents of three reactions run in parallel were transferred to a separatory funnel with water and brine and extracted with diethyl ether (3 × 15 ml). The combined organic phases were dried over $MgSO_4$ and concentrated. The crude was purified by flash column chromatography (PE 100%) to yield **2b** as colorless oil. (79%, 44.1 mg, 0.24 mmol).
^{1}H NMR (400 MHz, $CDCl_3$) δ [ppm] = 7.42 – 7.33 (m, 1H), 6.83 – 6.74 (m, 1H), 3.78 (s, 2H).
^{13}C NMR (101 MHz, $CDCl_3$) δ [ppm] = 158.85, 132.39, 115.88, 112.97, 55.59.

With **NQH$_2$-1** as photocatalyst (GC-FID)

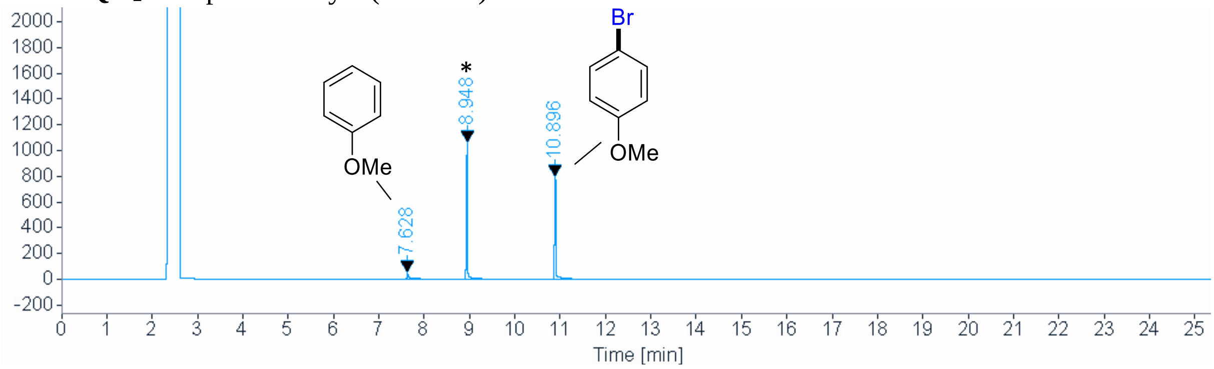

With **2-SAS** as photocatalyst (GC-FID)

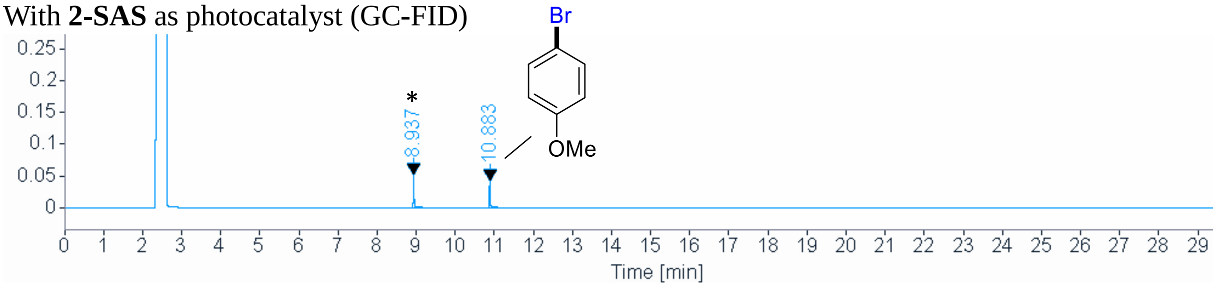

1,3,5-trimethoxy-2-thiocyanatobenzene 2c

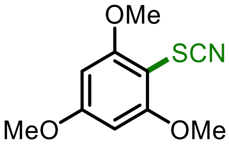

The title compound was synthesized following General Procedure B using 1,3,5-trimethoxybenzene (16.8, 0.10 mmol, 1.0 equiv) and sodium thiocyanate (40.5) mg, 0.50 mmol, 5.0 equiv). The yield of the desired product was determined by GC-FID with an added internal standard (4-methyl-anisole, 10 µl, 0.079 mmol). GC-yield: >95% (product).

The reaction also proceeded without added photocatalyst at 405 nm irradiation.

With 2-SAS as photocatalyst and irradiation at 450 nm under otherwise identical reaction conditions the reaction proceeded without background reaction : GC-yield: >95%.

GC-FID retention time [min] = 9.02 (standard), 12.83 (starting material), 17.13 (product).

GC-MS retention time [min] = 15.74 (product, m/z = 225, calcd = 225).

For isolation, the contents of two reactions with run in parallel with 2-SAS as catalyst were transferred to an extraction funnel with water and brine and extracted with EtOAc (3 × 10 ml). The organic phases were combined, dried over $MgSO_4$, and evaporated. The crude was purified by column chromatography (PE/EA, 20-30%) to yield **2c** as white powder (34 mg, 0.15 mmol, 75%).

^{1}H NMR (400 MHz, $CDCl_3$) δ [ppm] = 6.15 (s, 1H), 3.91 (s, 3H), 3.83 (s, 2H).

^{13}C NMR (101 MHz, $CDCl_3$) δ [ppm] = 164.37, 161.49, 111.96, 91.44, 89.81, 56.46, 55.70.

HRMS (EI+): exact mass calcd for $C_{10}H_{11}NO_3S^+$ [M$^+$]: m/z = 225.04542, found: 225.04516.

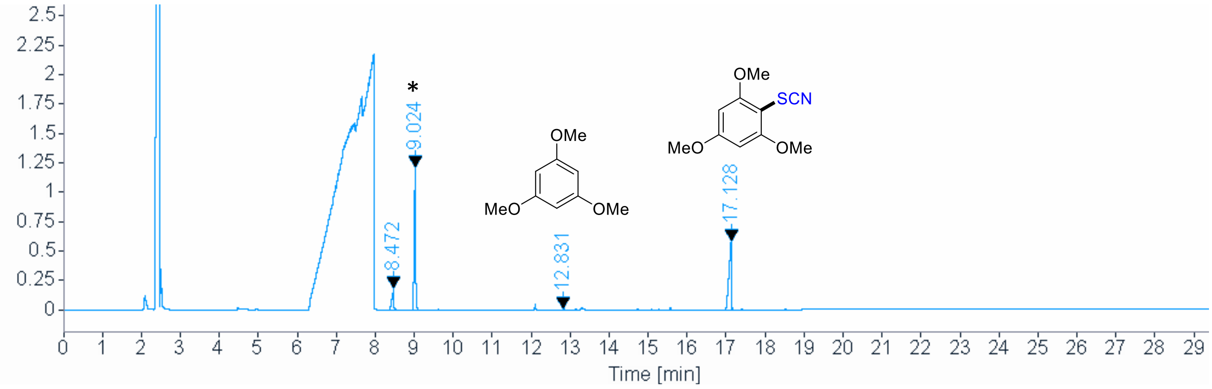

2,4-dimethoxy-1-thiocyanatobenzene 2d

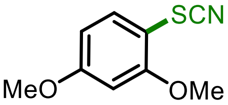

The title compound was synthesized following General Procedure B using 1,3-dimethoxybenzene (14 µl, 0.11 mmol, 1.0 equiv) and sodium thiocyanate (44.5) mg, 0.55 mmol, 5.0 equiv.

For isolation, the contents of two reactions with run in parallel were transferred to an extraction funnel with water and brine and extracted with EtOAc (3 × 10 ml). The organic phases were combined, dried over $MgSO_4$, and evaporated. The crude was purified by column chromatography (PE/EA, 20%) to yield the product as white powder (15 mg, 0.08 mmol, 38%).

^{1}H NMR (400 MHz, $CDCl_3$) δ [ppm] = 7.46 (d, *J*=8.6, 1H), 6.54 (dd, *J*=8.6, 2.5, 1H), 6.51 (d, *J*=2.5, 1H), 3.91 (s, 3H), 3.83 (s, 3H).

^{13}C NMR (101 MHz, $CDCl_3$) δ [ppm] = 163.16, 159.15, 133.98, 111.46, 106.29, 102.64, 99.73, 56.32, 55.80.

HRMS (EI+): exact mass calcd for $C_9H_9NO_2S^+$ [M^+]: m/z = 195.03485, found: 195.03518.

GC-FID retention time [min] = 8.97 (standard), 10.59 (starting material), 15.32 (product).

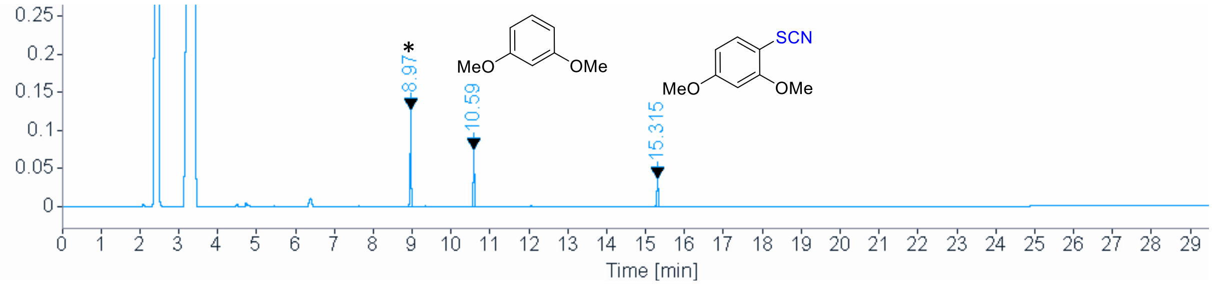

2,4,6-trimethoxybenzonitrile 2e

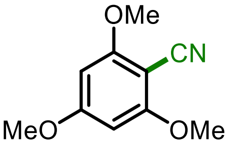

The title compound was synthesized following General Procedure B using 2-SAS as the photocatalyst, 1,3,5-trimethoxybenzene (23.4 mg, 0.14 mmol, 1.0 equiv) and potassium cyanide (20.3 mg, 0.31 mmol, 2.2 equiv). Water (0.2 ml) was added to the reaction to ensure better solubility of the cyanide salt, oxygen balloon was installed via syringe and the reaction was run for 93 hours. The yield of the desired product was determined by GC-FID. GC-yield: 66% (product).

GC-FID retention time [min] = 12.85 (starting material), 16.26 (product).

For isolation, the contents of two reactions run in parallel were transferred to an extraction funnel with water and brine and extracted with EtOAc (3 × 10 ml). The organic phases were combined, dried over

$MgSO_4$, and evaporated. The crude was purified by column chromatography (PE/EA, 20-100%) to yield **2e** as white powder (16 mg, 0.08 mmol, 41%).
^{1}H NMR (400 MHz, $CDCl_3$) δ [ppm] = 6.06 (s, 1H), 3.87 (s, 3H), 3.85 (s, 2H).
^{13}C NMR (101 MHz, $CDCl_3$) δ [ppm] = 165.47, 163.93, 114.72, 90.49, 84.24, 56.22, 55.81.
HRMS (EI+): exact mass calcd for $C_{10}H_{11}O_3^+$ [M^+]: m/z = 193.07334, found: 193.07323.

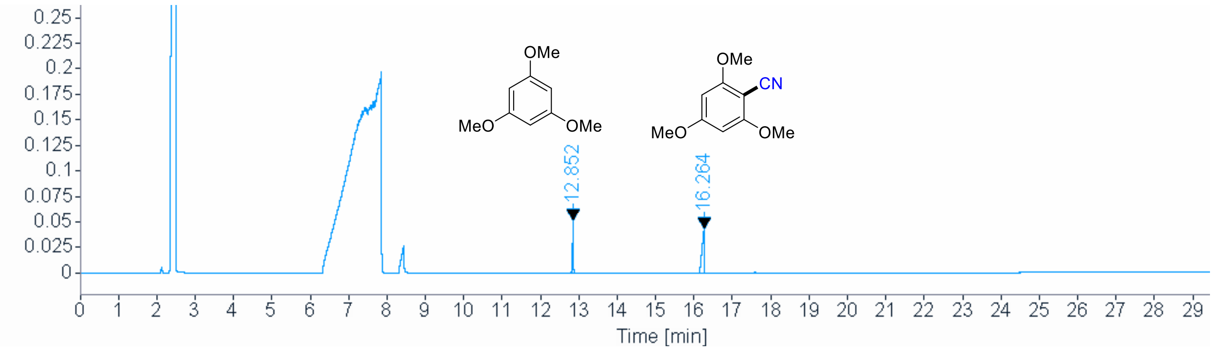

Diethyl (2,4,6-trimethoxyphenyl)phosphonate 2f

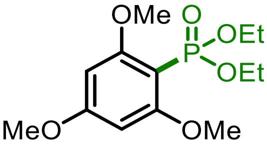

1,3,5-trimethoxybenzene (16.8 mg, 0.1 mmol, 1.0 equiv), photocatalyst **NQH_2-1** and $(NH_4)_2S_2O_8$ (50 mg, 0.22 mmol, 2.2 equiv) were weighted in a 5 ml crimp vial equipped with a magnetic stirring bar, sealed, and backfilled three times with nitrogen. Dry acetonitrile (1 ml) and triethyl phosphite (87 µl, 0.5 mmol, 5.0 equiv) were added via syringe and the mixture degassed by bubbling nitrogen. The mixture was irradiated through the plane bottom with 405 nm at 25°C for 16 hours. The yield of the desired product was determined by ^{31}P NMR of the crude reaction mixture with triphenylphosphine oxide (27.8 mg, 0.1 mmol, 1.0 equiv) as an internal standard. NMR-yield: 5%.
^{31}P NMR (162 MHz, $CDCl_3$) δ [ppm] = 28.9 (internal standard), 16.57 (product).
With 2-SAS as photocatalyst and irradiation at 450 nm under otherwise identical reaction conditions the reaction proceeded with higher yield: NMR-yield: 64%.
^{31}P NMR (162 MHz, $CDCl_3$) δ [ppm] = 28.9 (internal standard), 16.33 (product).
The spectral data was compared to literature values.[20]
GC-MS: product (m/z = 304, calcd = 304).

Diethyl (2,4-dimethoxyphenyl)phosphonate and diethyl (2,6-dimethoxyphenyl)phosphonate) 2g

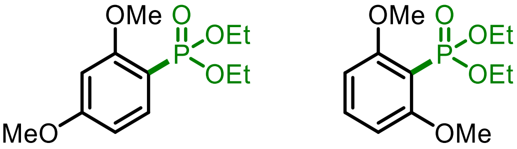

A B

1,3-dimethoxybenzene (13 µl, 0.1 mmol, 1.0 equiv), photocatalyst **NQH_2-1** and $(NH_4)_2S_2O_8$ (50 mg, 0.22 mmol, 2.2 equiv) were weighted in a 5 ml crimp vial equipped with a magnetic stirring bar, sealed, and backfilled three times with nitrogen. Dry acetonitrile (1 ml) and triethyl phosphite (87 µl, 0.5 mmol, 5.0 equiv) were added via syringe and the mixture degassed by bubbling nitrogen. The mixture was irradiated through the plane bottom with 405 nm at 25°C for 16 hours. The yield of the desired product was determined by ^{31}P NMR of the crude reaction mixture with triphenylphosphine oxide (27.8 mg, 0.1 mmol, 1.0 equiv) as an internal standard. NMR-yield: 12% (A), 2% (B).
^{31}P NMR (162 MHz, $CDCl_3$) δ [ppm] = 28.9 (internal standard), 18.16 (A), 15.7 (B).
With 2-SAS as photocatalyst and irradiation at 450 nm under otherwise identical reaction conditions the reaction proceeded with higher yield: NMR-yield: 79% (A), 11% (B).

^{31}P NMR (162 MHz, $CDCl_3$) δ [ppm] = 28.9 (internal standard), 17.81 (A), 16.10 (B).
The spectral data was compared to literature values.[21]
GC-MS: product B (m/z = 274, calcd = 274), product A (m/z = 274, calcd = 274).

Diethyl (2-methoxyphenyl)phosphonate 2h

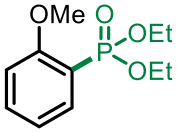

Anisole (11 µl, 0.1 mmol, 1.0 equiv), photocatalyst 2-SAS and $(NH_4)_2S_2O_8$ (50 mg, 0.22 mmol, 2.2 equiv) were weighted in a 5 ml crimp vial equipped with a magnetic stirring bar, sealed, and backfilled three times with nitrogen. Dry acetonitrile (1 ml) and triethyl phosphite (87 µl, 0.5 mmol, 5.0 equiv) were added via syringe and the mixture degassed by bubbling nitrogen. The mixture was irradiated through the plane bottom with 450 nm at 25°C for 16 hours. The yield of the desired product was determined by ^{31}P NMR of the crude reaction mixture with triphenylphosphine oxide (27.8 mg, 0.1 mmol, 1.0 equiv) as an internal standard. NMR-yield: 40% (ortho), 16% (para), 5% (meta).
^{31}P NMR (162 MHz, $CDCl_3$) δ [ppm] = 28.9 (internal standard), 19.86 (para), 18.74 (meta), 17.25 (ortho).
The spectral data was compared to literature values.[22]
GC-MS: ortho (m/z = 244, calcd = 244), meta (m/z = 244, calcd = 244), ortho (m/z = 244, calcd = 244).

1-(2,4,6-trimethoxyphenyl)-1H-pyrazole 2i

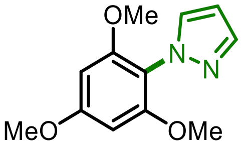

The title compound was synthesized following General Procedure B using 1,3,5-trimethoxybenzene (16.8 mg, 0.10 mmol, 1.0 equiv) and pyrazole (34 mg, 0.5 mmol, 5.0 equiv). An oxygen balloon was used instead of needle air inlets. To isolate the compound the contents of two reactions run in parallel were combined with water and brine in a separatory funnel and extracted with EtOAc (3 × 15 ml). The combined organic phases were washed twice with saturated $NaHCO_3$, dried over $MgSO_4$ and concentrated. The crude was purified by flash column chromatography (PE/EA 50-100%) to yield compound **2i** as white solid (13 mg, 0.06 mmol, 28%).
^{1}H NMR (400 MHz, $CDCl_3$) δ [ppm] = 7.74 (dd, J=1.9, 0.7, 1H), 7.45 (dd, J=2.4, 0.7, 1H), 6.41 (t, J=2.1, 1H), 6.19 (s, 2H), 3.86 (s, 3H), 3.74 (s, 6H).
^{13}C NMR (101 MHz, $CDCl_3$) δ [ppm] = 161.50, 157.48, 140.27, 132.93, 112.64, 105.53, 90.93, 56.30, 55.69.
HRMS (ESI+): exact mass calcd for $C_{12}H_{14}N_2O_3^+$ [M+H$^+$]: m/z = 235.1077, found: 235.1079.
With sodium anthraquinone-2-sulfonate as photocatalyst irradiated at 450 nm under otherwise identical conditions: GC-yield: 22%.
GC-FID retention time [min] = 9.06 (standard), 12.88 (starting material), 16.47 (product).
GC-MS retention time [min] = 11.58 (starting material, m/z = 168, calcd = 168), 15.11 (product, m/z = 234, calcd = 234).

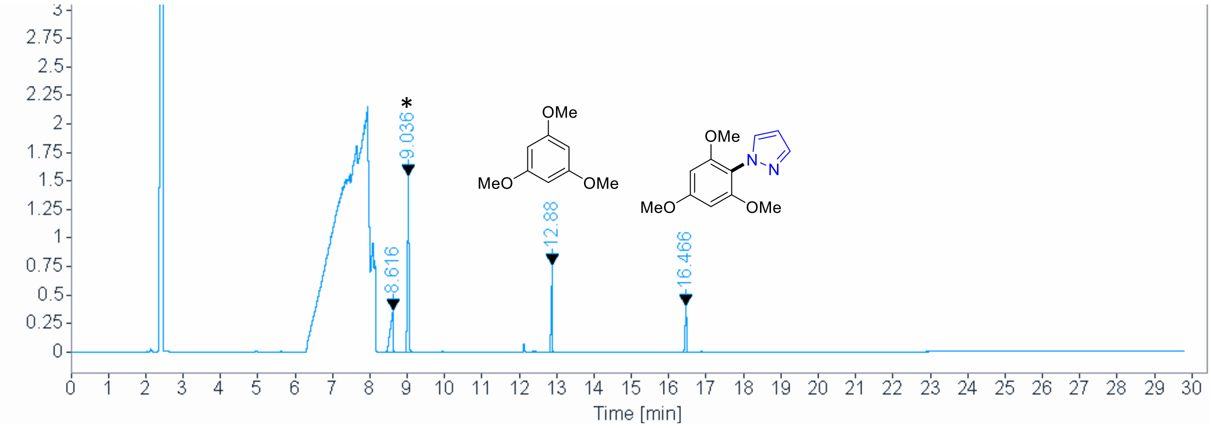

1-(naphthalen-2-yl)-1H-pyrazole 2j

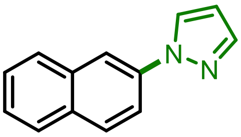

The title compound was synthesized following General Procedure B using naphthalene (12.9, 0.10 mmol, 1.0 equiv) and pyrazole (34 mg, 0.50 mmol, 5.0 equiv). Instead of air inlets, an oxygen balloon was used, and the reaction irradiated for 38 h. To isolate the compound, the contents of two reactions run in parallel were combined with water and brine in a separatory funnel and extracted with ethyl acetate (3 × 15 ml). The organic fractions were combined, dried over $MgSO_4$ and concentrated. The crude was purified by flash column chromatography (PE/EA 5-50%) to afford compound **2j** as white solid (7 mg, 0.036 mmol, 18%).
^{1}H NMR (400 MHz, $CDCl_3$) δ [ppm] = 7.97 – 7.90 (m, 1H), 7.85 (d, *J*=1.9, 0H), 7.83 – 7.78 (m, 1H), 7.59 – 7.47 (m, 3H), 6.55 (t, *J*=2.1, 0H).
^{13}C NMR (101 MHz, $CDCl_3$) δ [ppm] = 140.97, 137.54, 134.45, 131.77, 129.35, 129.05, 128.24, 127.35, 126.77, 125.23, 123.37, 123.33, 106.64.
HRMS (EI+): exact mass calcd for $C_{13}H_{10}N_2^+$ [M^+]: m/z = 194.08385, found: 194.08354.

1-(2,4,6-trimethoxyphenyl)-1H-imidazole 2k

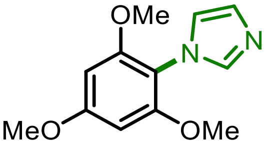

The title compound was synthesized following General Procedure B using 1,3,5-trimethoxybenzene (21.5, 0.13 mmol, 1.0 equiv) and imidazole (35 mg, 0.51 mmol, 4.0 equiv). Instead of air inlets, an oxygen balloon was used, and the reaction irradiated for 45 h. The yield of the desired product was determined by GC-FID. GC-yield: 16% (product).
GC-FID retention time [min] = 12.82 (starting material), 17.66 (product).
To isolate the compound, the contents of three reactions run in parallel with 2-SAS as photocatalyst (irradiation at 450 nm) were combined with water and brine in a separatory funnel and extracted with diethyl ether (3 × 15 ml). The organic fractions were combined, dried over $MgSO_4$ and concentrated. The crude was purified by flash column chromatography (PE/acetone 20-100%) to afford compound **2k** as white solid (19 mg, 0.081 mmol, 27%).
^{1}H NMR (400 MHz, $CDCl_3$) δ [ppm] = 7.49 (s, 1H), 7.14 (t, *J*=1.1, 1H), 6.95 (t, *J*=1.2, 1H), 6.20 (s, 2H), 3.85 (s, 3H), 3.74 (s, 6H).
^{13}C NMR (101 MHz, $CDCl_3$) δ 161.03, 156.27, 139.34, 128.04, 121.59, 108.94, 90.94, 56.10, 55.70.
HRMS (EI+): exact mass calcd for $C_{12}H_{14}N_2O_3^+$ [M^+]: m/z = 234.09989, found: 234.10054.

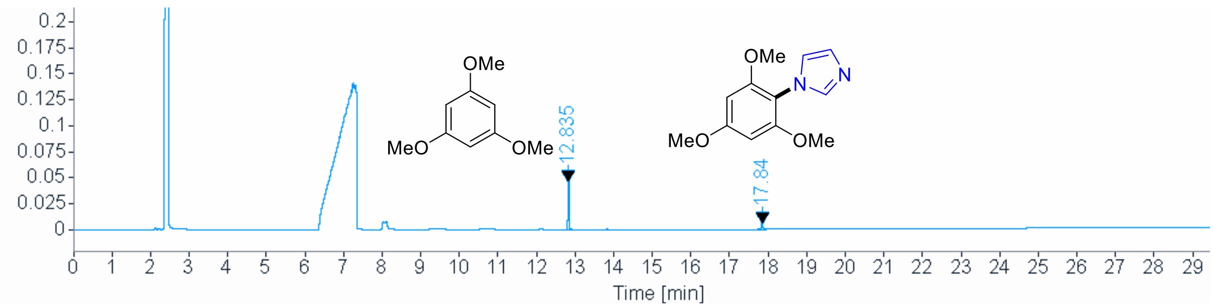
0.2
0.175
0.15
0.125
0.1
0.075
0.05
0.025
0
0 1 2 3 4 5 6 7 8 9 10 11 12 13 14 15 16 17 18 19 20 21 22 23 24 25 26 27 28 29
Time [min]
12.835
17.84
OMe
MeO
OMe
OMe
N
N
MeO
OMe

8.2 Photocatalytic HAT reactions

Acetophenone 3a

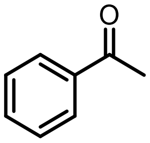

The title compound was synthesized according to general procedure C using 1-phenylethanol (36.2 µl, 0.3 mmol, 1.0 equiv). The yield of the reaction was determined by crude ^{1}H-NMR with 1,1,2,2,-tetrachlorethane (16 µl, 0.15 mmol, 0.5 equiv). NMR-yield: > 95%.
^{1}H NMR (300 MHz, $CDCl_3$) δ [ppm] = 6.00 (standard), 2.64 (product).
To isolate the compound, the contents of three reactions run in parallel were combined, the solvent evaporated, and the crude purified by flash column chromatography (PE/EA 50%) to obtain **3a** as colorless oil (91%, 98 mg, 0.81 mmol).
^{1}H NMR (300 MHz, $CDCl_3$) δ [ppm] = 8.03 – 7.87 (m, 2H), 7.63 – 7.50 (m, 1H), 7.46 (m, 2H), 2.61 (s, 3H).
^{13}C NMR (75 MHz, $CDCl_3$) δ [ppm] = 198.44, 137.17, 133.26, 128.68, 128.43, 26.73.
The compound was also synthesized using ethylbenzene (12.2 µl, 0.1 mmol, 1.0 equiv) as starting material. The yield of the reaction was determined by crude ^{1}H-NMR with 1,1,2,2,-tetrachlorethane (10 µl, 0.1 mmol, 1.0 equiv). NMR-yield: 77%.
^{1}H NMR (400 MHz, $CDCl_3$) δ [ppm] = 5.94 (standard), 2.51 (product).
GC-MS: product (m/z = 120, calcd = 120).
The spectral data was compared to literature values.[23]

Cyclohexanone 3b

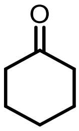

The title compound was synthesized according to general procedure C using cyclohexanol (10.5 µl, 0.1 mmol, 1.0 equiv). The yield of the reaction was determined by crude ^{1}H-NMR with 1,1,2,2,-tetrachlorethane (10 µl, 0.1 mmol, 1.0 equiv). NMR-yield: 71%.
^{1}H NMR (400 MHz, $CDCl_3$) δ [ppm] = 5.95 (standard), 2.30 (t, product).
GC-MS: dehydrogenation product (m/z = 82, calcd = 82), product (m/z = 98, calcd = 98).
The compound was also synthesized using cyclohexane (10.8 µl, 0.1 mmol, 1.0 equiv) as starting material. The yield of the reaction was determined by crude ^{1}H-NMR with 1,1,2,2,-tetrachlorethane (10 µl, 0.1 mmol, 1.0 equiv). NMR-yield: 8%.
Unreacted starting material was confirmed by ^{1}H NMR analysis.
^{1}H NMR (400 MHz, $CDCl_3$) δ [ppm] = 5.94 (standard), 2.23 (product), 1.23 (starting material).
GC-MS: product (m/z = 98, calcd = 98).
The spectral data was compared to literature values.[24]

Cyclopentanone 3c

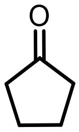

The title compound was synthesized according to general procedure C using cyclopentanol (9.1 µl, 0.1 mmol, 1.0 equiv. The yield of the reaction could not be estimated from crude NMR as the product peaks overlap with the solvent peaks and no more starting material peak could be found in the NMR. Cyclopentanol and cyclopentanone are inseparable by GC-FID but show separation in GC-MS chro-

matography. The yield was estimated from the ratio of product and starting material peaks in the GC-MS chromatogram. GC-MS yield: 91%.

GC-MS (retention time) [min] = 4.587 (starting material, m/z = 86, calcd = 86), 4.666 (product, m/z = 84, calcd = 84).

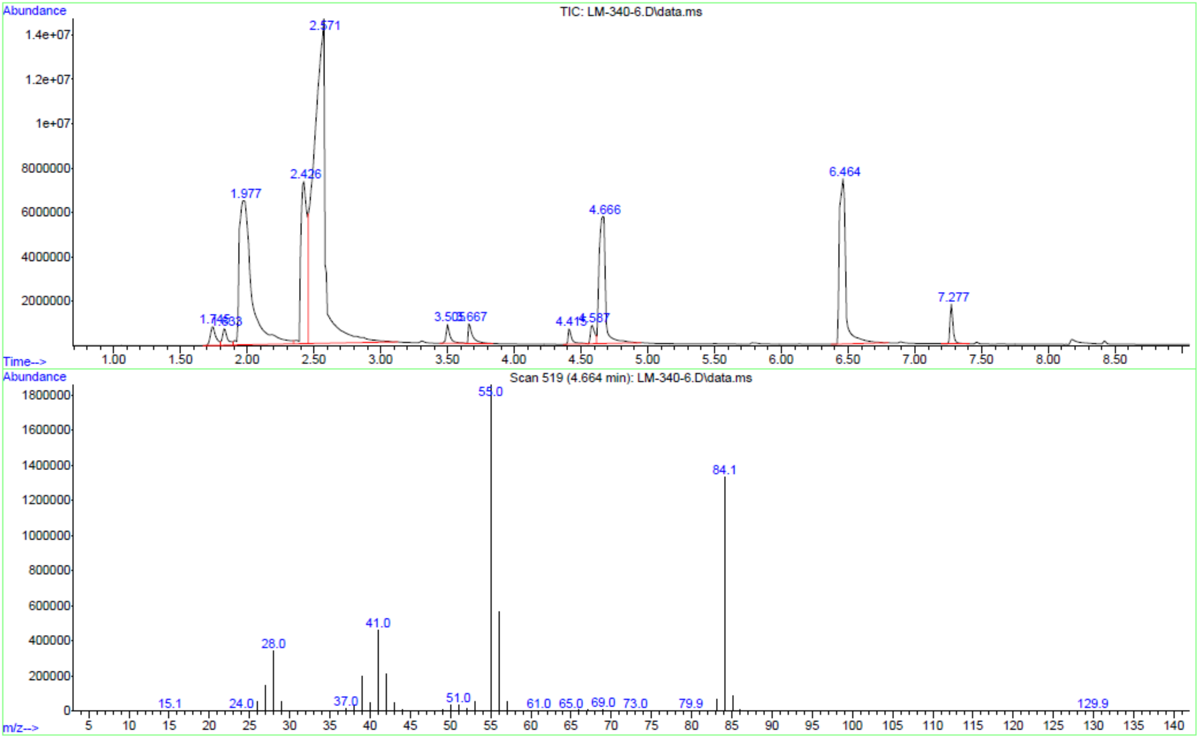

Peak #	Ret Time	Type	Width	Area	Start Time	End Time
1	1.745	BV	0.041	23200584	1.670	1.799
2	1.833	VV	0.039	19234728	1.799	1.895
3	1.977	VV	0.100	419921051	1.895	2.346
4	2.426	VV	0.047	221995816	2.346	2.456
5	2.571	VB	0.077	900773510	2.456	3.125
6	3.505	BV	0.053	17824075	3.442	3.647
7	3.667	VB	0.053	20107131	3.647	3.838
8	4.415	BB	0.033	13767336	4.368	4.559
9	4.587	BV	0.032	17128487	4.559	4.615
10	4.666	VB	0.054	191688979	4.615	4.948
11	6.464	BB	0.049	247243524	6.374	6.800
12	7.277	BB	0.026	27190797	7.197	7.403

Cyclobutanone 3d

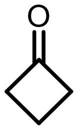

The title compound was synthesized according to general procedure C using cyclobutanol (8.0 µl, 0.1 mmol, 1.0 equiv). The yield of the reaction was determined by crude ^{1}H-NMR with 1,1,2,2,-tetrachlorethane (10 µl, 0.1 mmol, 1.0 equiv) NMR-yield: 52%.

^{1}H NMR (400 MHz, $CDCl_3$) δ [ppm] = 5.95 (standard), 3.05 (t, product).

The spectral data was compared to literature values.[25]

GC-MS: product (m/z = 70, calcd = 70).

Octan-2-one 3e

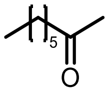

The title compound was synthesized according to general procedure C using octan-2-ol (16.0 µl, 0.1 mmol, 1.0 equiv. The yield of the reaction was determined by crude ^{1}H-NMR with 1,1,2,2,-tetrachlorethane (10 µl, 0.1 mmol, 1.0 equiv). NMR-yield: >95%.

^{1}H NMR (400 MHz, $CDCl_3$) δ [ppm] = 5.95 (standard), 2.40 (t, product).
The spectral data was compared to literature values.[26]
GC-MS: product (m/z = 128, calcd = 128).

4-oxopentanoic acid 3f

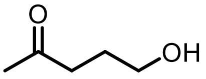

The title compound was synthesized according to general procedure C with pentane-1,4-diol (31.5 µl, 0.3 mmol, 1.0 equiv). The yield of the reaction was determined by crude ^{1}H-NMR with 1,1,2,2,-tetrachlorethane (16 µl, 0.15 mmol, 0.5 equiv). NMR-yield: 11%.
^{1}H NMR (300 MHz, $CDCl_3$) δ [ppm] = 5.95 (standard), 2.69 (t, *J*=6.4, product), 2.55 (t, *J*=6.4, product), 2.13 (s, product).
The spectral data was compared to literature value.[27]

2-Hydroxy-1-indanone 3g

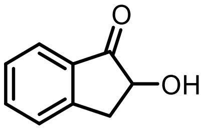

The title compound was synthesized according to general procedure C with 1,2-dihydroxyindan (45 mg, 0.3 mmol, 1.0 equiv). To isolate the compound, the combined contents of three reactions run in parallel were concentrated and the crude purified by flash column chromatography (PE/EA 10-100%) to give **3g** as white solid (39%, 52 mg, 0.35 mmol).
^{1}H NMR (400 MHz, $CDCl_3$) δ [ppm] = 7.85 (d, *J*=7.6, 1H), 7.75 – 7.63 (m, 1H), 7.56 – 7.40 (m, 2H), 4.66 (dd, *J*=7.5, 3.2, 1H), 3.84 (dd, *J*=18.1, 7.6, 1H), 3.43 (dd, *J*=18.1, 3.2, 1H).
The spectral data was compared to literature values.[28]
HRMS (APCI+): exact mass calcd for $C_9H_8O_2$ $[M+H^+]$: m/z = 129.1274; found 129.1271

Benzoic acid 3h

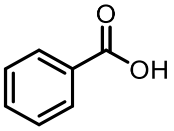

The title compound was synthesized according to general procedure C using benzylalcohol (10.5 µl, 0.1 mmol, 1.0 equiv). The yield of the reaction was determined by crude ^{1}H-NMR with 1,1,2,2,-tetrachlorethane (10 µl, 0.1 mmol, 1.0 equiv). NMR-yield: >95%.
^{1}H NMR (400 MHz, DMSO-d_6) δ [ppm] = 8.00 – 7.91 (m, 2H), 7.71 – 7.56 (m, 1H), 7.50 (t, *J*=7.7, 2H), 6.93 (standard).
GC-MS: product (m/z = 122, calcd =122).
The compound was also synthesized using toluene (10.6 µl, 0.1 mmol, 1.0 equiv) as starting material. The yield of the reaction was determined by crude ^{1}H-NMR with 1,1,2,2,-tetrachlorethane (10 µl, 0.1 mmol, 1.0 equiv). NMR-yield: 31%.
^{1}H NMR (400 MHz, DMSO-d_6) δ [ppm] = 7.95 (m, product), 6.92 (standard).
GC-MS: product (m/z = 122, calcd =122).
The spectral data was compared to literature values.[23]

1-(4-methoxyphenyl)ethan-1-one 3i

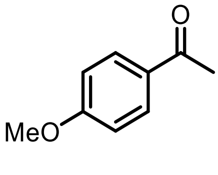

The title compound was synthesized according to general procedure C using 4-ethylanisole (14.2 µl, 0.1 mmol, 1.0 equiv). The yield of the reaction was determined by crude ^{1}H-NMR with 1,1,2,2,-tetrachlorethane (10 µl, 0.1 mmol, 1.0 equiv). NMR-yield: 77%.

^{1}H NMR (400 MHz, $CDCl_3$) δ [ppm] = 7.82 (d, product), 5.93 (standard).

The spectral data was compared to literature values.[29]

GC-MS: product (m/z = 150, calcd =150).

1-acetylnaphthalene 3j

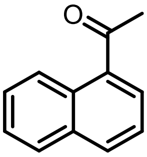

The title compound was synthesized according to general procedure C using 1-ethylnaphthalene (15.5 µl, 0.1 mmol, 1.0 equiv). The yield of the reaction was determined by crude ^{1}H-NMR with 1,1,2,2,-tetrachlorethane (10 µl, 0.1 mmol, 1.0 equiv). NMR-yield: 19%.

^{1}H NMR (400 MHz, $CDCl_3$) δ [ppm] = 8.63 (d, product), 5.93 (standard).

The spectral data was compared to literature values.[30]

GC-MS: product (m/z = 170, calcd =170).

1-tetralone *o*-3k and 2,3-dihydro-1,4-naphthoquinone *o,o*-3k

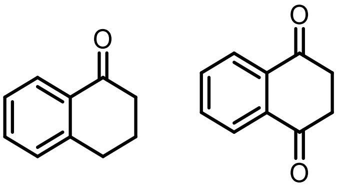

***o*-3k** ***o,o*-3k**

The title compounds were synthesized according to general procedure C using 1,2,3,4-tetrahydronaphthalene (13.6 µl, 0.1 mmol, 1.0 equiv). The yield of the reaction was determined by crude ^{1}H-NMR with 1,1,2,2,-tetrachlorethane (10 µl, 0.1 mmol, 1.0 equiv). NMR-yield: 50% (***o*-3k**), 27% (***o,o*-3k**).

^{1}H NMR (400 MHz, $CDCl_3$) δ [ppm] = 7.35 (t, ***o*-3k**), 5.93 (standard), 2.98 (s, ***o,o*-3k**).

The spectral data was compared to literature values.[5,31]

GC-MS: *o*-3k (m/z = 146, calcd =146), ***o,o*-3k** (m/z = 160, calcd =160).

9-fluorenone 3l

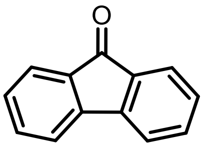

The title compound was synthesized according to general procedure C using fluorene (18.3 mg, 0.11 mmol, 1.0 equiv). The yield of the reaction was determined by crude ^{1}H-NMR with 1,1,2,2,-tetrachlorethane (10 µl, 0.1 mmol, 0.9 equiv). NMR-yield: 53%.

^{1}H NMR (400 MHz, $CDCl_3$) δ [ppm] = 7.53 (d, product), 5.93 (standard).

The spectral data was compared to literature values.[31]

GC-MS: product (m/z = 180, calcd =180).

8.3 Photoreductive transformations

1-methyl-2-(2-(trifluoromethyl)phenyl)-1H-pyrrole 4a

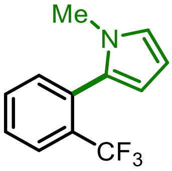

The title compound was synthesized according to general procedure D using 2-chlororbenzotrifluoride (13 µl, 0.10 mmol, 1.0 equiv) and N-methylpyrrole (89 µl, 1.0 mmol, 10 equiv. To isolate the compound, the contents of three vials were transferred with water and brine to an extraction funnel and extracted with diethyl ether (3 × 15 ml). The organic phases were combined, dried with $MgSO_4$, and evaporated. The crude was purified by column chromatography (PE/EA 5:1) to obtain **4a** as yellow oil (55%, 39 mg, 0.17 mmol).

^{1}H NMR (400 MHz, CDCl3) δ = 7.76 (d, *J*=7.2, 1H), 7.57 (t, *J*=7.0, 1H), 7.49 (t, *J*=7.4, 1H), 7.38 (d, *J*=7.5, 1H), 6.71 (t, *J*=2.2, 1H), 6.21 (dd, *J*=3.6, 2.6, 1H), 6.17 – 6.13 (m, 1H), 3.38 (s, 3H).

^{13}C NMR (101 MHz, CDCl3) δ = 133.58, 131.25, 129.61, 128.30, 126.36, 126.31, 126.26, 126.21, 122.38, 110.31, 110.29, 110.27, 107.40, 77.48, 76.84, 34.38.

^{19}F NMR (377 MHz, $CDCl_3$) δ [ppm] = -59.81.

The spectral data was compared to literature values.[32]

HRMS (EI+): exact mass calcd for $C_{12}H_{10}F_3N^+$ [M^+]: m/z = 225.07599, found: 225.07643.

1-phenyl-2-(2-(trifluoromethyl)phenyl)-1H-pyrrole 4b

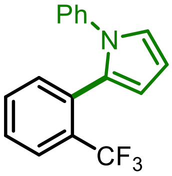

The title compound was synthesized according to general procedure D using 2-chlorobenzotrifluoride (13 µl, 0.10 mmol, 1.0 equiv) and N-phenylpyrrole (143 mg, 1.0 mmol, 10 equiv). The yield of the desired product was determined by ^{19}F NMR of the crude reaction mixture with fluorobenzene (10 µl, 0.11 mmol) as an internal standard. NMR-yield: 39%.

^{19}F NMR (377 MHz, $CDCl_3$) δ [ppm] = -57.96 (product), -62.57 (starting material), -113.15 (standard).

To isolate the compound, the combined contents of three reactions run in parallel were transferred with water and brine to an extraction funnel, extracted with DCM (5 × 15 ml), the combined organic phases dried over $MgSO_4$, filtered, and concentrated. The crude was purified by flash column chromatography (PE/EA, 0-5%) to give **4b** as red oil (10%, 17 mg, 0.06 mmol).

^{1}H NMR (400 MHz, $CDCl_3$) δ [ppm]= 7.70 (dd, *J*=7.6, 1.8, 1H), 7.37 – 7.27 (m, 2H), 7.26 – 7.21 (m, 2H), 7.20 – 7.15 (m, 1H), 7.10 – 7.05 (m, 2H), 7.05 – 6.99 (m, 2H), 6.44 – 6.41 (m, 1H), 6.38 (dd, *J*=3.6, 2.8, 1H).

^{13}C NMR (101 MHz, $CDCl_3$) δ [ppm] = 140.24, 133.80, 130.92, 129.31, 129.03, 127.45, 126.51, 126.42, 126.37, 125.41, 123.49, 113.04 (q, *J*=3.0), 109.01.

^{19}F NMR (377 MHz, $CDCl_3$) δ [ppm] = -58.57.

HRMS (EI+): exact mass calcd for $C_{17}H_{12}F_3N^+$ [M^+]: m/z = 287.09164, found: 287.09152.

1-phenyl-2-(4-(trifluoromethyl)phenyl)-1H-pyrrole 4c

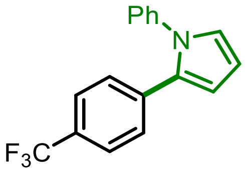

The title compound was synthesized according to general procedure D using 4-chlorobenzotrifluoride (13 µl, 0.10 mmol, 1.0 equiv) and N-phenylpyrrole (143 mg, 1.0 mmol, 10 equiv). The yield of the desired product was determined by ^{19}F NMR of the crude reaction mixture with fluorobenzene (9.5 µl, 0.10 mmol) as an internal standard. NMR-yield: 15%.
^{19}F NMR (377 MHz, $CDCl_3$) δ [ppm] = -62.31 (product), -62.51 (starting material), -113.15 (standard).
The spectral data was compared to literature values.[33]

(2-(2-(trifluoromethyl)phenyl)ethene-1,1-diyl)dibenzene 4d

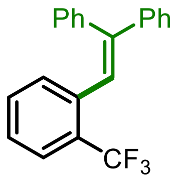

The title compound was synthesized according to general procedure D using 2-chlorobenzotrifluoride (13 µl, 0.10 mmol, 1.0 equiv) and 1,1-diphenylethylene (176 µl, 1.0 mmol, 10 equiv). The yield of the desired product was determined by ^{19}F NMR of the crude reaction mixture with fluorobenzene (10 µl, 0.11 mmol) as an internal standard. NMR-yield: 18%.
^{19}F NMR (377 MHz, $CDCl_3$) δ [ppm] = -60.63 (product), -62.58 (starting material), -113.15 (standard).
To isolate the compound, the combined contents of four reactions run in parallel were transferred with water and brine to an extraction funnel, extracted with DCM (5 × 15 ml), the combined organic phases dried over $MgSO_4$, filtered, and concentrated. The crude was purified by flash column chromatography (PE, 100%) to give **4d** as colorless oil (16%, 21 mg, 0.07 mmol).
^{1}H NMR (400 MHz, $CDCl_3$) δ [ppm] = 7.63 (d, *J*=7.7, 1H), 7.36 – 7.32 (m, 5H), 7.24 – 7.18 (m, 5H), 7.15 (d, *J*=1.4, 1H), 7.13 – 7.08 (m, 2H), 6.95 (d, *J*=7.7, 1H).
^{13}C NMR (101 MHz, $CDCl_3$) δ [ppm] = 145.56, 142.90, 139.79, 137.03 (q, *J*=1.7), 132.10, 131.09, 130.97, 129.13 (q, *J*=29.5), 128.40, 128.31, 128.29, 128.10, 127.55, 126.61, 125.66 (q, *J*=5.5), 124.52.
^{19}F NMR (377 MHz, $CDCl_3$) δ [ppm] = -61.21.
HRMS (EI+): exact mass calcd for $C_{21}H_{15}F_3^+$ [M$^+$]: m/z = 324.11204, found: 324.11231.

1-methyl-2-phenyl-1H-pyrrole 4e

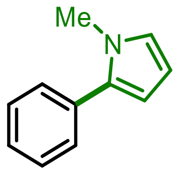

The title compound was synthesized according to general procedure D using chlorobenzene (10 µl, 0.10 mmol, 1.0 equiv) and N-methylpyrrole (89 µl, 1.0 mmol, 10 equiv). To isolate the compound, the contents of four vials were transferred with water and brine to an extraction funnel and extracted with diethyl ether (3 × 15 ml). The organic phases were combined, dried with $MgSO_4$ and evaporated. The crude was purified by column chromatography (PE/EA 5:1) to obtain **4e** as yellow oil (21%, 13 mg, 0.08 mmol).
^{1}H NMR (400 MHz, $CDCl_3$) δ = 7.45 – 7.38 (m, 1H), 6.72 (s, 1H), 6.26 – 6.18 (m, 2H), 3.67 (s, 3H).
^{13}C NMR (101 MHz, $CDCl_3$) δ 134.76, 133.50, 128.80, 128.48, 126.87, 123.77, 108.78, 107.90, 77.48, 77.36, 77.16, 76.84, 35.20.
The spectral data was compared to literature values.[34]
HRMS (EI+): exact mass calcd for $C_{11}H_{11}N^+$ [M$^+$]: m/z = 157.08860, found: 157.08887.

2-(4-methoxyphenyl)-1-methyl-1H-pyrrole 4f

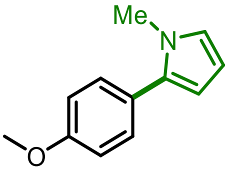

The title compound was synthesized according to general procedure D using 1-chloro-4-methoxybenzene (13 µl, 0.10 mmol, 1.0 equiv) and N-methylpyrrole (89 µl, 1.0 mmol, 10 equiv). The yield was determined by GC-FID with 4-methylanisole as internal standard (12.6 µl, 0.10 mmol, 1.0 equiv). GC-FID yield: 16%

To isolate the compound, the contents of four vials were transferred with water and brine to an extraction funnel and extracted with diethyl ether (3 × 15 ml). The organic phases were combined, dried with $MgSO_4$ and evaporated. The crude was purified by column chromatography (PE/EA 5:1) to obtain **4f** as yellow oil (7%, 5 mg, 0.03 mmol).

^{1}H NMR (400 MHz, CDCl3) δ = 7.36 – 7.29 (m, 2H), 6.99 – 6.90 (m, 2H), 6.69 (dd, *J*=2.7, 1.8, 1H), 6.19 (dd, *J*=3.5, 2.6, 1H), 6.15 (dd, *J*=3.6, 1.8, 1H), 3.84 (s, 3H), 3.63 (s, 3H).

^{13}C NMR (101 MHz, CDCl3) δ 158.79, 134.52, 130.18, 126.12, 123.11, 113.94, 108.13, 107.70, 77.48, 77.36, 77.16, 76.84, 55.45, 35.02.

The spectral data was compared to literature values.[34,35]

HRMS (EI+): exact mass calcd for $C_{12}H_{13}NO^+$ [M^+]: m/z = 187.09917, found: 187.09899.

The reaction was also carried out with 1-bromo-4-methoxybenzene as starting material (12.6 µL, 0.10 mmol, 1.0 equiv). The yield was determined by GC-FID with 4-methylanisole as internal standard (12.6 µl, 0.10 mmol, 1.0 equiv). GC-FID yield: 17%

GC-FID retention time [min] = 9.88 (standard), 10.92 (starting material), 14.87 (product).

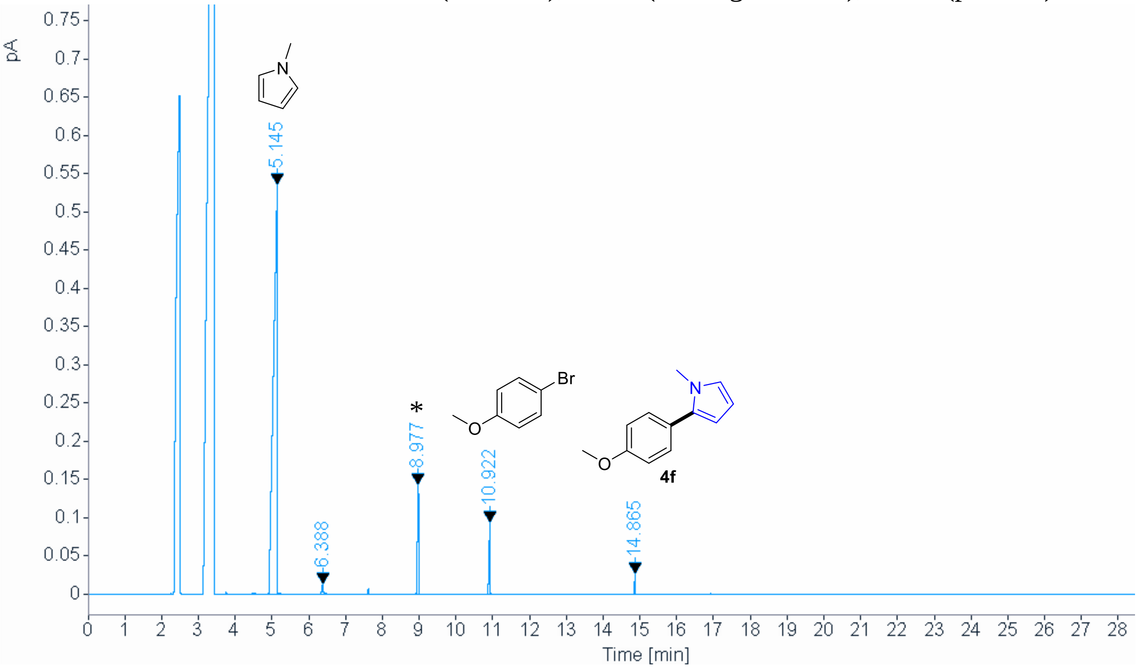

2-(5-chloro-2-(trifluoromethyl)phenyl)-1-methyl-1H-pyrrole and regioisomers 4g

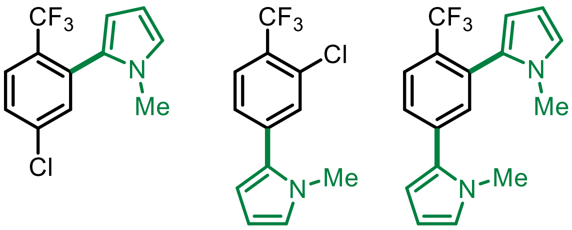

The title compound was synthesized according to general procedure D using 2,4-dichloro-1-(trifluoromethyl)benzene (16 µl, 0.10 mmol, 1.0 equiv) and N-methylpyrrole (89 µl, 1.0 mmol, 10 equiv). The yield of the desired product was determined by ^{19}F NMR of the crude reaction mixture with fluorobenzene (10 µl, 0.10 mmol, 1.0 equiv) as an internal standard. NMR-yield: 47%.
^{19}F NMR (376 MHz, $CDCl_3$) δ [ppm] = -58.88 (disubstituted product, 4%), -59.04 (Ortho, 37%), -62.09 (para, 6%), -62.42 (starting material), -113.15 (standard).
To isolate the compound, the combined contents of three reactions run in parallel were transferred to a separatory funnel with water and brine and extracted with ethyl acetate (3 × 15 ml). The combined organic phases were dried over $MgSO_4$, filtered and concentrated. The crude was purified by column chromatography (PE : EA, 0 - 10%) to yield the ortho-substituted compound **4g-o** as colorless oil (9 mg, 0.03 mmol, 12%), the para-substituted compound **4g-p** as colorless oil (3 mg, 0.01 mmol, 4%), and the ortho-/para-disubstituted compound **4g-o/p** as colorless oil (25 mg, 0.05 mmol, 13%).
4g-o
^{1}H NMR (400 MHz, $CDCl_3$) δ [ppm] = 7.69 (d, *J*=8.5, 1H), 7.47 (ddd, *J*=8.4, 2.1, 0.9, 1H), 7.39 (d, *J*=2.2, 1H), 6.72 (dd, *J*=2.7, 1.8, 1H), 6.20 (dd, *J*=3.6, 2.7, 1H), 6.18 – 6.13 (m, 1H), 3.40 (s, 3H).
^{19}F NMR (376 MHz, $CDCl_3$) δ [ppm] = -59.65.
^{13}C NMR (101 MHz, $CDCl_3$) δ [ppm] = 137.43, 134.51 (d, *J*=1.9), 133.54, 129.40 (d, *J*=30.0), 128.45, 128.12, 127.74 (q, *J*=5.2), 123.64 (d, *J*=273.6), 122.94, 110.84 (d, *J*=2.0), 107.67, 34.45.
HRMS (EI+): exact mass calcd for $C_{12}H_9ClF_3N^+$ [M^+]: m/z = 259.03701, found: 259.03643.
4g-p
^{1}H NMR (400 MHz, $CDCl_3$) δ [ppm] = 7.69 (d, *J*=8.2, 1H), 7.54 (d, *J*=0.7, 1H), 7.38 (ddd, *J*=8.2, 1.8, 0.9, 1H), 6.77 (dd, *J*=2.6, 1.8, 1H), 6.34 (dd, *J*=3.7, 1.8, 1H), 6.22 (dd, *J*=3.7, 2.7, 1H), 3.71 (s, 3H).
^{13}C NMR (101 MHz, $CDCl_3$) δ [ppm] = 138.32, 132.46 (d, *J*=2.1), 131.74, 130.61, 127.72 (q, *J*=5.3), 126.09 (d, *J*=31.6), 126.02, 125.84, 123.16 (d, *J*=272.7), 110.89, 108.66, 35.51.
^{19}F NMR (376 MHz, $CDCl_3$) δ [ppm] = -62.70.
HRMS (EI+): exact mass calcd for $C_{12}H_9ClF_3N^+$ [M^+]: m/z = 259.03701, found: 259.03683.
4g-o/p
^{1}H NMR (400 MHz, $CDCl_3$) δ [ppm] = 7.76 (d, *J*=8.2, 1H), 7.52 (dd, *J*=8.2, 1.9, 1H), 7.42 (d, *J*=1.9, 1H), 6.76 (dd, *J*=2.6, 1.9, 1H), 6.73 (dd, *J*=2.7, 1.8, 1H), 6.33 (dd, *J*=3.7, 1.8, 1H), 6.24 – 6.20 (m, 2H), 6.18 (dd, *J*=3.7, 1.7, 1H), 3.72 (s, 3H), 3.42 (s, 3H).
^{13}C NMR (101 MHz, $CDCl_3$) δ [ppm] = 136.31, 132.82, 132.72, 129.51, 128.79, 127.54, 126.55 (d, *J*=5.2), 125.40, 122.47, 110.41, 110.38 (d, *J*=1.8), 108.51, 107.47, 35.54, 34.46.
^{19}F NMR (376 MHz, $CDCl_3$) δ [ppm] = -59.52.
HRMS (EI+): exact mass calcd for $C_{17}H_{15}F_3N_2^+$ [M^+]: m/z = 304.11818, found: 304.11801.

4,4,5,5-tetramethyl-2-(2-(trifluoromethyl)phenyl)-1,3,2-dioxaborolane 4h

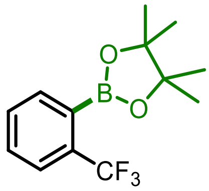

The title compound was synthesized according to general procedure D using 2-chlorobenzotrifluoride (13 µl, 0.10 mmol, 1.0 equiv) and bis(pinacolato)diboron (51 mg, 0,2 mmol, 2.0 equiv). The yield of the desired product was determined by ^{19}F NMR of the crude reaction mixture with fluorobenzene (10 µl, 0.10 mmol, 1.0 equiv) as an internal standard. NMR-yield: 37%.
The reaction also proceeded without photocatalyst in reduced yield. NMR-yield: 28%.
^{19}F NMR (377 MHz, $CDCl_3$) δ [ppm] = -62.56 (starting material), -62.62 (product), -113.15 (standard).
The spectral data was compared to literature values.[36]
GC-MS: product (m/z = 272, calcd = 272).

Dimethyl(2-(trifluoromethyl)phenyl)phosphonate (4i)

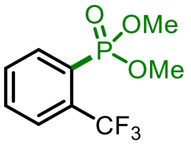

The title compound was synthesized according to general procedure D using 2-chlorobenzotrifluoride (13 µl, 0.10 mmol, 1.0 equiv) and trimethyl phosphite (118 µl, 1.0 mmol, 10 equiv). The yield of the desired product was determined by ^{19}F NMR of the crude reaction mixture with fluorobenzene (28 µl, 0.30 mmol) as an internal standard. NMR-yield: 51%.

^{19}F NMR (377 MHz, $CDCl_3$) δ [ppm] = -59.29 (product), -62.57 (starting material), -113.15 (standard).

The spectral data was compared to literature values.[37]

Dimethyl(4-(trifluoromethyl)phenyl)phosphonate 4j

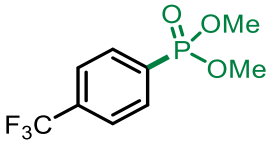

Synthesized according to general procedure D using 4-chlorobenzotrifluoride (14 µl, 0.10 mmol, 1.0 equiv) and trimethyl phosphite (118 µl, 1.0 mmol, 10 equiv). The yield of the desired product was determined by ^{19}F NMR of the crude reaction mixture with fluorobenzene (28 µl, 0.30 mmol) as an internal standard NMR-yield: 16%.

^{19}F NMR (377 MHz, $CDCl_3$) δ [ppm] = -62.51 (starting material), -63.24 (product), -113.15 (standard).

The spectral data was compared to literature values.[37]

Diethyl (2-(trifluoromethyl)phenyl)phosphonate 4k

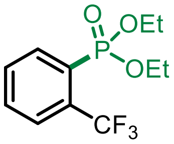

The title compound was synthesized according to general procedure D using 2-chlorobenzotrifluoride (13 µl, 0.10 mmol, 1.0 equiv) and triethyl phosphite (87 µl, 0.5 mmol, 5 equiv). The yield of the desired product was determined by ^{19}F NMR of the crude reaction mixture with fluorobenzene (10 µl, 0.11 mmol) as an internal standard. NMR-yield: 57%.

^{19}F NMR (377 MHz, $CDCl_3$) δ [ppm] = -58.77 (product), -62.57 (starting material), -113.15 (standard).

The spectral data was compared to literature values.[38]

The reaction also proceeds to a lower extent without added photocatalyst. NMR-yield: 43%

^{19}F NMR (377 MHz, $CDCl_3$) δ [ppm] = -58.76 (product), -62.56 (starting material), -113.15 (standard).

Diethyl (4-(trifluoromethyl)phenyl)phosphonate 4l

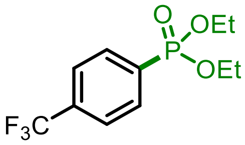

The title compound was synthesized according to general procedure D using 4-chlorobenzotrifluoride (13 µl, 0.10 mmol, 1.0 equiv) and triethyl phosphite (173 µl, 1.0 mmol, 10 equiv). The yield of the desired product was determined by ^{19}F NMR of the crude reaction mixture with fluorobenzene (10 µl, 0.11 mmol) as an internal standard. NMR-yield: 18%.

^{19}F NMR (377 MHz, $CDCl_3$) δ [ppm] = -62.50 (starting material), -63.19 (product), -113.15 (standard).
The spectral data was compared to literature values.[39]

Diisopropyl (2-(trifluoromethyl)phenyl)phosphonate 4m

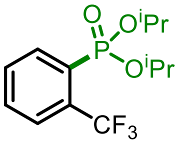

The title compound was synthesized according to general procedure D using 2-chlorobenzotrifluoride (13 µl, 0.10 mmol, 1.0 equiv) and triisopropyl phosphite (247 µl, 1.0 mmol, 10 equiv). The yield of the desired product was determined by ^{19}F NMR of the crude reaction mixture with fluorobenzene (10 µl, 0.11 mmol) as an internal standard. NMR-yield: 49%.
^{19}F NMR (377 MHz, $CDCl_3$) δ [ppm] = -58.04 (product), -62.56 (starting material), -113.15 (standard).
To isolate the compound, the combined contents of three reactions run in parallel were transferred to a separatory funnel with water and brine and extracted with DCM (3 × 15 ml). The combined organic phases were dried over $MgSO_4$, filtered and concentrated. The crude was purified by column chromatography (DCM/MeOH/PE/EA, 9/1/5/5) to yield compound **4m** as yellow oil (37 mg, 0.12 mmol, 40%).
^{1}H NMR (400 MHz, $CDCl_3$) δ [ppm] = 8.33 – 8.21 (m, 1H), 7.83 – 7.74 (m, 1H), 7.67 – 7.57 (m, 2H), 4.79 (h, 6.1, 2H), 1.38 (d, *J*=6.2, 6H), 1.24 (d, *J*=6.2, 6H).
^{13}C NMR (101 MHz, $CDCl_3$) δ [ppm] = 135.97 (d, *J*=7.8), 132.15 (d, *J*=2.8), 131.36 (d, *J*=13.3), 127.50 (q, *J*=5.7), 71.77 (d, *J*=6.3), 24.05 (d, *J*=4.5), 23.84 (d, *J*=4.8).
^{19}F NMR (377 MHz, $CDCl_3$) δ [ppm] = -58.62.
^{31}P NMR (162 MHz, $CDCl_3$) δ [ppm] = 13.02.
HRMS (ESI+): exact mass calcd for $C_{13}H_{18}F_3O_3PNa^+$ [M+Na$^+$]: m/z = 333.0838, found: 333.0837.

Diisopropyl (4-(trifluoromethyl)phenyl)phosphonate 4n

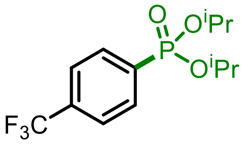

The title compound was synthesized according to general procedure D using 4-chlorobenzotrifluoride (13 µl, 0.10 mmol, 1.0 equiv) and triisopropyl phosphite (247 µl, 1.0 mmol, 10 equiv). The yield of the desired product was determined by ^{19}F NMR of the crude reaction mixture with fluorobenzene (10 µl, 0.11 mmol) as an internal standard. NMR-yield: 20%.
^{19}F NMR (377 MHz, $CDCl_3$) δ [ppm] = -62.50 (starting material), -63.12 (product), -113.15 (standard).
The spectral data was compared to literature values.[40]

Diphenyl (2-(trifluoromethyl)phenyl)phosphonate 4o

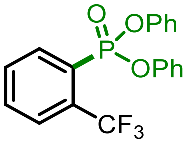

The title compound was synthesized according to general procedure D using 2-chlorobenzotrifluoride (13 µl, 0.10 mmol, 1.0 equiv) and triphenyl phosphite (247 µl, 1.0 mmol, 10 equiv). The yield of the desired product was determined by ^{19}F NMR of the crude reaction mixture with fluorobenzene (28 µl, 0.30 mmol) as an internal standard. NMR-yield: 27%.

^{19}F NMR (377 MHz, $CDCl_3$) δ [ppm] = -58.29 (product), -62.58 (starting material), -113.15 (standard).
To isolate the compound, the combined contents of four reactions run in parallel were transferred to a separatory funnel with water and brine and extracted with DCM (3 × 15 ml). The combined organic phases were dried over $MgSO_4$, filtered and concentrated. The crude was purified by column chromatography (PE : EA, 5:1) to yield compound **4o** as yellow oil (27 mg, 0.07 mmol, 18%).
^{1}H NMR (400 MHz, $CDCl_3$) δ [ppm] = 8.42 (dd, *J*=15.8, 7.5, 1H), 7.92 (t, *J*=7.0, 1H), 7.79 – 7.65 (m, 2H), 7.30 (t, *J*=7.9, 4H), 7.19 (s, 2H), 7.15 (t, *J*=7.3, 2H).
^{13}C NMR (101 MHz, $CDCl_3$) δ [ppm] = 150.46 (d, *J*=8.4), 136.91 (d, *J*=8.3), 133.41 (d, *J*=2.9), 132.89 (d, *J*=7.1), 132.56 (d, *J*=6.9), 131.74 (d, *J*=14.5), 129.78, 127.92 (dd, *J*=12.0, 5.8), 125.38 (d, *J*=1.4), 120.64 (d, *J*=4.7).
^{19}F NMR (377 MHz, $CDCl_3$) δ [ppm] = -58.83.
^{31}P NMR (162 MHz, $CDCl_3$) δ [ppm] = 8.06.
HRMS (ESI+): exact mass calcd for $C_{19}H_{14}F_3O_3PNa^+$ [M+Na$^+$]: m/z = 401.0525, found: 401.0524.

Diethyl (4-methoxyphenyl)phosphonate 4p

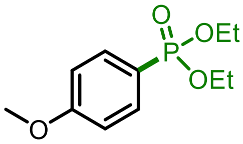

The title compound was synthesized according to general procedure D using 4-bromoanisole (12.6 µl, 0.10 mmol, 1.0 equiv) and triethyl phosphite (172 µl, 1.0 mmol, 10 equiv). The yield of the desired product was determined by ^{31}P NMR of the crude reaction mixture with triphenylphosphine oxide (27.8 mg, 0.10 mmol, 1.0 equiv) as an internal standard. NMR-yield: 36%.
^{31}P NMR (162 MHz, $CDCl_3$) δ [ppm] = 28.9 (standard), 19.60 (product).
The spectral data was compared to literature values.[22]
GC-MS: product (m/z = 244, calcd = 244).

Tetraisopropyl (4-(trifluoromethyl)-1,3-phenylene)bis(phosphonate) and Diisopropyl (5-chloro-2-(trifluoromethyl)phenyl)phosphonate 4q

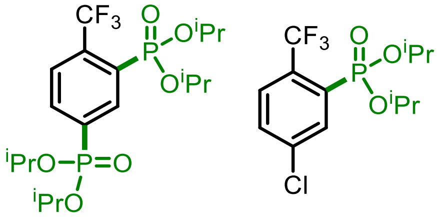

The title compound was synthesized according to general procedure D using 2,4-dichloro-1-(trifluoromethyl)benzene (16 µl, 0.10 mmol, 1.0 equiv) and triisopropyl phosphite (247 µl, 1.0 mmol, 10 equiv). The yield of the desired product was determined by ^{19}F NMR of the crude reaction mixture with fluorobenzene (10 µl, 0.10 mmol, 1.0 equiv) as an internal standard. NMR-yield: 13% ortho-product, 34% disubstituted product.
^{19}F NMR (376 MHz, $CDCl_3$) δ [ppm] = -57.94 (ortho-substituted product), -58.50 (disubstituted product), -62.43 (starting material) , -113.15 (standard).
To isolate the compound, the combined contents of four reactions run in parallel were transferred to a separatory funnel with water and brine and extracted with DCM (3 × 15 ml). The combined organic phases were dried over $MgSO_4$, filtered and concentrated. The crude was purified by column chromatography (PE : acetone, 10 - 40%) to yield the ortho-substituted compound **4q-o** as colorless oil (5 mg, 0.01 mmol, 4%) and the ortho-/para-disubstituted compound **4q-o/p** as colorless oil (25 mg, 0.05 mmol, 13%).
4q-o

^{1}H NMR (400 MHz, $CDCl_3$) δ [ppm] = 8.26 (dd, *J*=15.4, 2.3, 1H), 7.72 (dd, *J*=8.5, 6.0, 1H), 7.59 (m, 1H).
^{13}C NMR (101 MHz, $CDCl_3$) δ [ppm] = 138.16 (d, *J*=19.3), 136.00 (d, *J*=8.6), 132.07 (d, *J*=2.8), 129.08 (dd, *J*=12.4, 5.9), 72.27 (d, *J*=6.2), 23.93 (dd, *J*=19.1, 4.6).
^{19}F NMR (377 MHz, $CDCl_3$) δ [ppm] = -58.49.
^{31}P NMR (162 MHz, $CDCl_3$) δ [ppm] = 10.95.
HRMS (ESI+): exact mass calcd for $C_{13}H_{17}ClF_3O_3PNa^+$ [M+Na$^+$]: m/z = 367.0448, found: 367.0448.
4q-o/p
^{1}H NMR (400 MHz, $CDCl_3$) δ [ppm] = 8.60 (t, *J*=13.9, 1H), 8.08 (dd, *J*=13.0, 8.0, 1H), 7.86 (ddd, *J*=8.3, 5.0, 3.5, 1H), 4.86 – 4.77 (m, 2H), 4.77 – 4.67 (m, 2H), 1.38 (dd, *J*=6.1, 1.5, 13H), 1.26 (dd, *J*=9.3, 6.2, 12H).
^{13}C NMR (101 MHz, $CDCl_3$) δ [ppm] = 138.40 (dd, *J*=11.3, 8.4), 135.37 (dd, *J*=10.2, 2.6), 133.48 (d, *J*=12.6), 130.41 (d, *J*=11.7), 128.55 (d, *J*=13.2), 127.74 – 127.05 (m), 123.08 (dd, *J*=274.3, 5.1), 72.15 (d, *J*=6.3), 71.78 (d, *J*=5.9), 24.12 (dd, *J*=10.9, 4.3), 23.89 (dd, *J*=19.8, 4.9).
^{19}F NMR (377 MHz, $CDCl_3$) δ [ppm] = -59.07.
^{31}P NMR (162 MHz, $CDCl_3$) δ [ppm] = 13.43 (d, *J*=5.2, 1P), 11.56 (d, *J*=5.0, 1P).
HRMS (ESI+): exact mass calcd for $C_{19}H_{31}F_3O_6P_2Na^+$ [M+Na$^+$]: m/z = 497.1440, found: 497.1443.

2-(benzo[b]thiophen-2-yl)propan-2-ol 5a

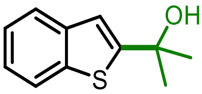

The title compound was synthesized according to general procedure E, using benzothiophene (13.4 mg, 0.1 mmol, 1.0 equiv) and acetone (74 µl, 1.0 mmol, 10 equiv.). To isolate the compound, the combined contents of four reaction run in parallel were transferred with water and brine to a separatory funnel, extracted with ethyl acetate (3 × 15 ml), the combined organic phases dried over $MgSO_4$, filtered, and concentrated. The crude was purified by flash column chromatography (PE/EA 0-50%) to obtain **5a** as light brown crystals (81%, 62 mg, 0.32 mmol). The compound was found to eliminate water in $CDCl_3$ over time to give 2-(prop-1-en-2-yl)benzo[b]thiophene. Therefor the NMR analysis was conducted in DCM-d_2.
^{1}H NMR (400 MHz, CD_2Cl_2) δ = 7.80 (m, 1H), 7.74 – 7.69 (m, 1H), 7.39 – 7.24 (m, 2H), 7.16 (s, 1H), 1.70 (s, 7H), 2.17 (s, 1H).
^{13}C NMR (101 MHz, CD_2Cl_2) δ = 155.74, 140.35, 139.60, 124.59, 124.31, 123.68, 122.62, 118.64, 71.91, 32.22.
The spectral data was compared to literature values.[41]
HRMS (EI+): exact mass calcd for $C_{11}H_{12}OS^+$ [M$^+$]: m/z = 192.06034, found 192.06088.

1-(benzo[b]thiophen-2-yl)cyclohexan-1-ol 5b

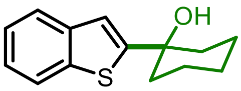

The title compound was synthesized according to general procedure E, using benzothiophene (13.4 mg, 0.1 mmol, 1.0 equiv) and cyclohexanone (100 µl, 1.0 mmol, 10 equiv.). To isolate the compound, the combined contents of four reaction run in parallel were transferred with water and brine to a separatory funnel, extracted with ethyl acetate (3 × 15 ml), the combined organic phases dried over $MgSO_4$, filtered, and concentrated. The crude was purified by flash column chromatography (PE/EA 0-50%) to yield **5b** as off-white crystals (40%, 37 mg, 0.16 mmol).
^{1}H NMR (400 MHz, $CDCl_3$) δ = 7.84 – 7.77 (m, 1H), 7.74 – 7.67 (m, 1H), 7.40 – 7.27 (m, 2H), 7.20 (s, 1H), 2.07 – 1.92 (m, 5H), 1.86 – 1.74 (m, 2H), 1.74 – 1.58 (m, 2H).
^{13}C NMR (101 MHz, $CDCl_3$) δ = 155.34, 140.04, 139.21, 127.24, 124.32, 124.12, 123.53, 122.50, 118.63, 72.62, 39.87, 25.54, 22.44.
The spectral data was compared to literature values.[42]

HRMS (EI+): exact mass calcd for $C_{14}H_{16}OS$ [M^+]: m/z = 232.09164; found 232.09143.

1-(benzo[b]thiophen-2-yl)cyclobutan-1-ol 5c

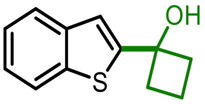

The title compound was synthesized according to general procedure E, using benzothiophene (13.4 mg, 0.1 mmol, 1.0 equiv) and cyclobutanone (74 µl, 1.0 mmol, 10 equiv.). To isolate the compound, the combined contents of four reaction run in parallel were transferred with water and brine to a separatory funnel, extracted with ethyl acetate (3 × 15 ml), the combined organic phases dried over $MgSO_4$, filtered, and concentrated. The crude was purified by flash column chromatography (PE/EA 0-50%) to yield **5c** as of-white solid (14%, 11 mg, 0.06 mmol). Unreacted starting material was recovered and confirmed by GC-FID analysis.

^{1}H NMR (400 MHz, $CDCl_3$) δ [ppm] = 7.81 (d, *J*=8.3, 1H), 7.73 (d, *J*=6.8, 1H), 7.39 – 7.28 (m, 3H), 7.27 (s, 1H), 2.69 – 2.56 (m, 2H), 2.55 – 2.42 (m, 2H), 2.08 – 1.91 (m, 1H), 1.90 – 1.72 (m, 1H).

^{13}C NMR (101 MHz, $CDCl_3$) δ [ppm] = 152.22, 139.82, 139.80, 124.45, 124.34, 123.63, 122.59, 119.27, 75.33, 38.32, 12.97.

HRMS (EI+): exact mass calcd for $C_{12}H_{12}OS$ [M^+]: m/z = 204.06034; found 204.06025.

The spectral data was compared to literature values.[41]

GC-FID analysis of the crude reaction mixture, rt [min] = 9.10 (standard), 11.10 (starting material), 15.84 (product).

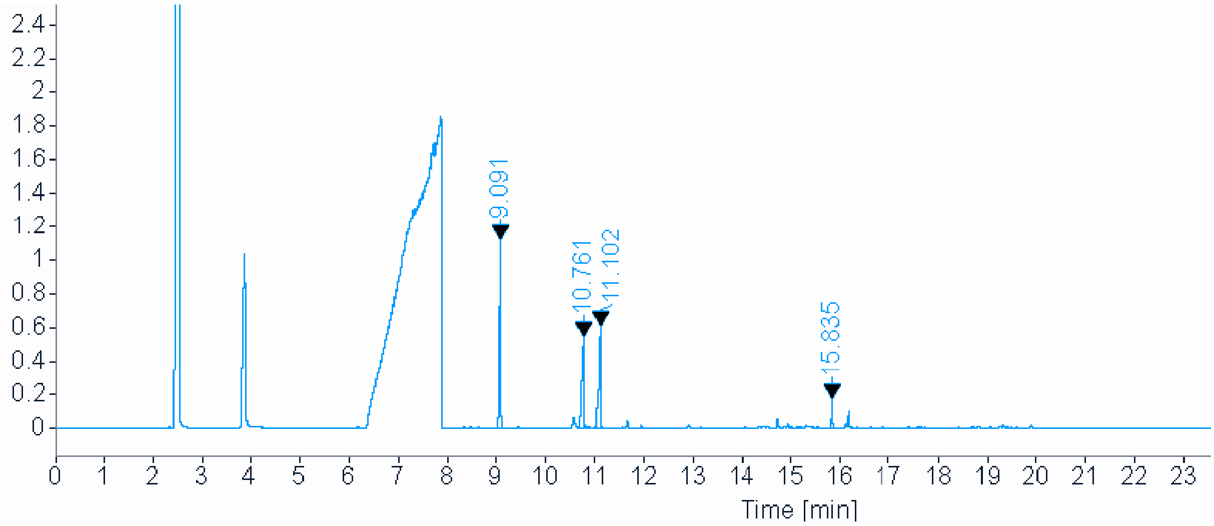

2-(cyclopent-1-en-1-yl)benzo[b]thiophene 5d

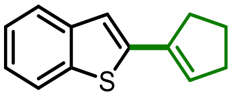

The title compound was synthesized according to general procedure E, using benzothiophene (13.4 mg, 0.1 mmol, 1.0 equiv) and cyclopentanon (85 µl, 1.0 mmol, 10 equiv.). To isolate the compound, the combined contents of four reaction run in parallel were transferred with water and brine to a separatory funnel, extracted with ethyl acetate (3 × 15 ml), the combined organic phases dried over $MgSO_4$, filtered, and concentrated. The crude was purified by flash column chromatography (PE/EA 0-50%) to yield **5d** as white crystals (10%, 8.0 mg, 0.04 mmol). The alcohol group was likely lost by water elimination on the acidic silica. Unreacted starting material was recovered and confirmed by GC-FID analysis.

^{1}H NMR (400 MHz, $CDCl_3$) δ = 7.74 (m, 1H), 7.68 (m, 1H), 7.37 – 7.22 (m, 2H), 7.07 (s, 1H), 6.15 (p, *J*=2.4, 1H), 2.79 (m, 2H), 2.56 (m, 2H), 2.06 (qd, *J*=7.8, 6.8, 2H).

^{13}C NMR (101 MHz, $CDCl_3$) δ = 141.18, 140.50, 139.22, 137.42, 129.27, 124.38, 123.43, 122.21, 120.24, 33.96, 33.62, 23.56.
The spectral data was compared to literature values.[43]
HRMS (EI+): exact mass calcd for $C_{13}H_{12}S$ [M^+]: m/z = 200.06542, found 200.06537.
GC-FID analysis of crude reaction mixture, rt [min] = 9.10 (standard), 11.10 (starting material), 12.82-12.85 (aldol condensation of cyclopentanon), 16.66 (product), 17.04 (product, alcohol form intact).

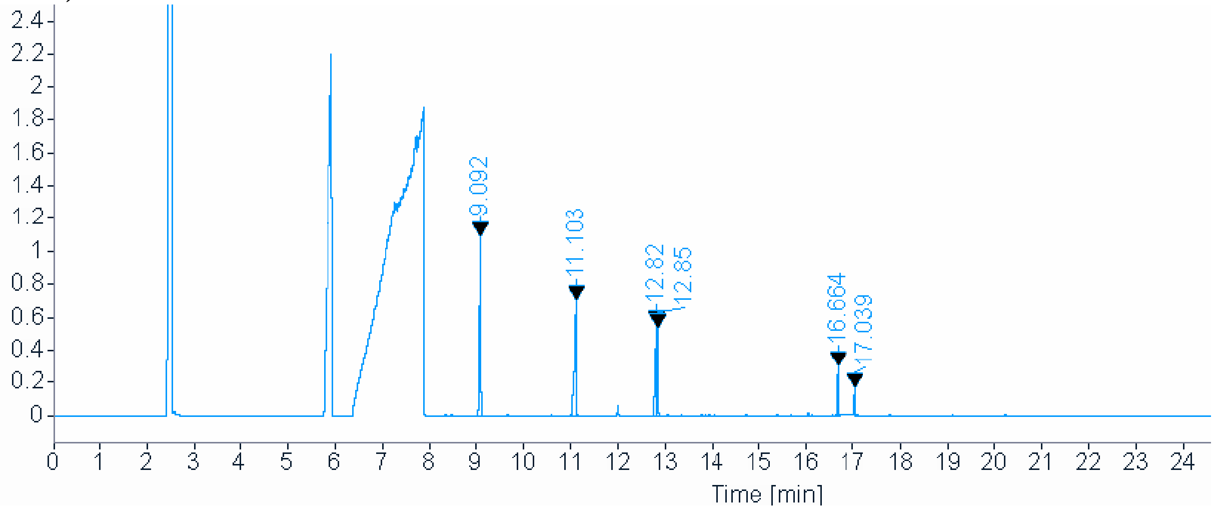

benzo[b]thiophene-2-carboxylic acid 5e

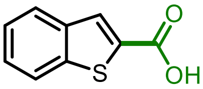

The title compound was synthesized according to procedure E. The trapping agent CO_2 (22 cm^3) was added *via* Luerlock syringe. To isolate the compound the contents of four reactions run in parallel were combined and transferred with water and diethyl ether to an extraction funnel. The ether layer was extracted with water (3 × 10 ml) and the combined aqueous layers were acidified with aqueous HCl (4 M) to adjust to an acidic pH. The aqueous layer was then extracted with ethyl acetate (3 × 15 ml) and the combined ethyl acetate layers were dried over $MgSO_4$, filtered, and concentrated *in vacuo*. The crude material was purified by silica flash column chromatography using petroleum ether and ethyl acetate with 0.5% acetic acid as eluents to give **5e** as off-white solid (22%, 16 mg, 0.09 mmol).
^{1}H NMR (300 MHz, DMSO-d_6) δ = 13.46 (s, 1H), 8.11 (s, 1H), 8.08 – 7.87 (m, 2H).
^{13}C NMR (75 MHz, DMSO-d_6) δ 163.56, 141.33, 138.75, 134.75, 130.28, 127.06, 125.77, 125.09, 123.00, 40.35, 40.07, 39.79, 39.52, 39.24, 38.96, 38.68.
HRMS (EI+): exact mass calcd for $C_9H_6O_2S$ [M^+]: m/z =178.00830; found 178.00864.
The spectral data was compared to literature values.[41]

Diisopropyl (4-methoxy-3-(trifluoromethyl)phenyl)phosphonate 6a

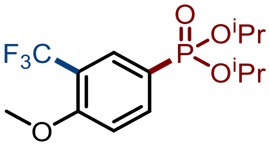

The title compound was synthesized according to general procedure D using 4-bromo-1-methoxy-2-(trifluoromethyl)benzene (16.3 µl, 0.10 mmol, 1.0 equiv) and triisopropylphosphite (247 µl, 1.0 mmol, 10 equiv). The yield of the desired product was determined by ^{19}F NMR of the crude reaction mixture with fluorobenzene (10 µl, 0.10 mmol, 1.0 equiv) as an internal standard. NMR-yield: 47%.
^{19}F NMR (376 MHz, $CDCl_3$) δ [ppm] = -62.73 (starting material), -62.74 (product), -113.15 (standard).

To isolate the compound the contents of three reactions run in parallel were transferred with water and brine to a separatory funnel and extracted with ethyl acetate (3 × 15 ml). The combined organic phases were dried over $MgSO_4$ and evaporated. The crude was purified by flash column chromatography (PE:acetone, 0 - 60%) to obtain compound **6a** as yellow oil (28 mg, 0.082 mmol, 27%).
^{1}H NMR (400 MHz, $CDCl_3$) δ [ppm] = 8.04 – 7.91 (m, 2H), 7.05 (dd, *J*=8.5, 3.1, 1H), 4.67 (dhept, *J*=8.0, *J*=6.2, 2H), 3.95 (s, 3H), 1.36 (d, *J*=6.2, 6H), 1.22 (d, *J*=6.2, 6H).
^{13}C NMR (101 MHz, $CDCl_3$) δ [ppm] = 160.44 (dd, *J*=3.0, 1.6), 137.54 (d, *J*=10.9), 131.04 (dq, *J*=12.4, 5.3), 123.28 (dd, *J*=272.8, 2.2), 122.66, 120.69, 119.03 (dq, *J*=31.5, 15.9), 71.12 (d, *J*=5.6), 56.23, 24.17 (d, *J*=4.1), 23.97 (d, *J*=4.8).
^{19}F NMR (376 MHz, $CDCl_3$) δ [ppm] = -63.34.
^{31}P NMR (162 MHz, $CDCl_3$) δ [ppm] = 15.95.
HRMS (ESI+): exact mass calcd for $C_{10}H_{20}F_3O_4P^+$ [M+H$^+$]: m/z = 341.1124, found: 341.1129.

Dimethyl (4-methoxy-3-(trifluoromethyl)phenyl)phosphonate 6b

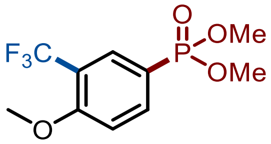

The title compound was synthesized according to general procedure D using 4-bromo-1-methoxy-2-(trifluoromethyl)benzene (16.3 µl, 0.10 mmol, 1.0 equiv) and trimethylphosphite (173 µl, 1.0 mmol, 10 equiv). The yield of the desired product was determined by ^{19}F NMR of the crude reaction mixture with fluorobenzene (10 µl, 0.10 mmol, 1.0 equiv) as an internal standard. NMR-yield: 61%.
^{19}F NMR (376 MHz, $CDCl_3$) δ [ppm] = -62.72 (starting material), -62.82 (product), -113.15 (standard).
To isolate the compound the contents of three reactions run in parallel were transferred with water and brine to an extraction funnel and extracted with ethyl acetate (3 × 15 ml). The combined organic fractions were dried over $MgSO_4$, filtered and concentrated. The crude was purified by flash column chromatography (DCM/MeOH, 0 - 10%). To remove leftover phosphonate sideproducts, the crude was further purified by preparative TLC (PE/EA/DCM/MeOH, 5:5:9:1, product visible under 254 nm on TLC, phosphonate sideproducts can be stained with potassium permanganate stain) to yield compound **6b** as colorless oil (21 mg, 0.07 mmol, 25%).
^{1}H NMR (400 MHz, $CDCl_3$) δ [ppm] = 8.01 – 7.91 (m, 2H), 7.09 (dd, *J*=9.1, 3.1, 1H), 3.96 (s, 3H), 3.77 (s, 3H), 3.75 (s, 3H).
^{13}C NMR (101 MHz, $CDCl_3$) δ [ppm] = 160.91, 137.89 (d, *J*=11.2), 131.23 (dq, *J*=12.3, 5.4), 123.16 (d, *J*=270.8), 119.50, 119.40 (dd, *J*=31.5, 16.3), 112.07 (d, *J*=16.0), 56.32, 52.92 (d, *J*=5.6).
^{19}F NMR (376 MHz, $CDCl_3$) δ [ppm] = -63.43.
^{31}P NMR (162 MHz, $CDCl_3$) δ [ppm] = 21.02.
HRMS (ESI+): exact mass calcd for $C_{10}H_{12}F_3O_4P^+$ [M+H$^+$]: m/z = 285.0498, found: 285.0500.

2-(4-methoxy-3-(trifluoromethyl)phenyl)-1-methyl-1H-pyrrole 6c

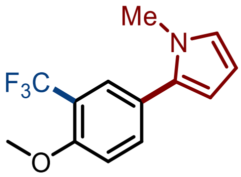

The title compound was synthesized according to general procedure D using 4-bromo-1-methoxy-2-(trifluoromethyl)benzene (16.3 µl, 0.10 mmol, 1.0 equiv) and 1-methylpyrrole (89 µl, 1.0 mmol, 10 equiv). The yield of the desired product was determined by ^{19}F NMR of the crude reaction mixture with fluorobenzene (10 µl, 0.10 mmol, 1.0 equiv) as an internal standard. NMR-yield: 28%.
^{19}F NMR (376 MHz, $CDCl_3$) δ [ppm] = -62.35 (product), -62.72 (starting material), -113.15 (standard).

To isolate the compound, the combined contents of three reactions run in parallel were transferred to a separatory funnel with water and brine and extracted with ethyl acetate (3 × 15 ml). The combined organic phases were dried over $MgSO_4$, filtered and concentrated. The crude was purified by column chromatography (PE/EA, 0 - 10%) to yield compound **6c** as colorless oil (14 mg, 0.01 mmol, 18%).
^{1}H NMR (400 MHz, $CDCl_3$) δ [ppm] = 7.60 (d, *J*=2.3, 1H), 7.52 (dd, *J*=8.5, 2.3, 1H), 7.05 (d, *J*=8.6, 1H), 6.71 (t, *J*=2.3, 1H), 6.26 – 6.15 (m, 2H), 3.94 (s, 3H), 3.63 (s, 3H).
^{19}F NMR (376 MHz, $CDCl_3$) δ [ppm] = -62.98.
^{13}C NMR (101 MHz, $CDCl_3$) δ [ppm] = 133.52, 133.07, 127.62 (q, *J*=5.2), 125.74, 123.78, 122.34, 118.70, 112.21, 108.88, 107.99, 56.21, 35.03.
HRMS (EI+): exact mass calcd for $C_{13}H_{12}F_3NO^+$ [M $^+$]: m/z = 255.08655, found: 255.08664.

9. NMR spectra

Sodium 1,4-dihydroxynaphthalene-2-sulfonate (NQH_2-1)

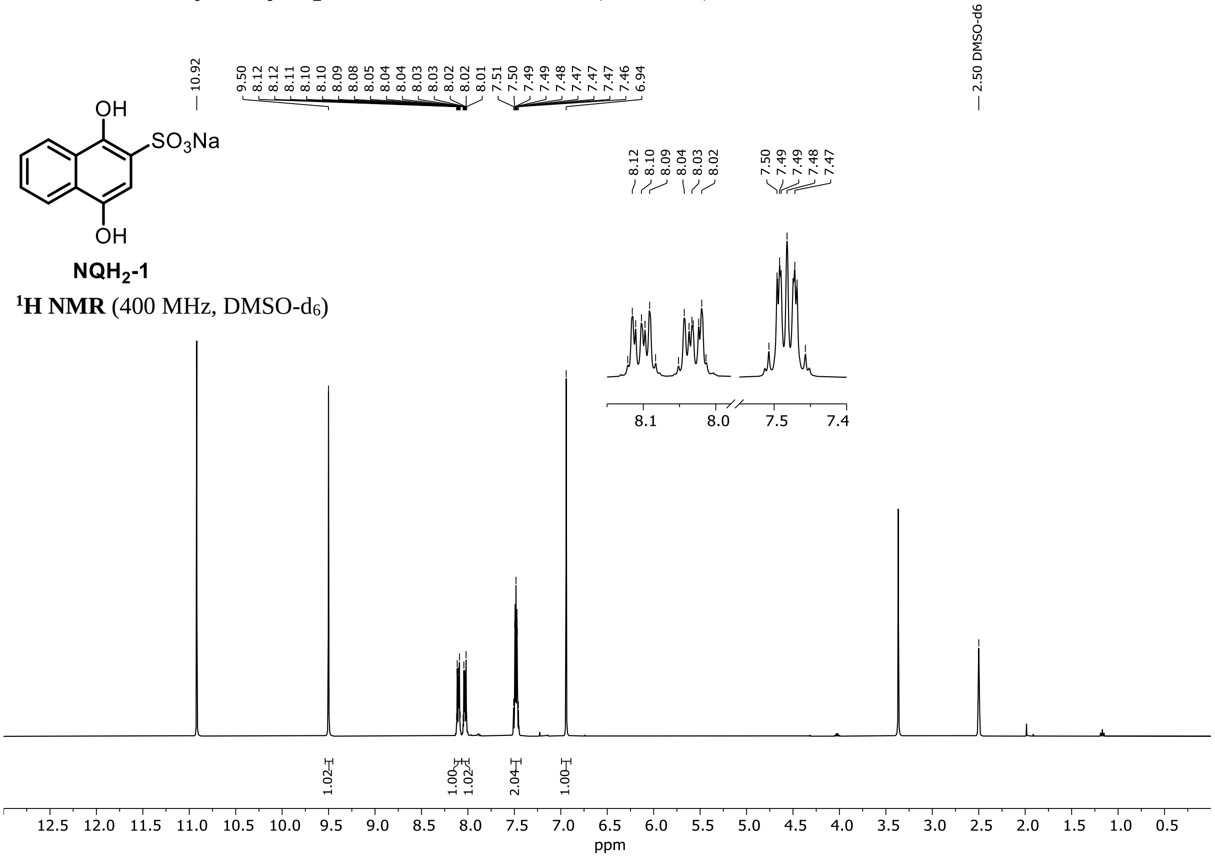

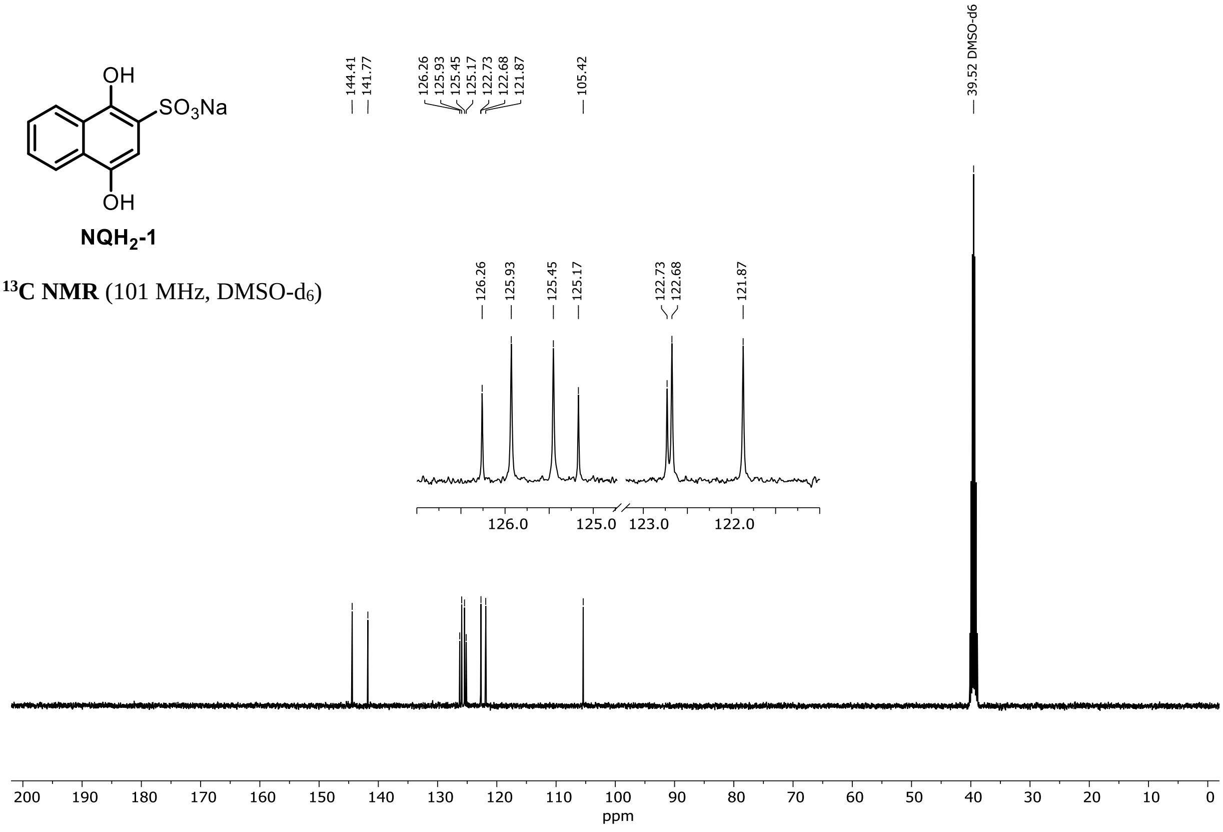

2-methylnaphthalene-1,4-diol (NQH_2-2)

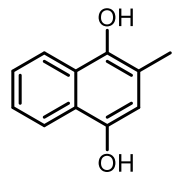

NQH_2-2

^{1}H NMR (300 MHz, DMSO-d_6)

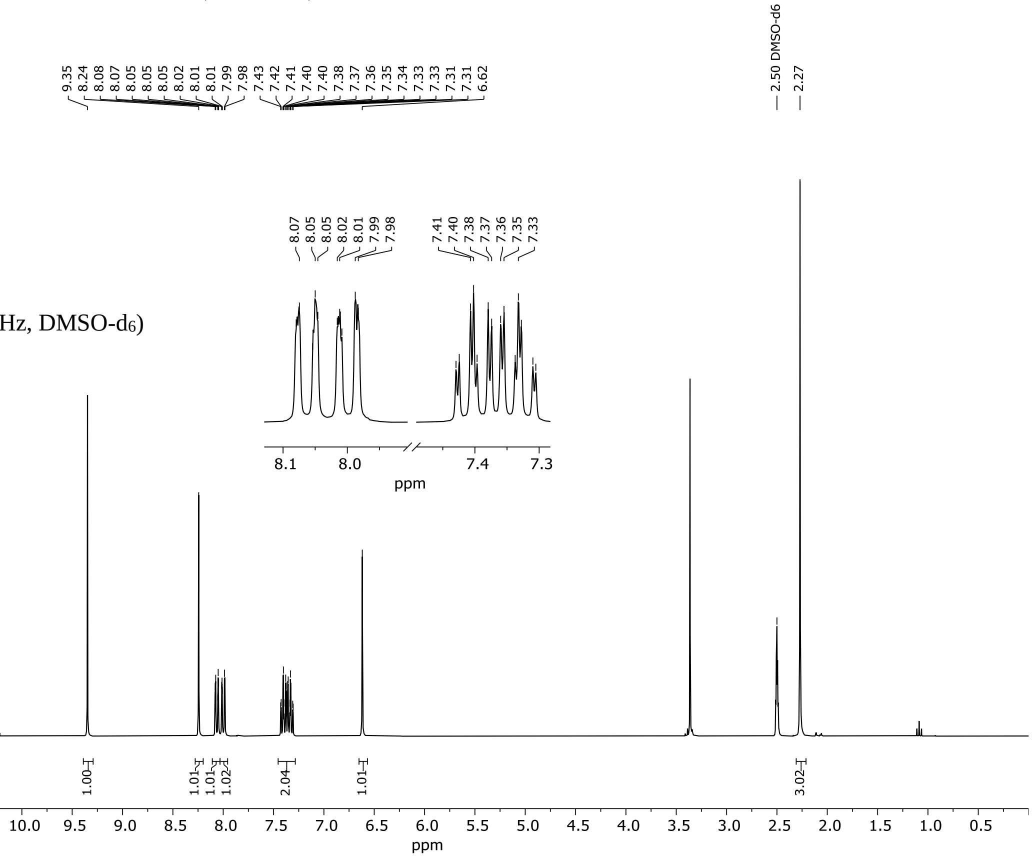

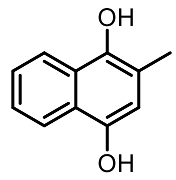

NQH_2-2

^{13}C NMR (101 MHz, DMSO-d_6)

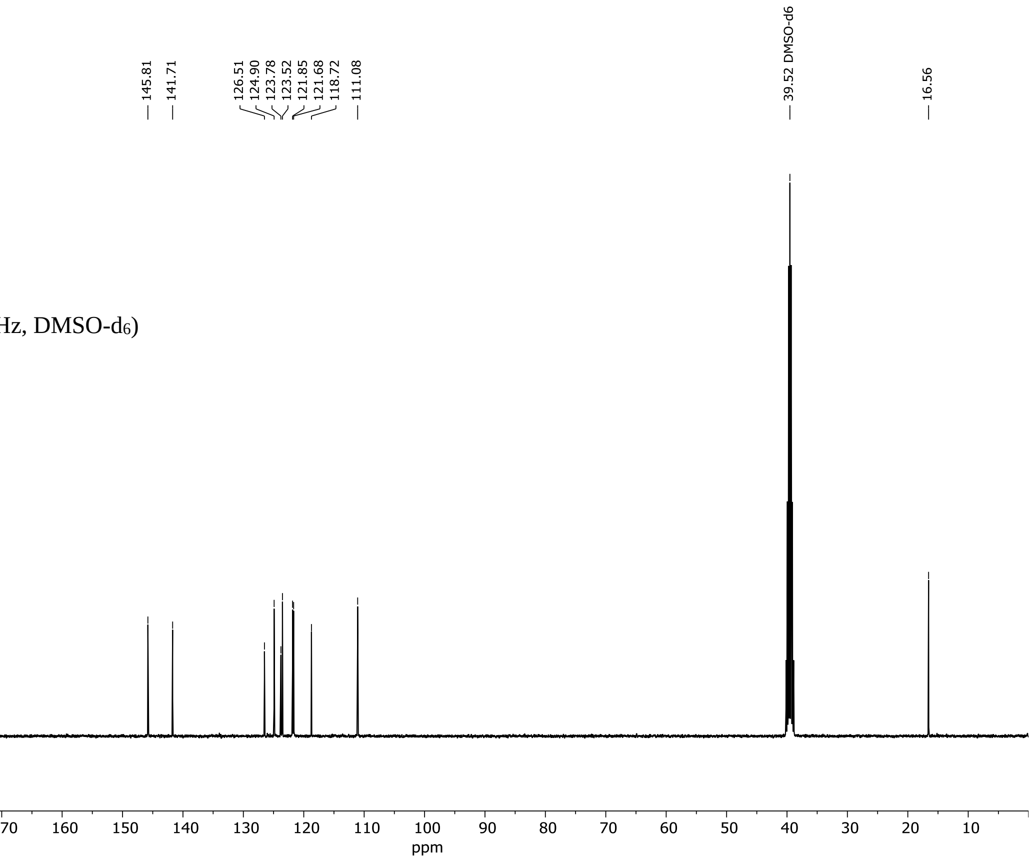

Naphthalene-1,2,4-triol (**NQH$_2$-3**)

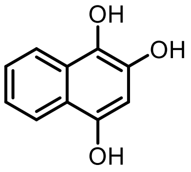

NQH$_2$-3

^{1}H NMR (300 MHz, DMSO-d$_6$)

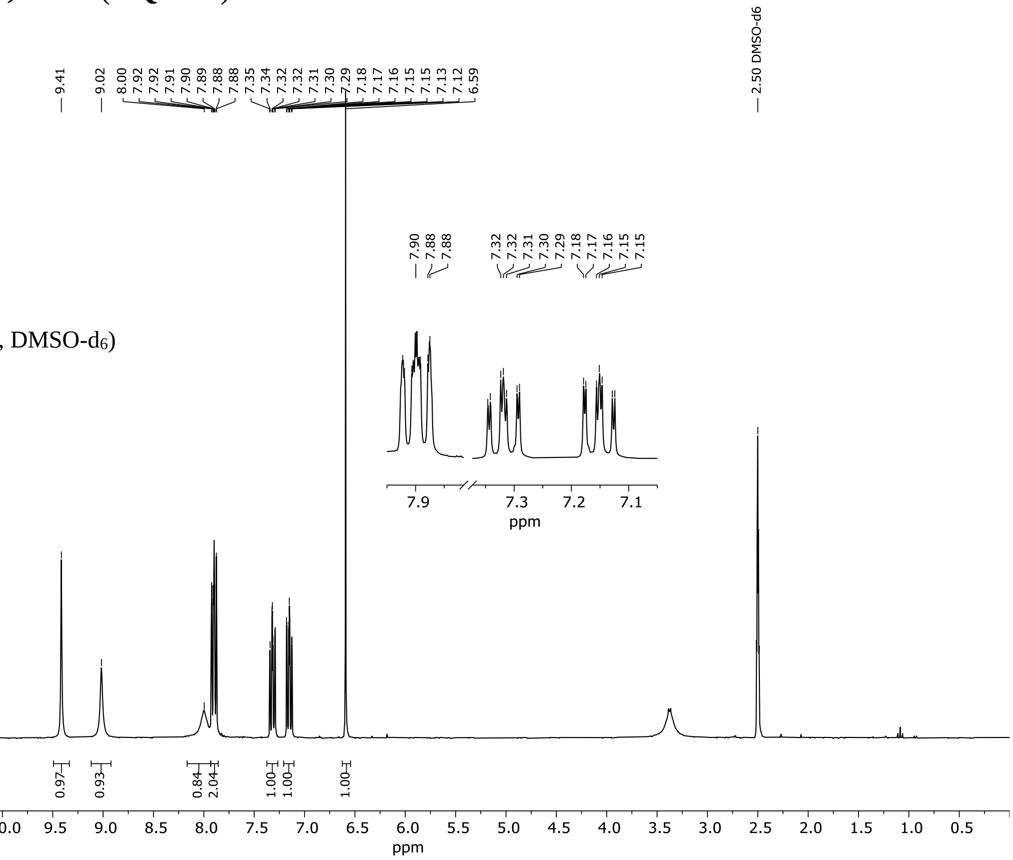

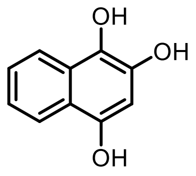

NQH$_2$-3

^{13}C NMR (101 MHz, DMSO-d$_6$)

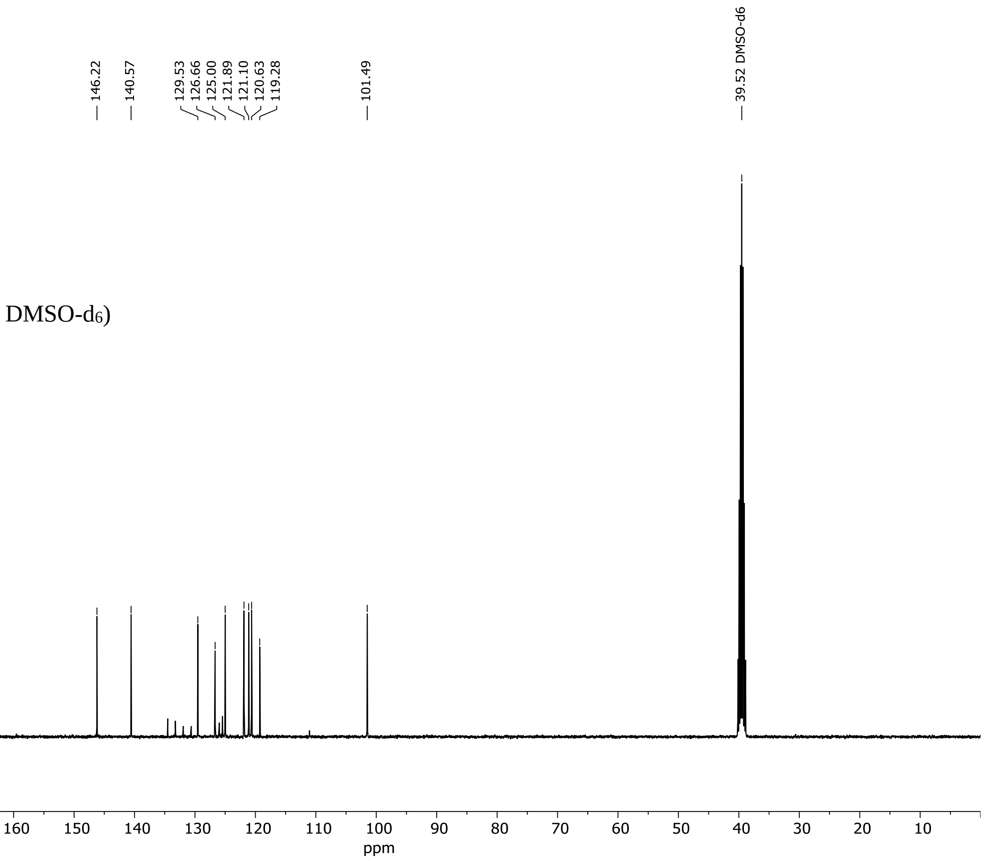

Naphthalene-1,4,5-triol (NQH2-4)

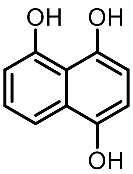

NQH$_2$-4

^{1}H NMR (300 MHz, DMSO-d6)

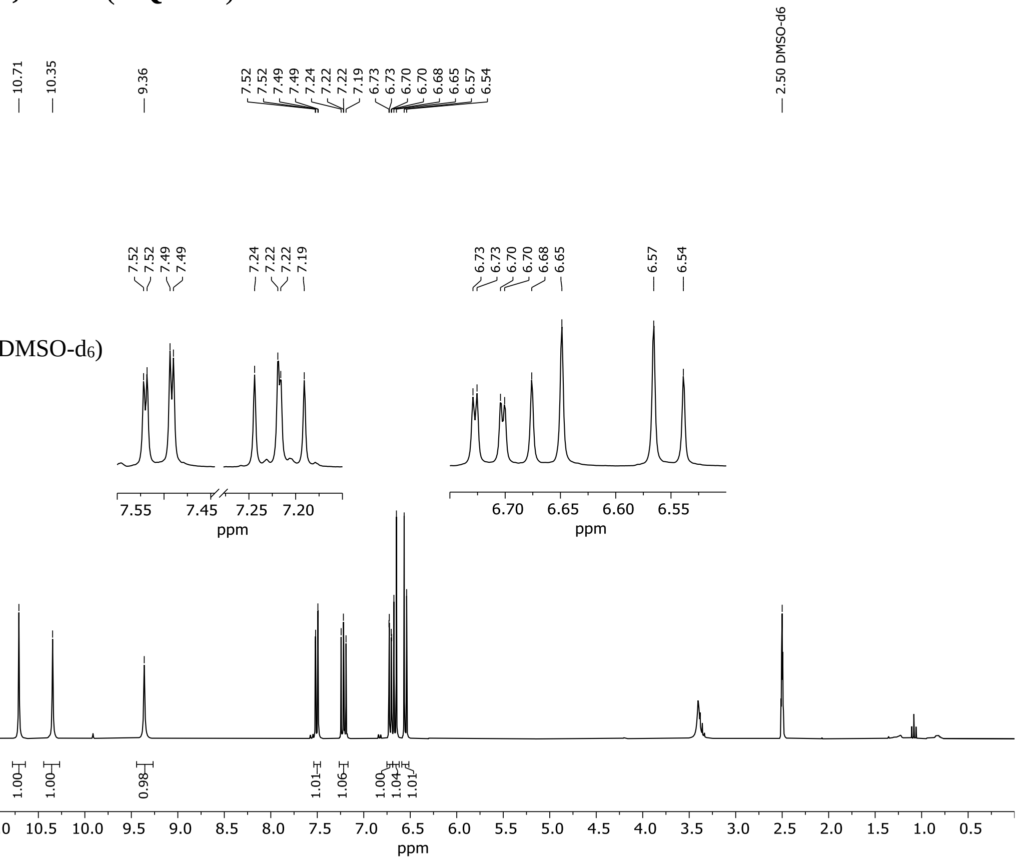

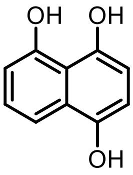

NQH$_2$-4

^{13}C NMR (101 MHz, DMSO-d6)

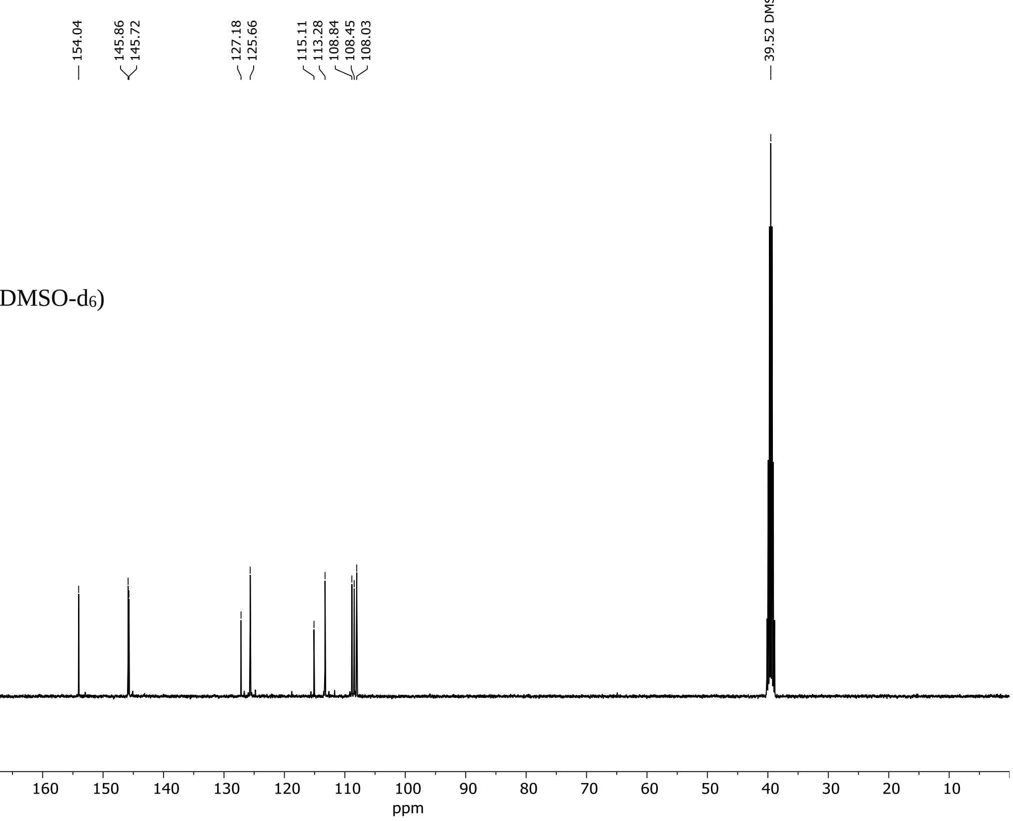

Sodium 1,4-naphthoquinone-2-sulfonate (NQ-1)

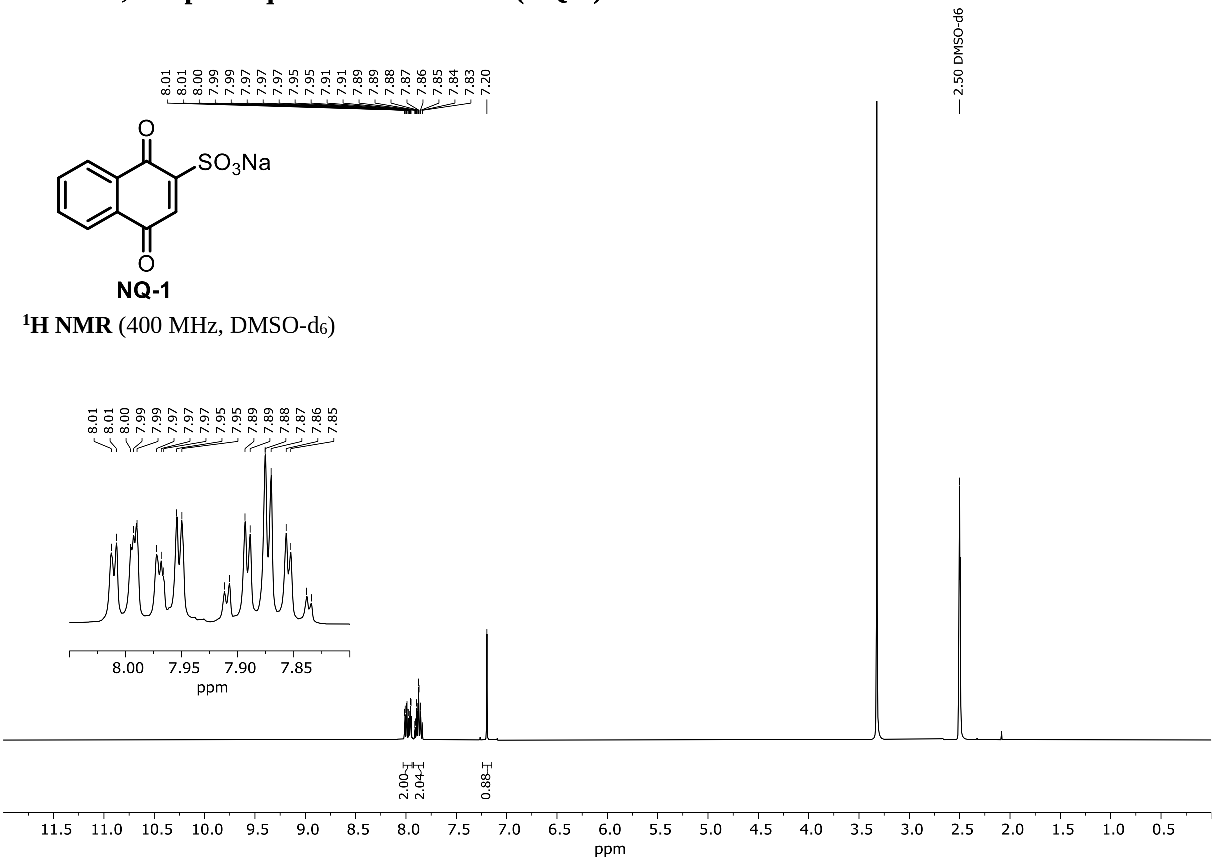

NQ-1

^{1}H NMR (400 MHz, DMSO-d6)

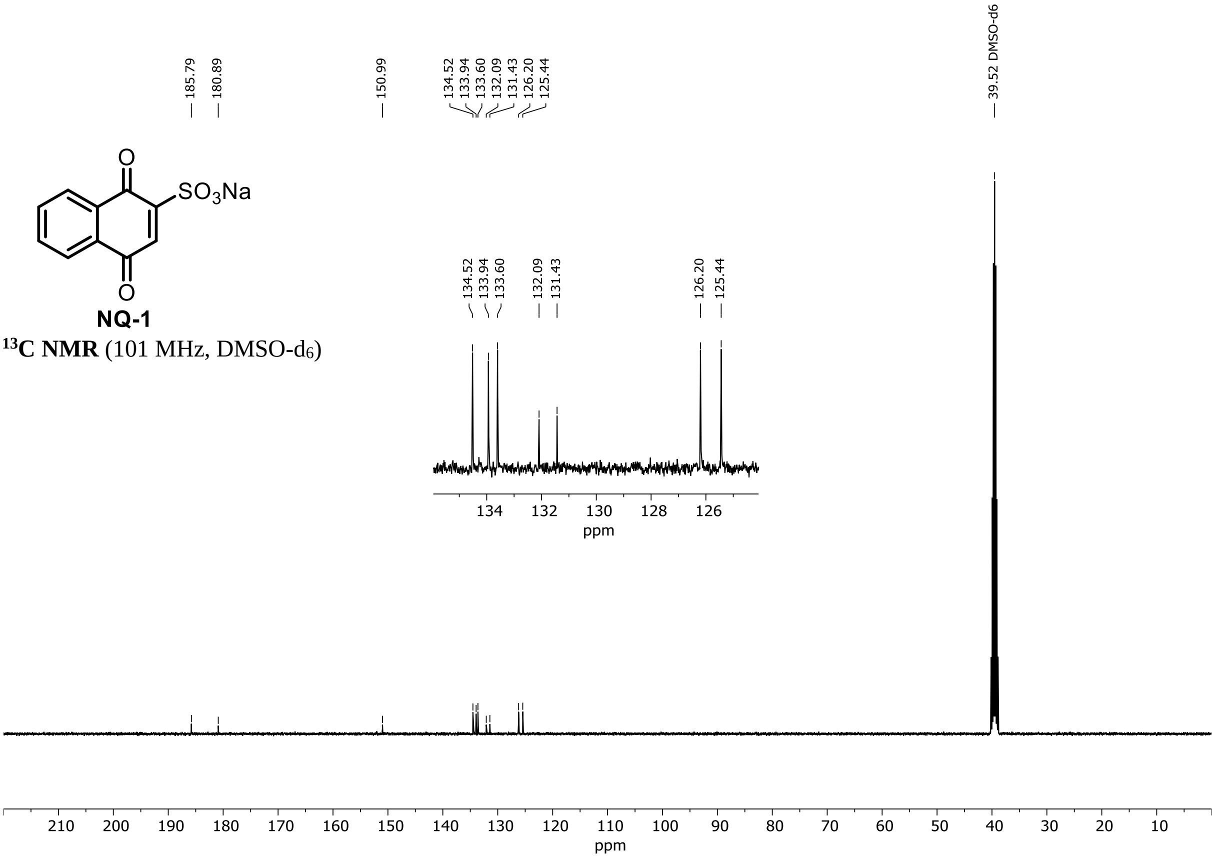

NQ-1

^{13}C NMR (101 MHz, DMSO-d6)

Oxidative compounds

1,3,5-trimethoxy-2-(trifluoromethyl)benzene 1a

Crude **^{19}F NMR** (377 MHz, $CDCl_3$)

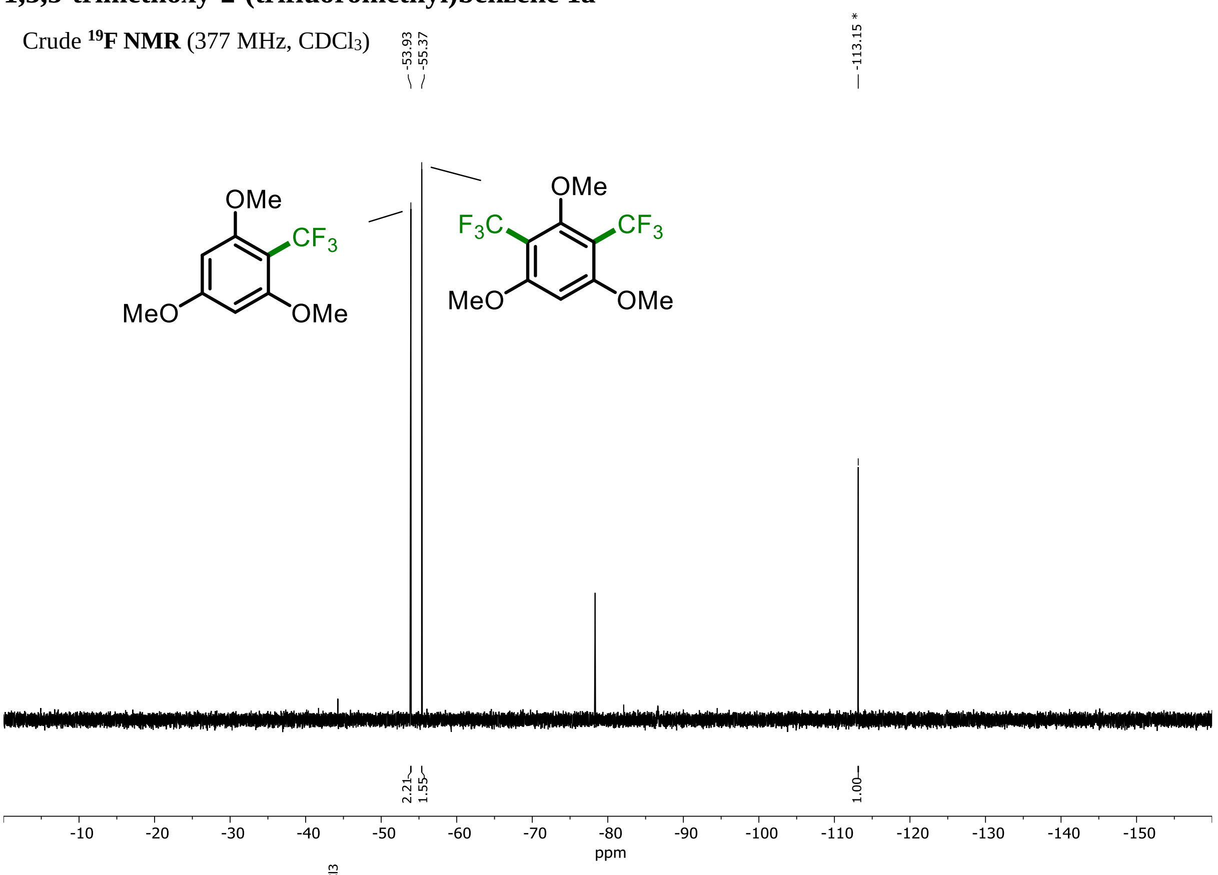

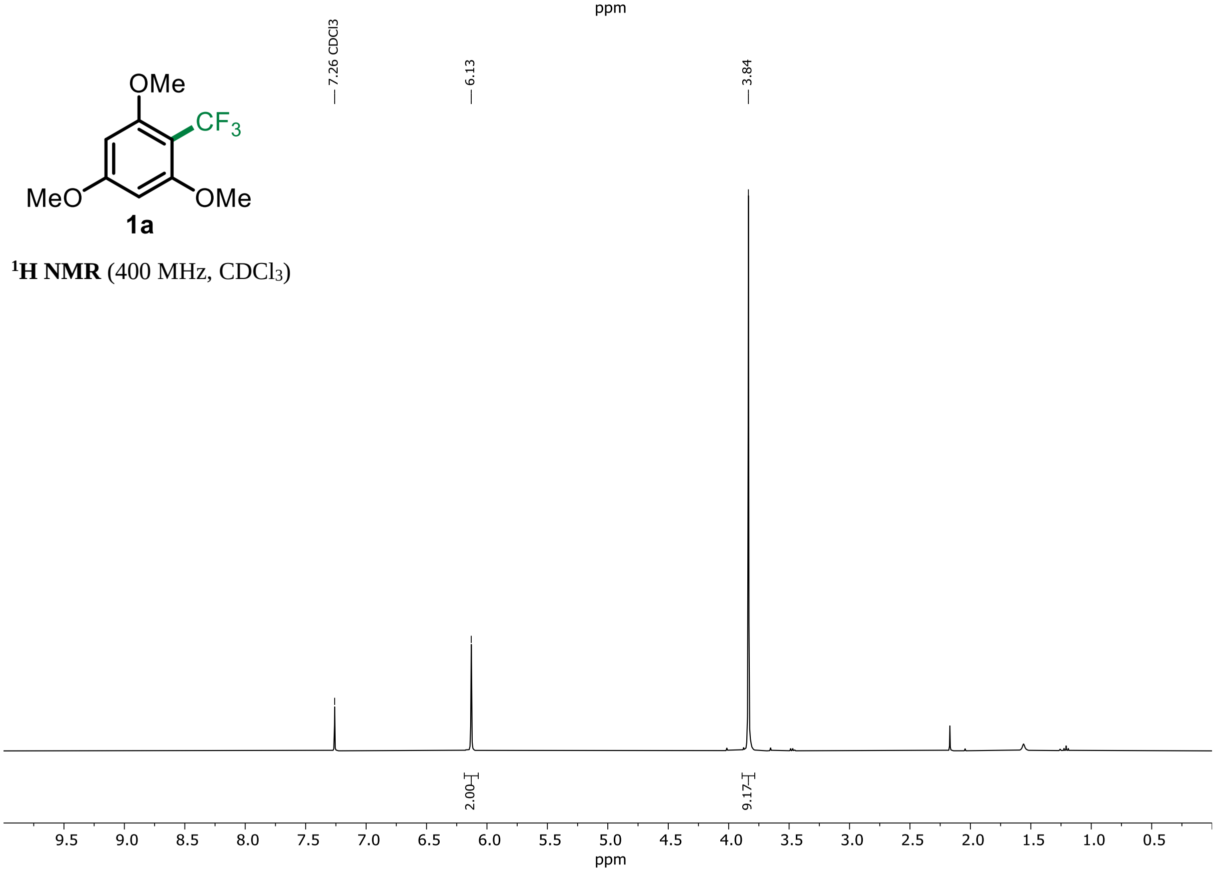

^{1}H NMR (400 MHz, $CDCl_3$)

1a

^{13}C NMR (101 MHz, $CDCl_3$)

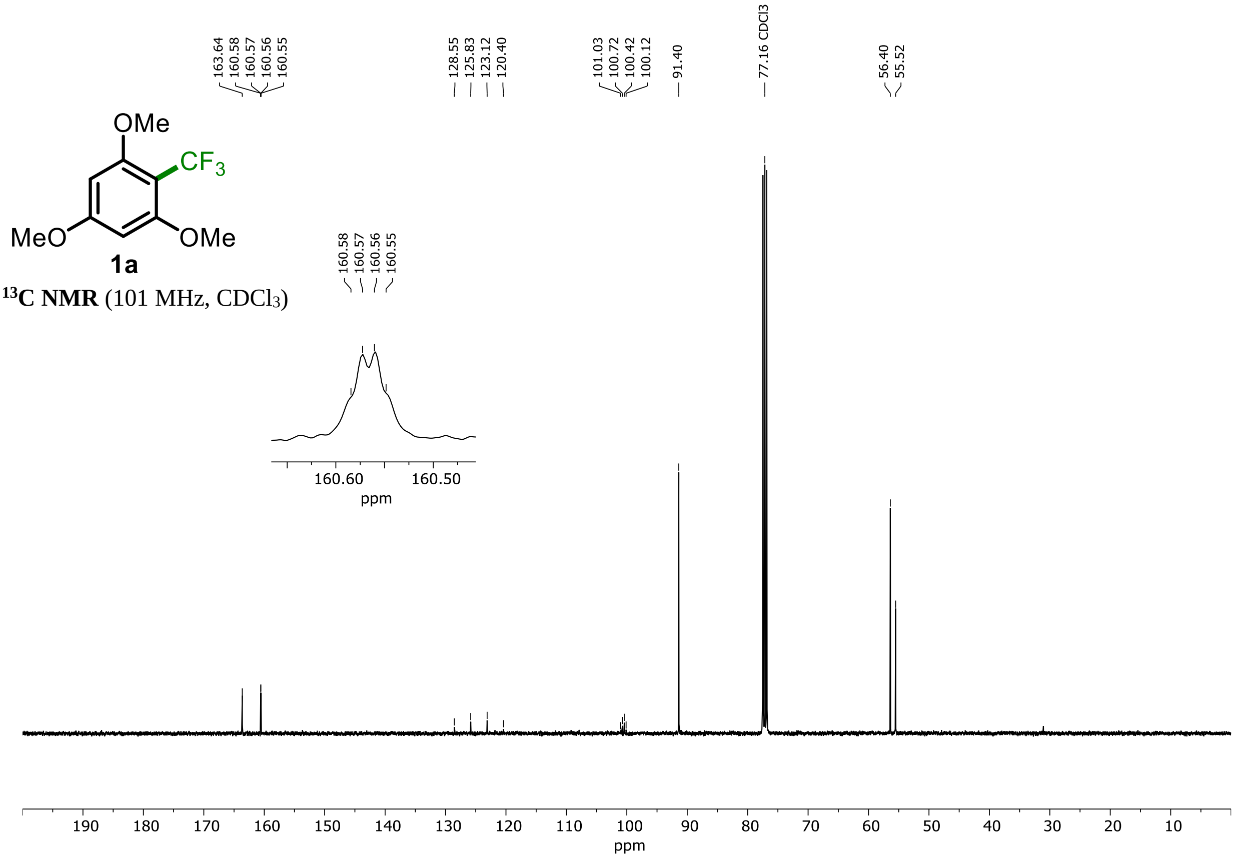

1a

^{19}F NMR (376 MHz, $CDCl_3$)

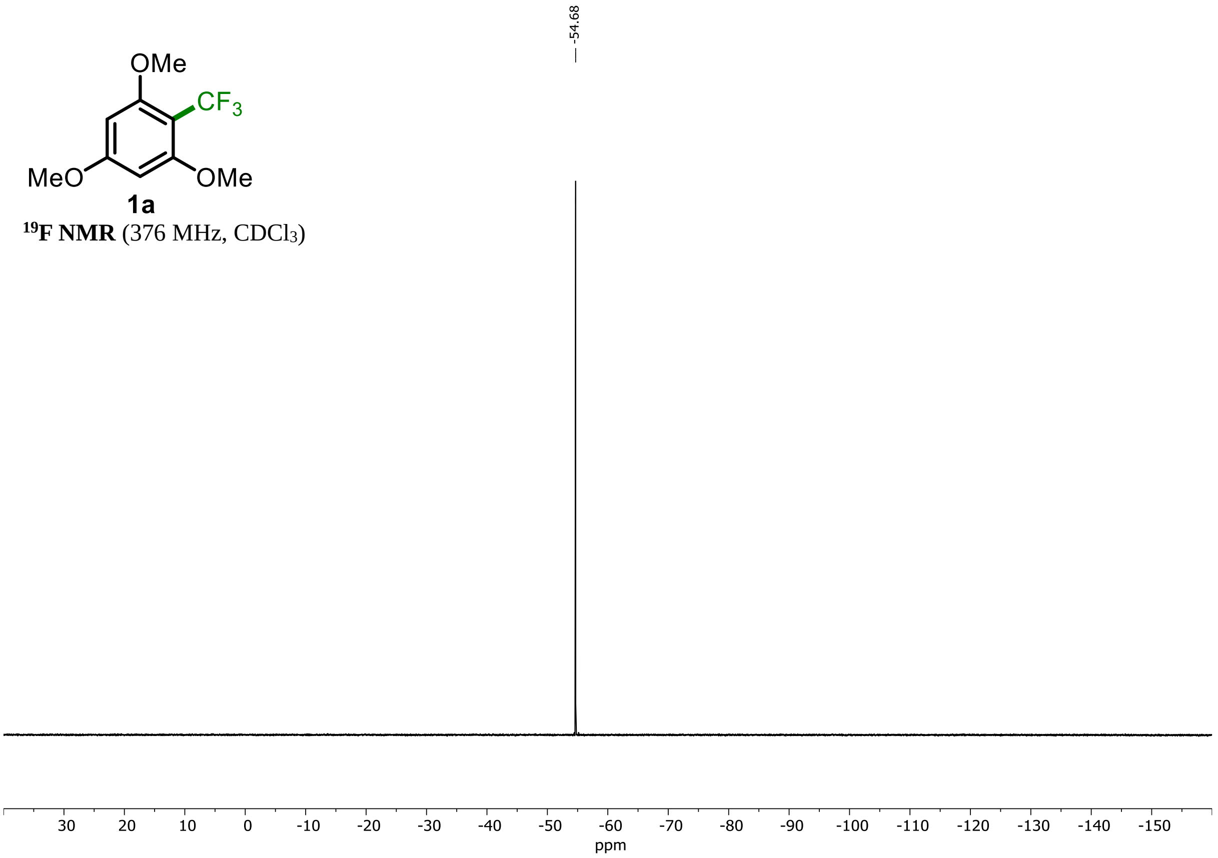

1,3-dimethoxy-4-(trifluoromethyl)benzene 1b

Crude ^{19}F NMR (377 MHz, $CDCl_3$)

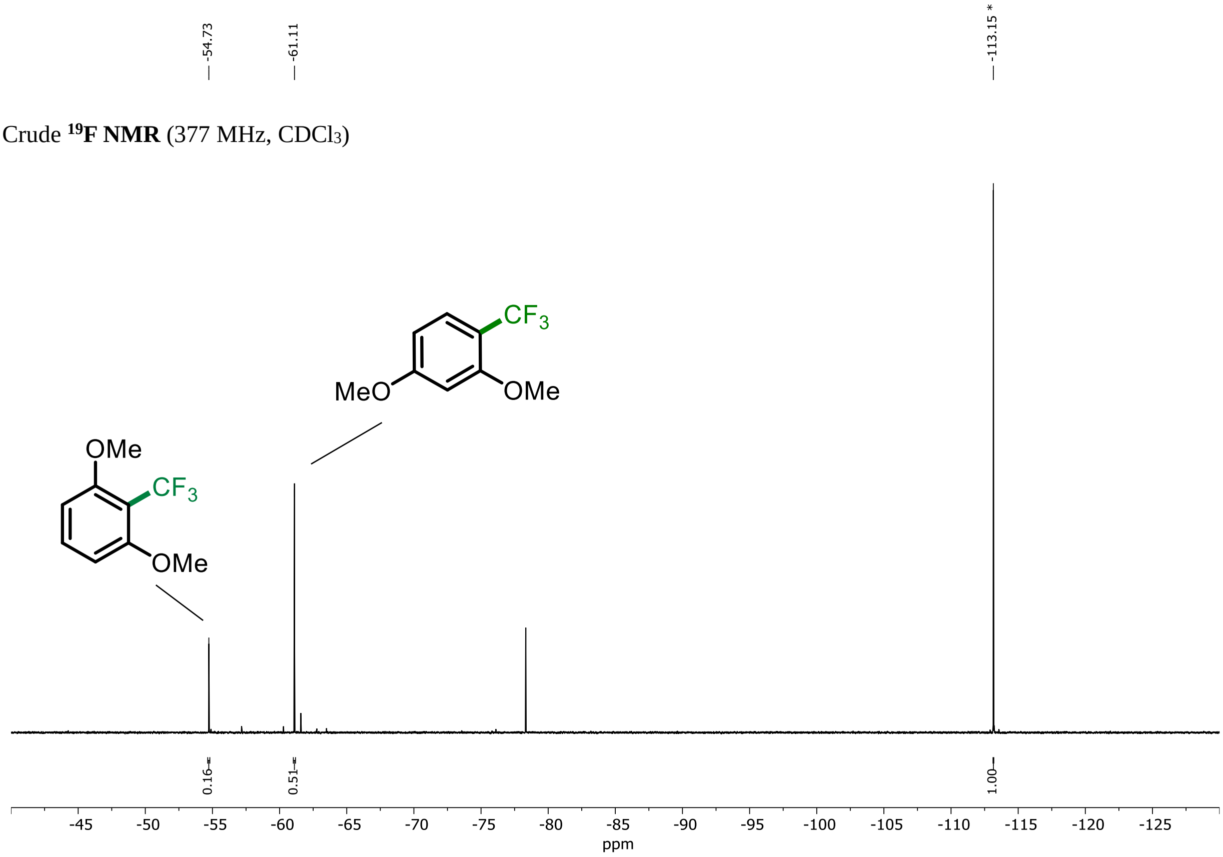

1-methoxy-2-(trifluoromethyl)benzene 1c

Crude ^{19}**F NMR** (377 MHz, $CDCl_3$)

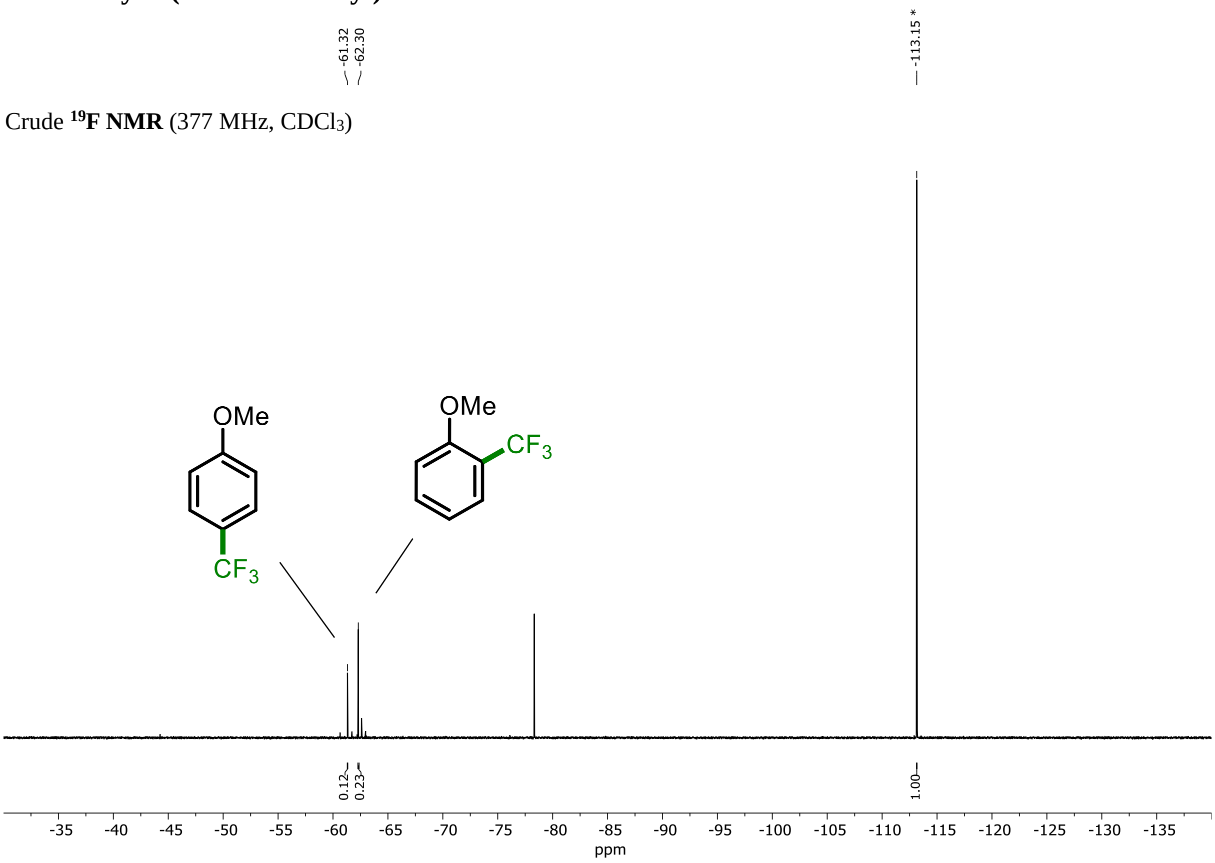

1-bromo-4-methoxy-2-(trifluoromethyl)benzene 1d

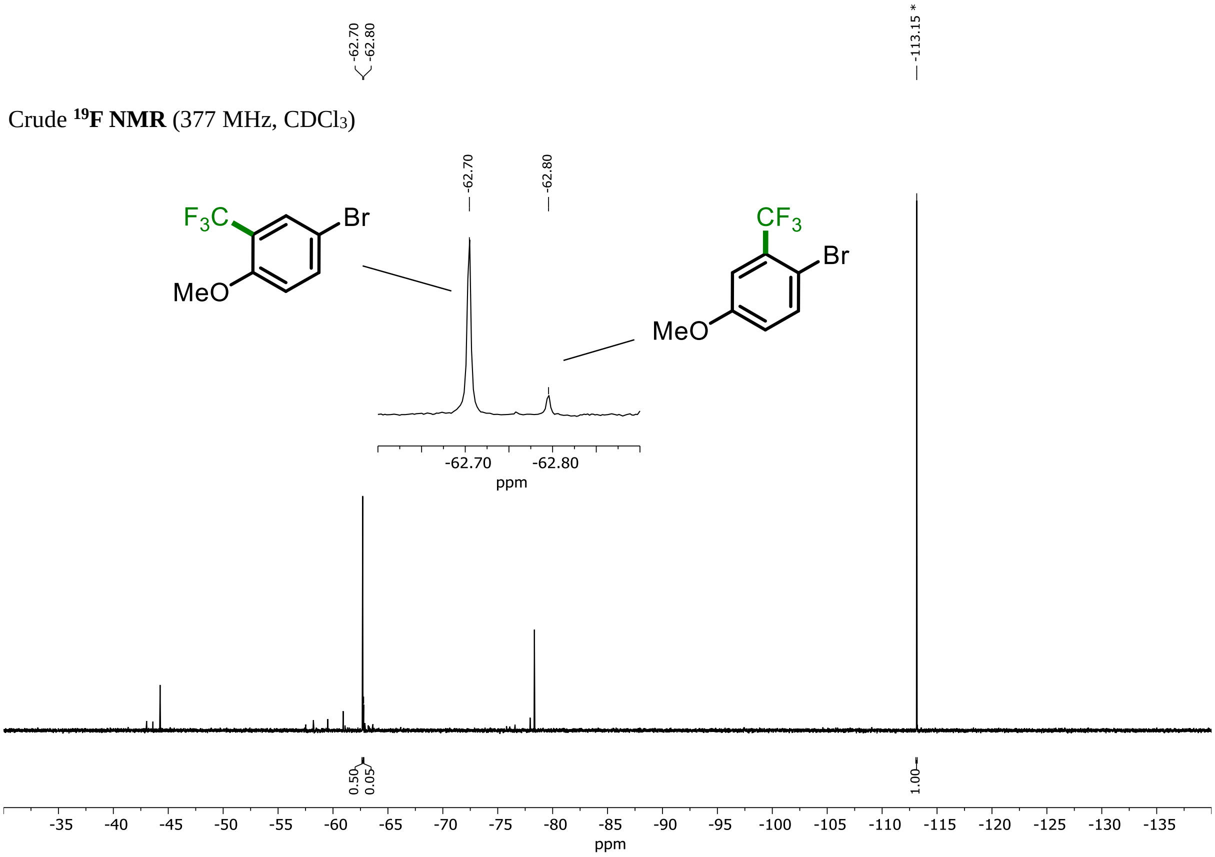

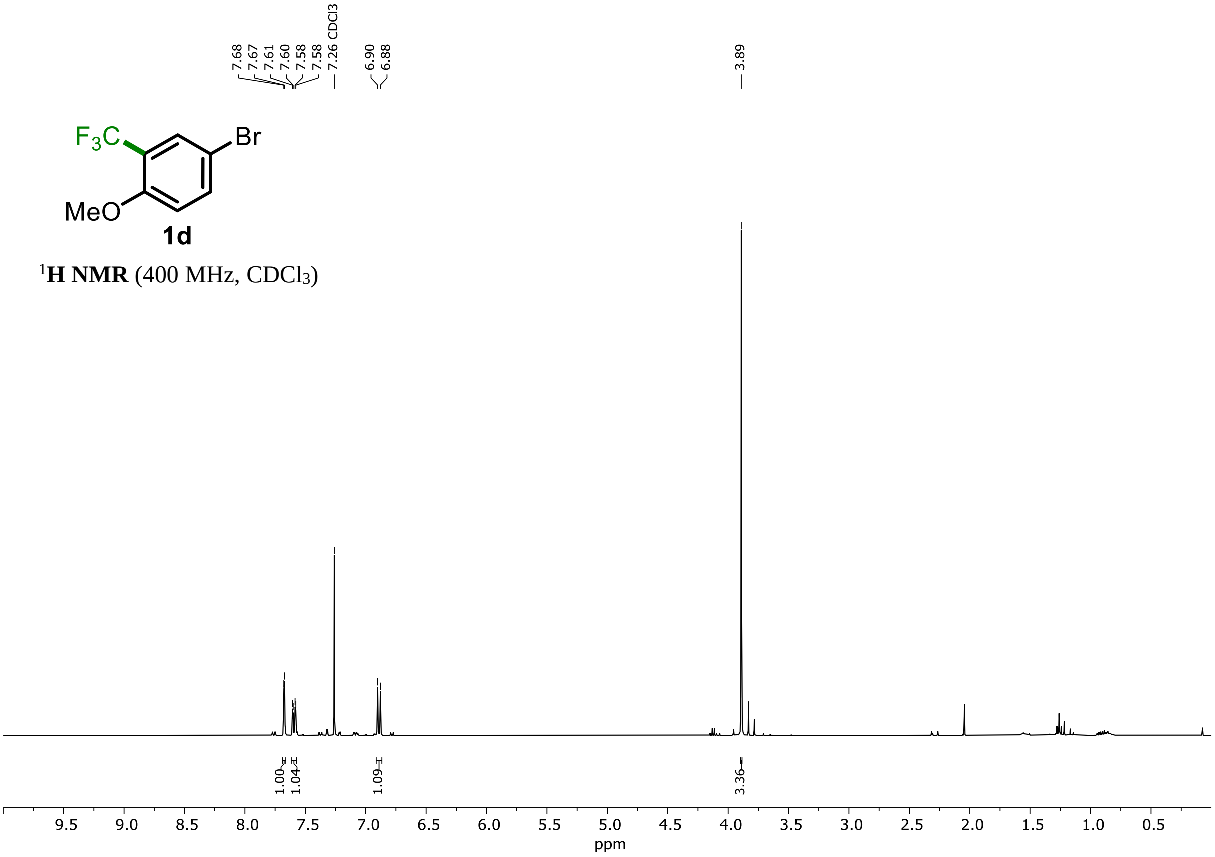

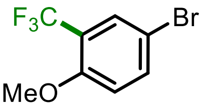

1d

^{19}F NMR (376 MHz, CDCl3)

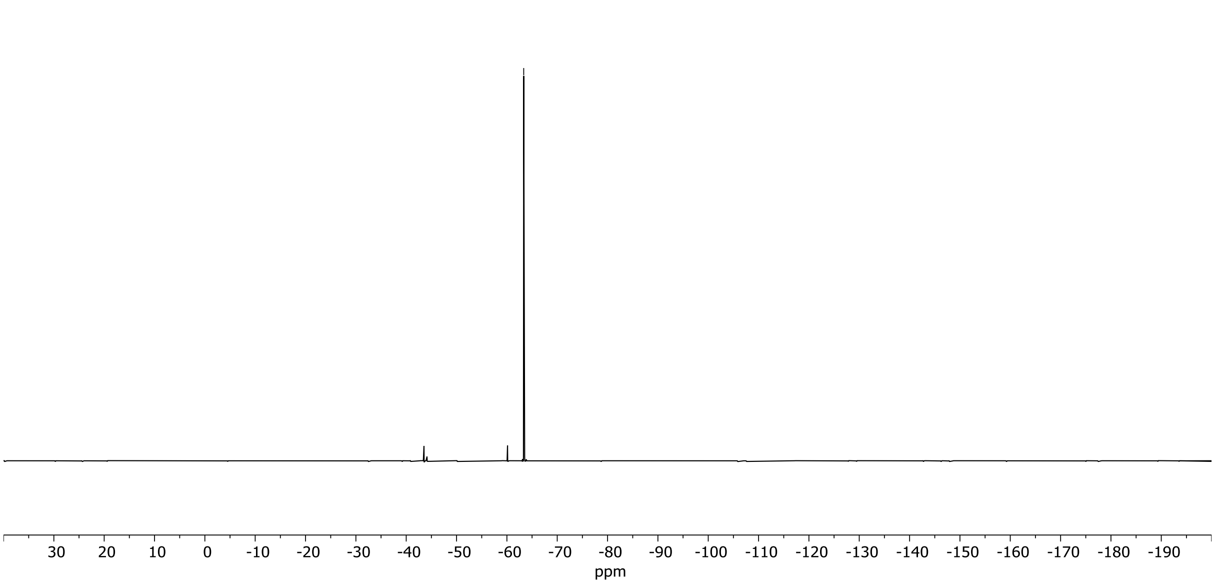

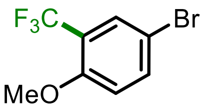

1d

^{13}C NMR (101 MHz, CDCl3)

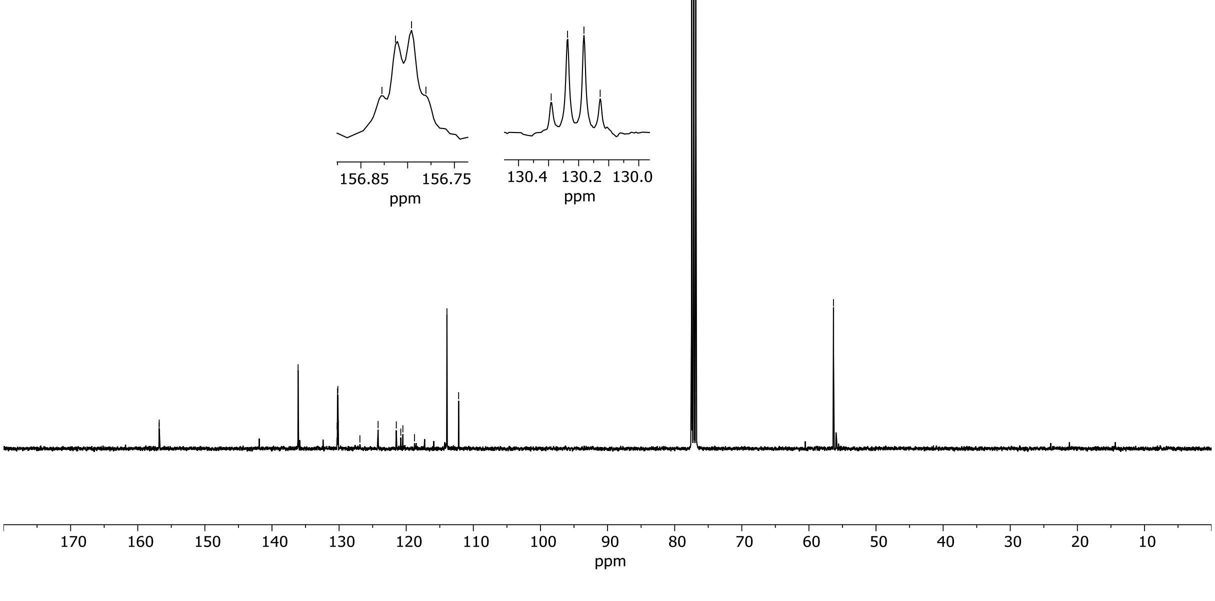

1-chloro-4-methoxy-2-(trifluoromethyl)benzene 1e

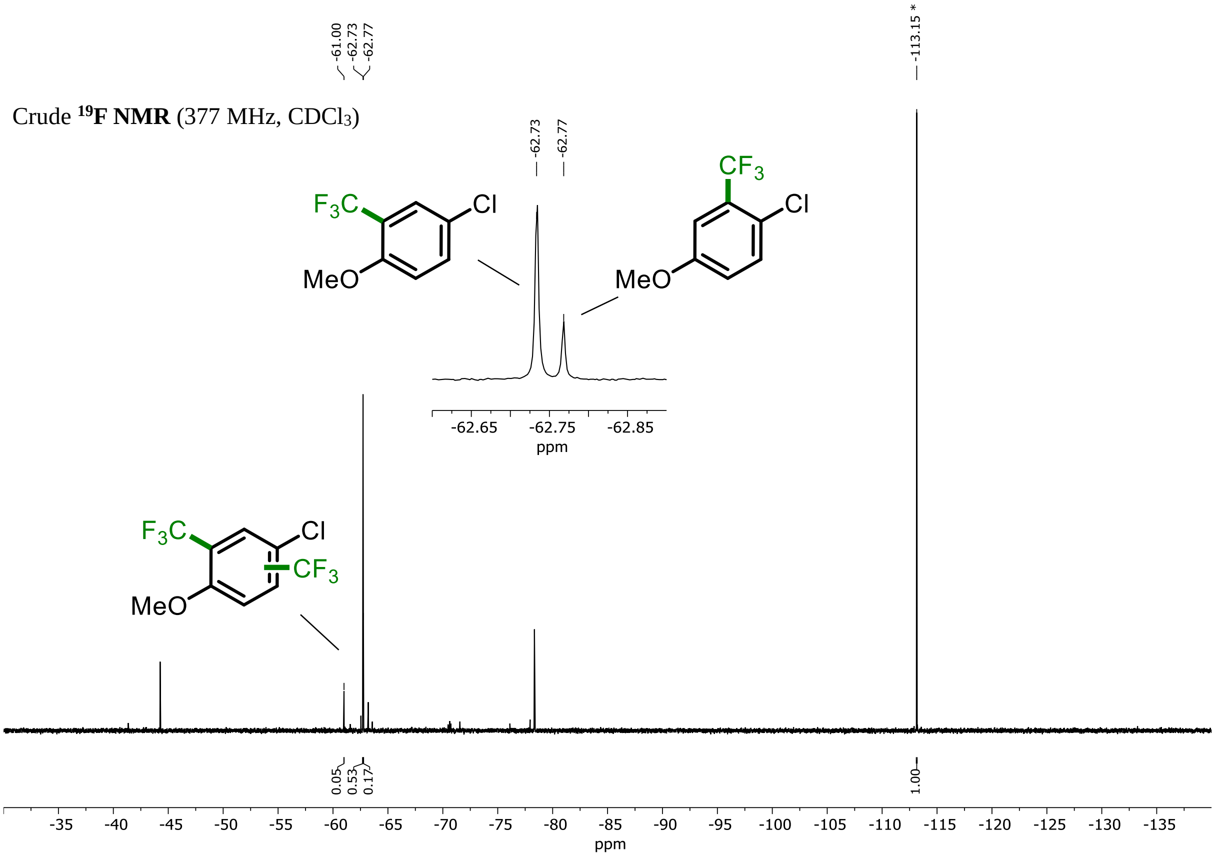

Trifluortoluene 1f

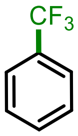

1f

Crude **^{19}F NMR** (377 MHz, $CDCl_3$)

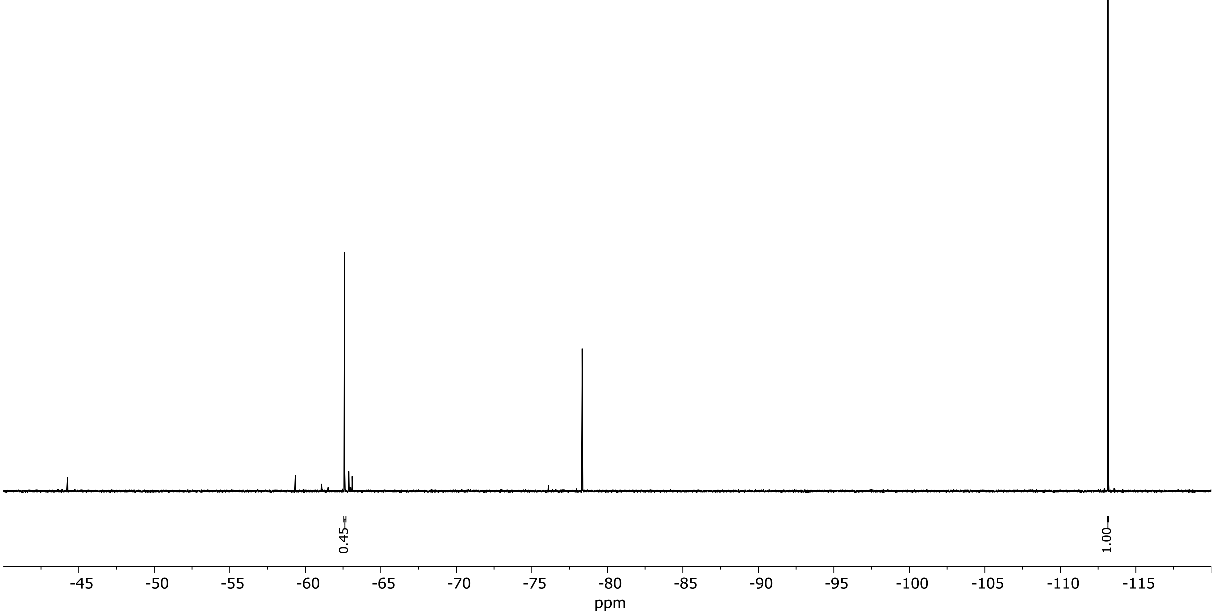

2,6-dimethoxy-3-(trifluoromethyl)pyridine 1g

Crude ^{19}**F NMR** (377 MHz, $CDCl_3$)

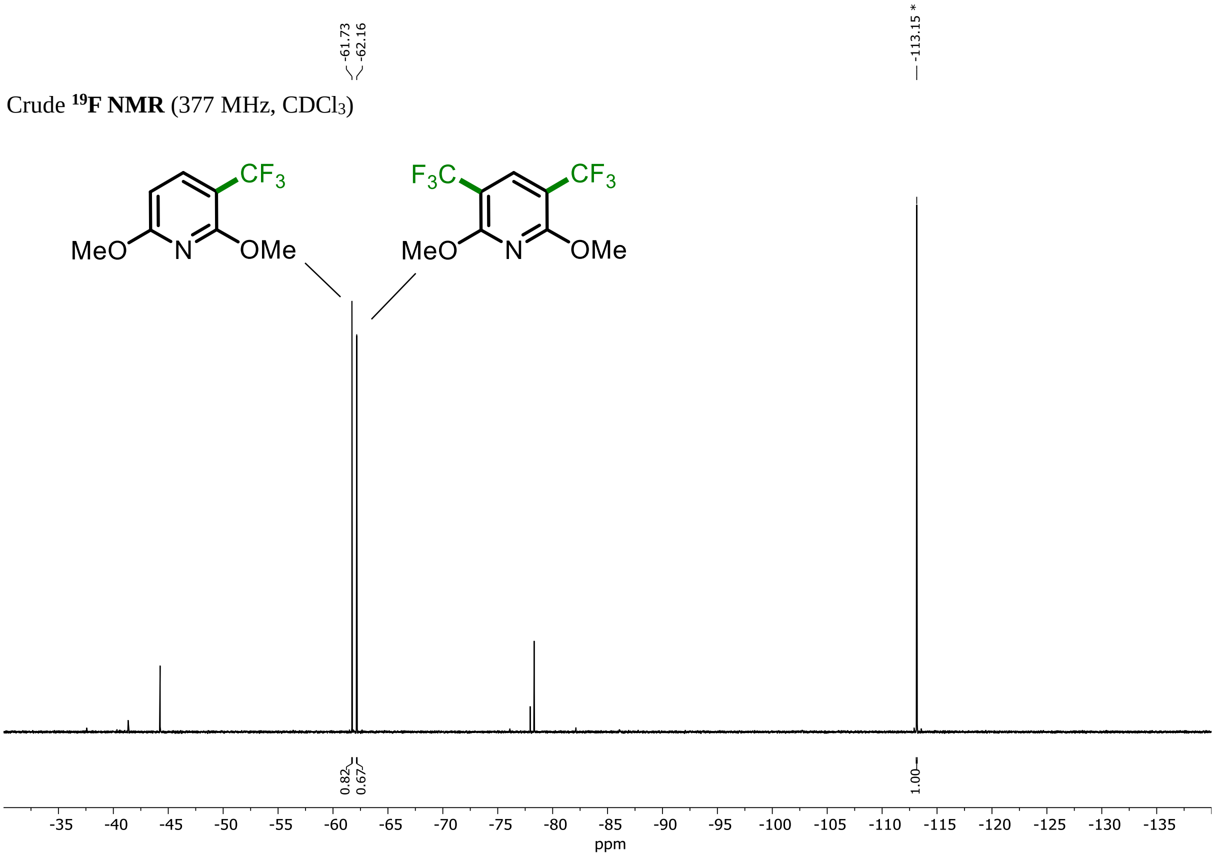

2-(trifluoromethyl)pyridine 1h

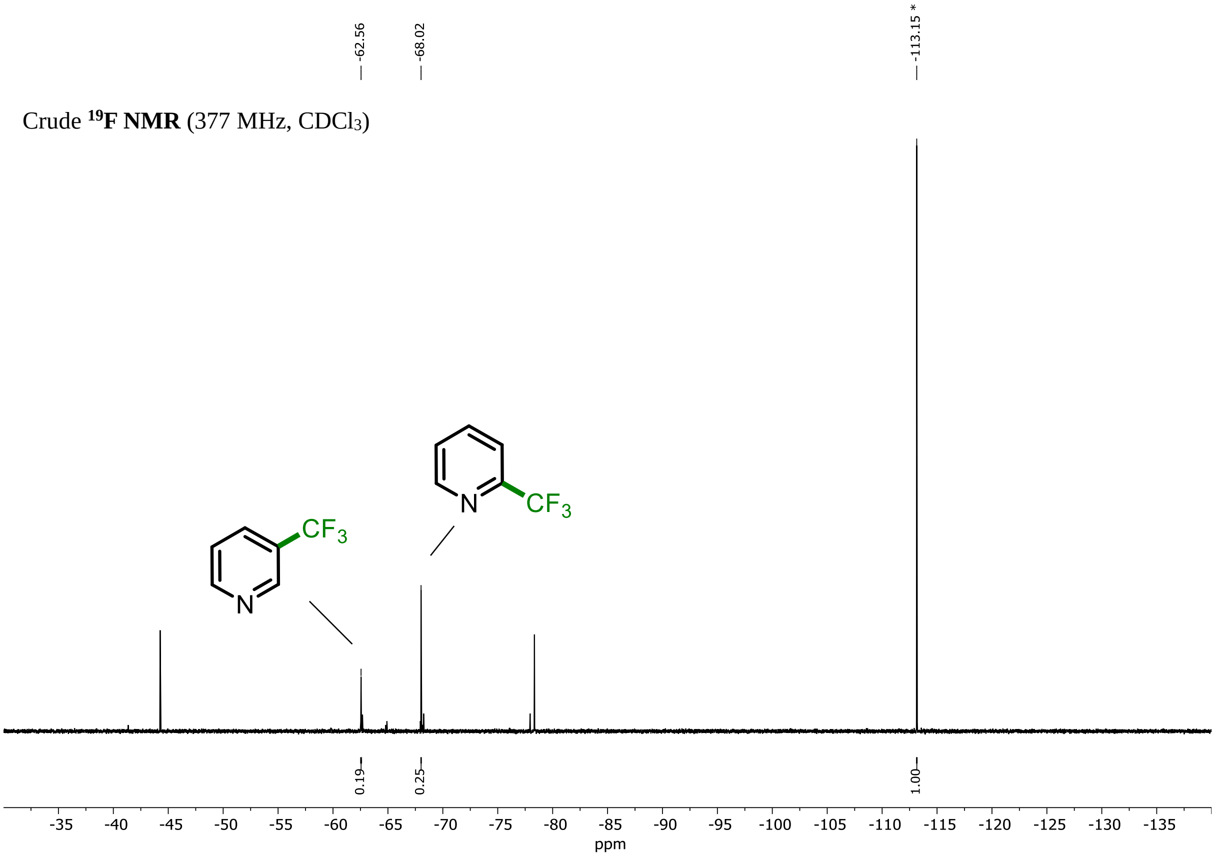

3-(trifluoromethyl)isonicotinonitrile 1i

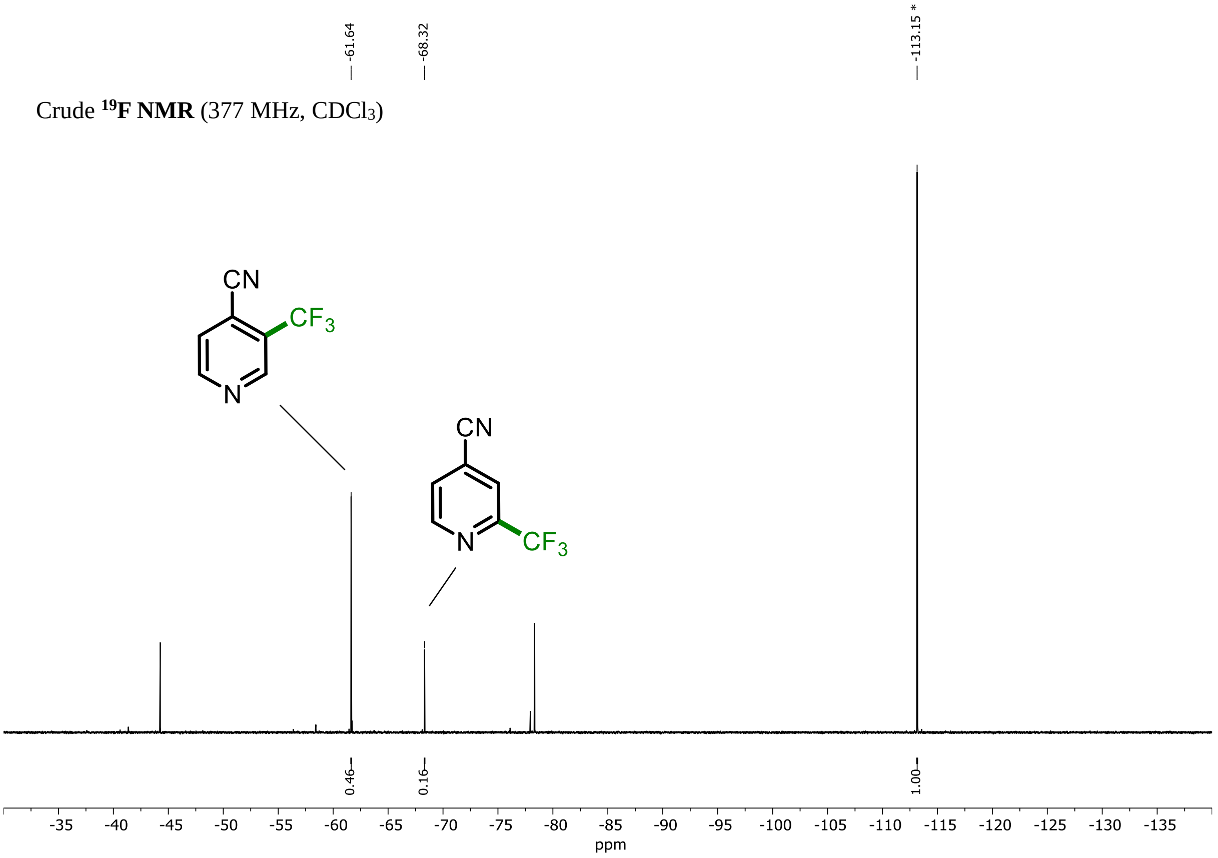

2,4,6-trimethoxy-5-(trifluoromethyl)pyrimidine 1j

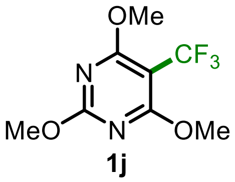

Crude ^{19}**F NMR** (377 MHz, $CDCl_3$)

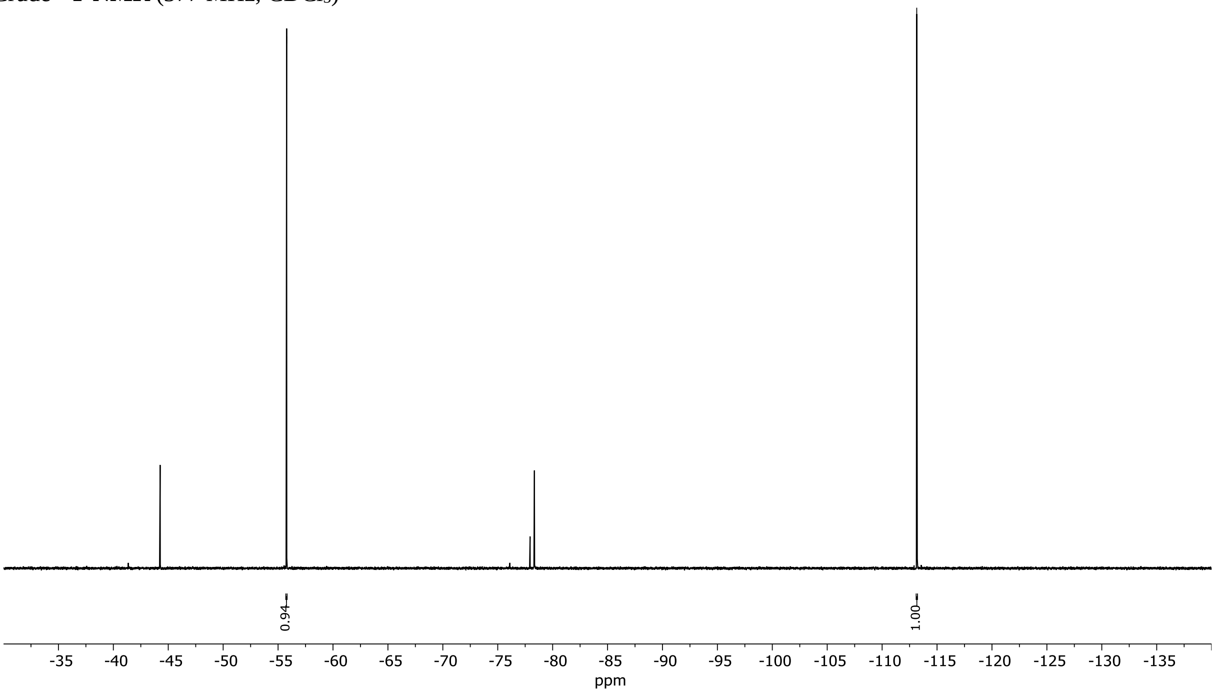

1,3-dimethyl-5-(trifluoromethyl)pyrimidine-2,4(1H,3H)-dione 1k

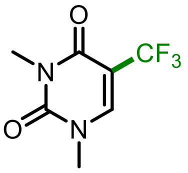

Crude ^{19}**F NMR** (377 MHz, $CDCl_3$)

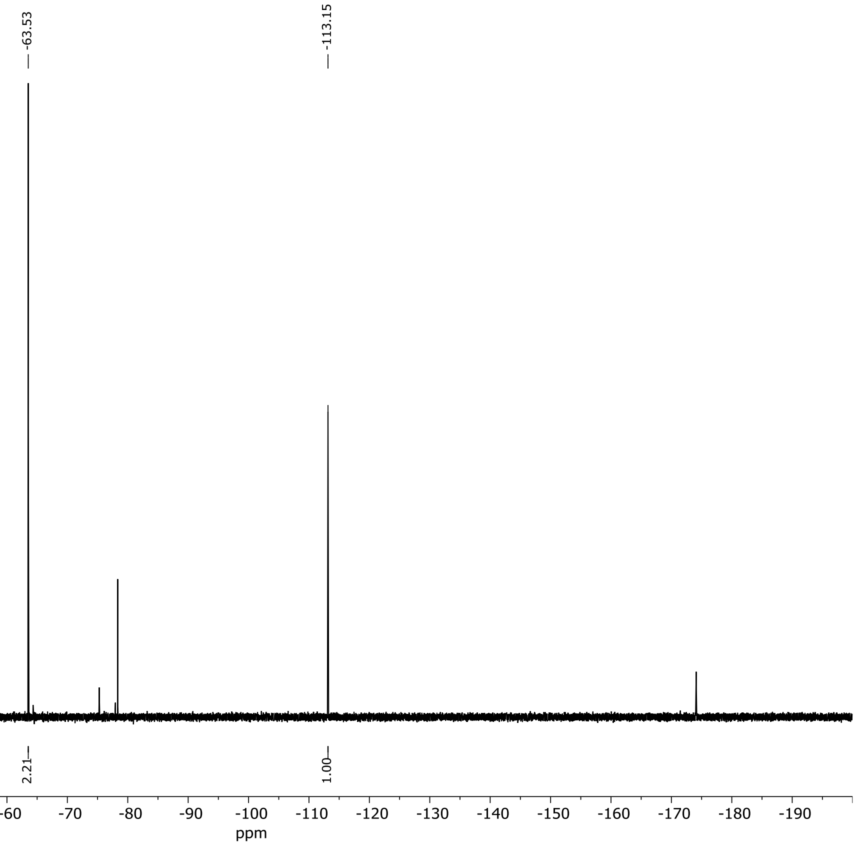

8-(trifluoromethyl)-9H-purin-6-amine 1l

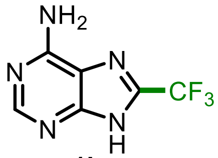

1l

Crude **^{19}F NMR** (377 MHz, $CDCl_3$)

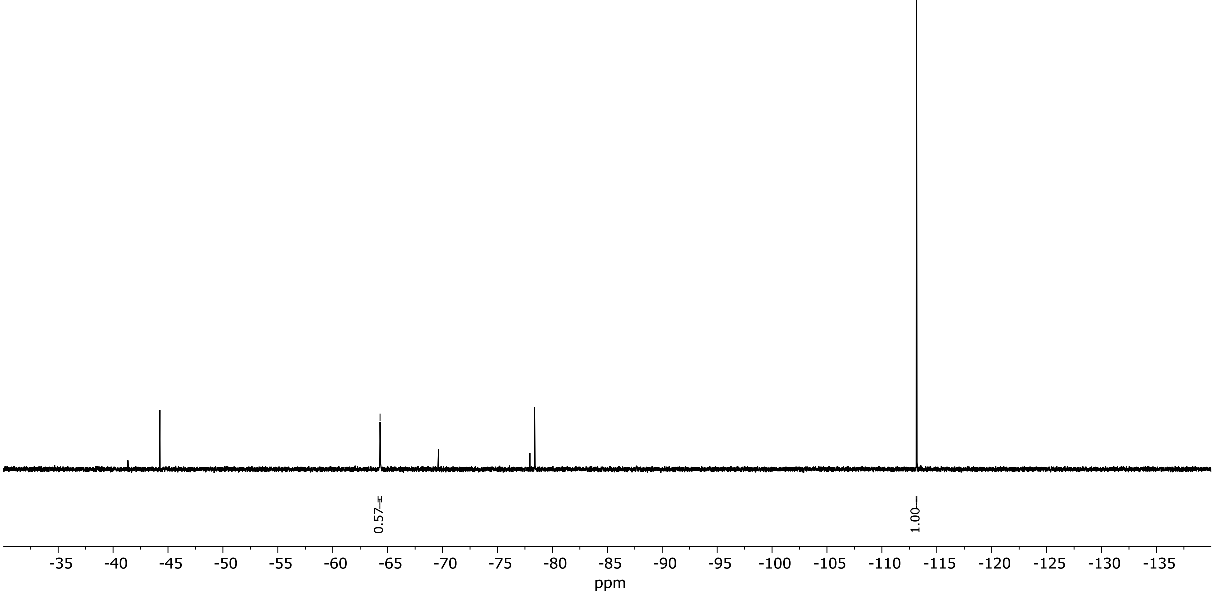

1,3,7-trimethyl-8-(trifluoromethyl)-3,7-dihydro-1H-purine-2,6-dione 1m

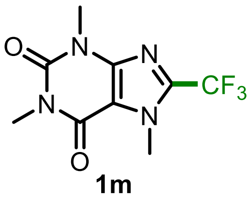

1m

Crude [19]**F NMR** (377 MHz, $CDCl_3$)

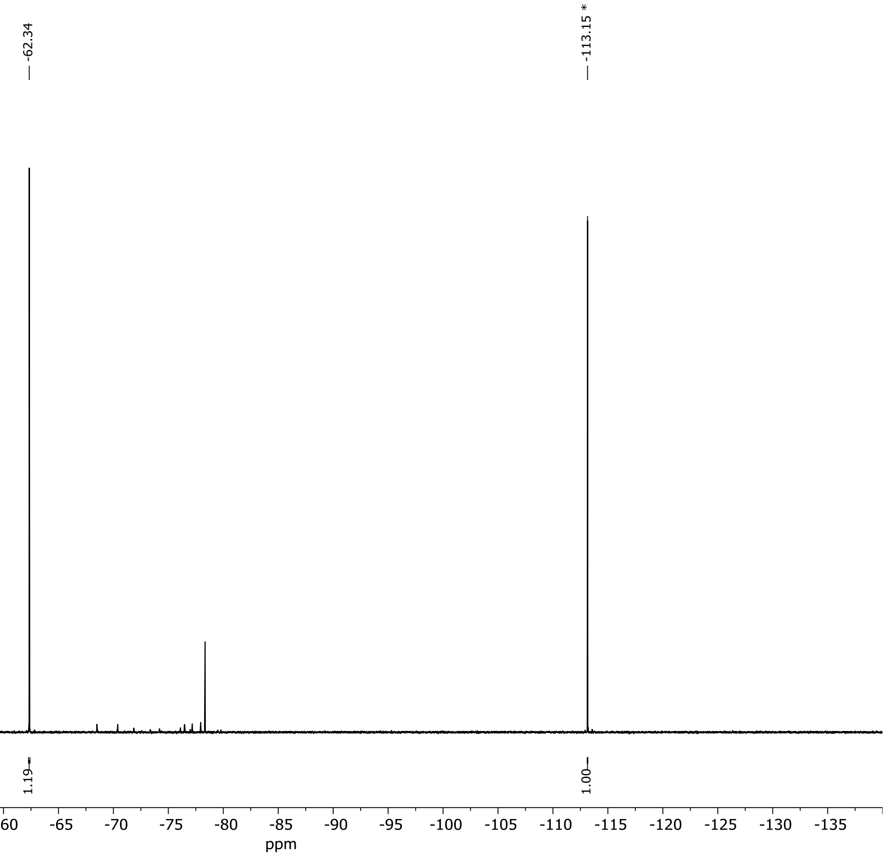

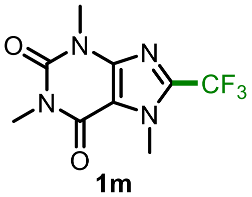

1m

[1]**H NMR** (400 MHz, $CDCl_3$)

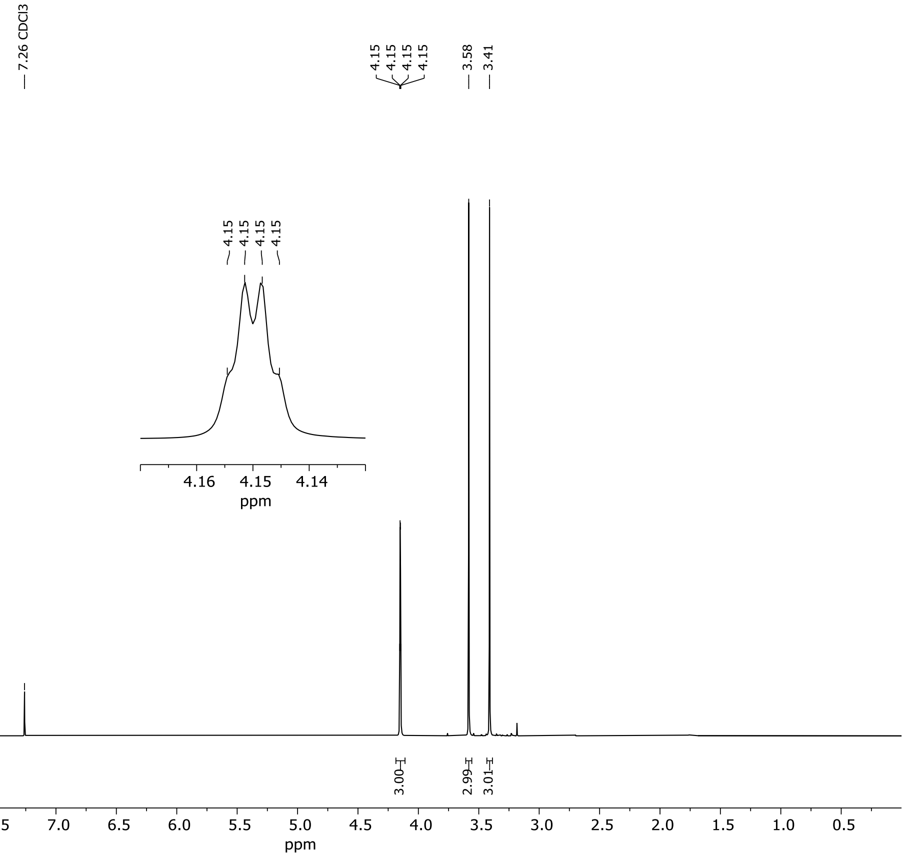

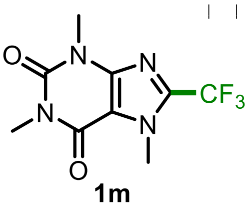

1m

^{13}C NMR (101 MHz, $CDCl_3$)

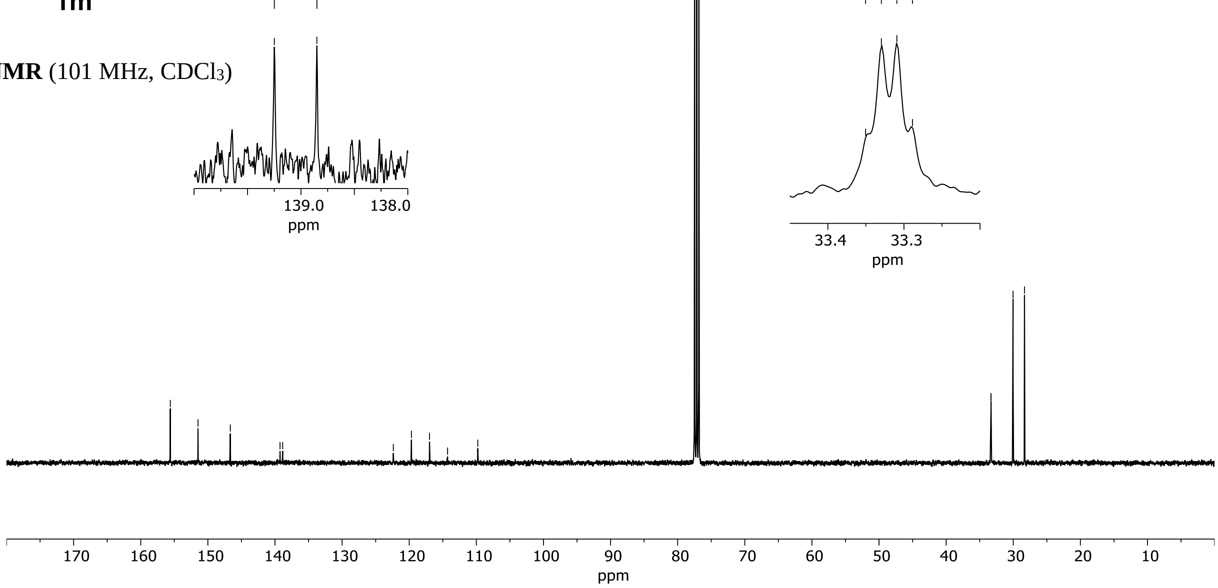

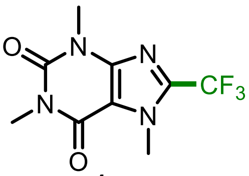

1m

^{19}F NMR (377 MHz, $CDCl_3$)

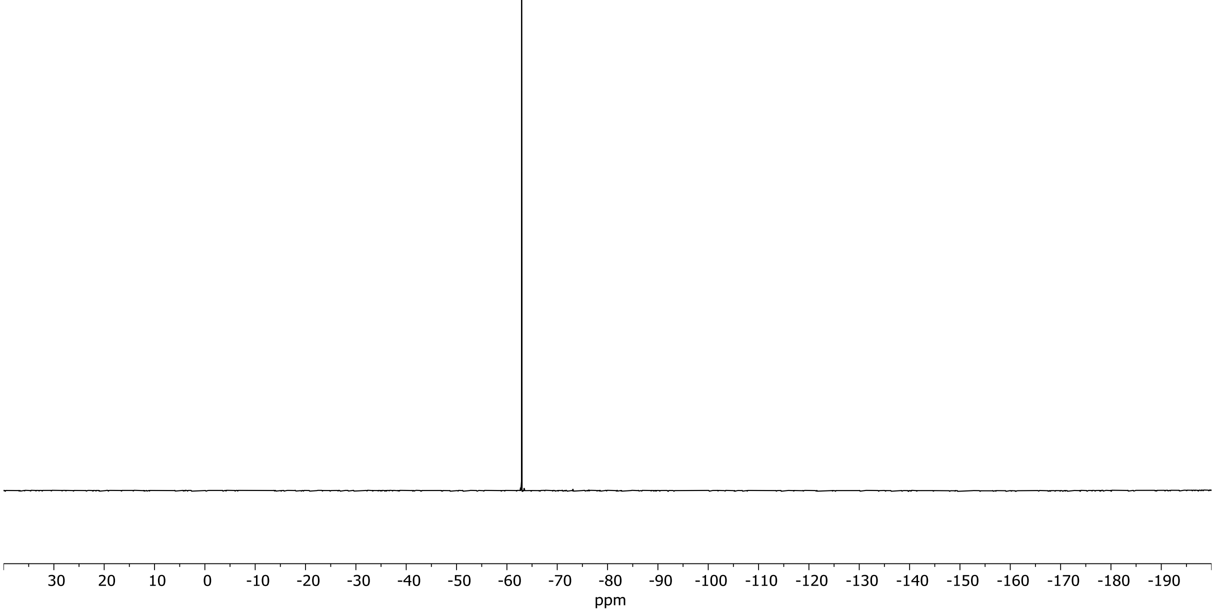

2-(difluoromethyl)-1,3,5-trimethoxybenzene 1n

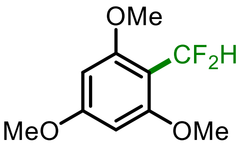

1n

Crude [19]**F NMR** (377 MHz, CDCl3)

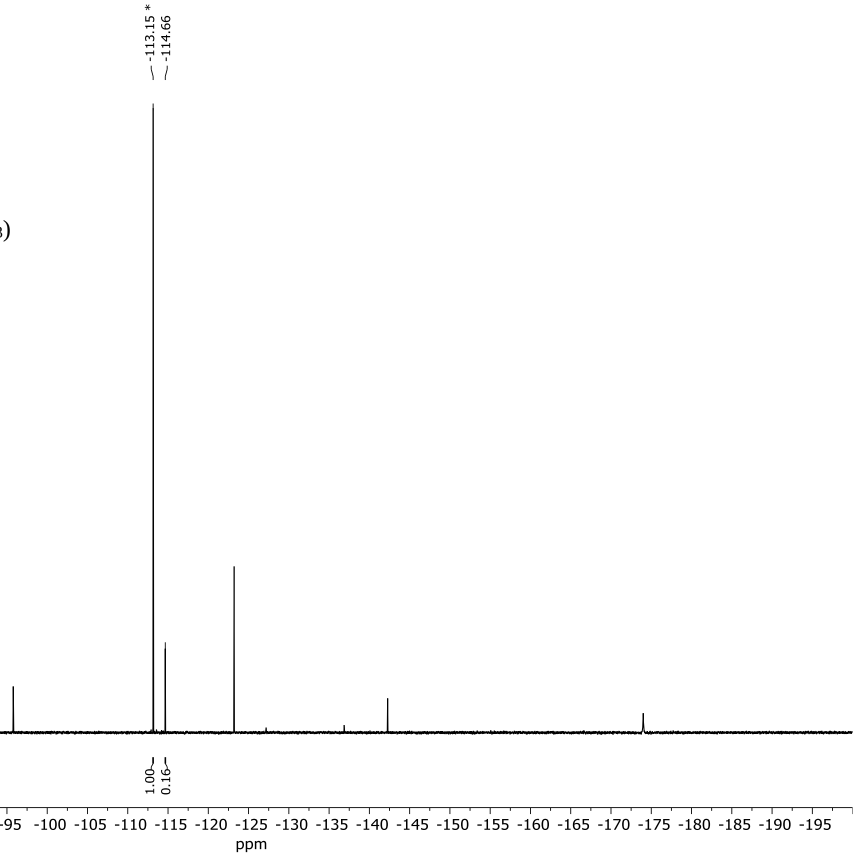

5-(difluoromethyl)-1,3-dimethylpyrimidine-2,4(1H,3H)-dione 1o

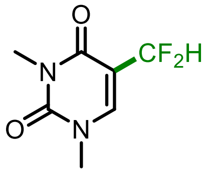

1o

Crude [19]**F NMR** (377 MHz, CDCl3)

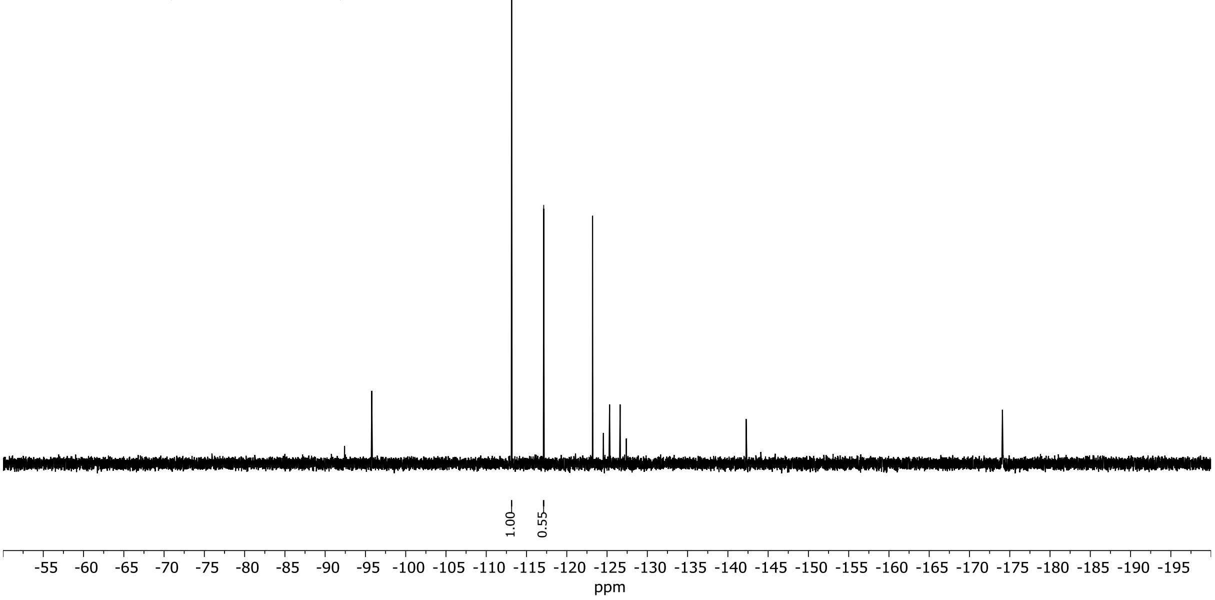

8-(difluoromethyl)-1,3,7-trimethyl-3,7-dihydro-1H-purine-2,6-dione 1p

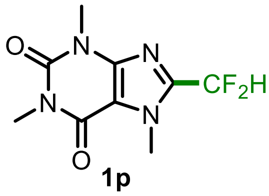

1p

Crude [19]**F NMR** (377 MHz, $CDCl_3$)

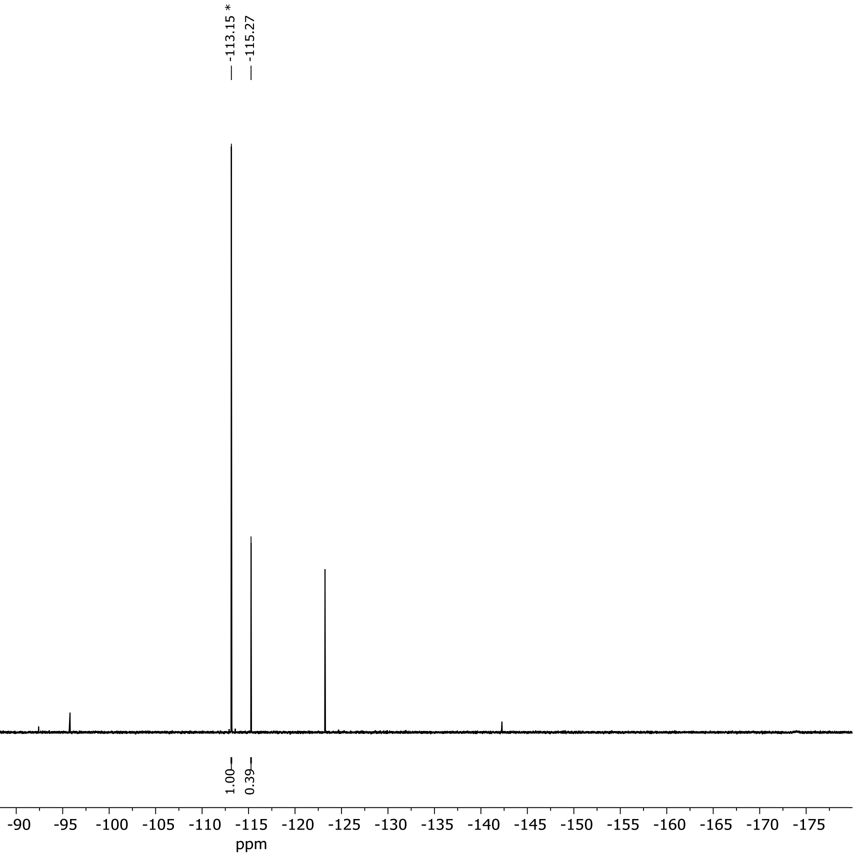

2-bromo-1,3,5-trimethoxybenzene 2a

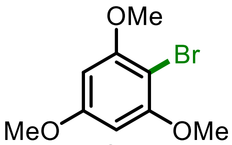

2a

^{1}H NMR (400 MHz, $CDCl_3$)

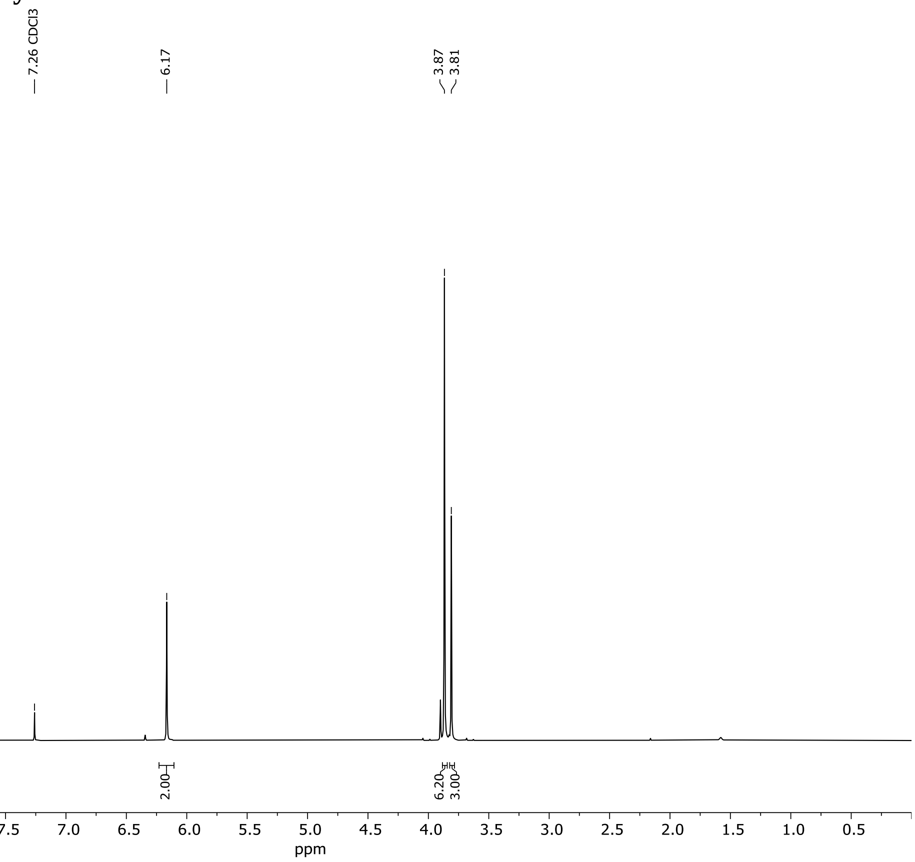

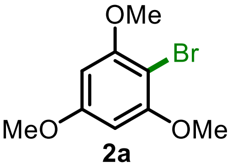

2a

^{13}C NMR (101 MHz, $CDCl_3$)

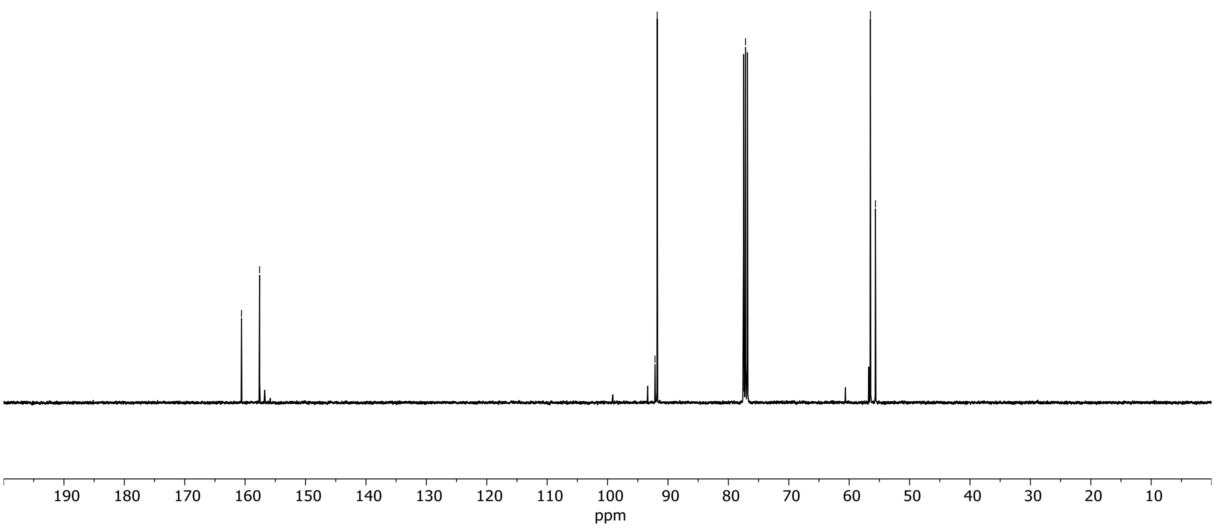

1-bromo-4-methoxybenzene 2b

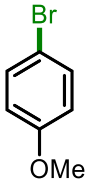

2b

^{1}H NMR (400 MHz, $CDCl_3$)

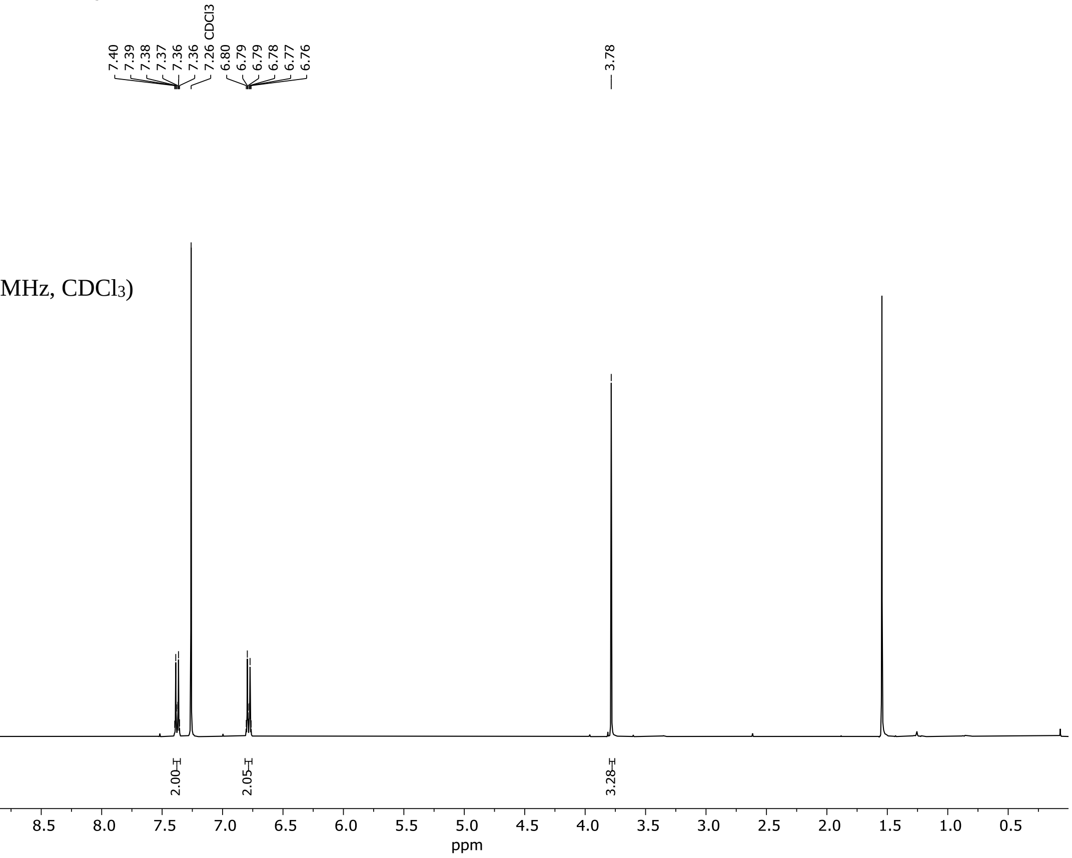

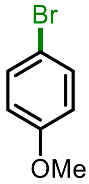

2b

^{13}C NMR (101 MHz, $CDCl_3$)

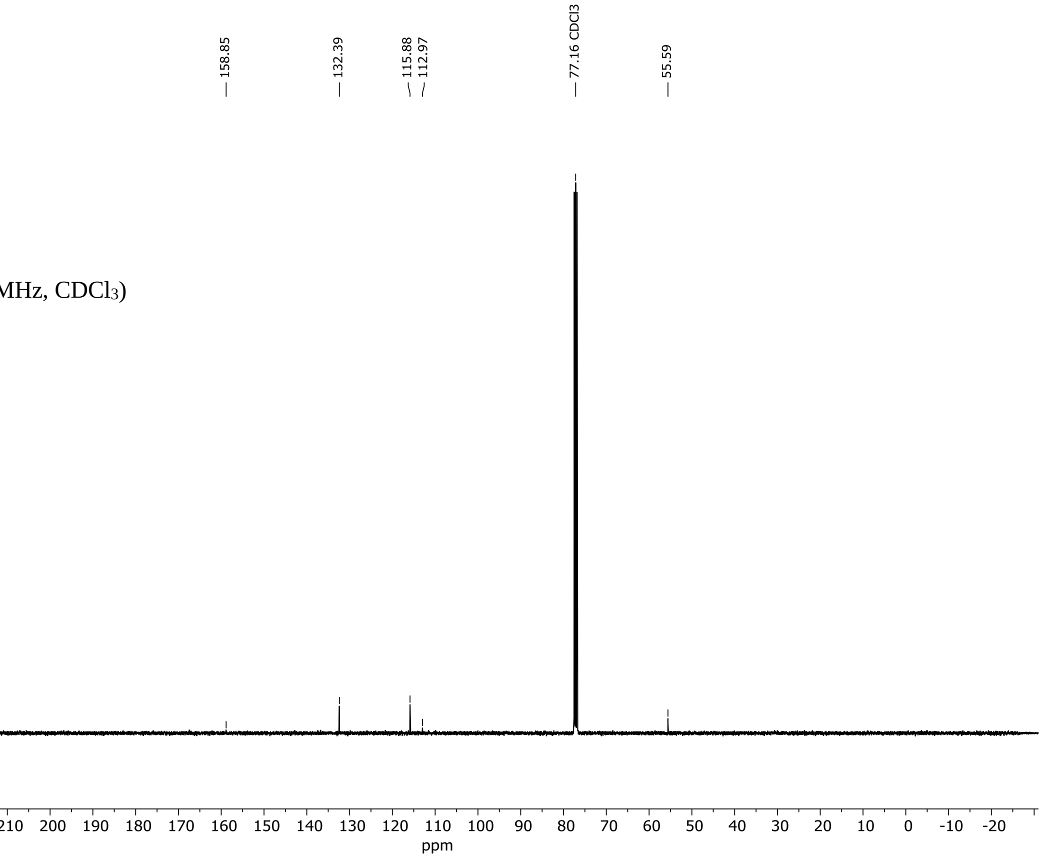

1,3,5-trimethoxy-2-thiocyanatobenzene 2c

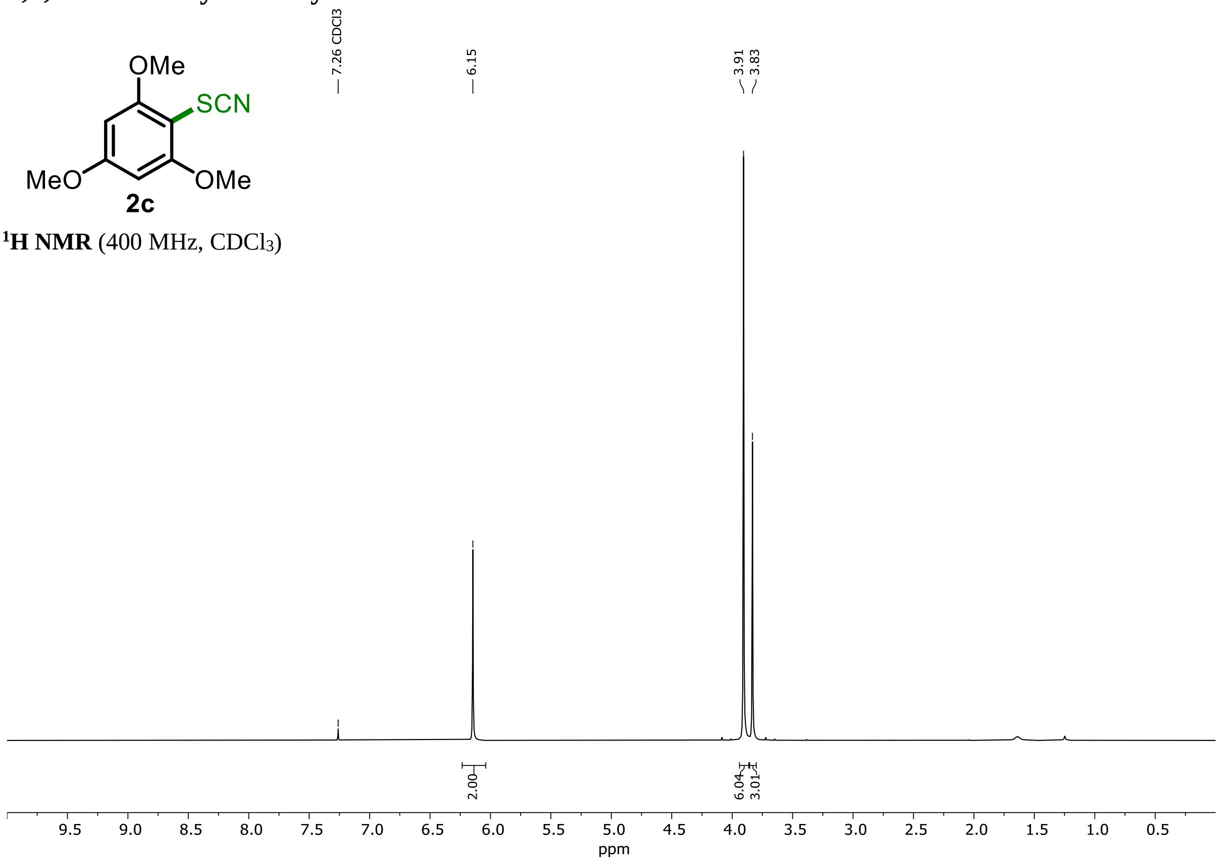

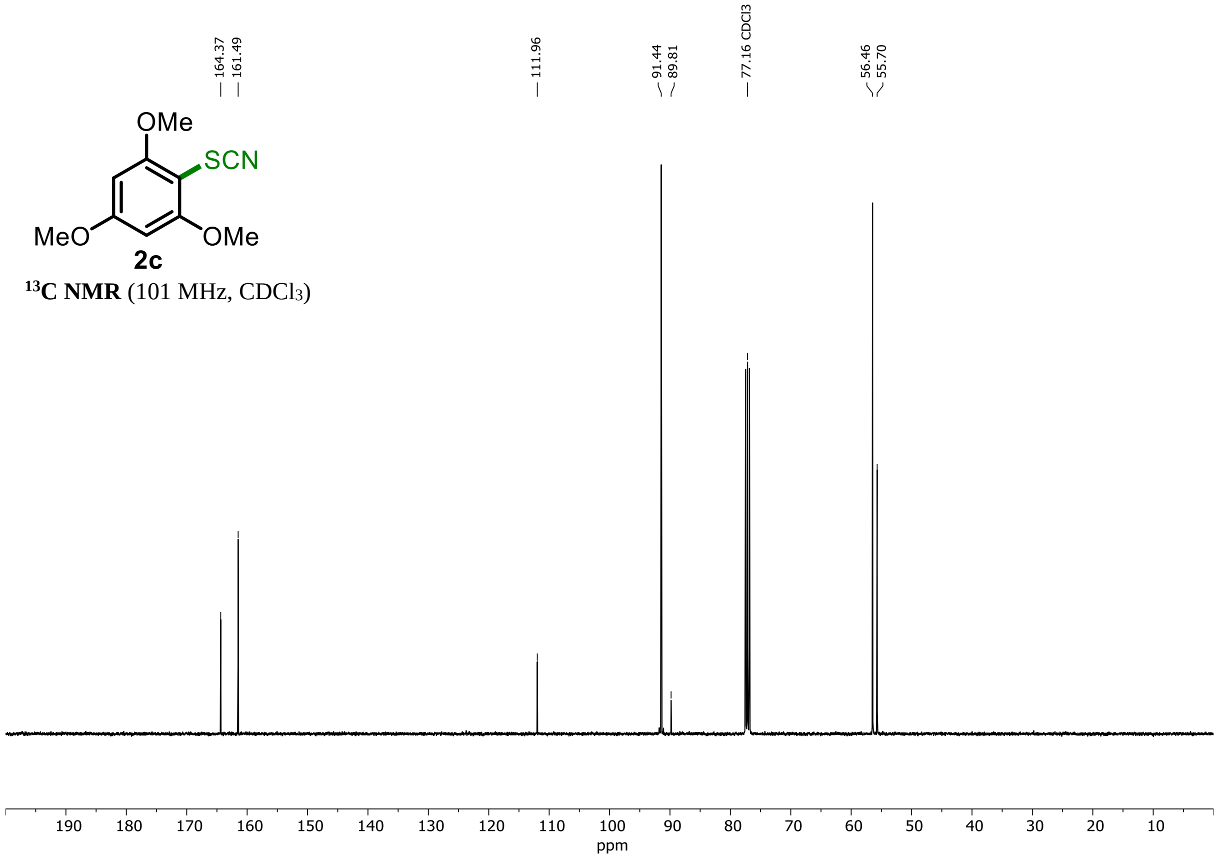

2,4-dimethoxy-1-thiocyanatobenzene 2d

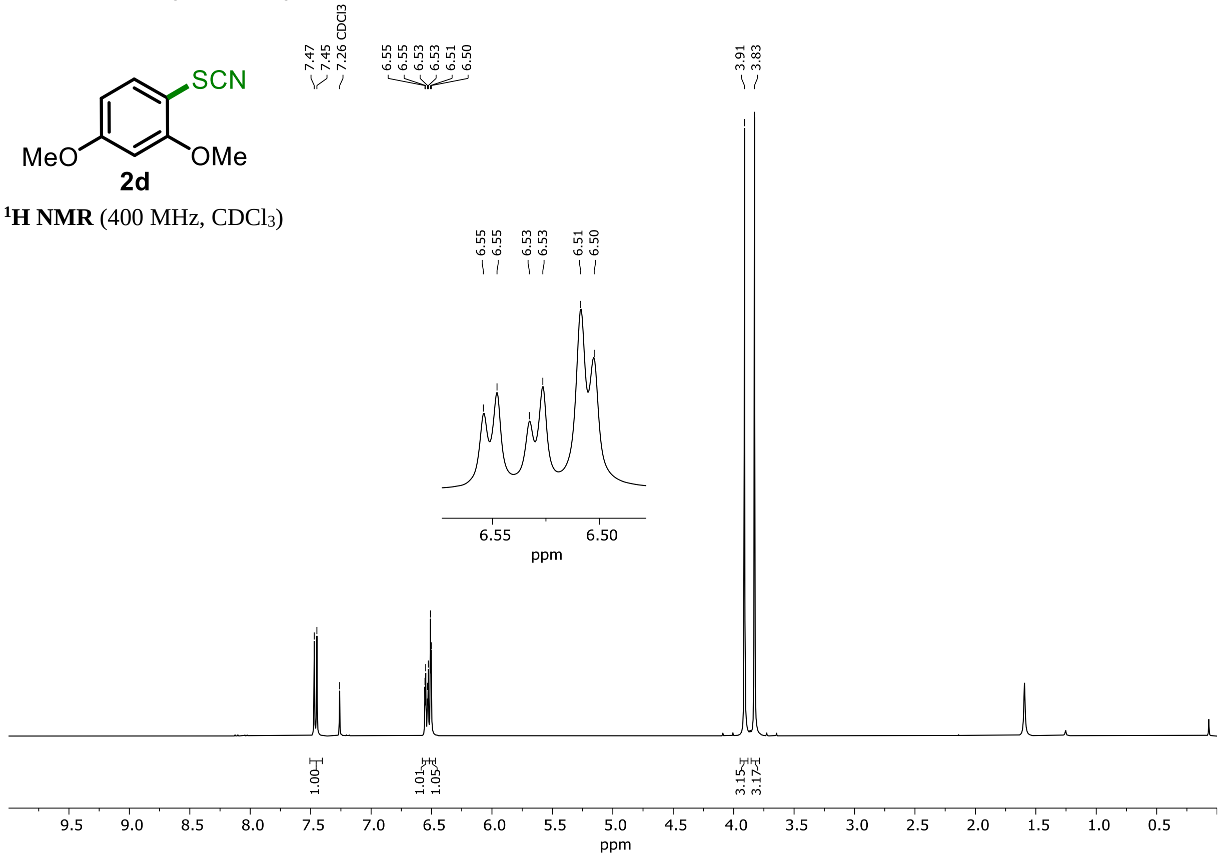

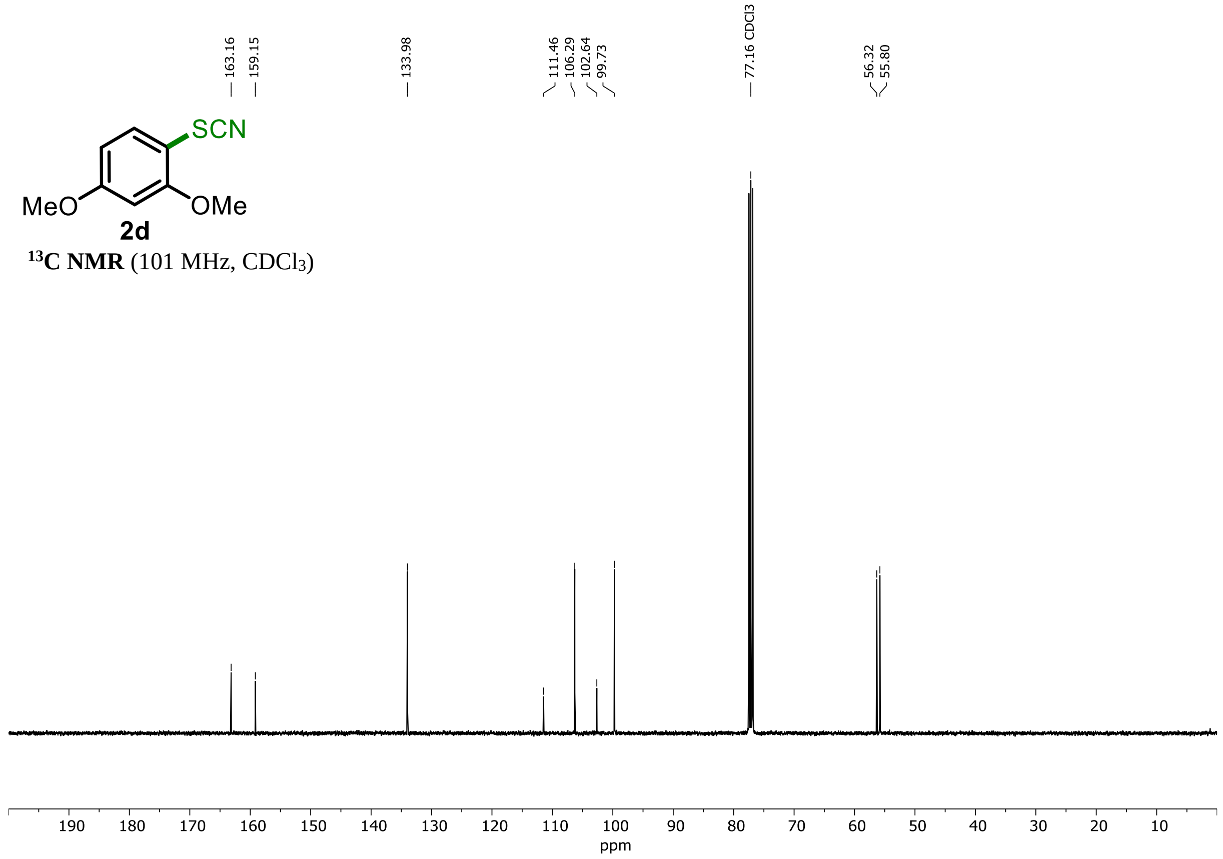

2,4,6-trimethoxybenzonitrile 2e

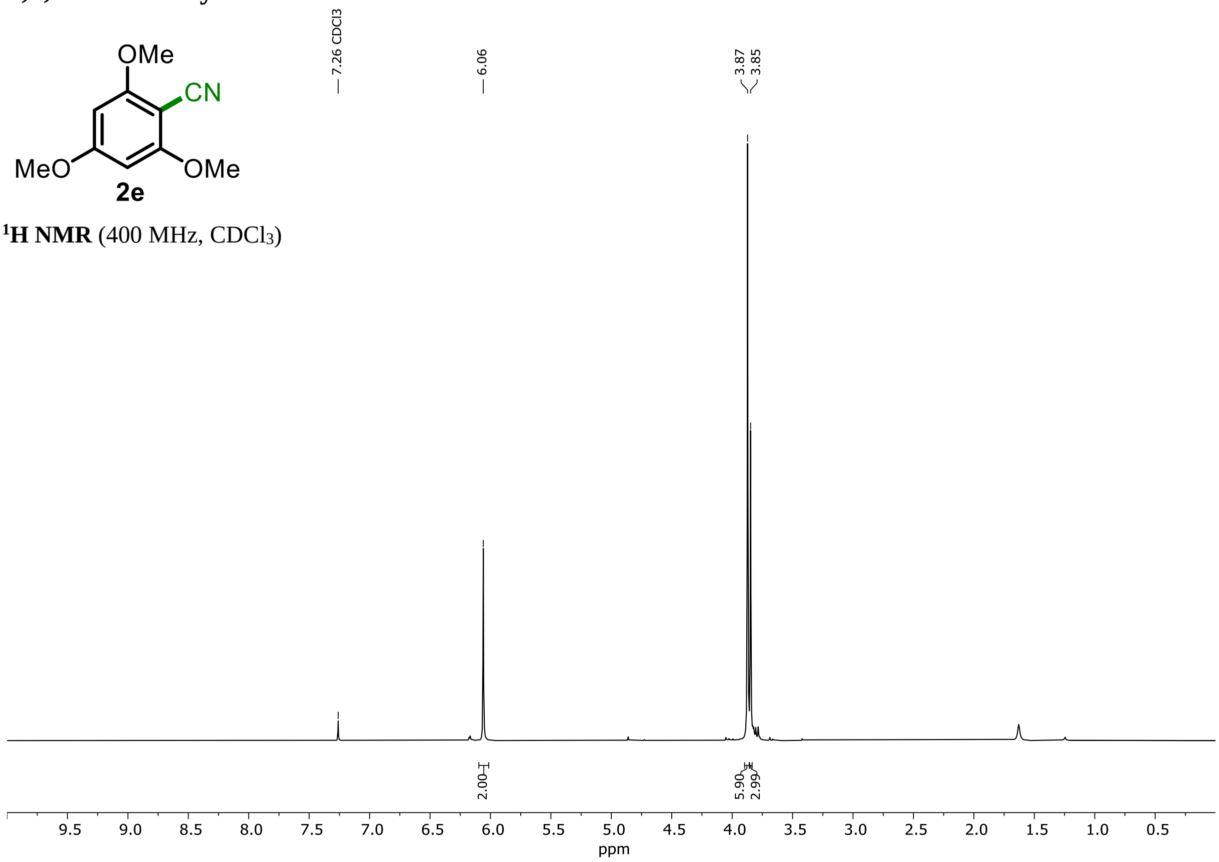

2e

^{1}H NMR (400 MHz, $CDCl_3$)

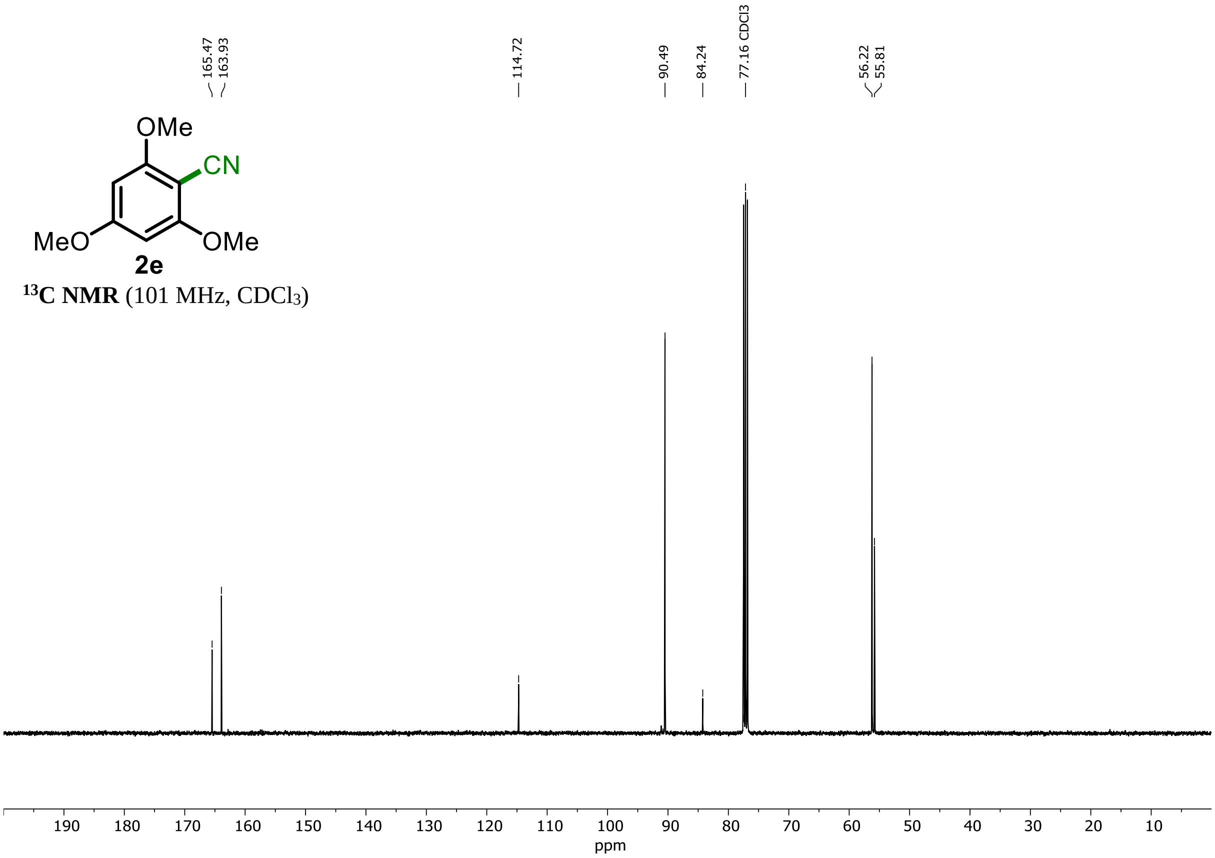

2e

^{13}C NMR (101 MHz, $CDCl_3$)

Diethyl (2,4,6-trimethoxyphenyl)phosphonate 2f

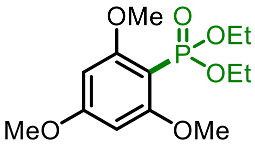

2f

Crude **^{31}P NMR** (162 MHz, $CDCl_3$), **NQH$_2$-1**

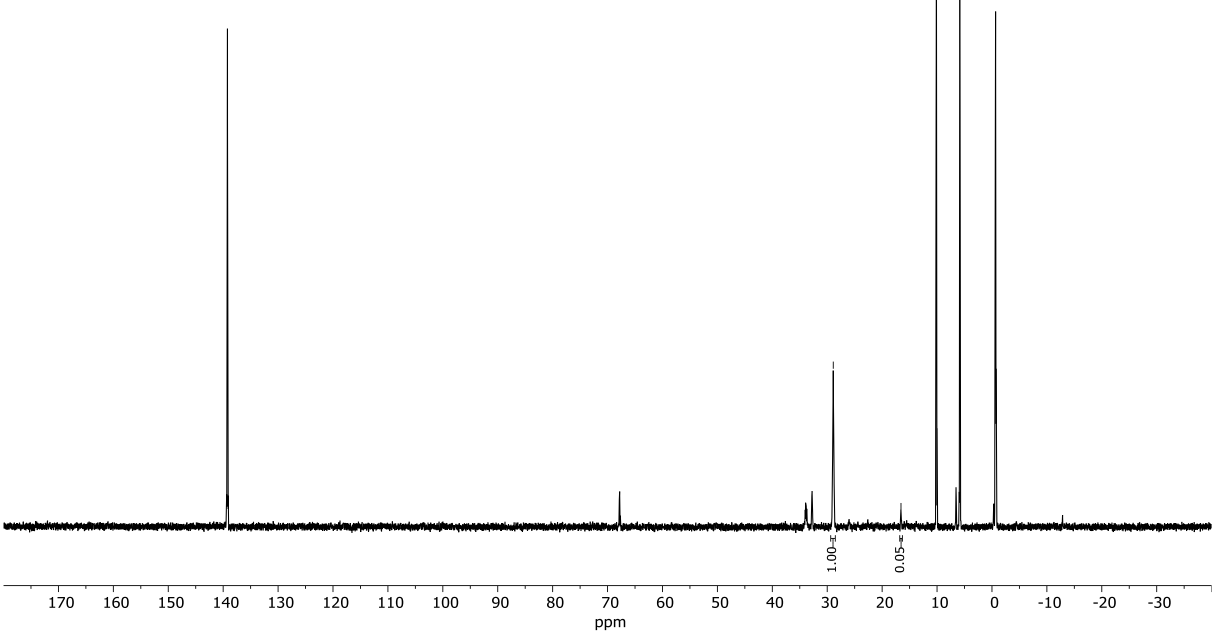

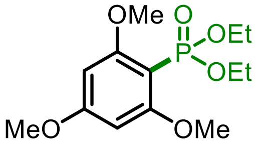

2f

Crude **^{31}P NMR** (162 MHz, $CDCl_3$), **2-SAS**

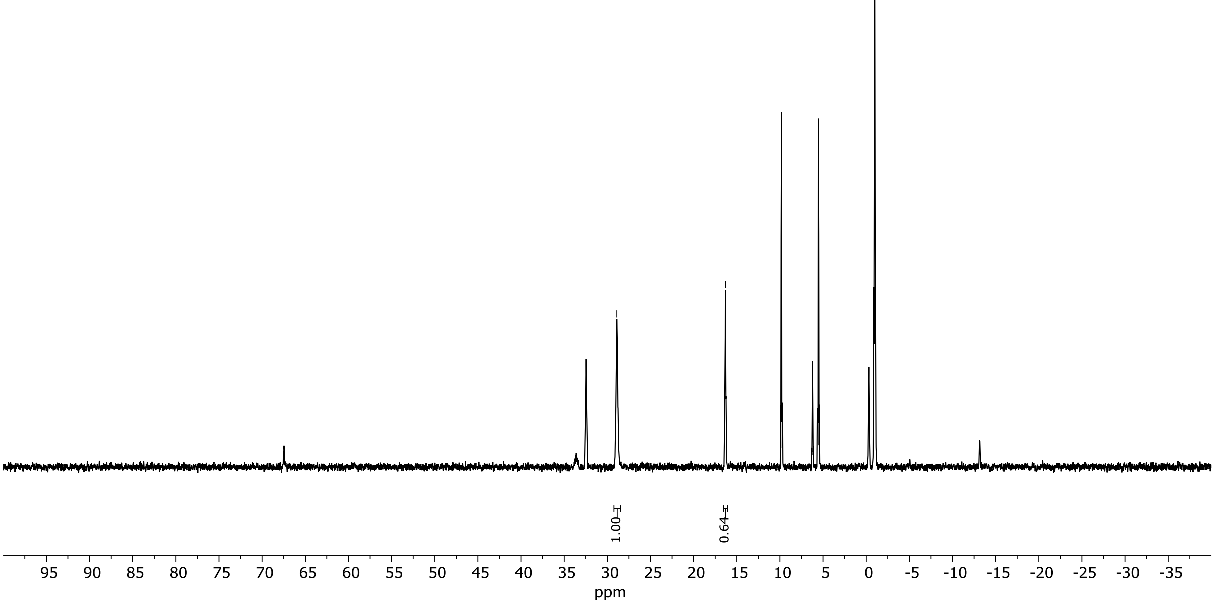

Diethyl (2,4-dimethoxyphenyl)phosphonate and diethyl (2,6-dimethoxyphenyl)phosphonate) 2g

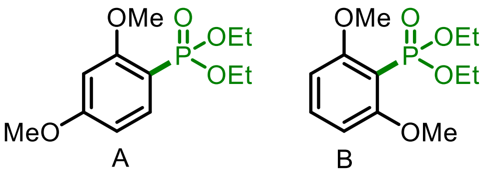

Crude ^{31}**P NMR** (162 MHz, $CDCl_3$), **NQH$_2$-1**

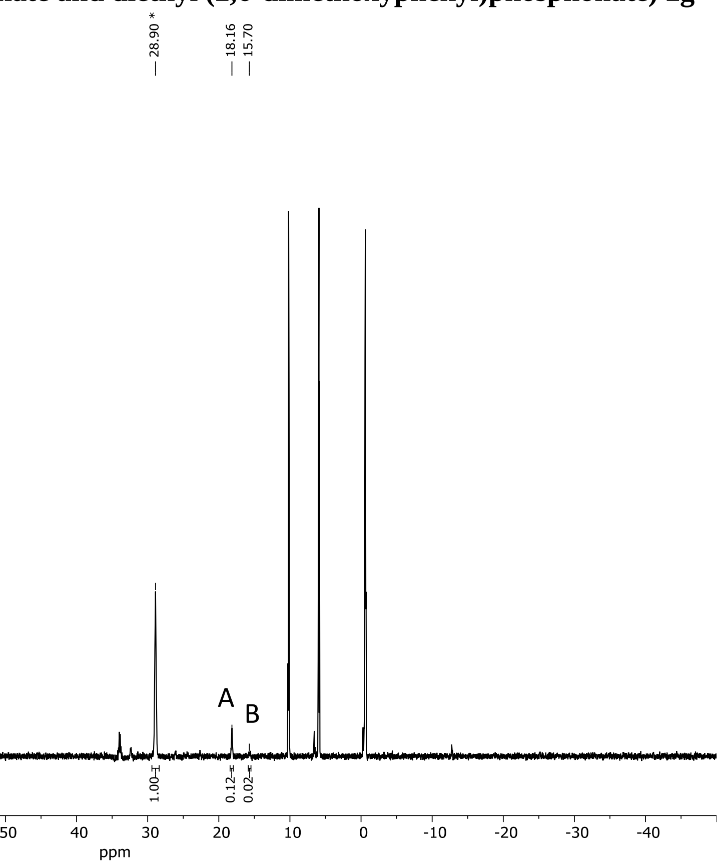

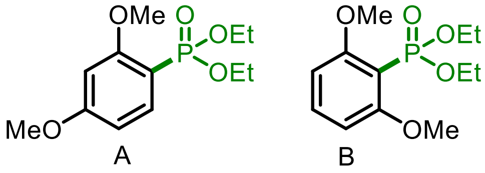

Crude ^{31}**P NMR** (162 MHz, $CDCl_3$), **2-SAS**

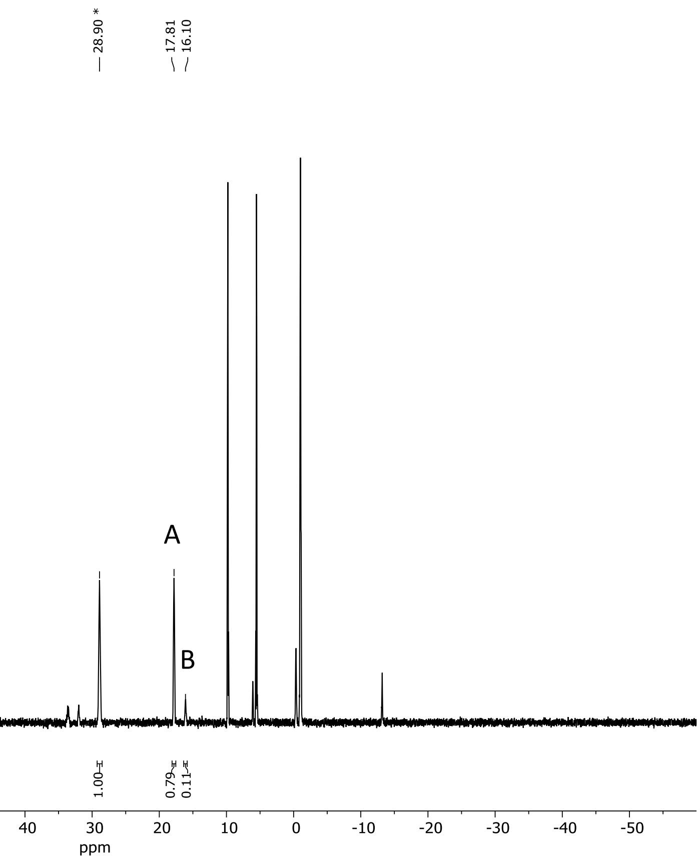

Diethyl (2-methoxyphenyl)phosphonate 2h

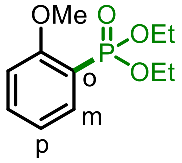

Crude ^{31}**P NMR** (162 MHz, $CDCl_3$), **2-SAS**

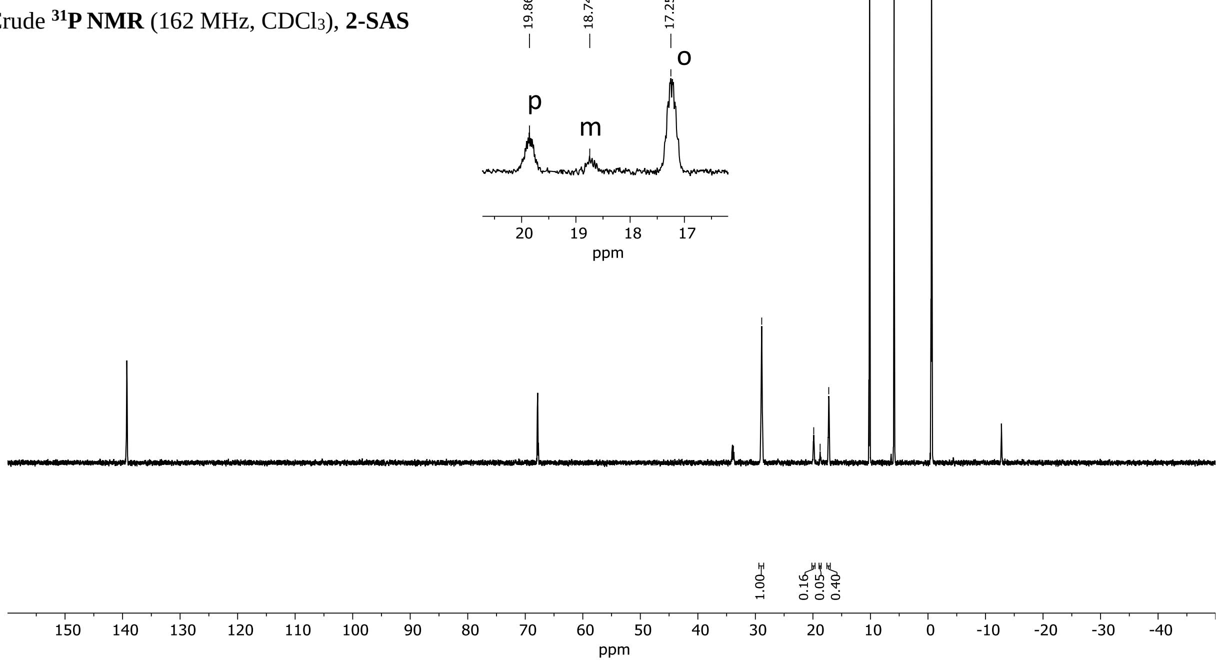

1-(2,4,6-trimethoxyphenyl)-1H-pyrazole 2i

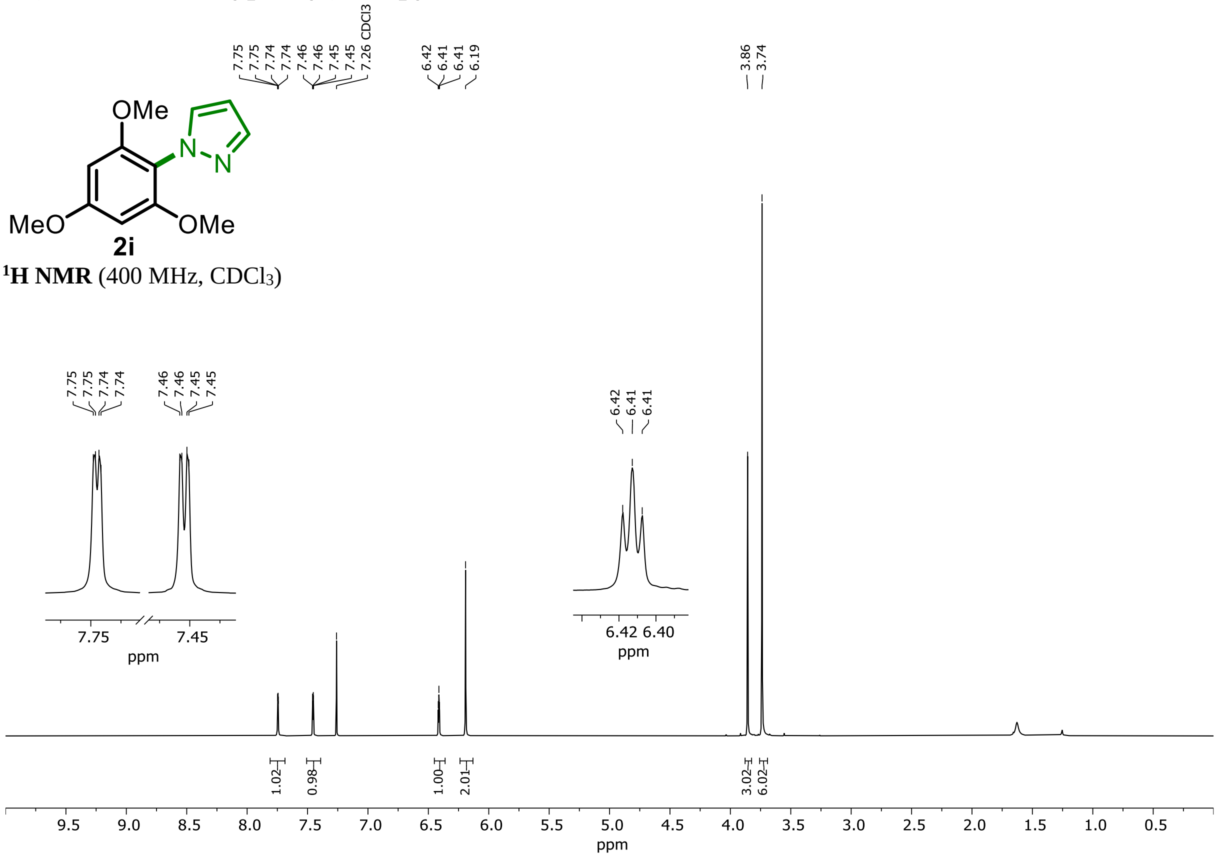

^{1}**H NMR** (400 MHz, $CDCl_3$)

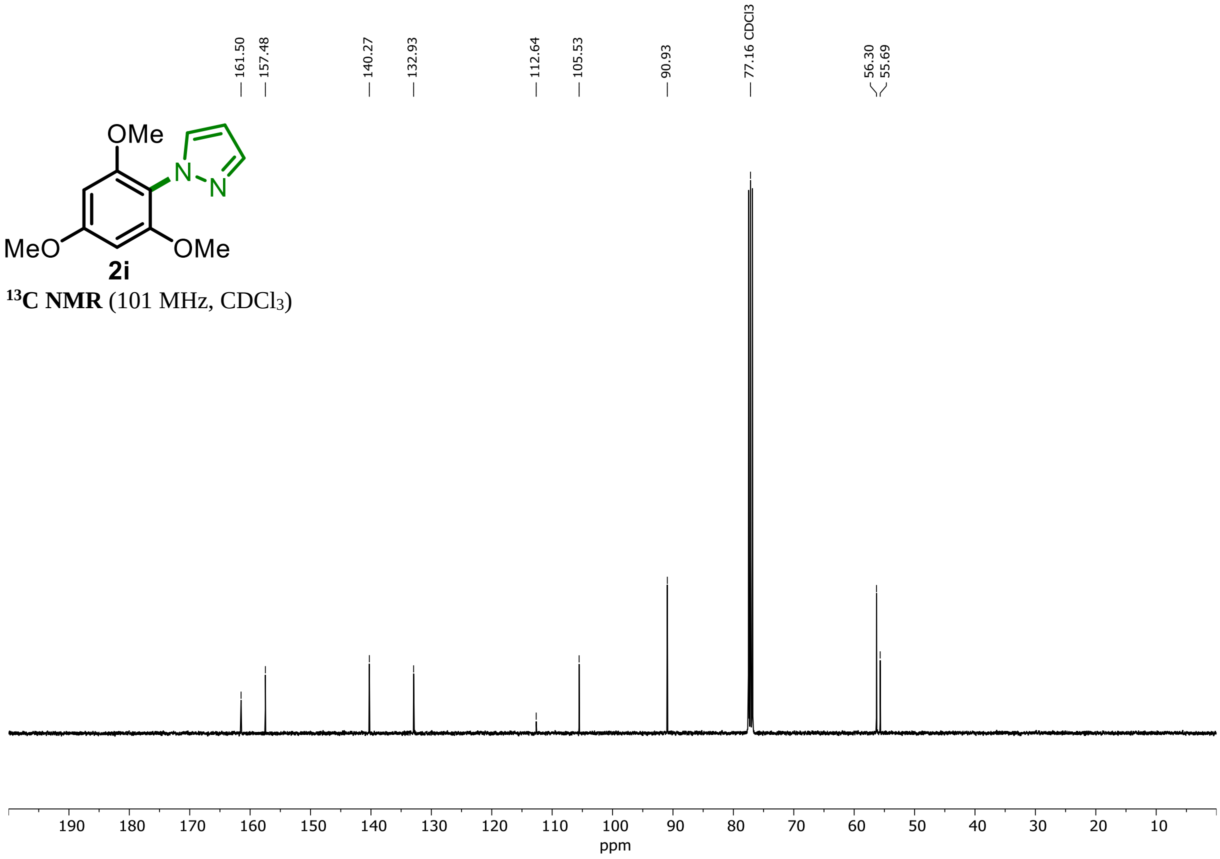

^{13}**C NMR** (101 MHz, $CDCl_3$)

1-(naphthalen-2-yl)-1H-pyrazole 2j

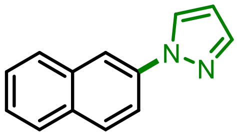

2j

^{1}H NMR (400 MHz, $CDCl_3$)

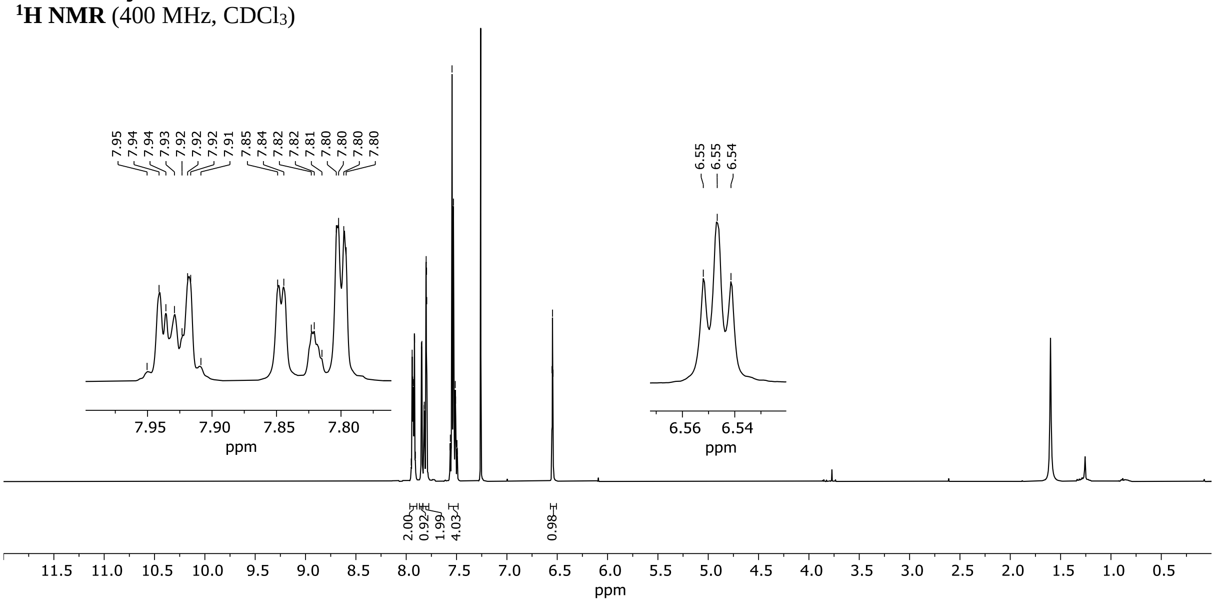

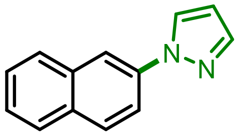

2j

^{13}C NMR (101 MHz, $CDCl_3$)

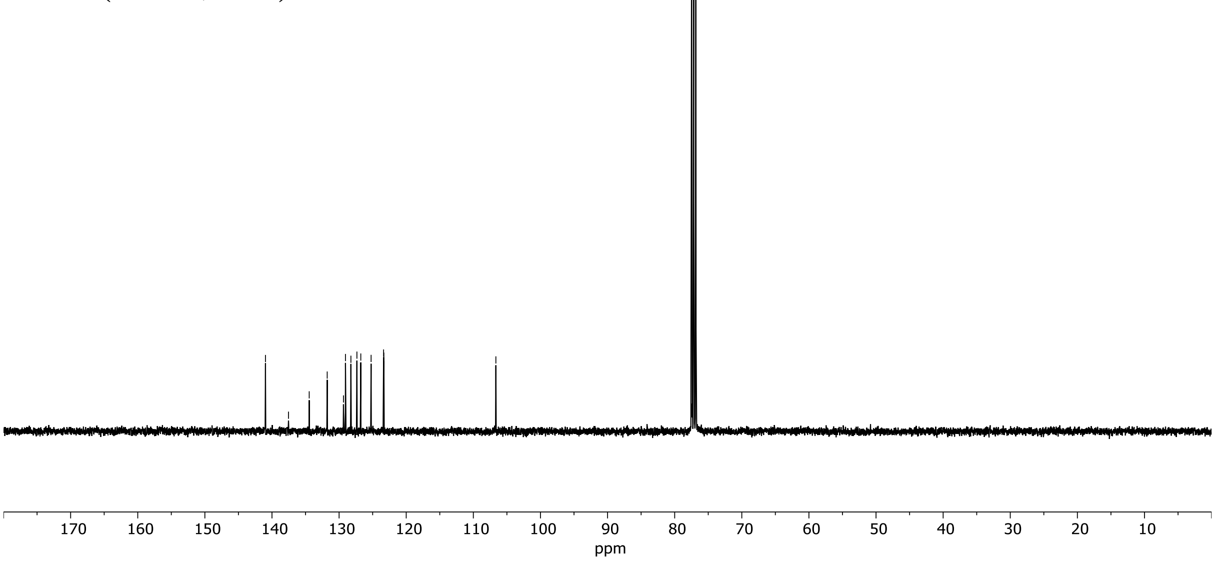

1-(2,4,6-trimethoxyphenyl)-1H-imidazole 2k

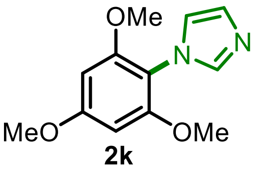

2k

^{1}H NMR (400 MHz, $CDCl_3$)

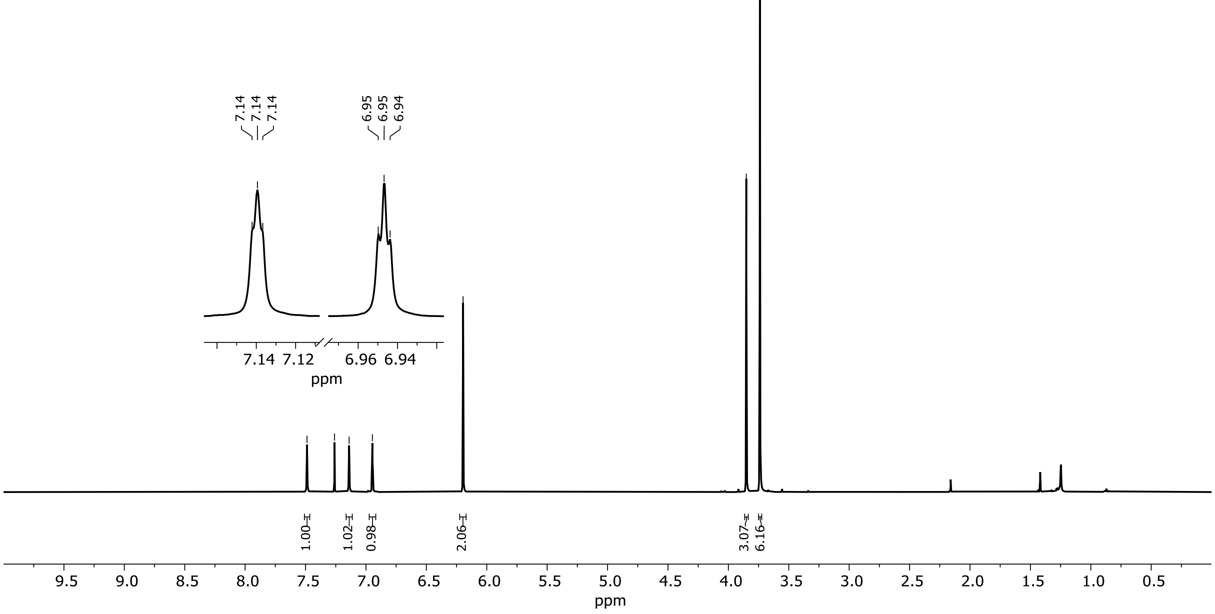

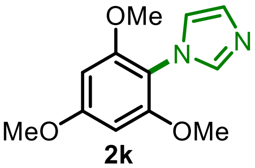

2k

^{13}C NMR (101 MHz, $CDCl_3$)

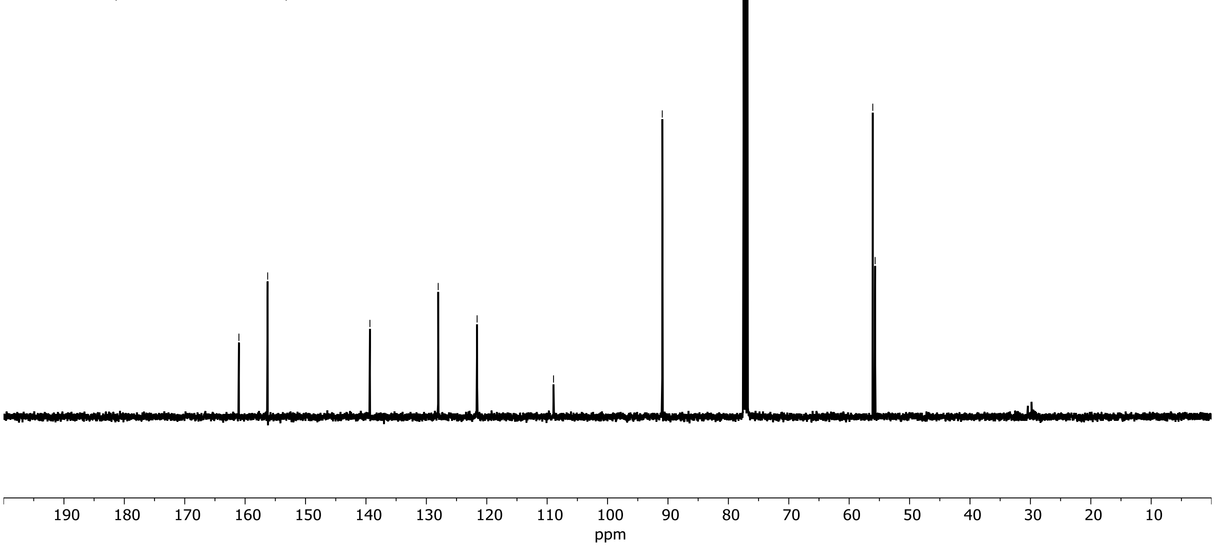

Acetophenone 3a

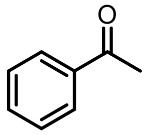

Crude **¹H NMR** (300 MHz, $CDCl_3$)
synthesis from 1-phenylethanol

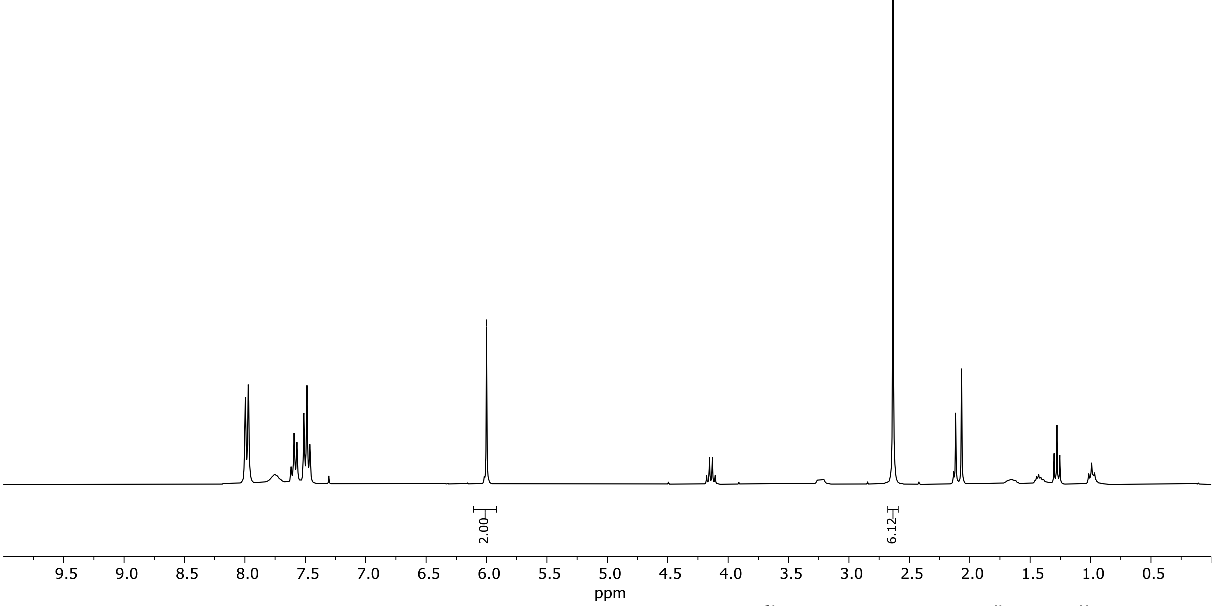

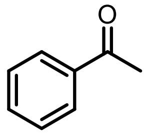

Crude **¹H NMR** (400 MHz, $CDCl_3$)
synthesis from ethylbenzene

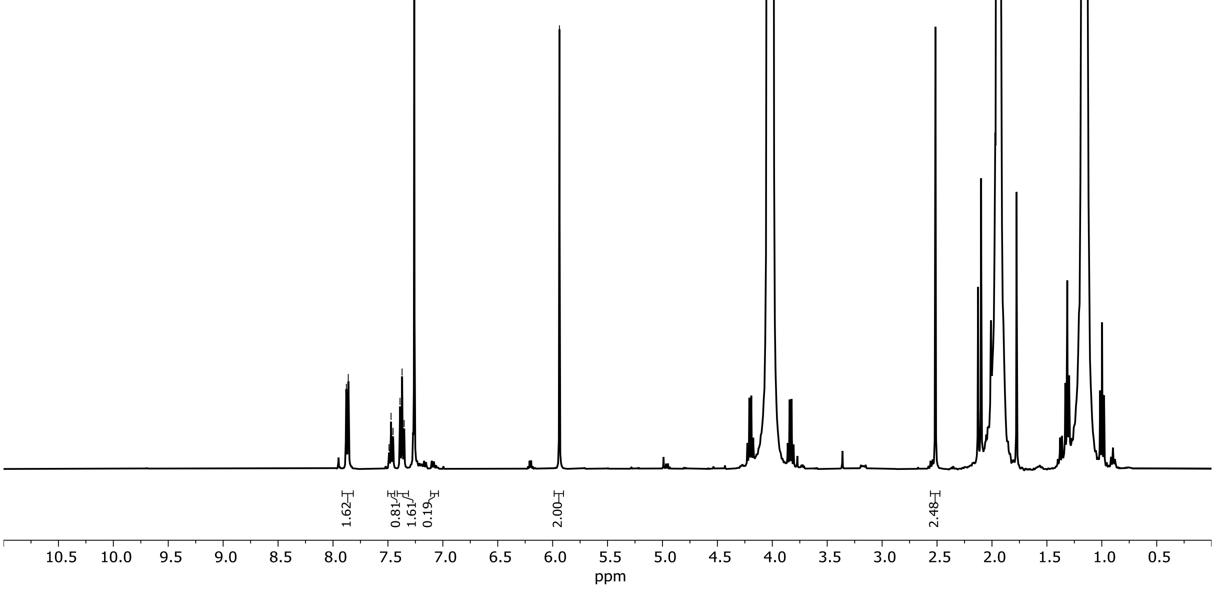

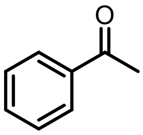

3a

^{1}H NMR (300 MHz, $CDCl_3$)

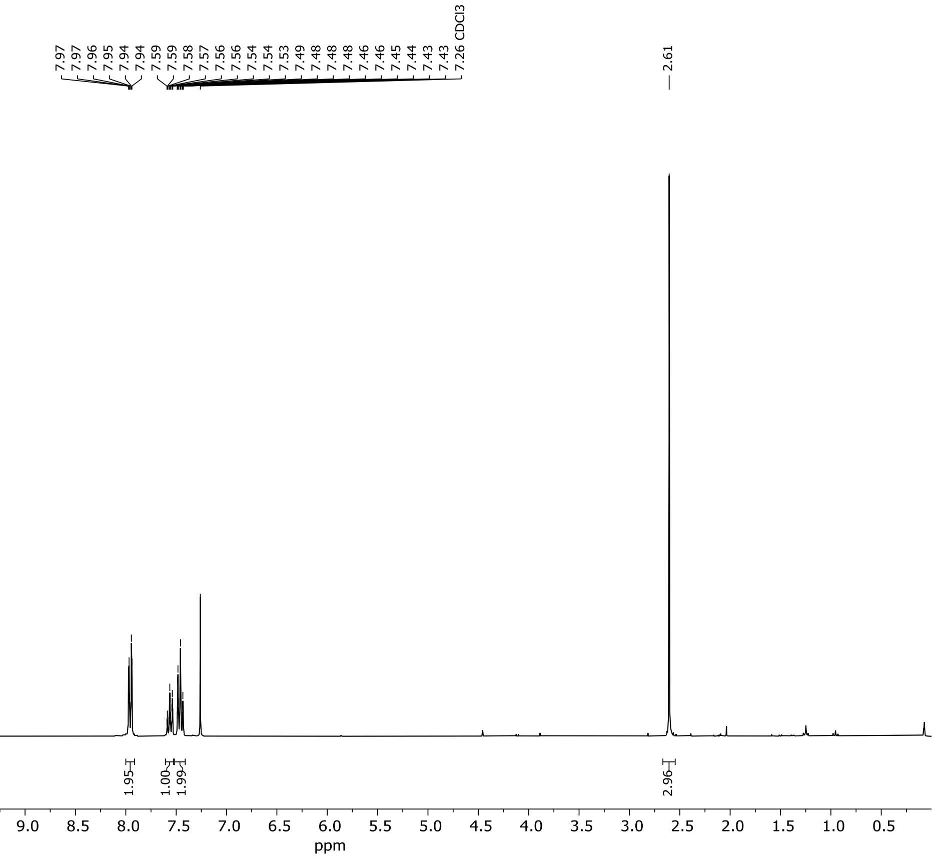

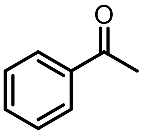

3a

^{13}C NMR (101 MHz, $CDCl_3$)

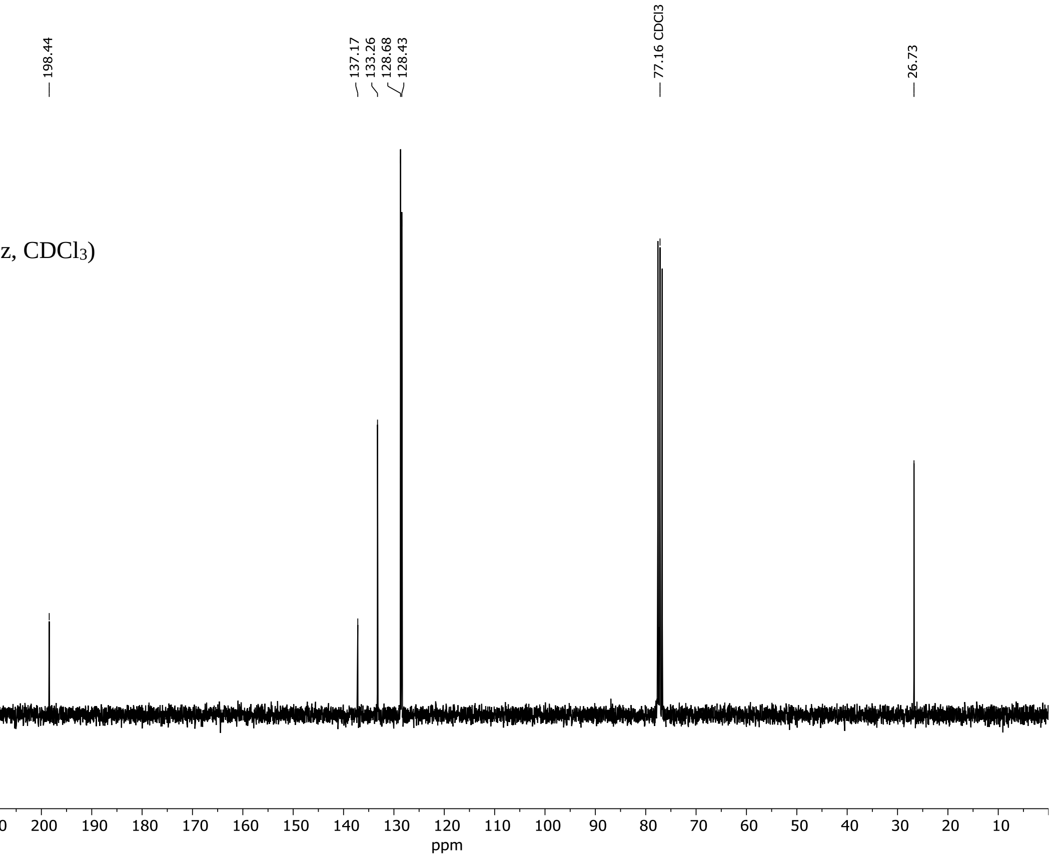

Cyclohexanone 3b

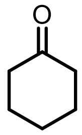

Crude ¹H NMR (400 MHz, $CDCl_3$)
Synthesis from cyclohexanol

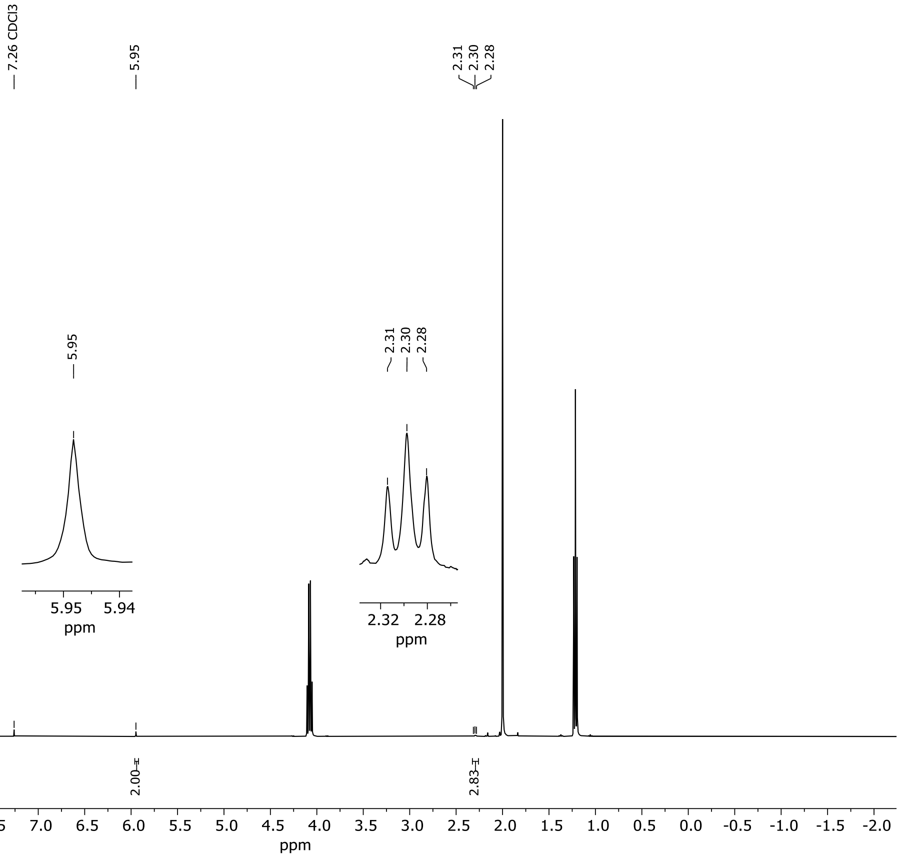

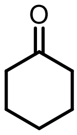

Crude ¹H NMR (400 MHz, $CDCl_3$)
Synthesis from cyclohexane

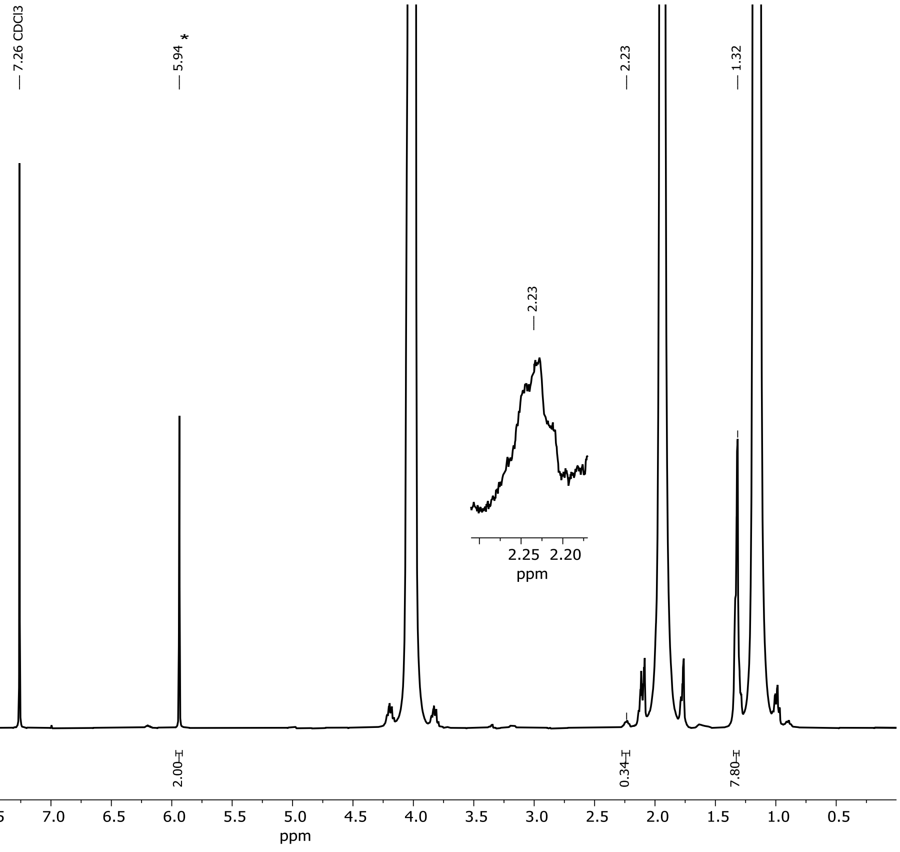

Cyclobutanone 3d

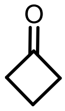

Crude **^{1}H NMR** (400 MHz, $CDCl_3$)

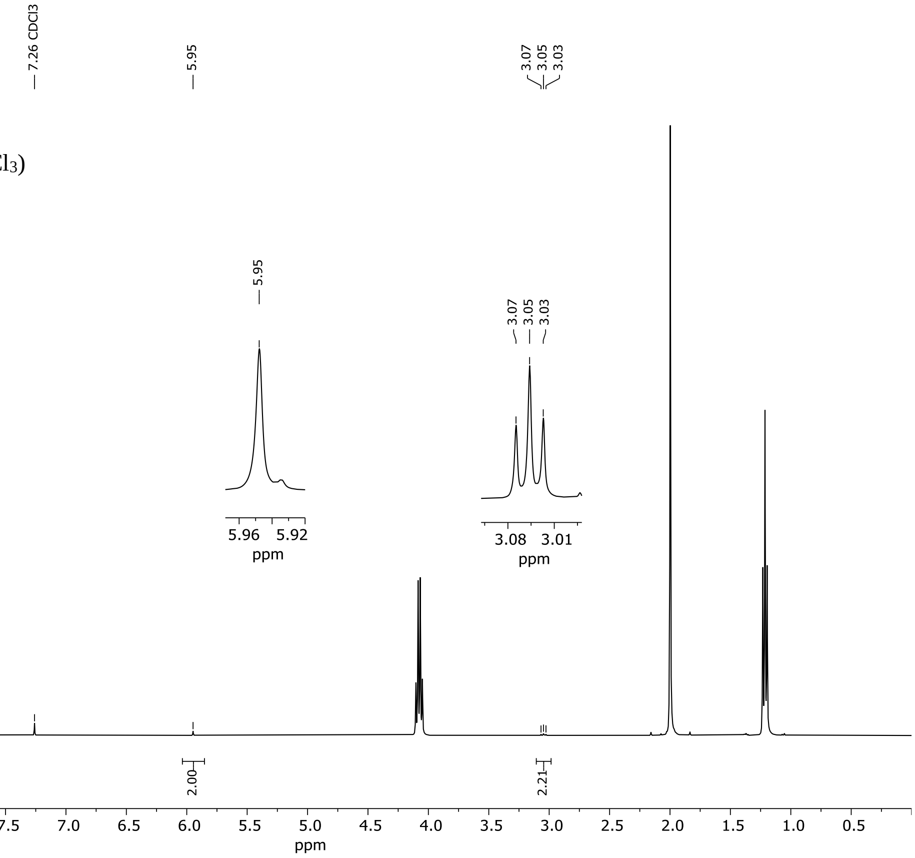

Octan-2-one 3e

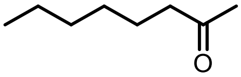

Crude **^{1}H NMR** (400 MHz, $CDCl_3$)

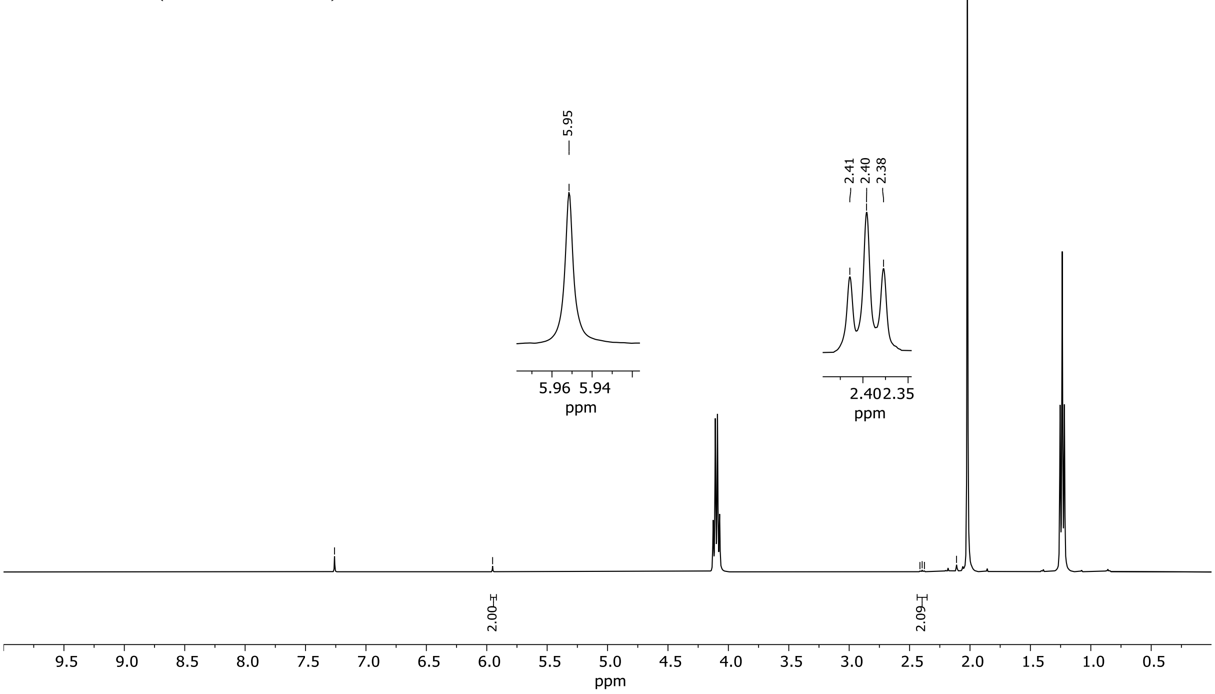

4-oxopentanoic acid 3f

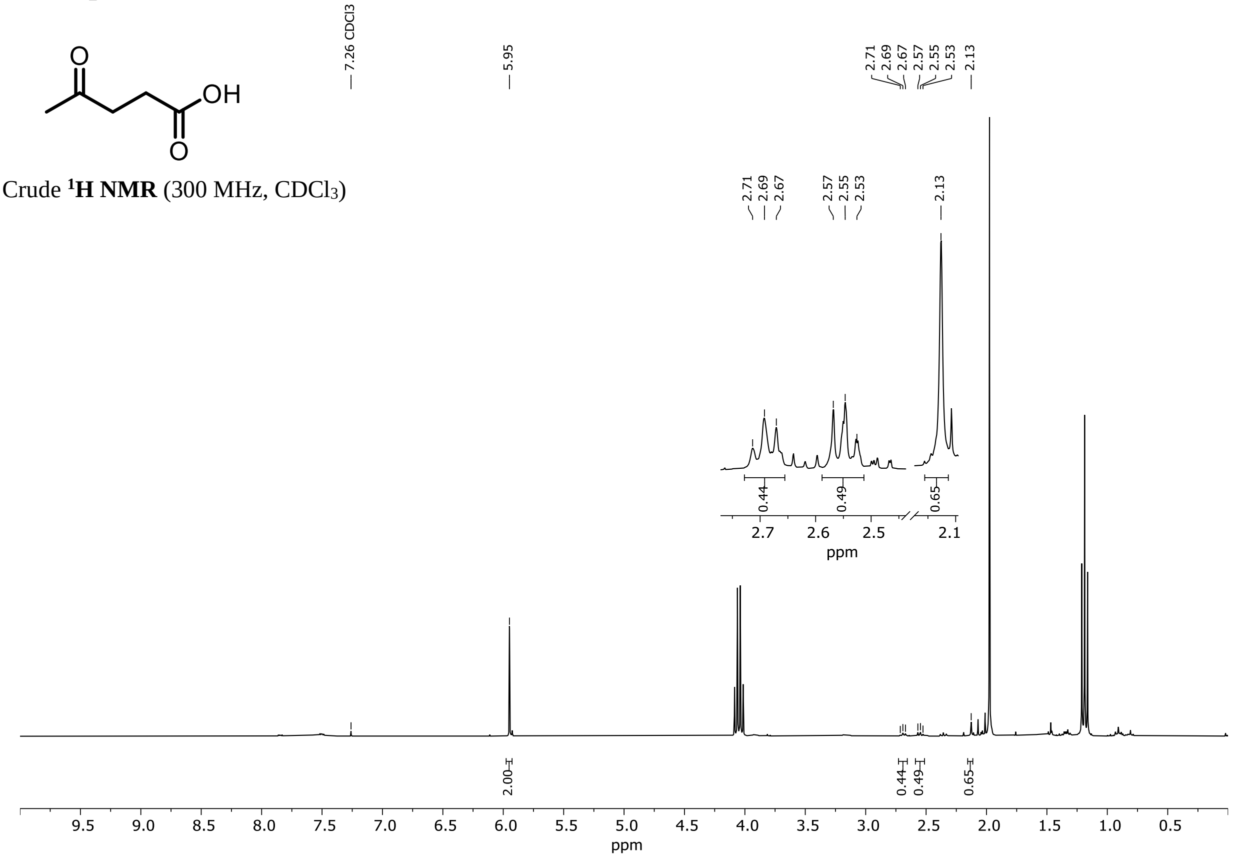

Crude [1]**H NMR** (300 MHz, $CDCl_3$)

2-Hydroxy-1-indanone 3g

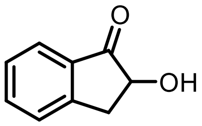

3g

^{1}H NMR (400 MHz, $CDCl_3$)

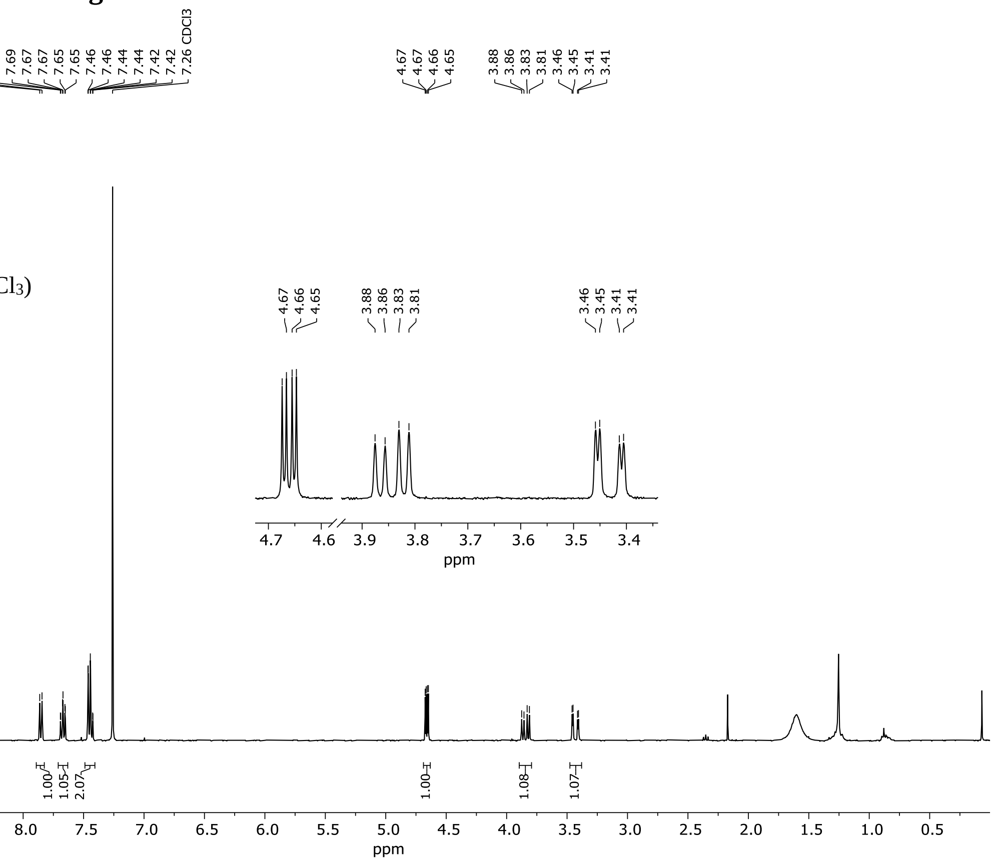

Benzoic acid 3h

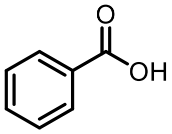

Crude ^{1}**H NMR** (400 MHz, DMSO-d$_6$)
synthesis from benzylic alcohol

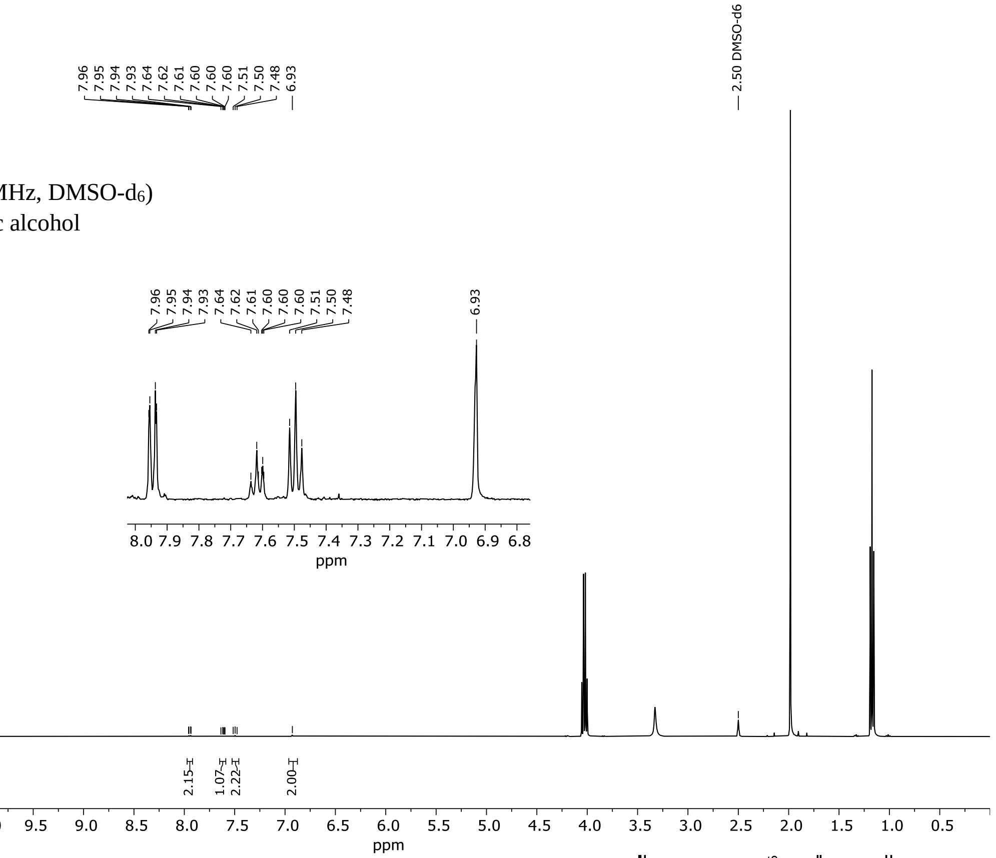

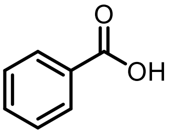

Crude ^{1}**H NMR** (400 MHz, DMSO-d$_6$)
synthesis from benzylic alcohol

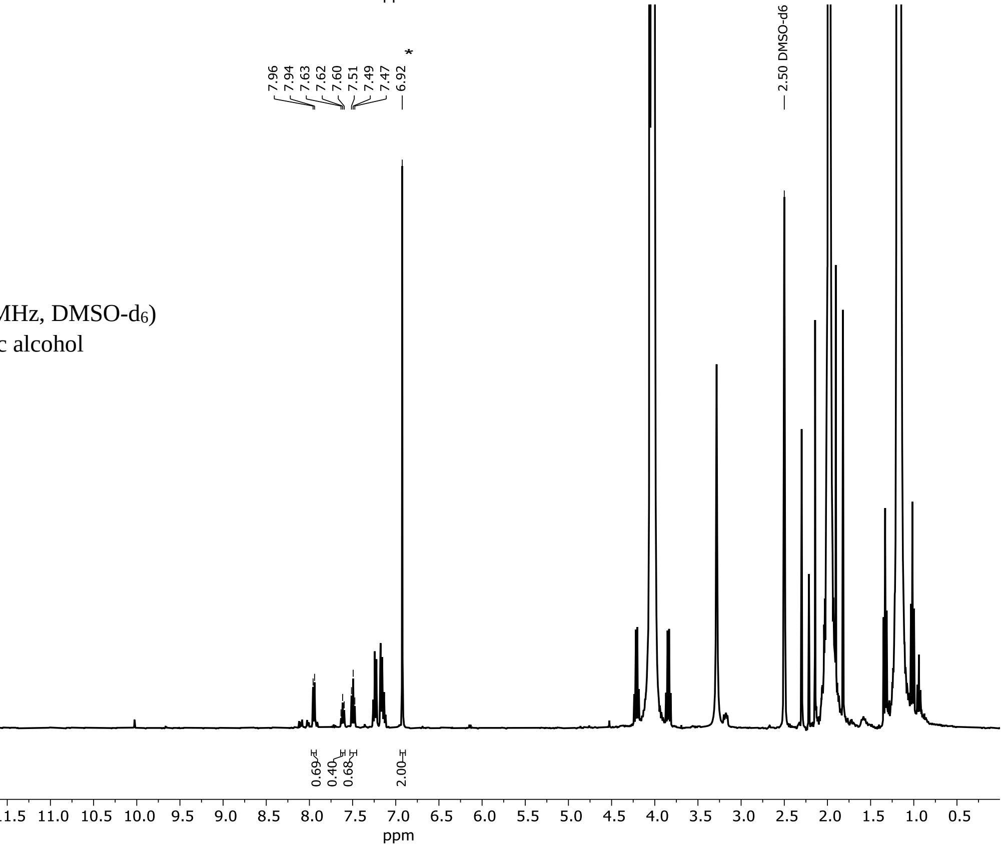

1-(4-methoxyphenyl)ethan-1-one 3i

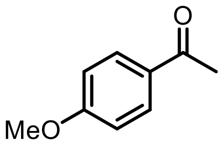

Crude **^{1}H NMR** (400 MHz, DMSO-d6)

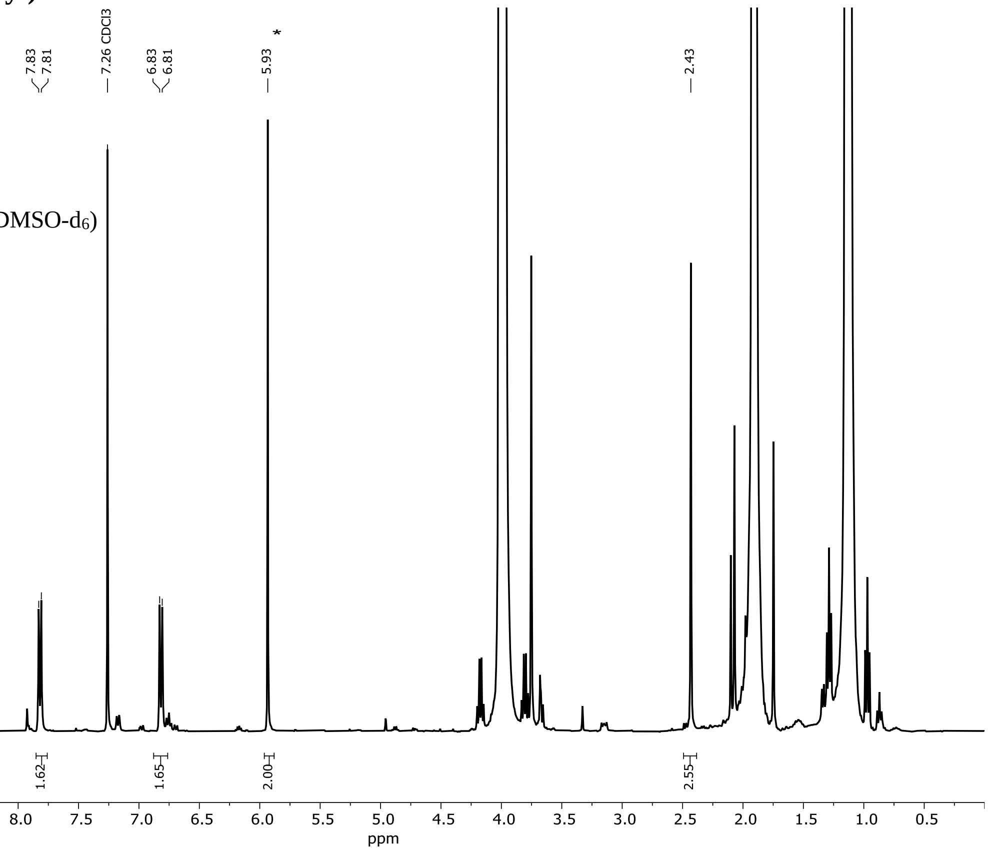

1-acetylnaphthalene 3j

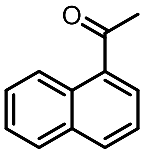

Crude ^{1}**H NMR** (400 MHz, DMSO-d_6)

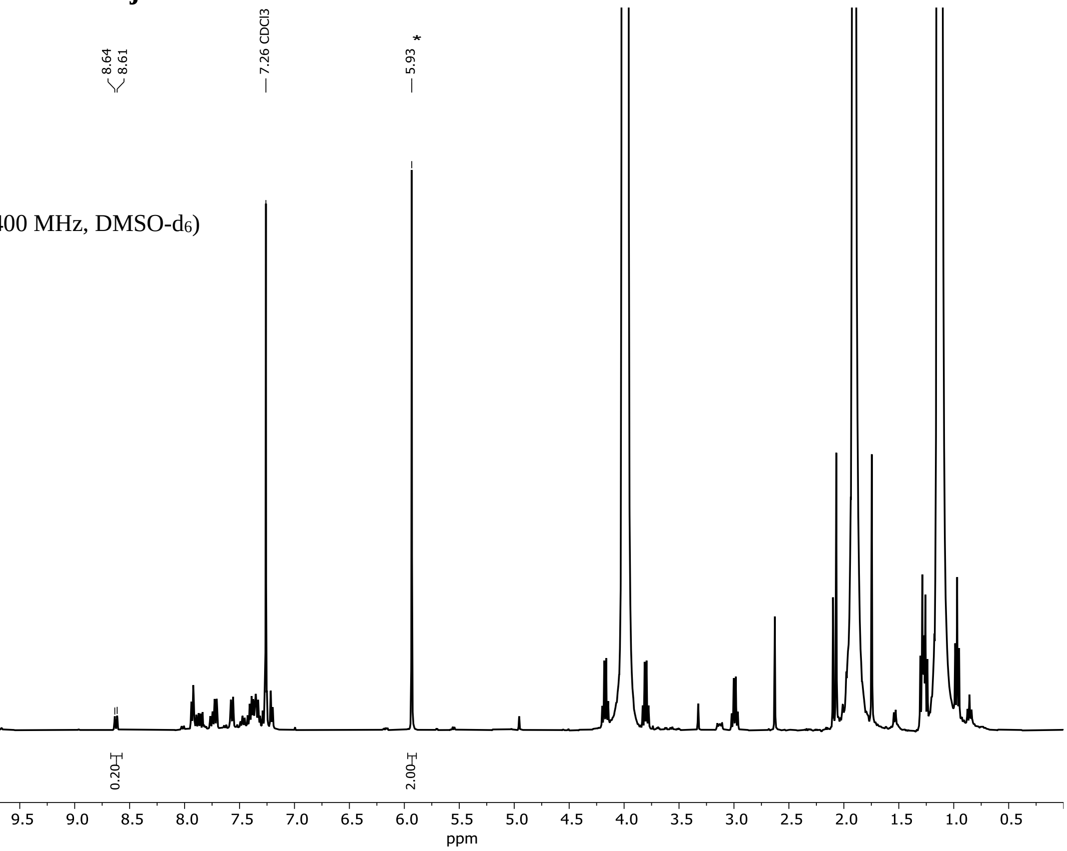

1-tetralone *o*-3k and 2,3-dihydro-1,4-naphthoquinone *o,o*-3k

Crude ^{1}**H NMR** (400 MHz, DMSO-d_6)

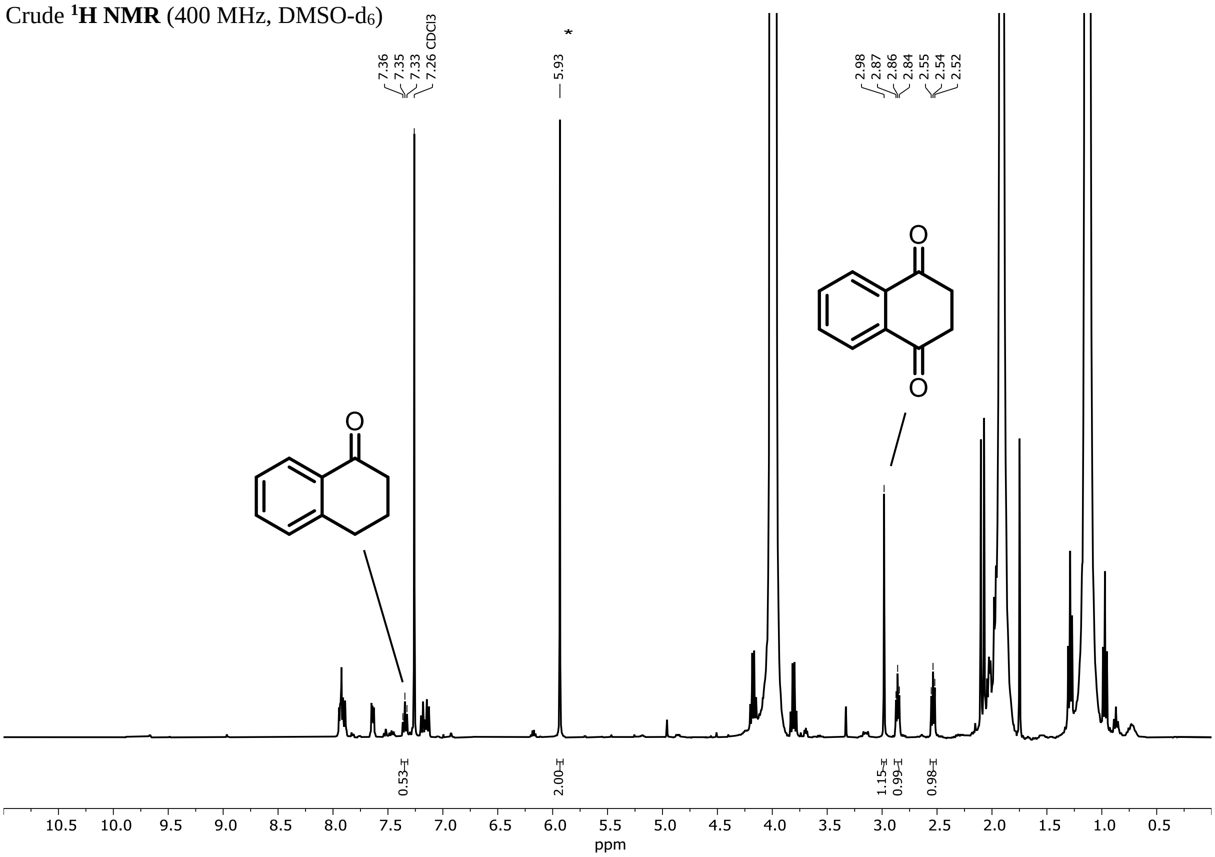

9-fluorenone 3l

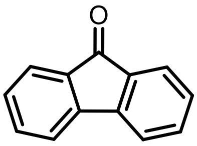

Crude ^{1}**H NMR** (400 MHz, DMSO-d$_6$)

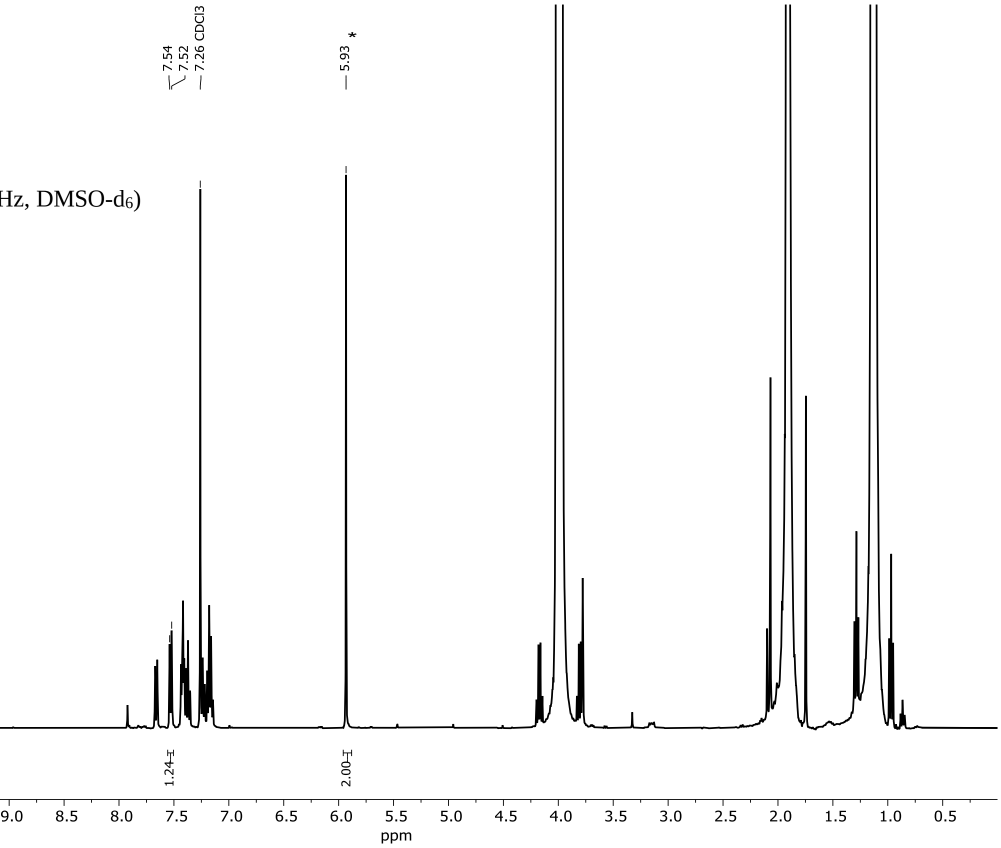

1-methyl-2-(2-(trifluoromethyl)phenyl)-1H-pyrrole 4a

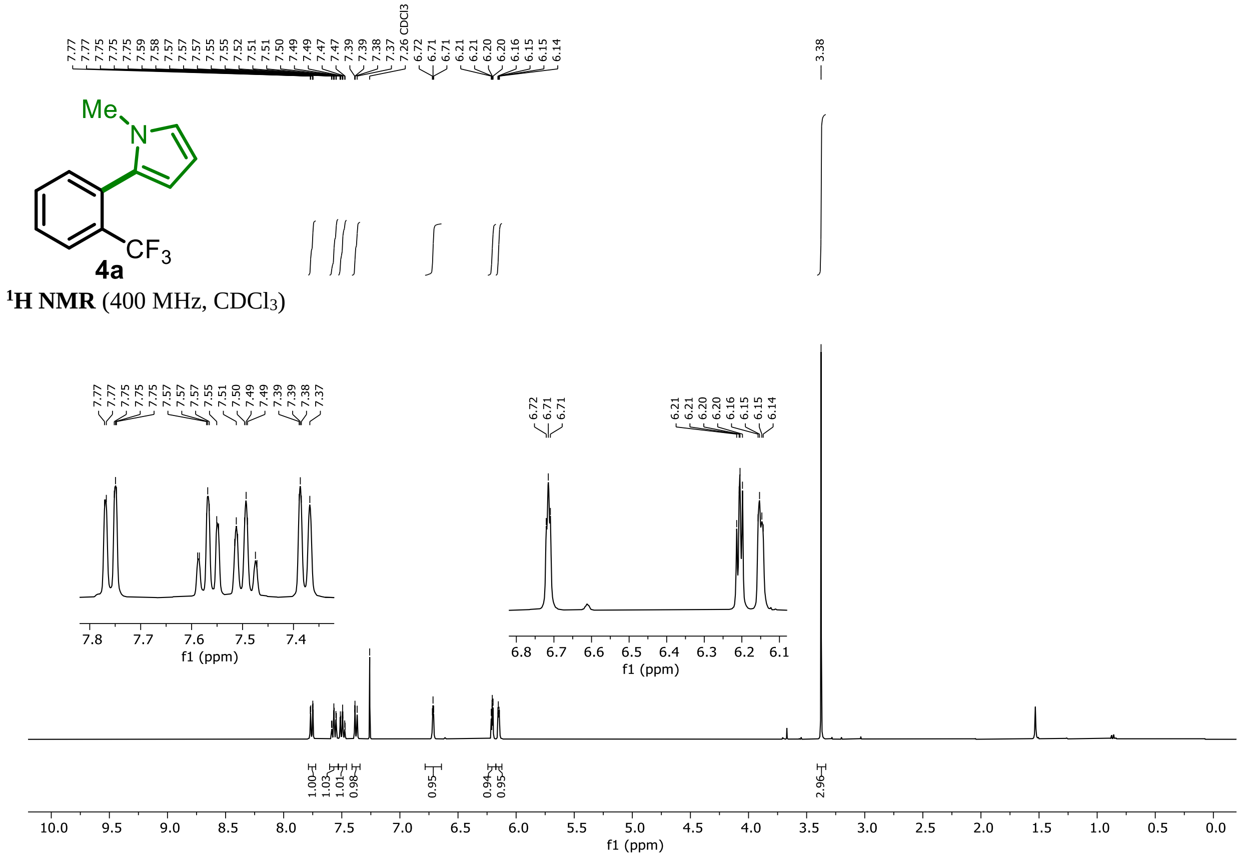

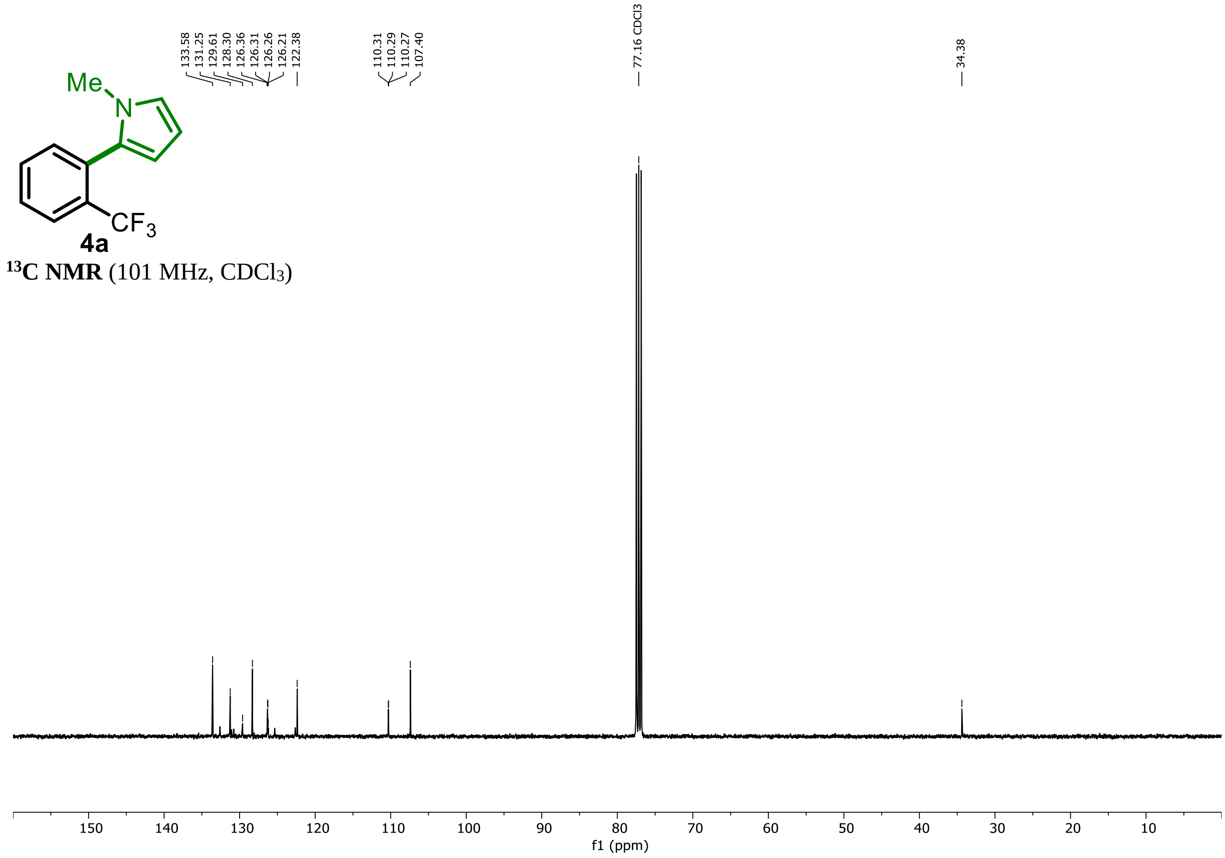

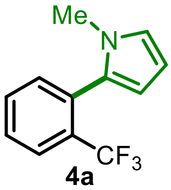

4a

^{19}F NMR (377 MHz, $CDCl_3$)

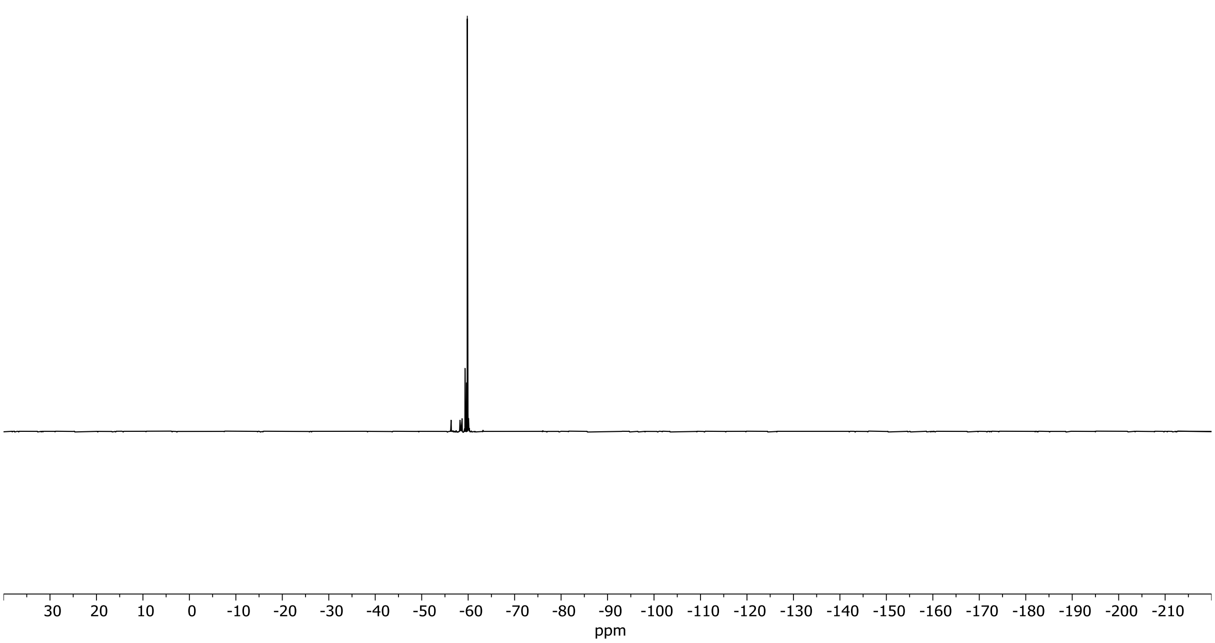

1-phenyl-2-(2-(trifluoromethyl)phenyl)-1H-pyrrole 4b

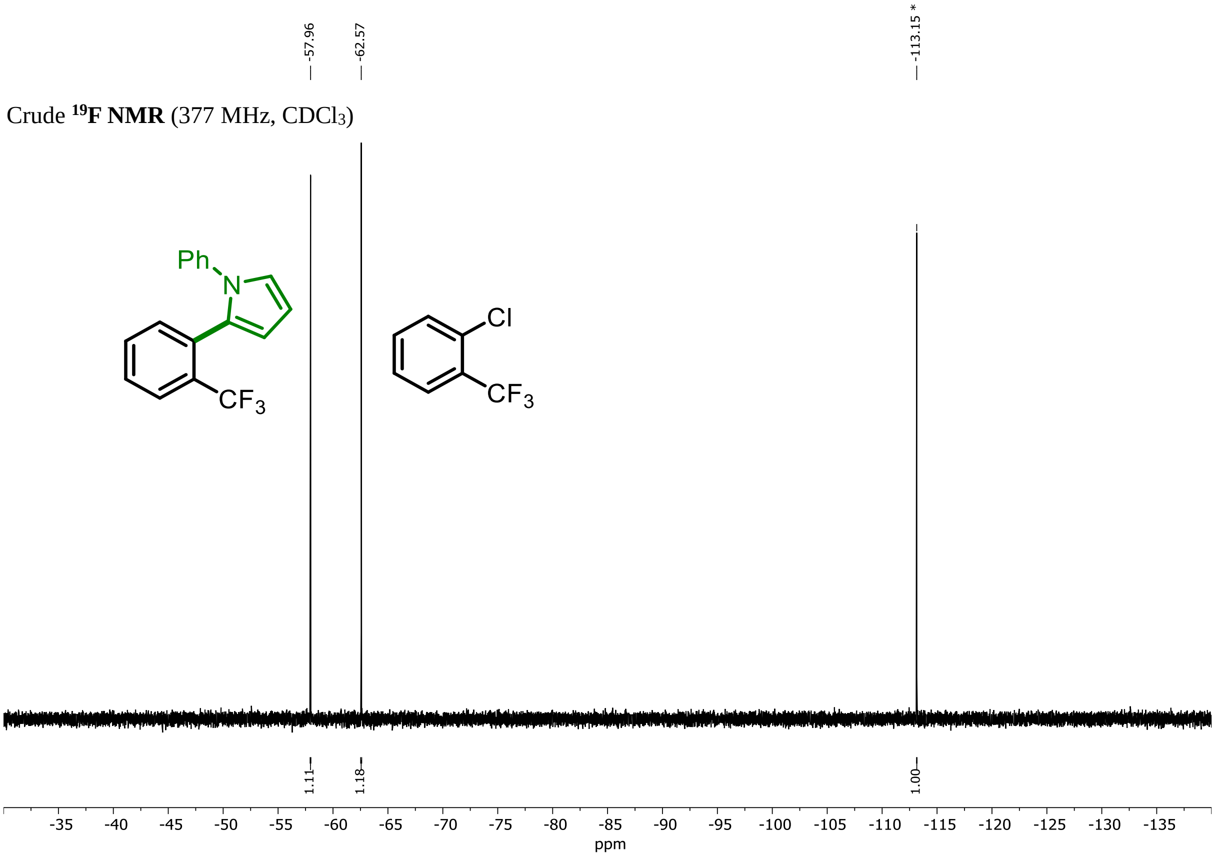

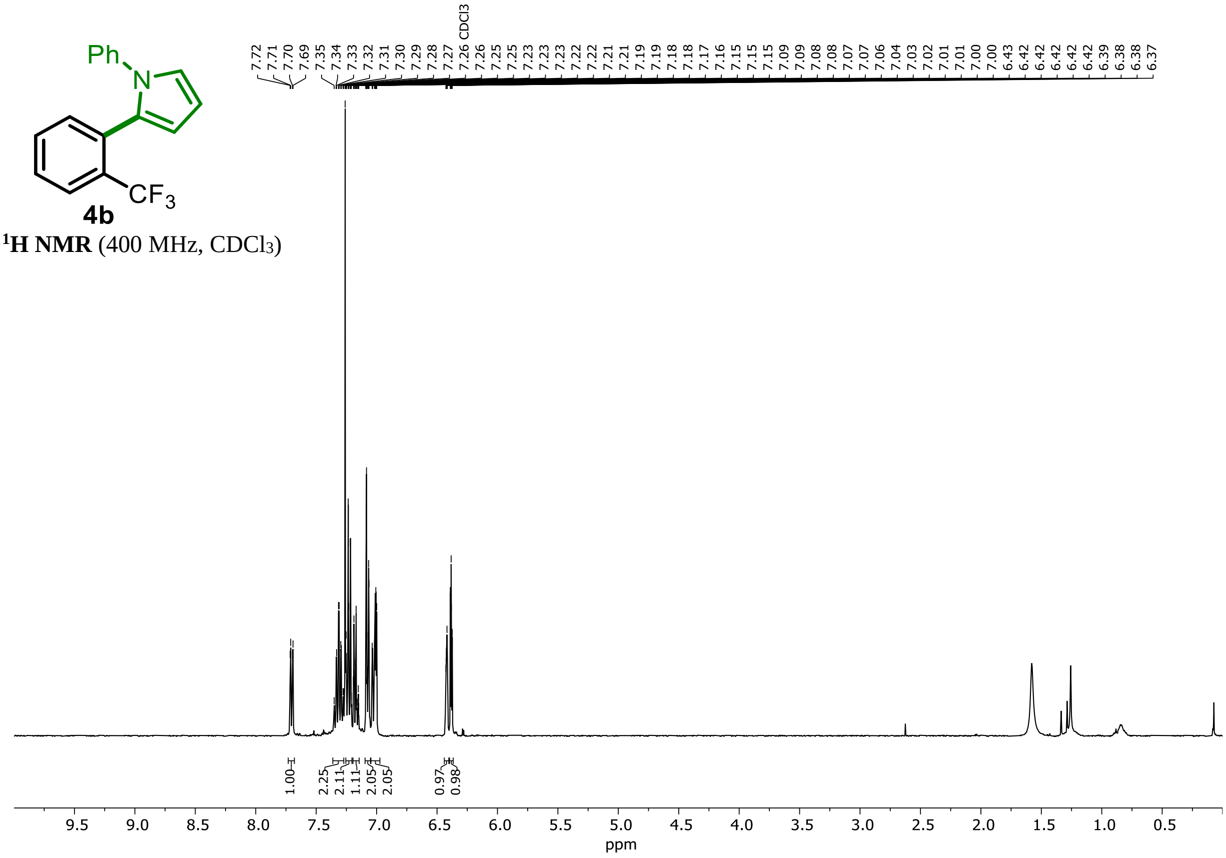

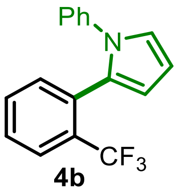

4b

^{13}**C NMR** (101 MHz, $CDCl_3$)

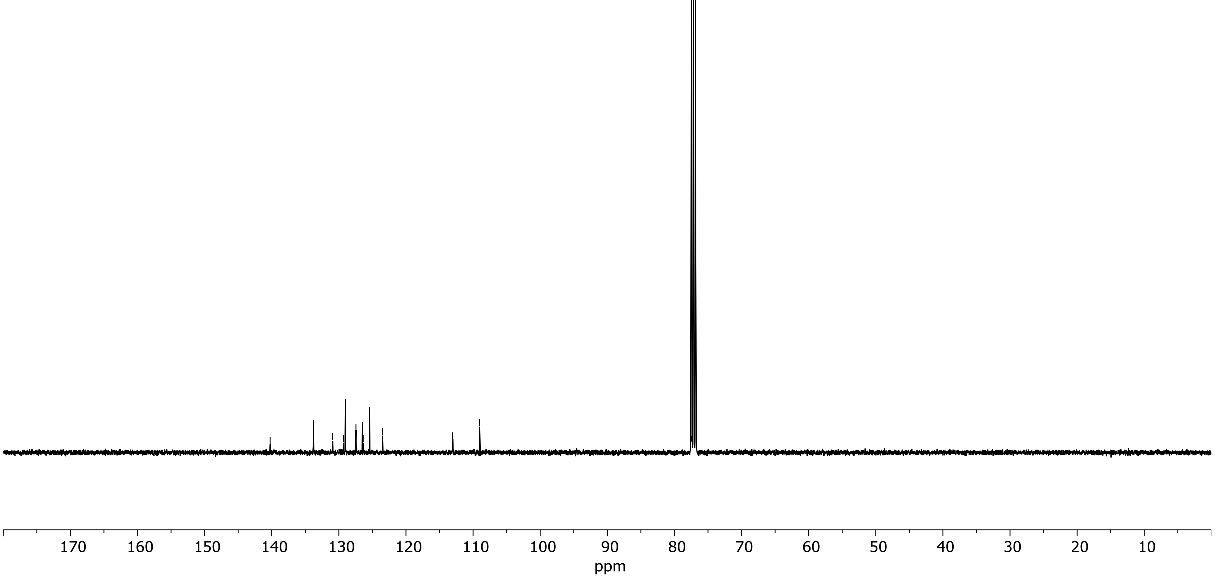

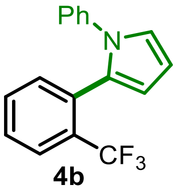

4b

^{19}**F NMR** (377 MHz, $CDCl_3$)

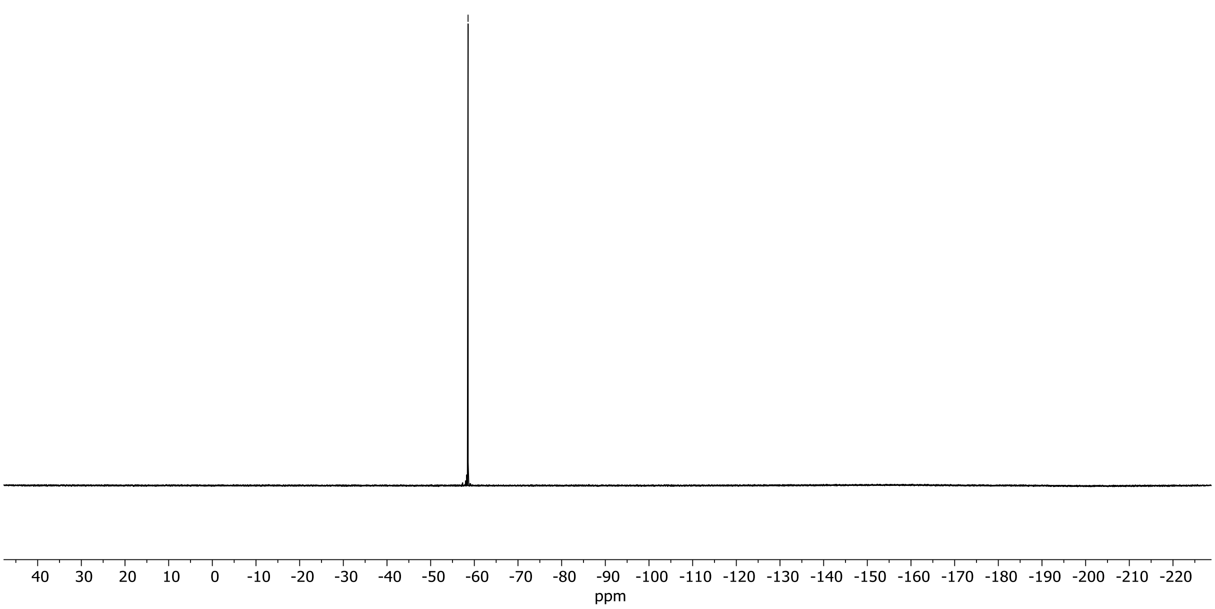

1-phenyl-2-(4-(trifluoromethyl)phenyl)-1H-pyrrole 4c

Crude [19]**F NMR** (377 MHz, $CDCl_3$)

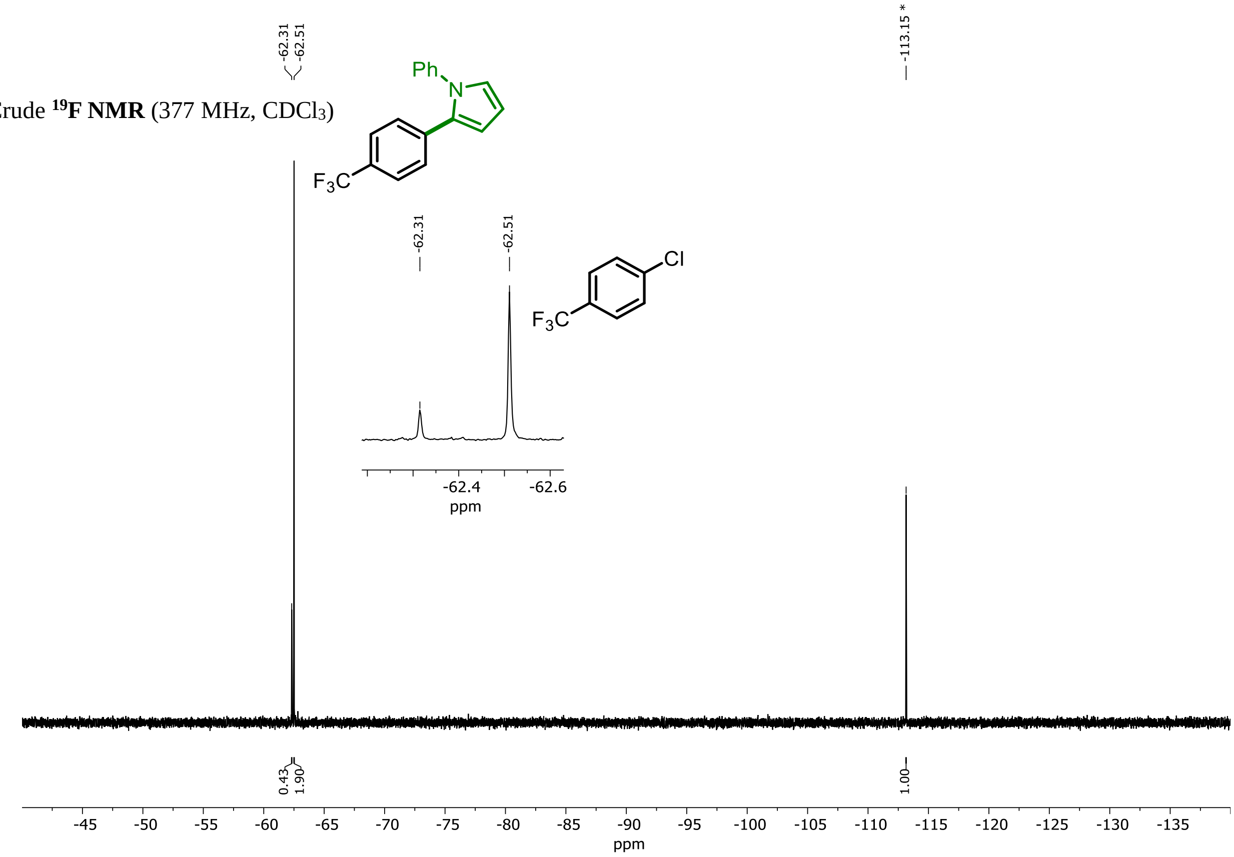

(2-(2-(trifluoromethyl)phenyl)ethene-1,1-diyl)dibenzene 4d

Crude ^{19}F NMR (377 MHz, $CDCl_3$)

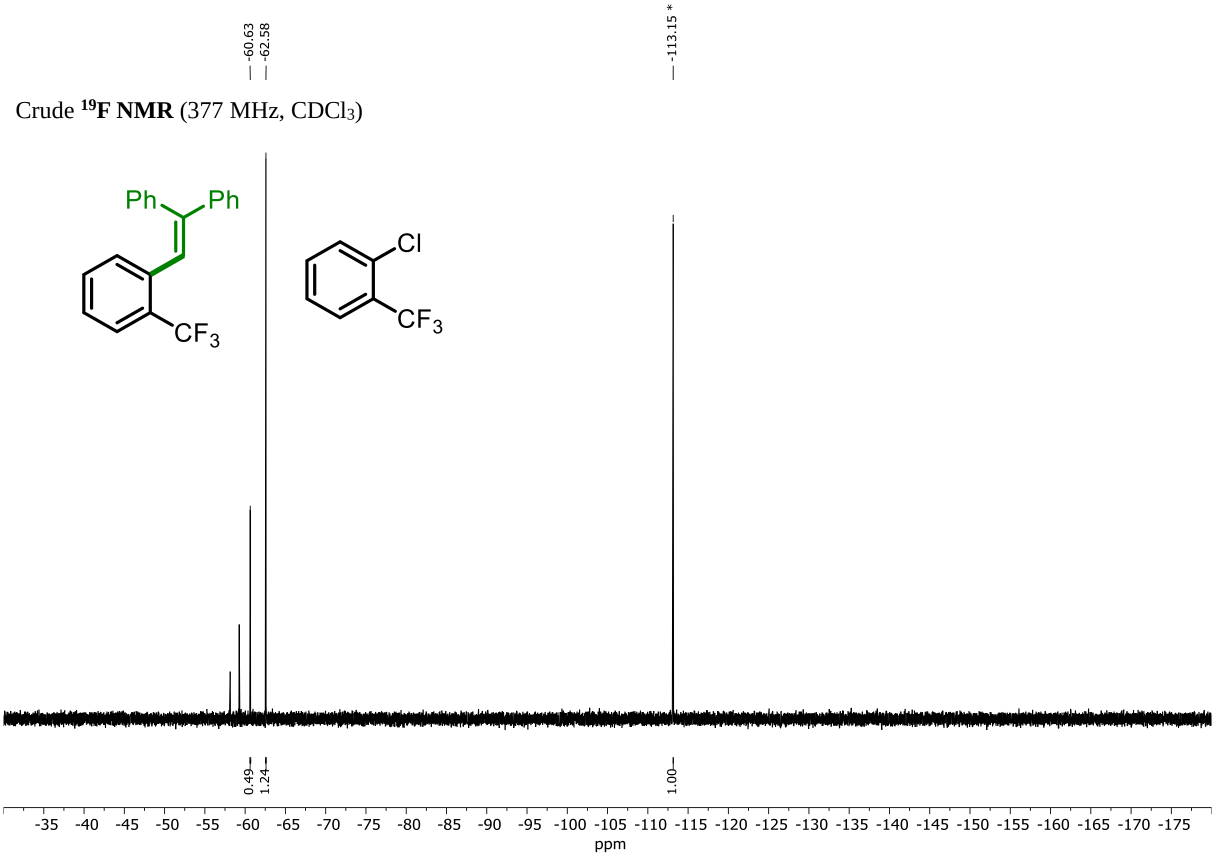

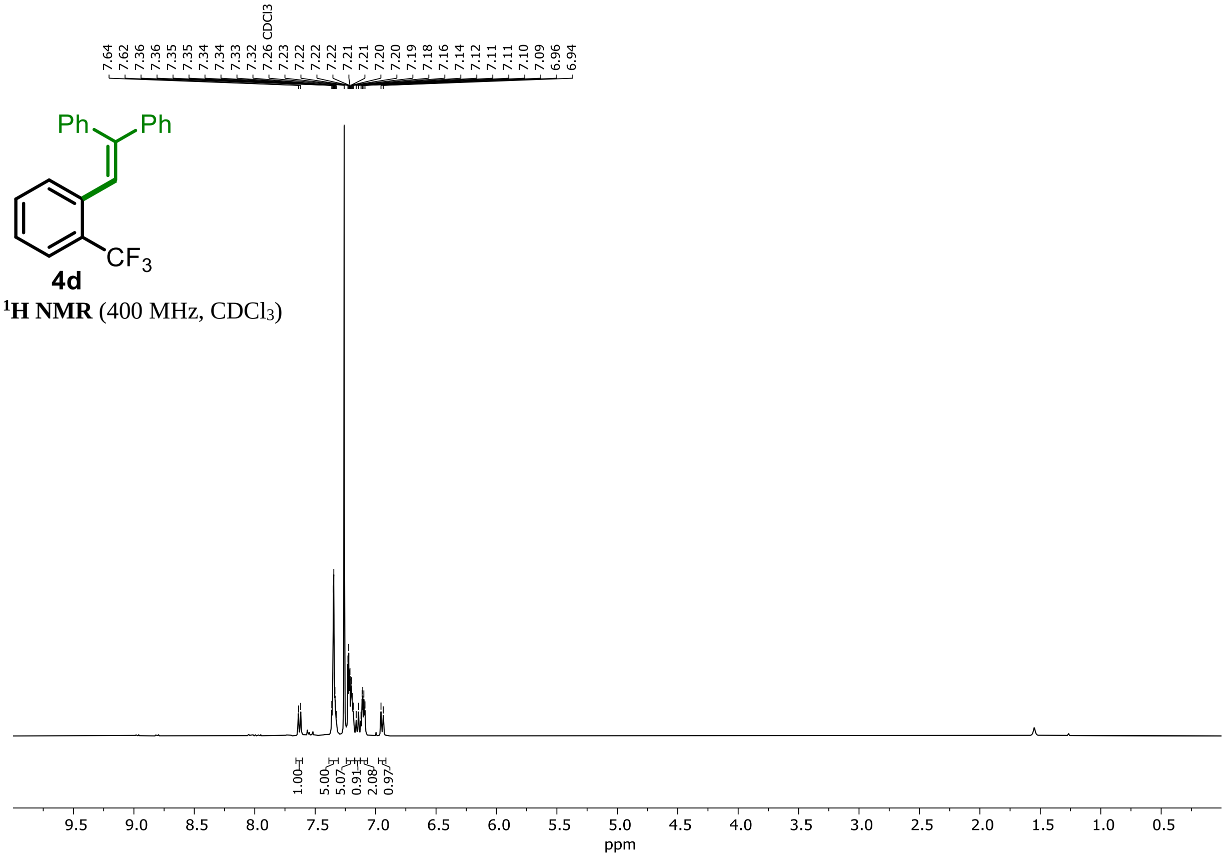

^{1}H NMR (400 MHz, $CDCl_3$)

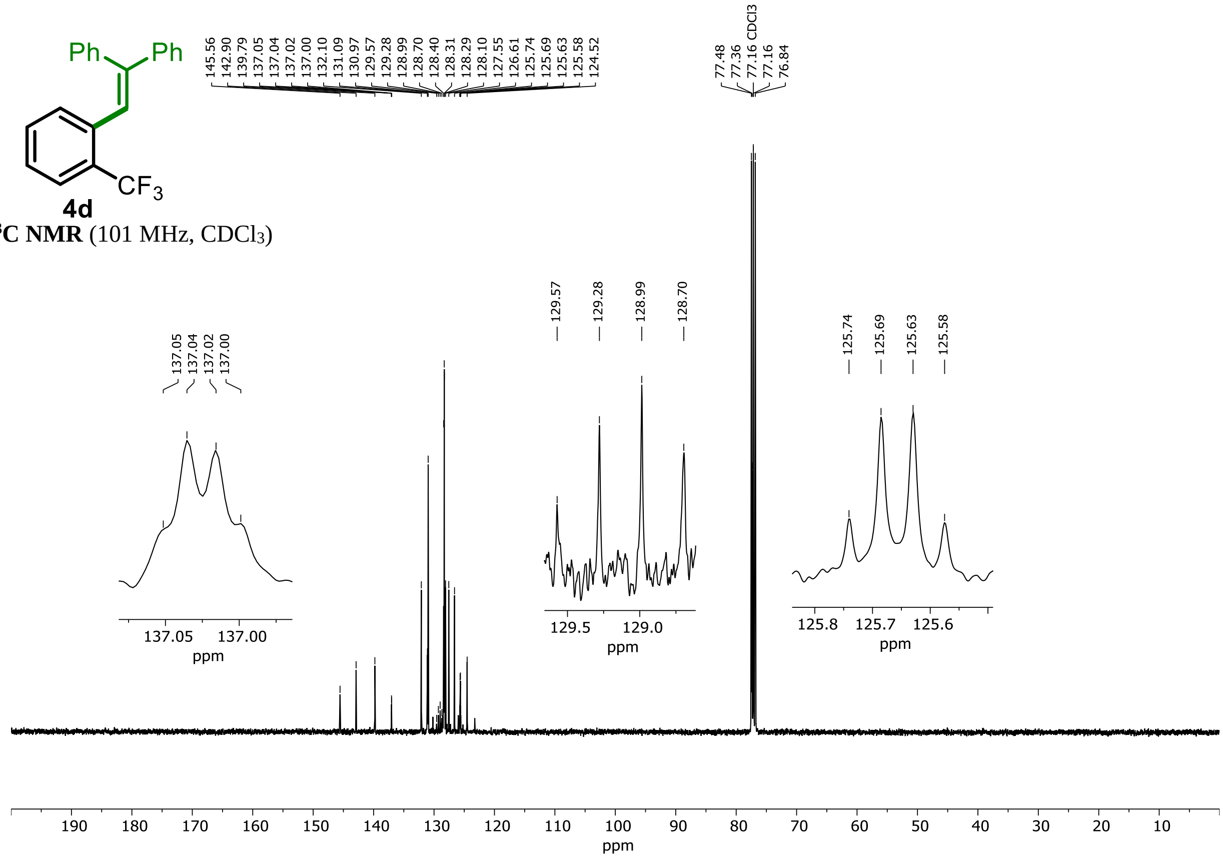

¹³C NMR (101 MHz, $CDCl_3$)

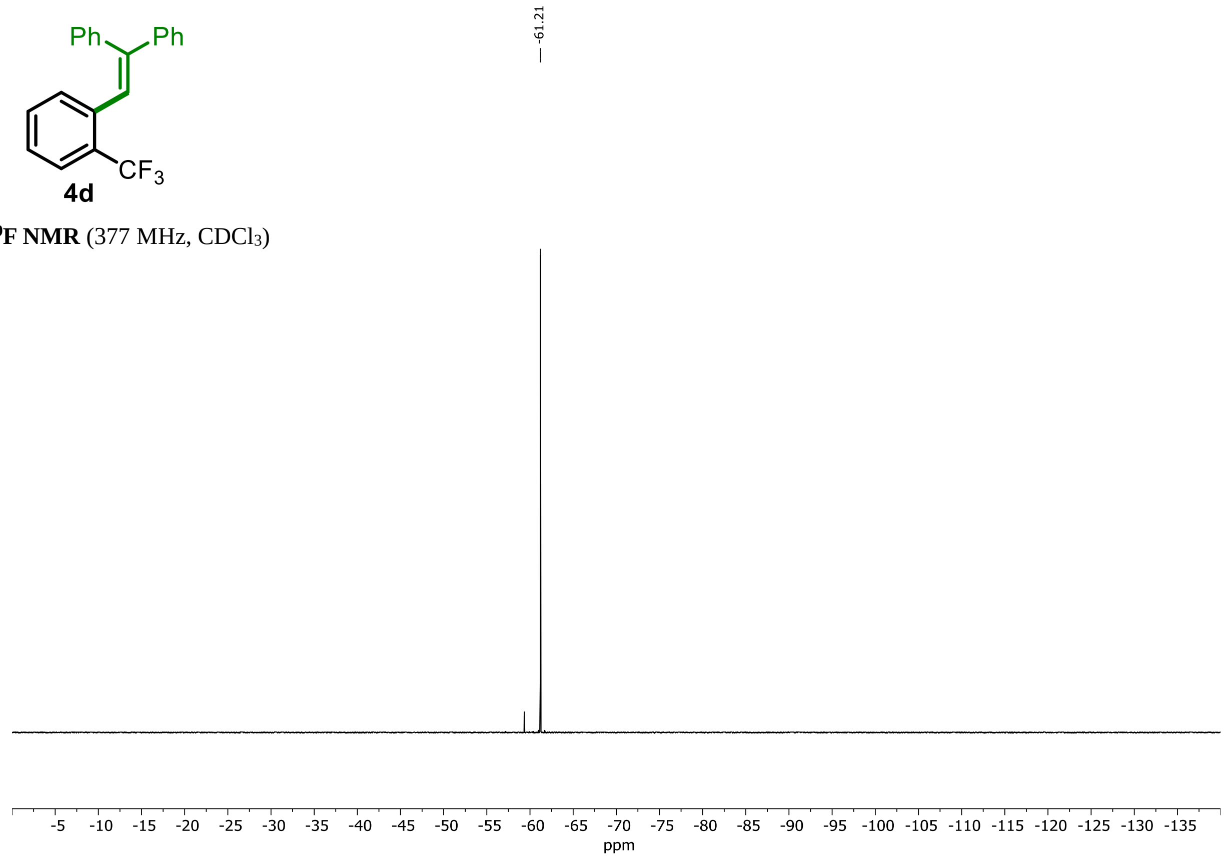

¹⁹F NMR (377 MHz, $CDCl_3$)

1-methyl-2-phenyl-1H-pyrrole 4e

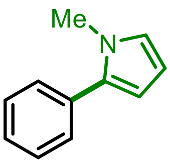

4e

^{1}H NMR (400 MHz, $CDCl_3$)

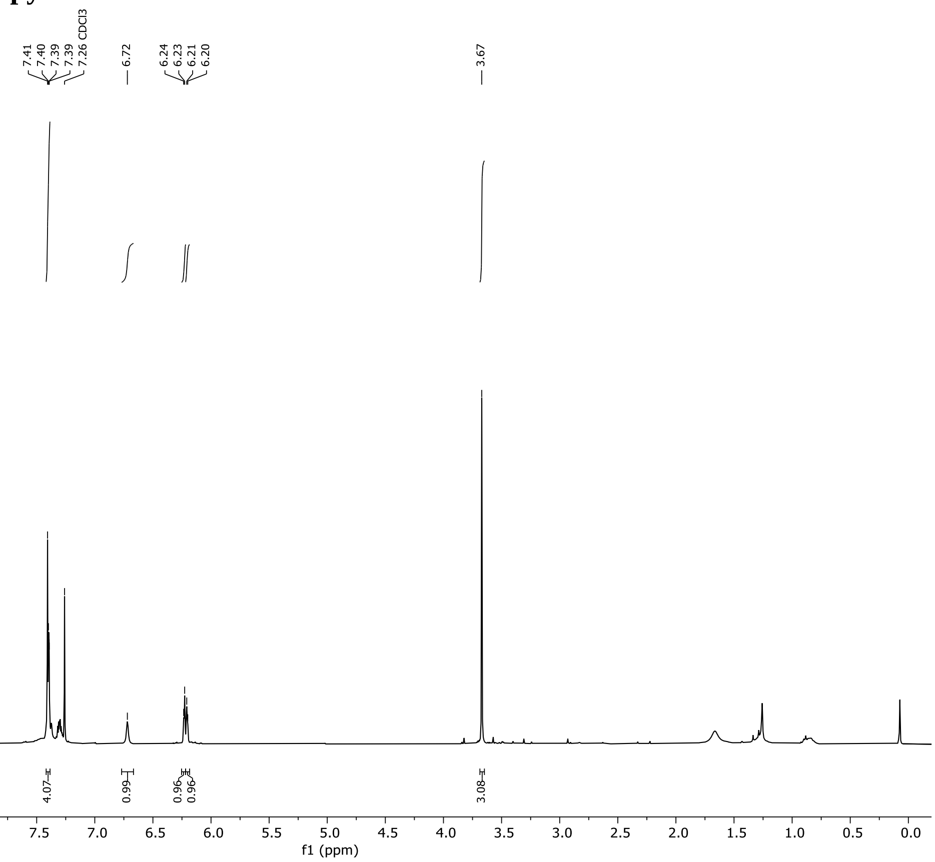

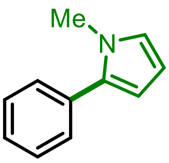

4e

^{13}C NMR (101 MHz, $CDCl_3$)

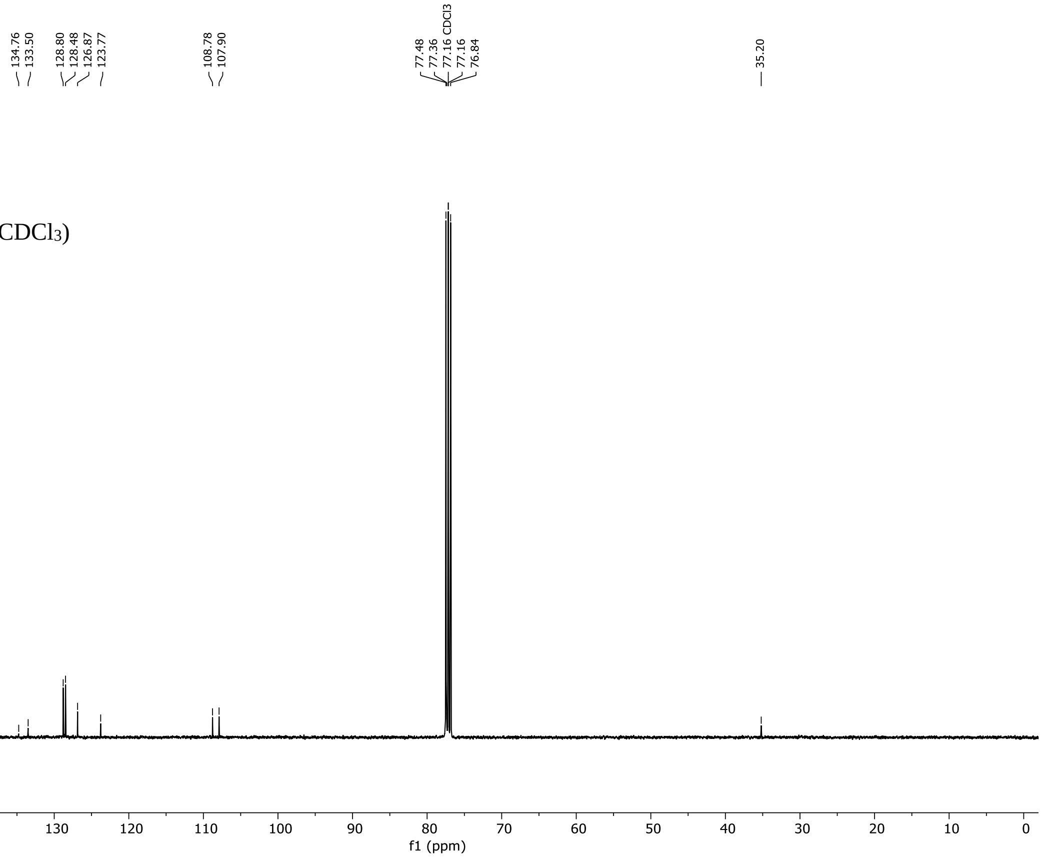

2-(4-methoxyphenyl)-1-methyl-1H-pyrrole 4f

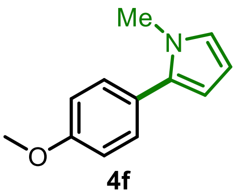

4f

^{1}H NMR (400 MHz, $CDCl_3$)

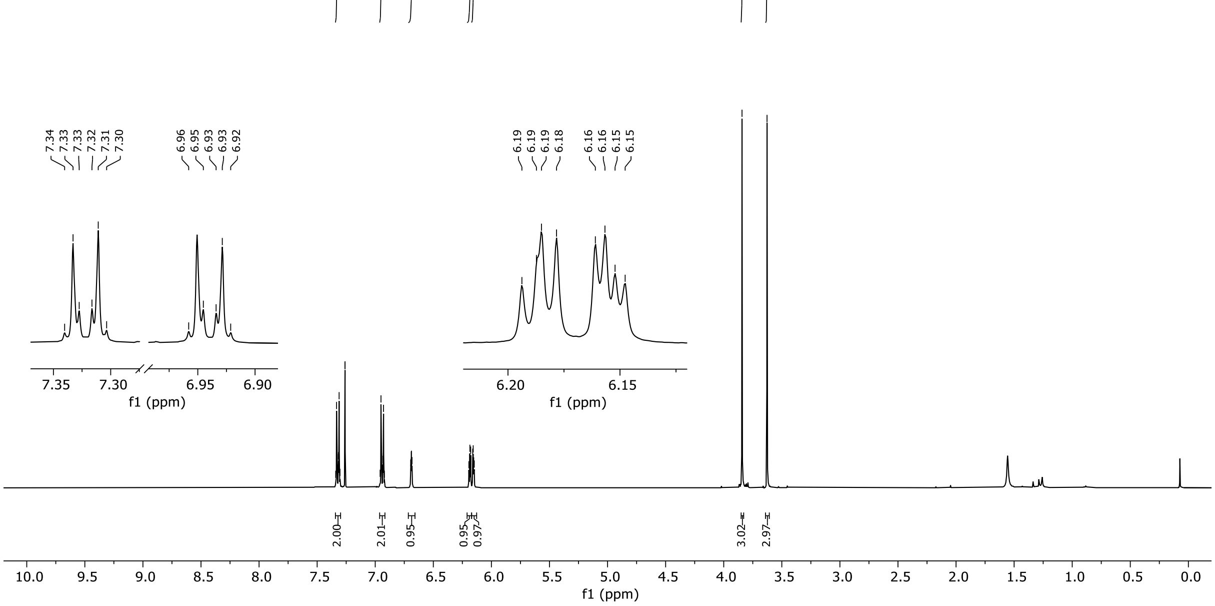

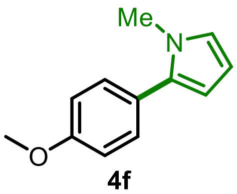

4f

^{13}C NMR (101 MHz, $CDCl_3$)

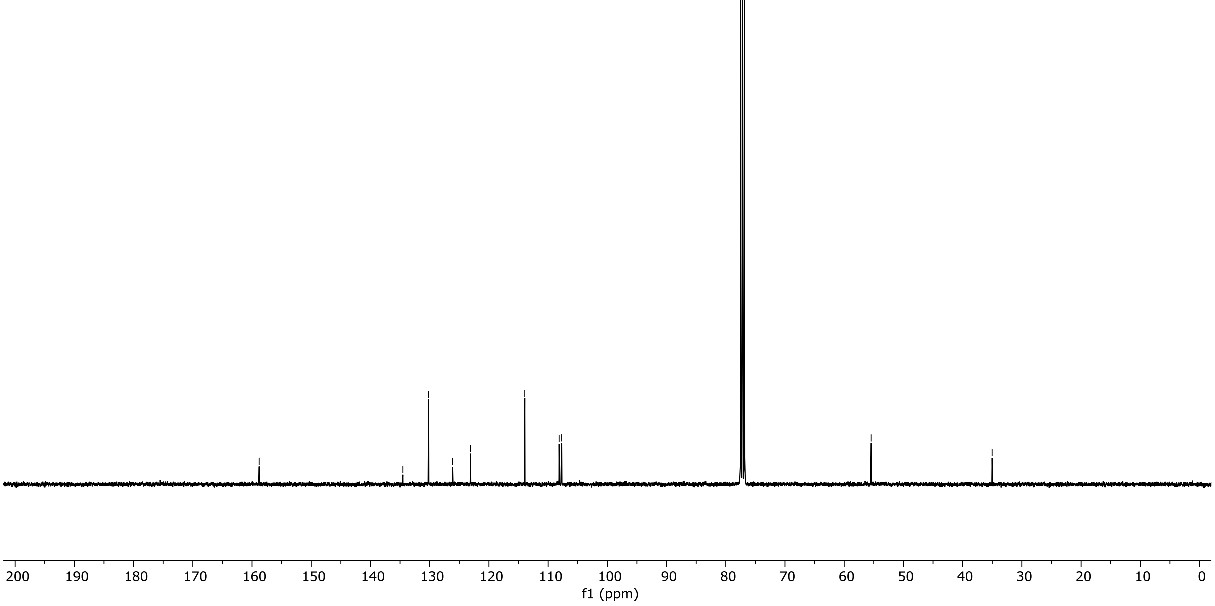

2-(5-chloro-2-(trifluoromethyl)phenyl)-1-methyl-1H-pyrrole and regioisomers 4g

Crude [19]**F NMR** (376 MHz, CDCl3)

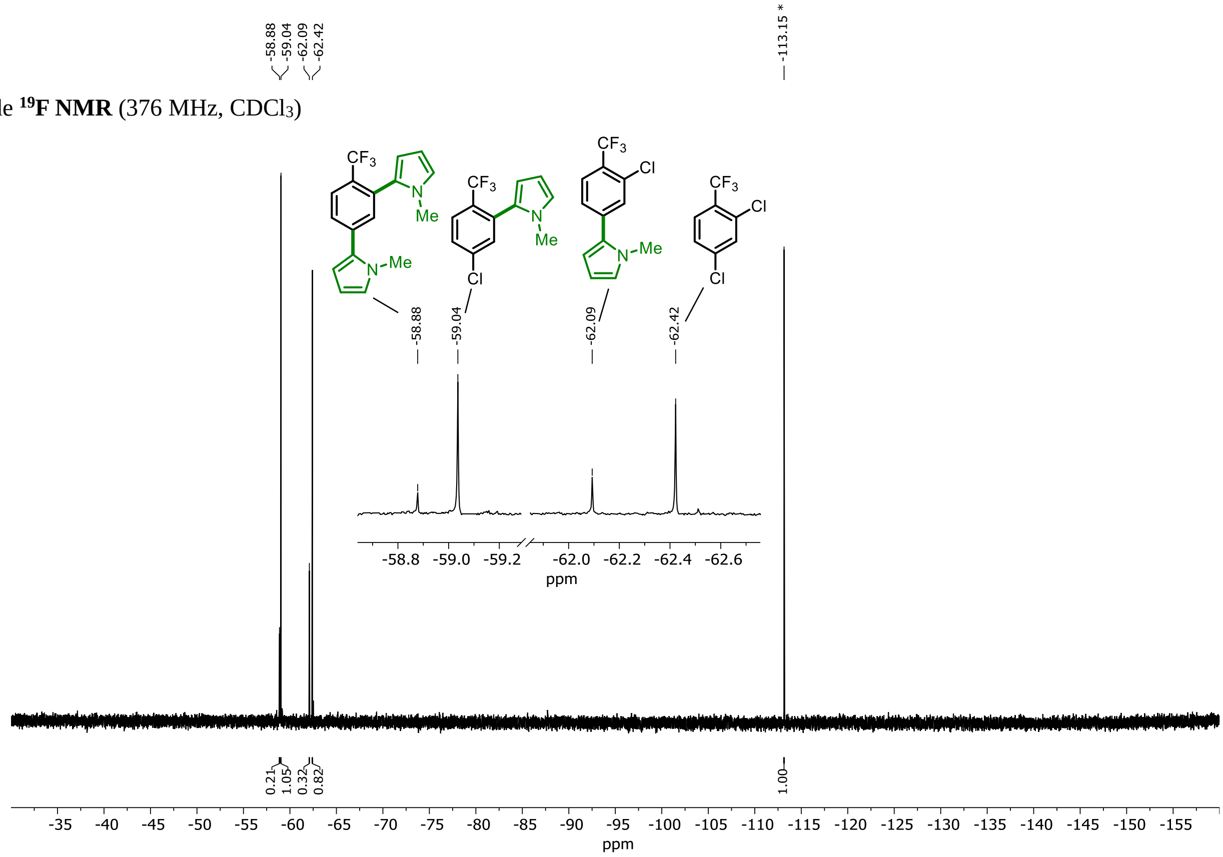

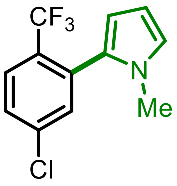

4g-o

[1]**H NMR** (400 MHz, CDCl3)

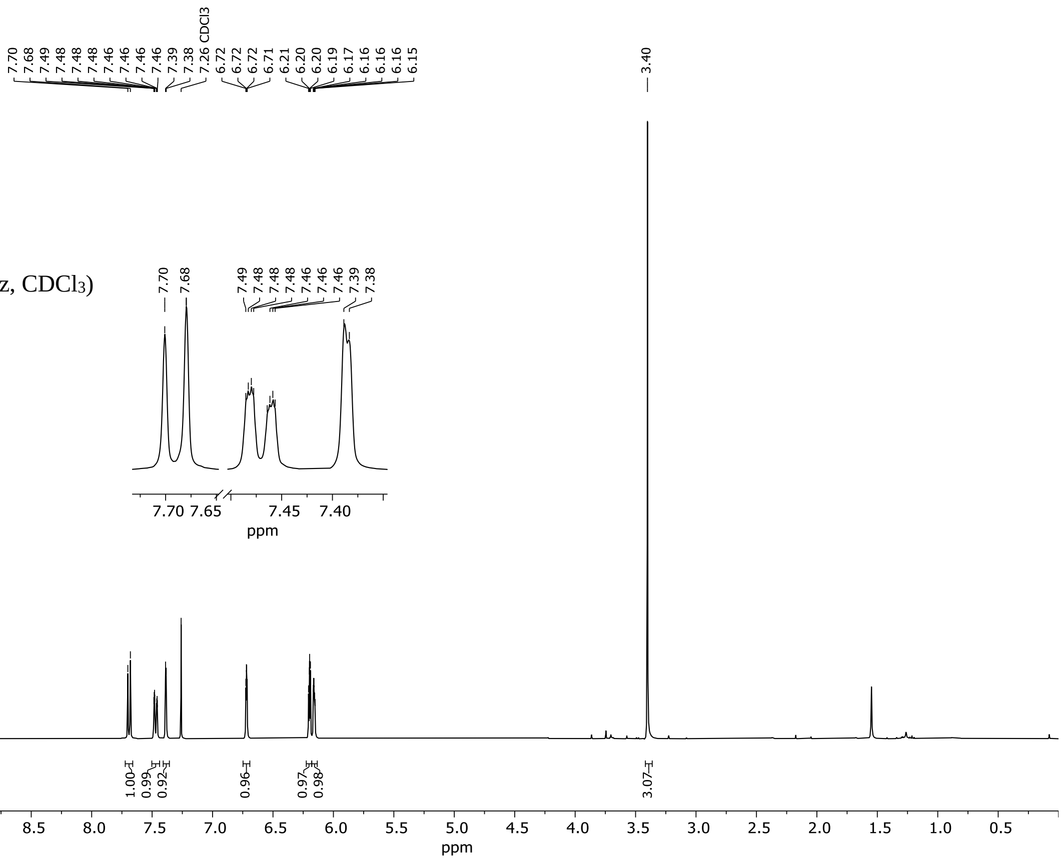

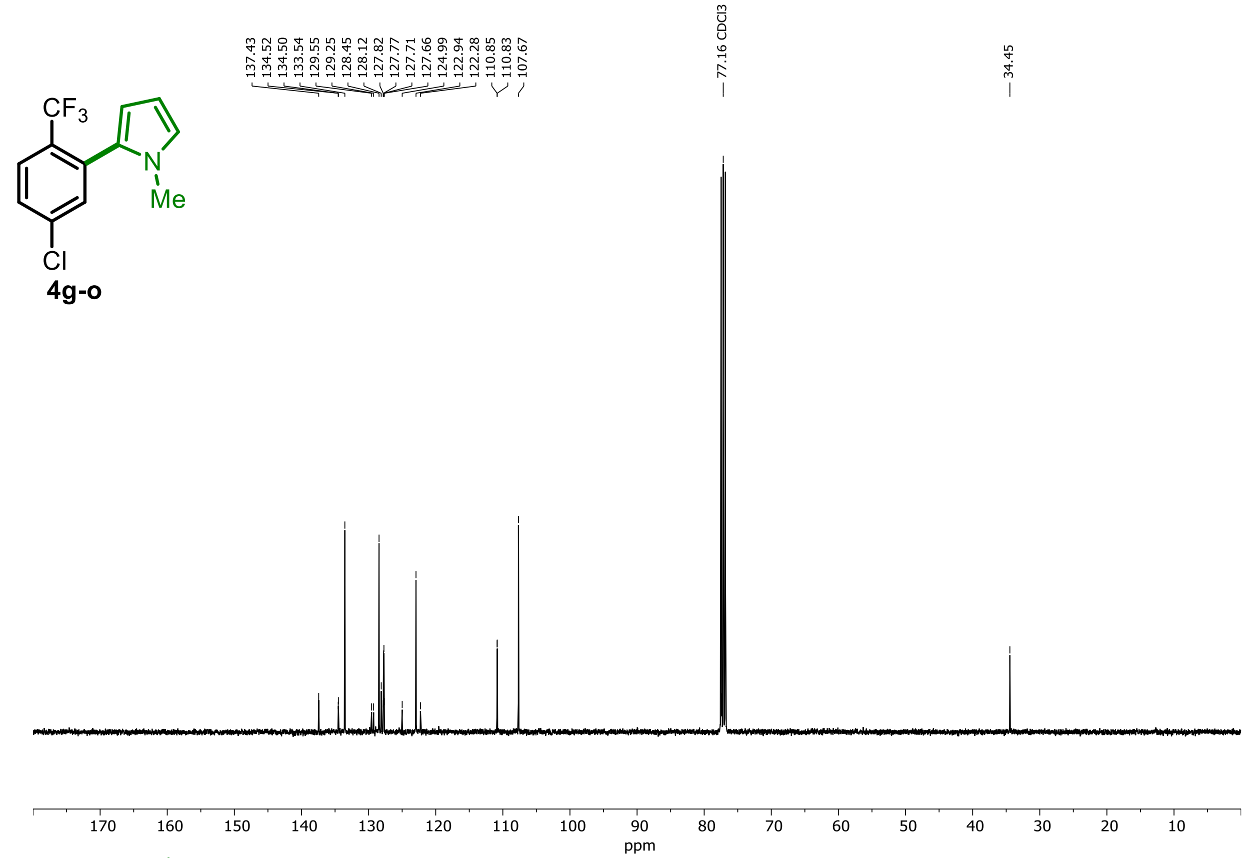

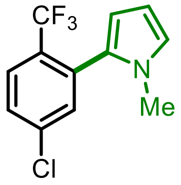

^{19}F NMR (376 MHz, $CDCl_3$)

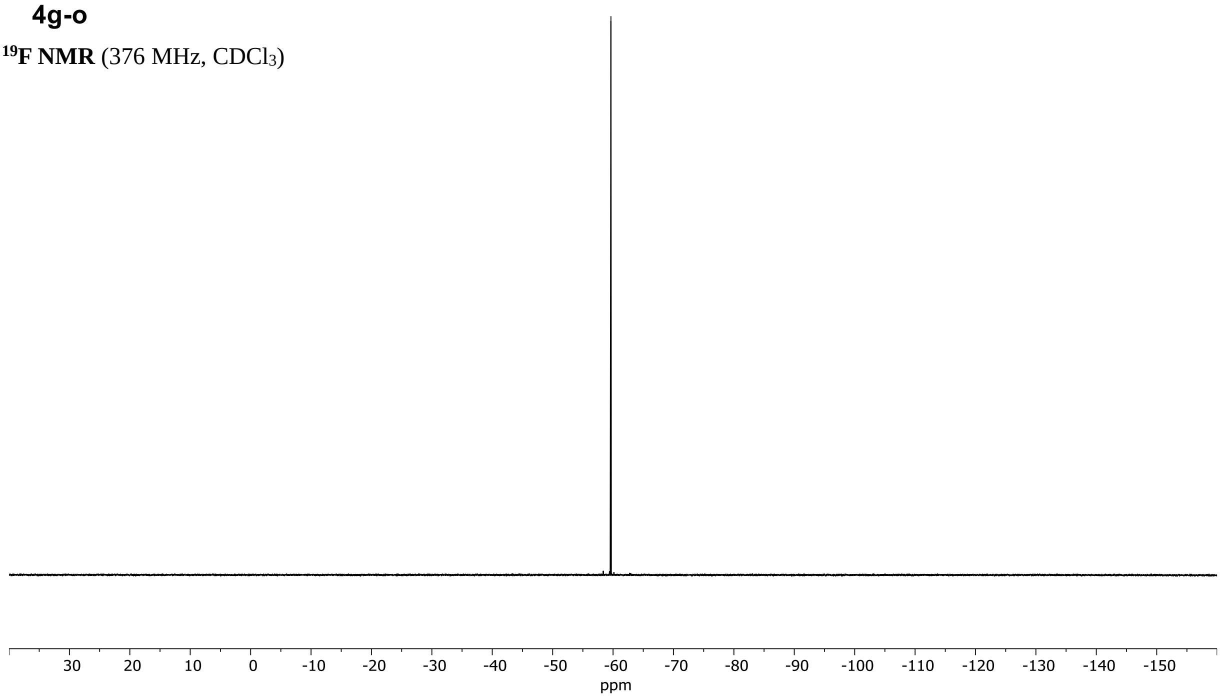

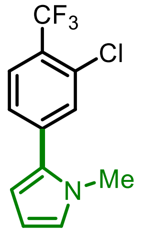

4g-p

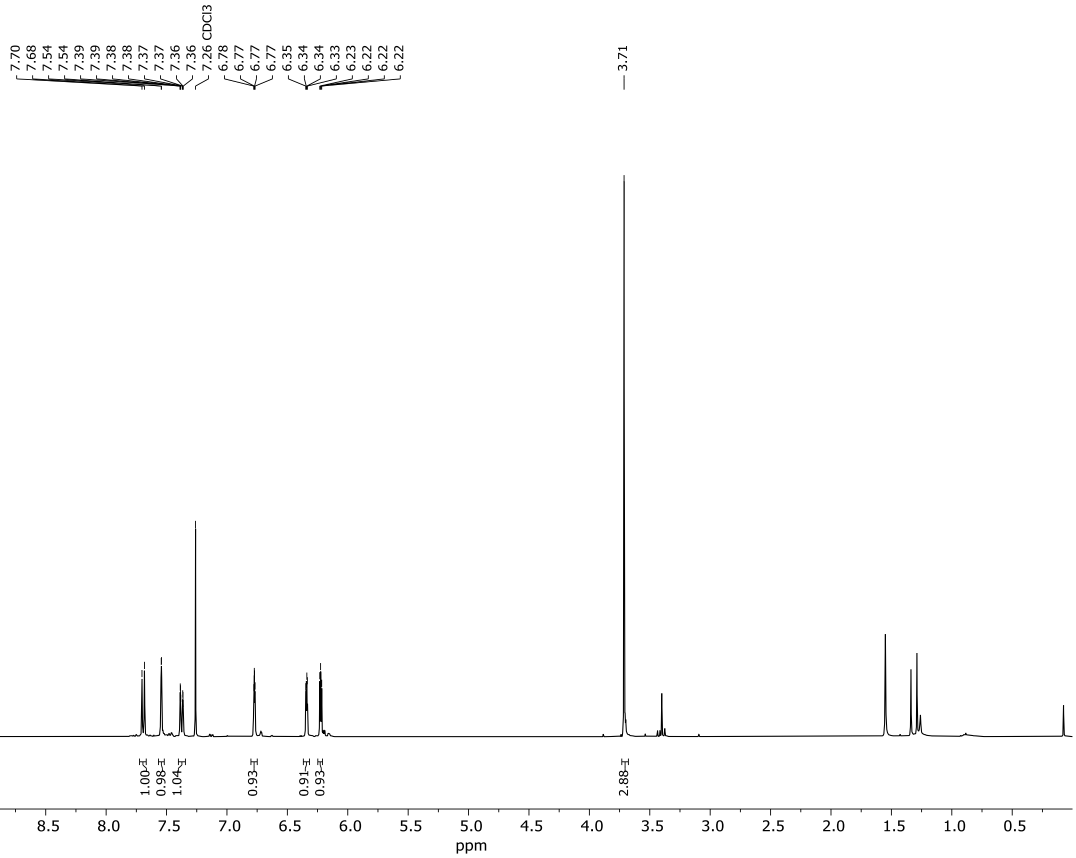

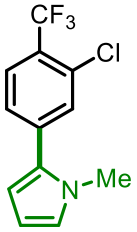

4g-p

^{13}C NMR (101 MHz, $CDCl_3$)

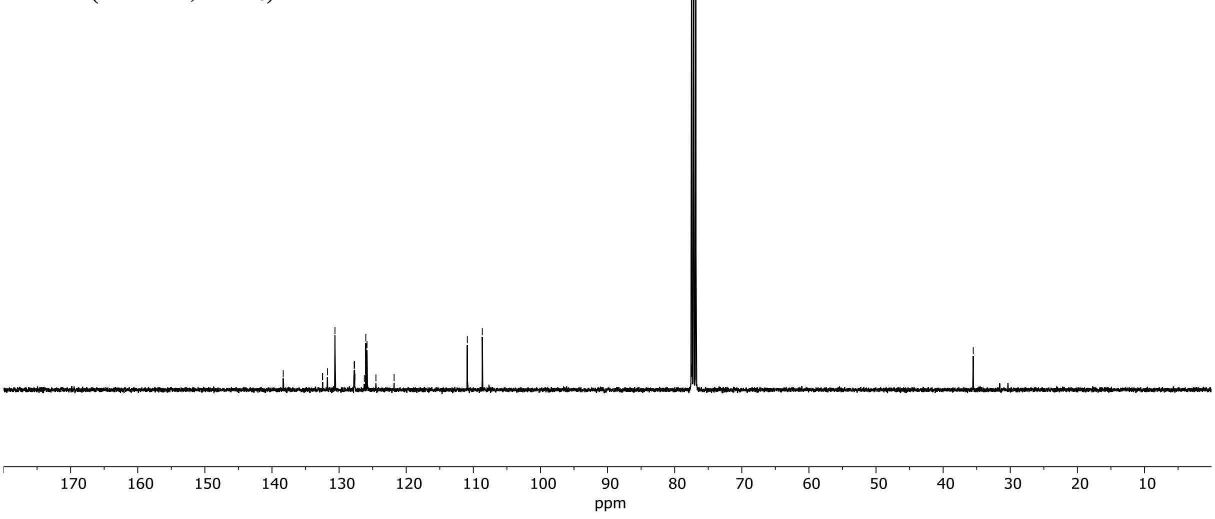

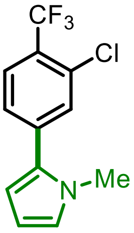

4g-p

^{19}F NMR (376 MHz, $CDCl_3$)

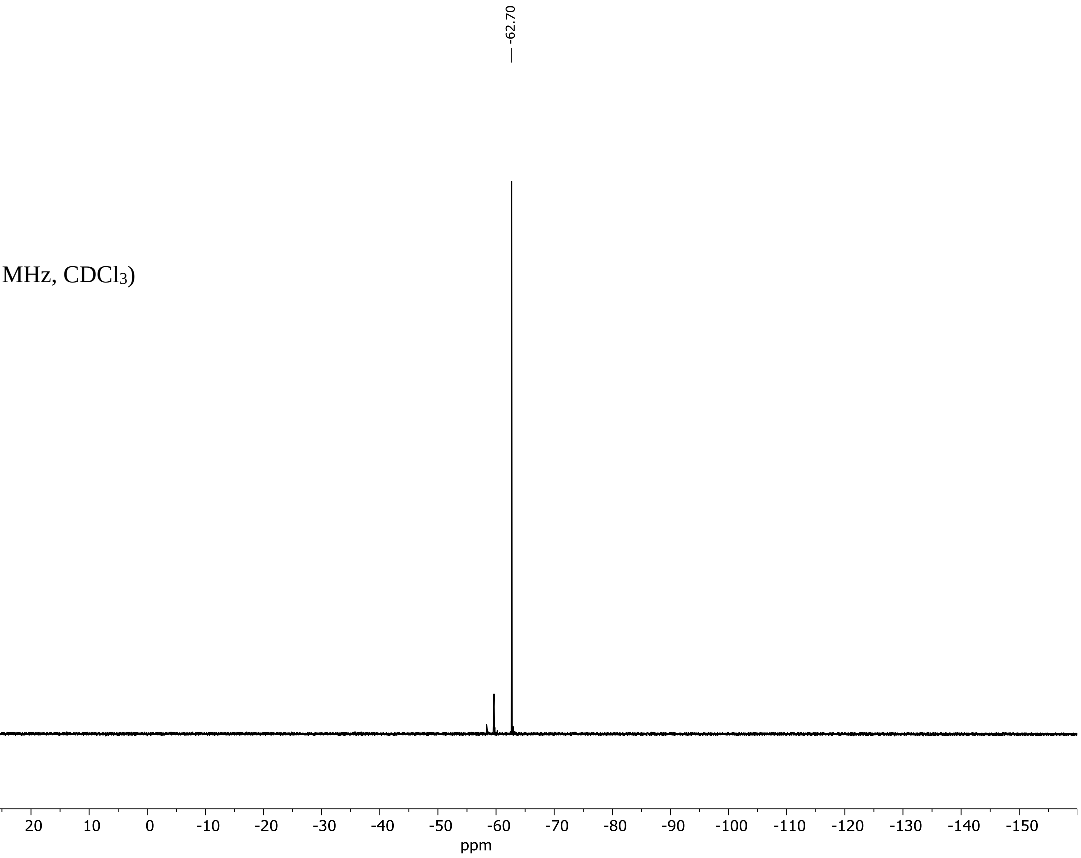

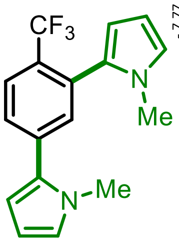

4g-o,p

^{1}H NMR (400 MHz, $CDCl_3$)

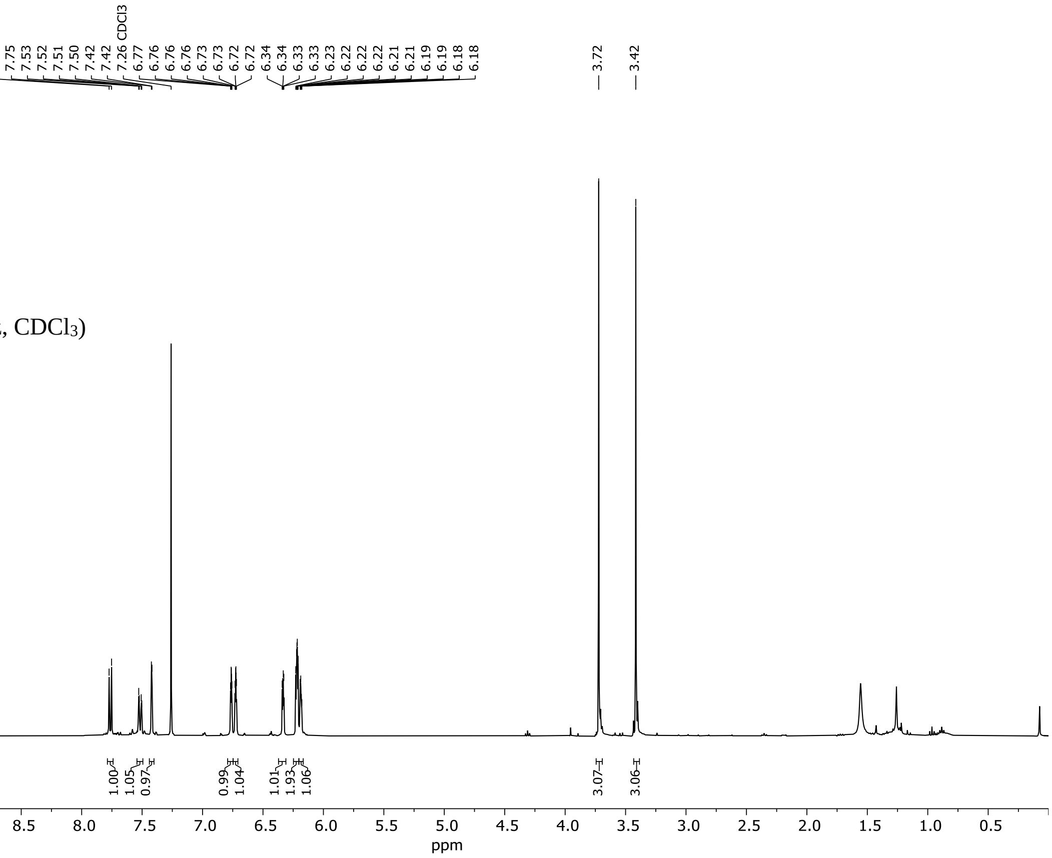

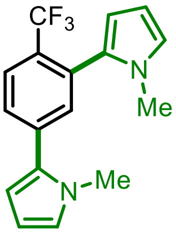

4g-o,p

^{13}C NMR (101 MHz, $CDCl_3$)

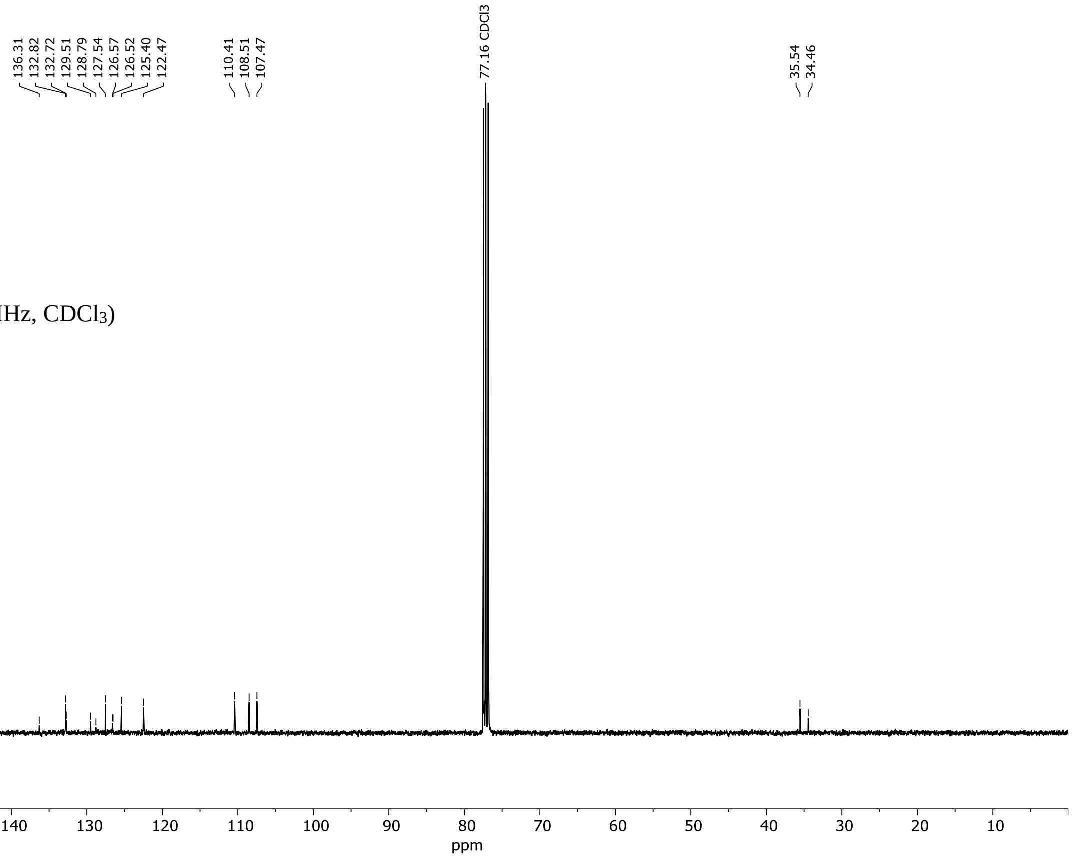

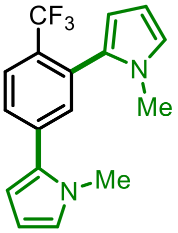

4g-o,p

^{19}F NMR (376 MHz, $CDCl_3$)

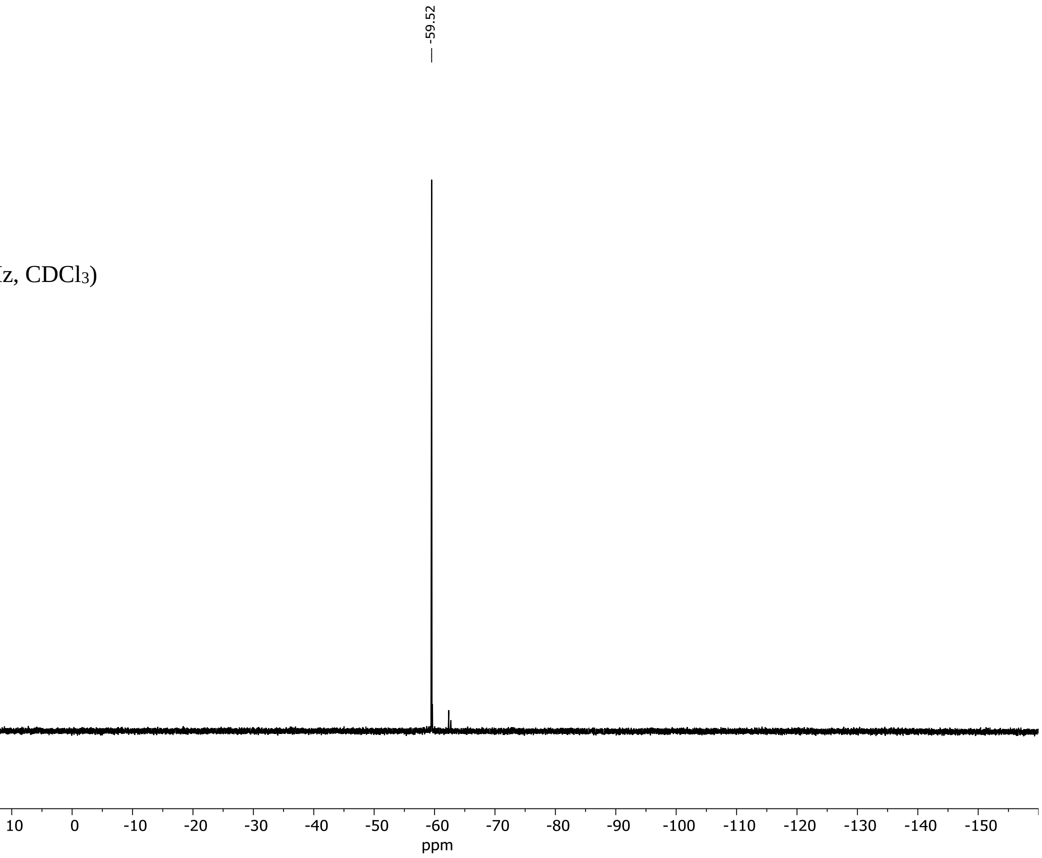

4,4,5,5-tetramethyl-2-(2-(trifluoromethyl)phenyl)-1,3,2-dioxaborolane 4h

Crude **^{19}F NMR** (377 MHz, $CDCl_3$)

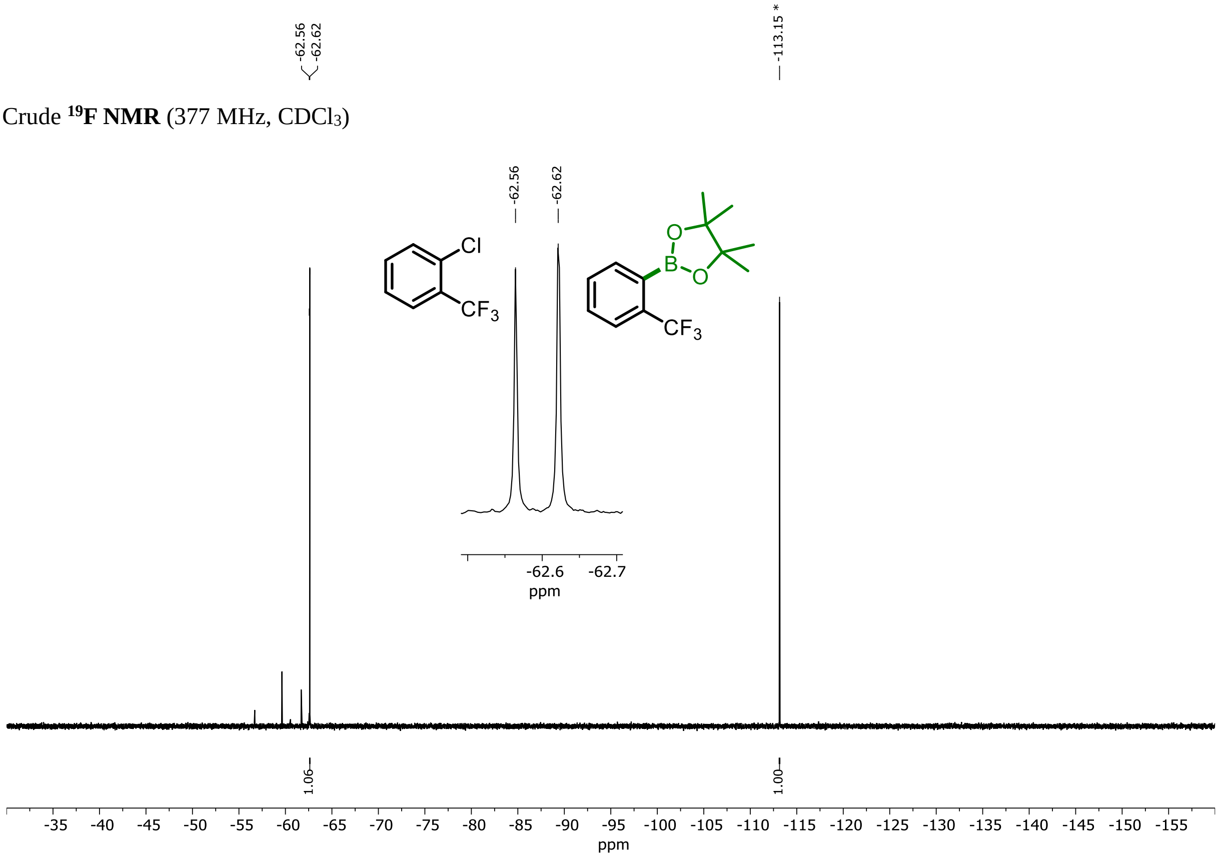

Dimethyl(2-(trifluoromethyl)phenyl)phosphonate (4i)

Crude ^{19}**F NMR** (377 MHz, $CDCl_3$)

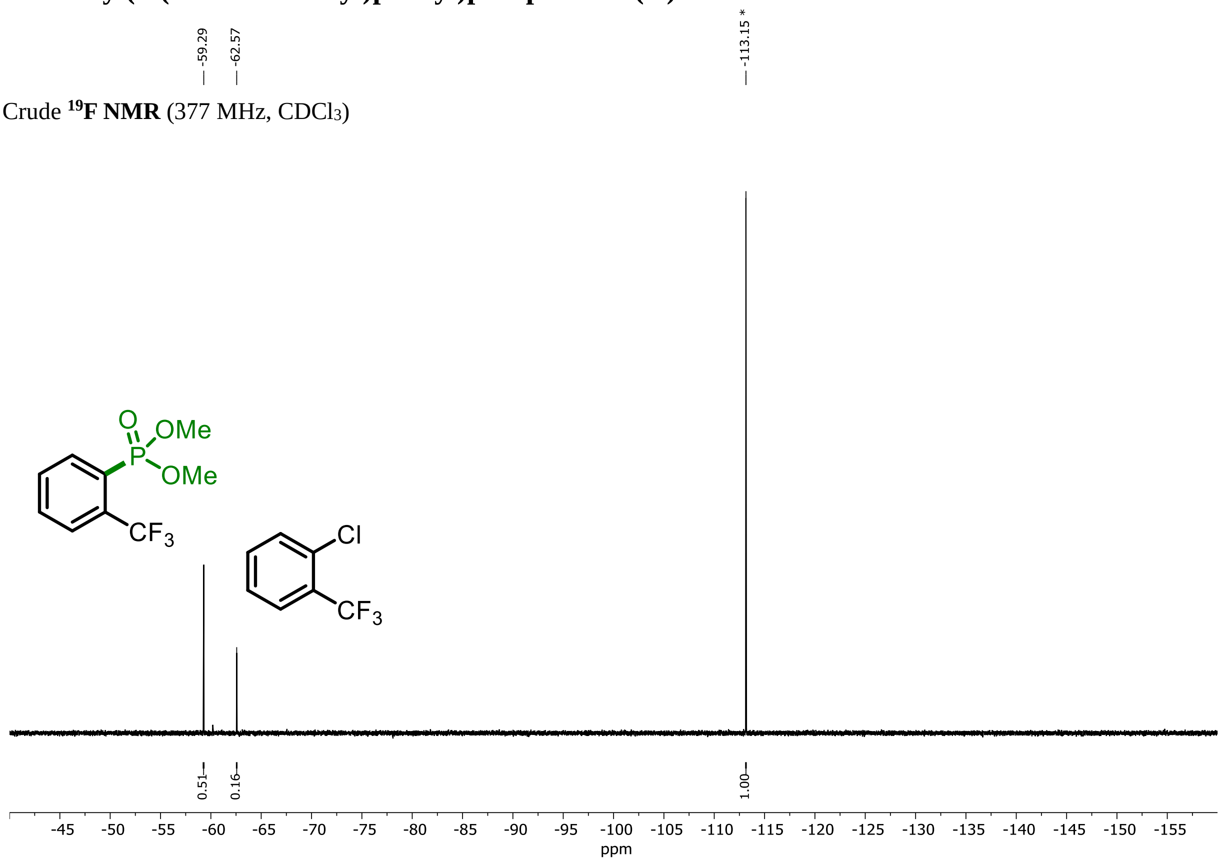

Dimethyl(4-(trifluoromethyl)phenyl)phosphonate 4j

Crude ^{19}**F NMR** (377 MHz, $CDCl_3$)

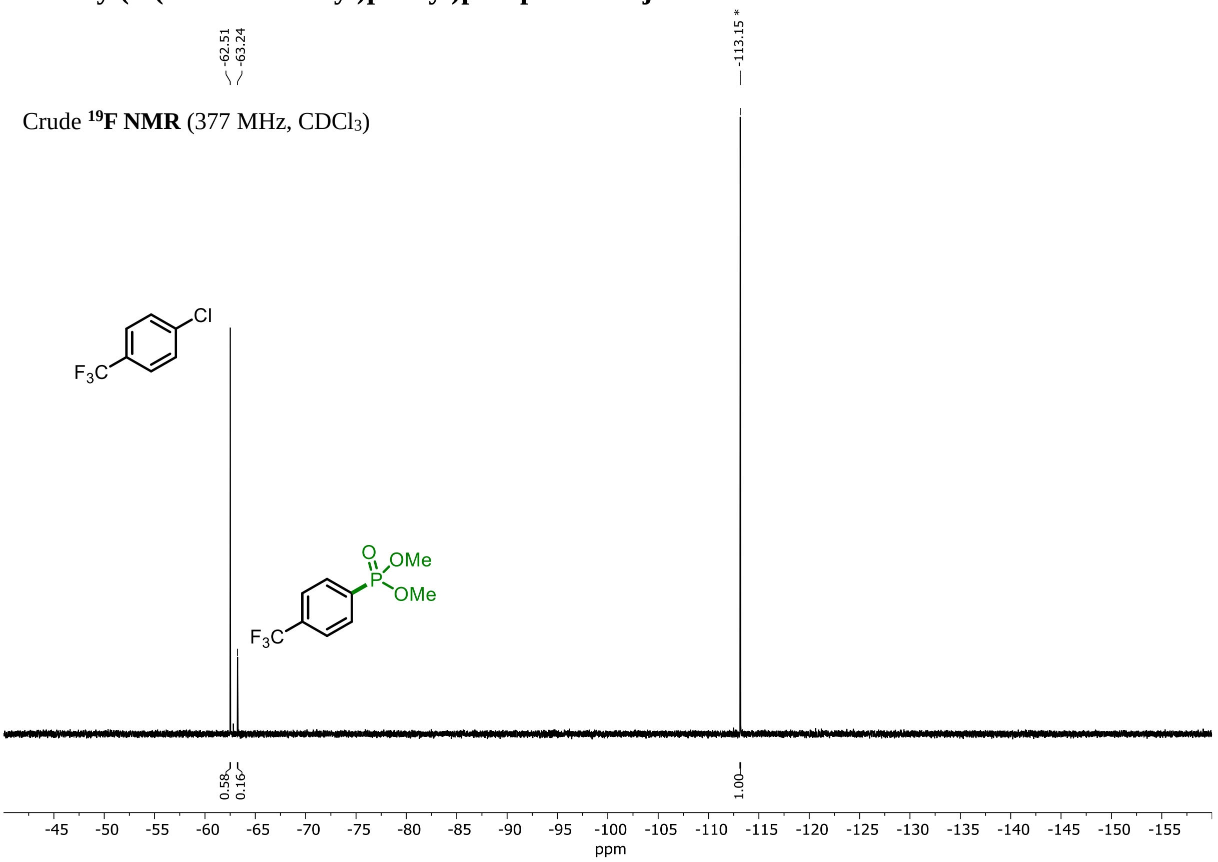

Diethyl (2-(trifluoromethyl)phenyl)phosphonate 4k

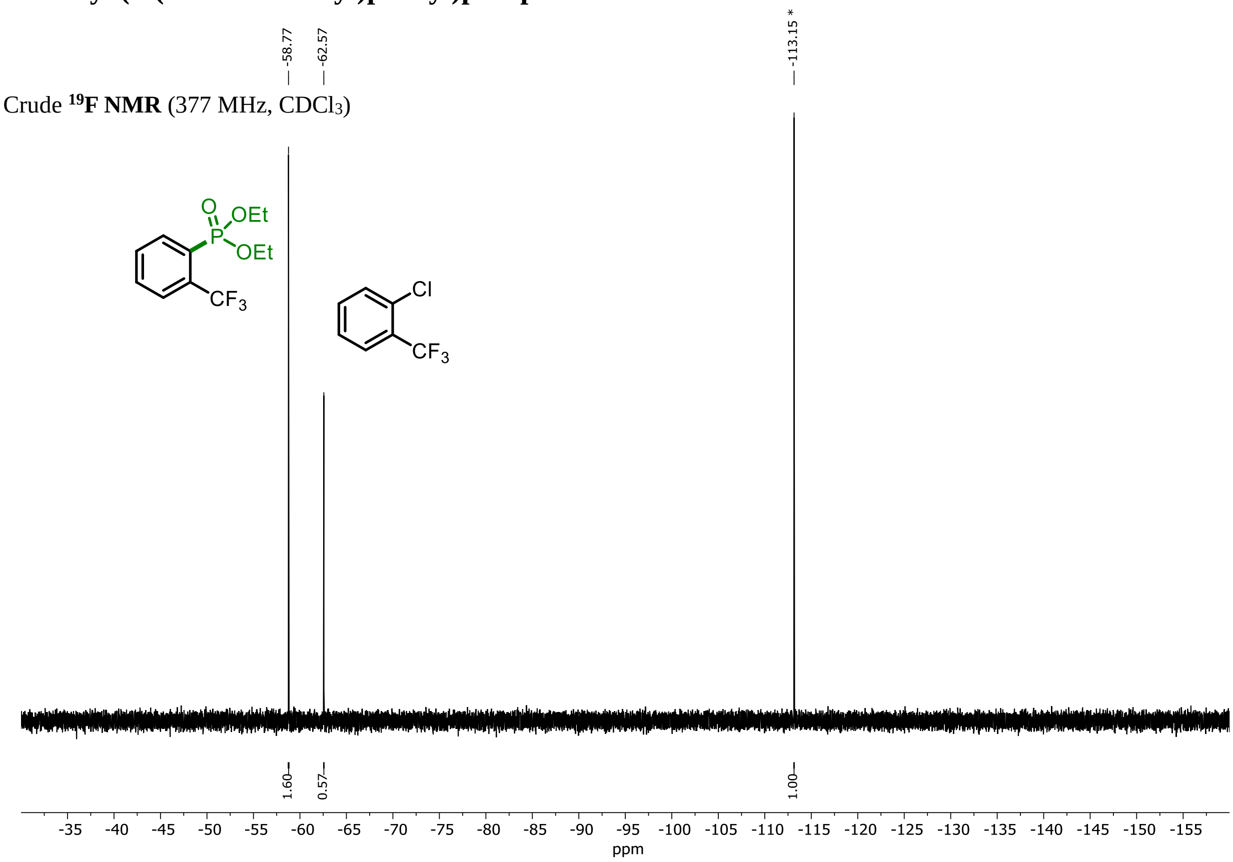

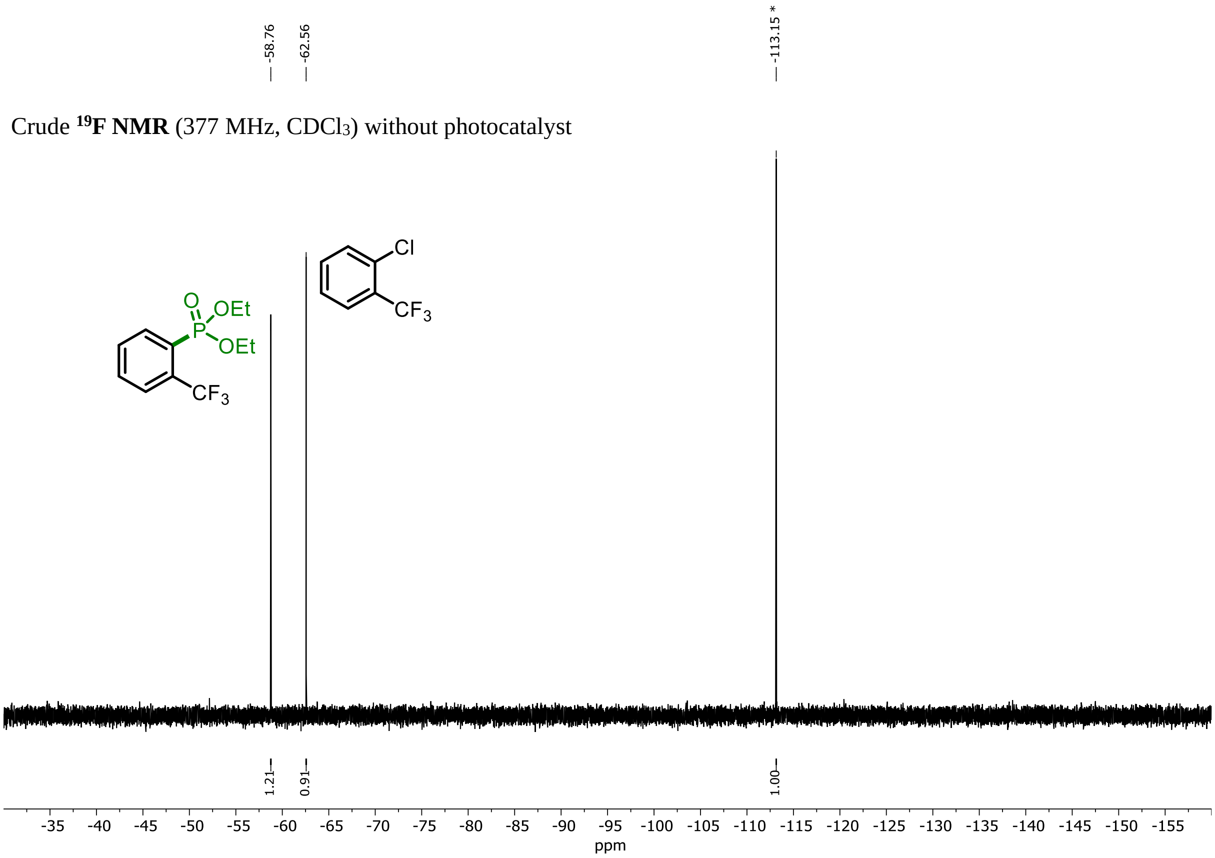

Diethyl (4-(trifluoromethyl)phenyl)phosphonate 4l

Crude ^{19}**F NMR** (377 MHz, $CDCl_3$)

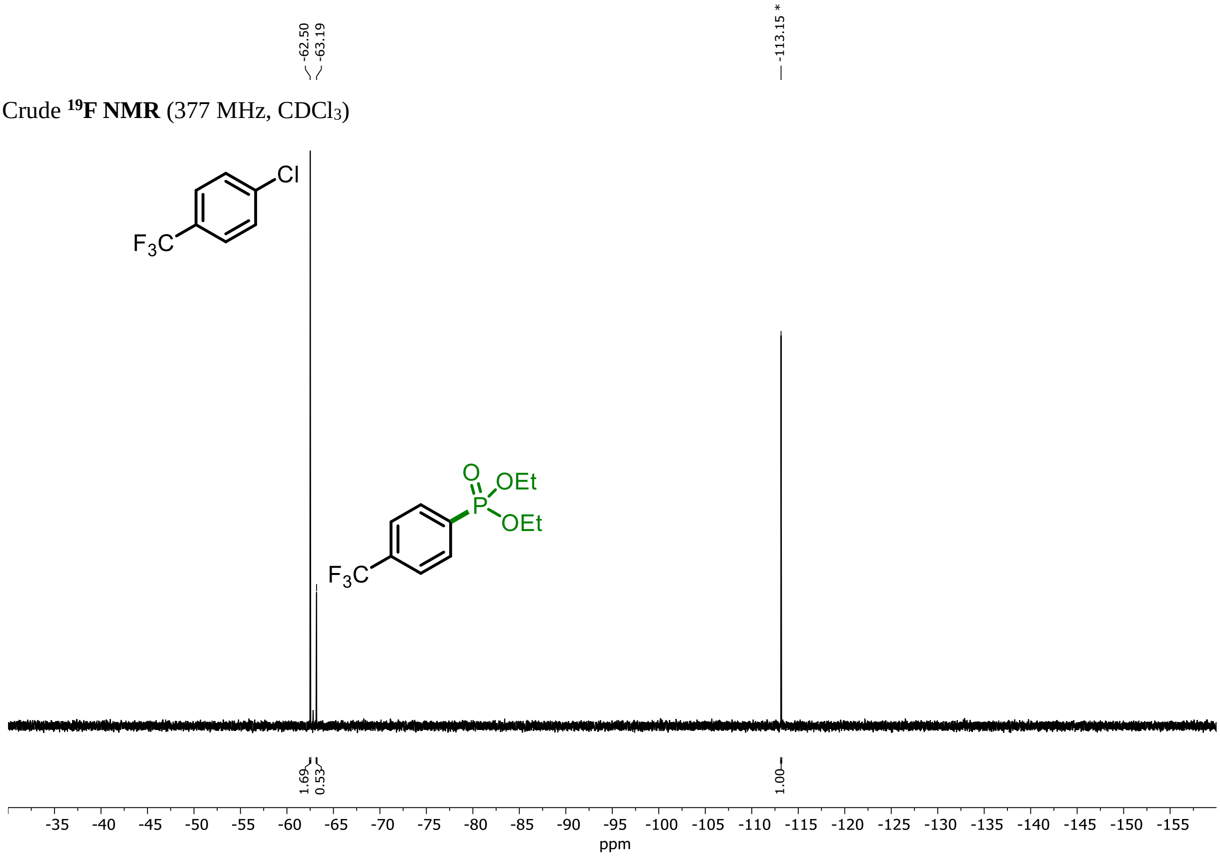

Diisopropyl (2-(trifluoromethyl)phenyl)phosphonate 4m

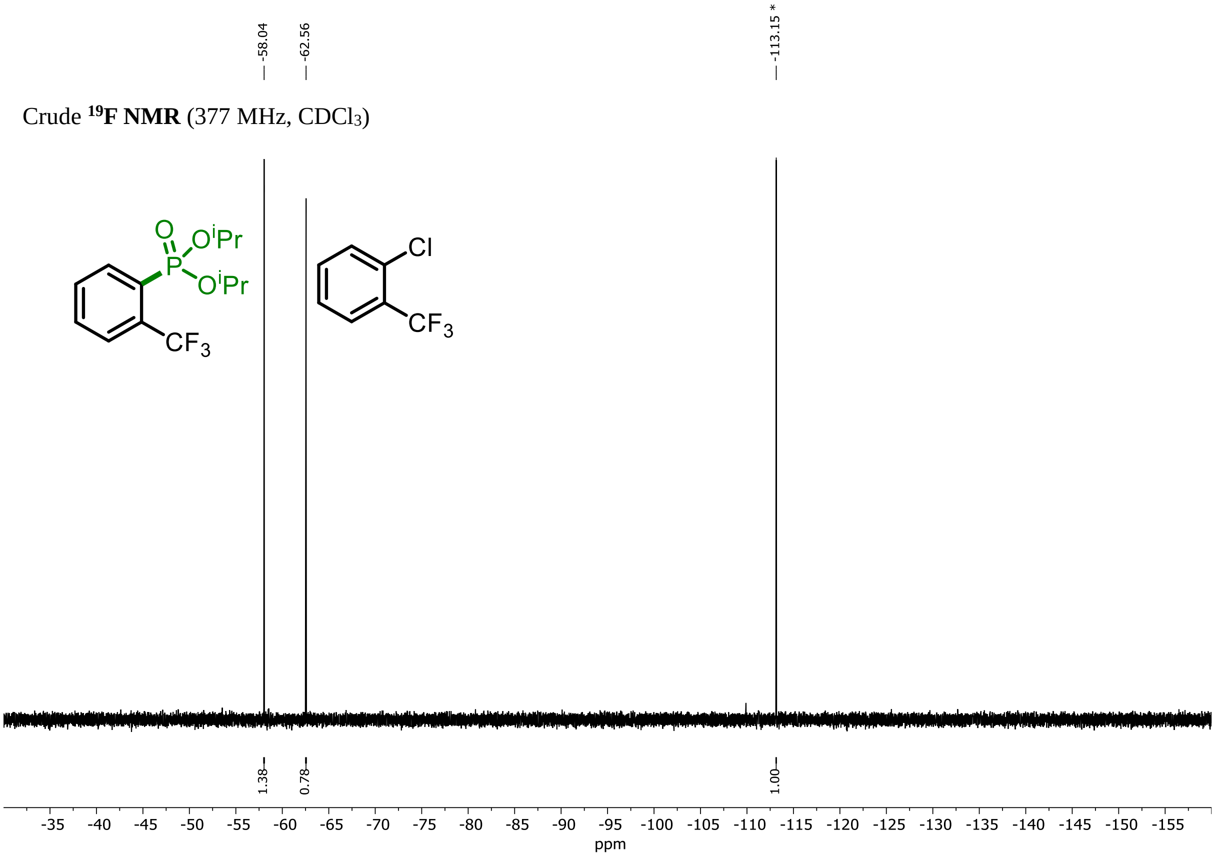

Crude ^{19}F NMR (377 MHz, $CDCl_3$)

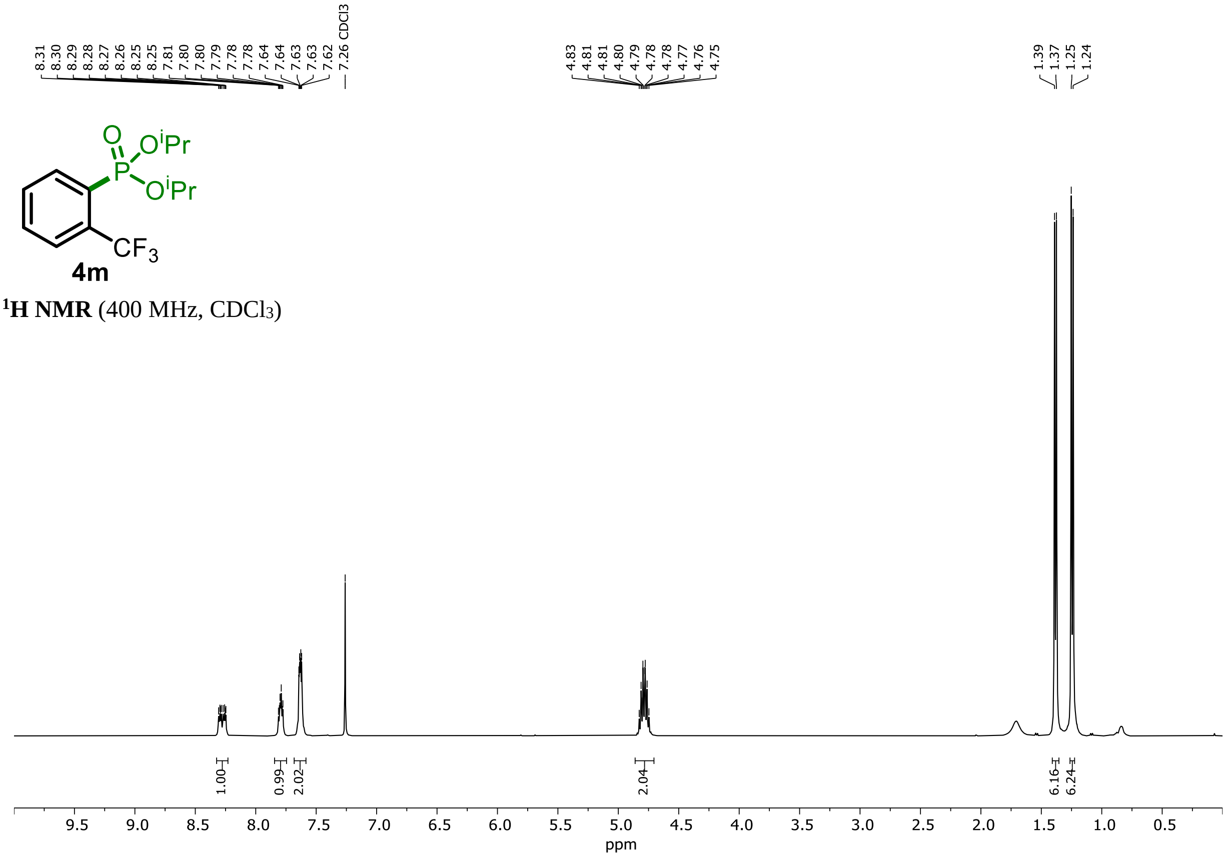

^{1}H NMR (400 MHz, $CDCl_3$)

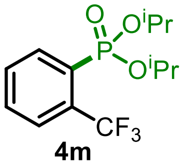

4m

^{13}**C NMR** (101 MHz, $CDCl_3$)

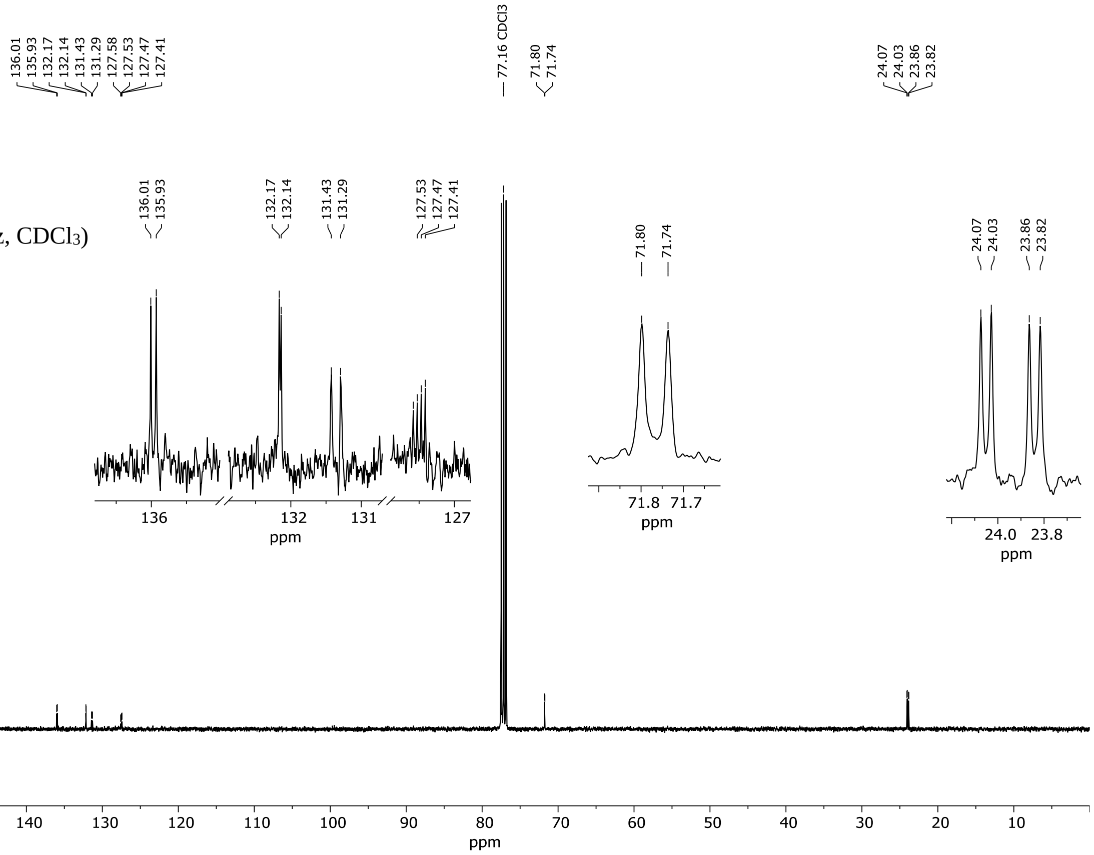

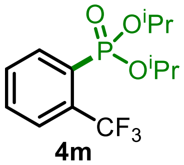

4m

^{19}**F NMR** (377 MHz, $CDCl_3$)

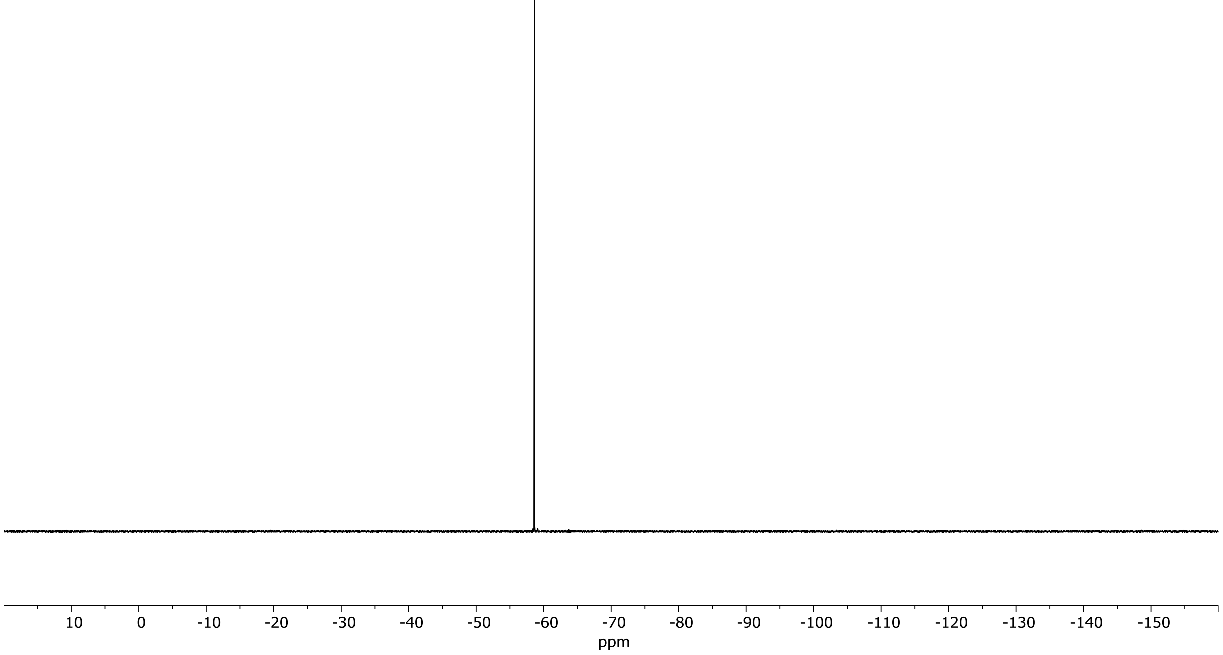

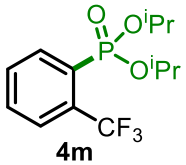

4m

^{31}P NMR (162 MHz, $CDCl_3$)

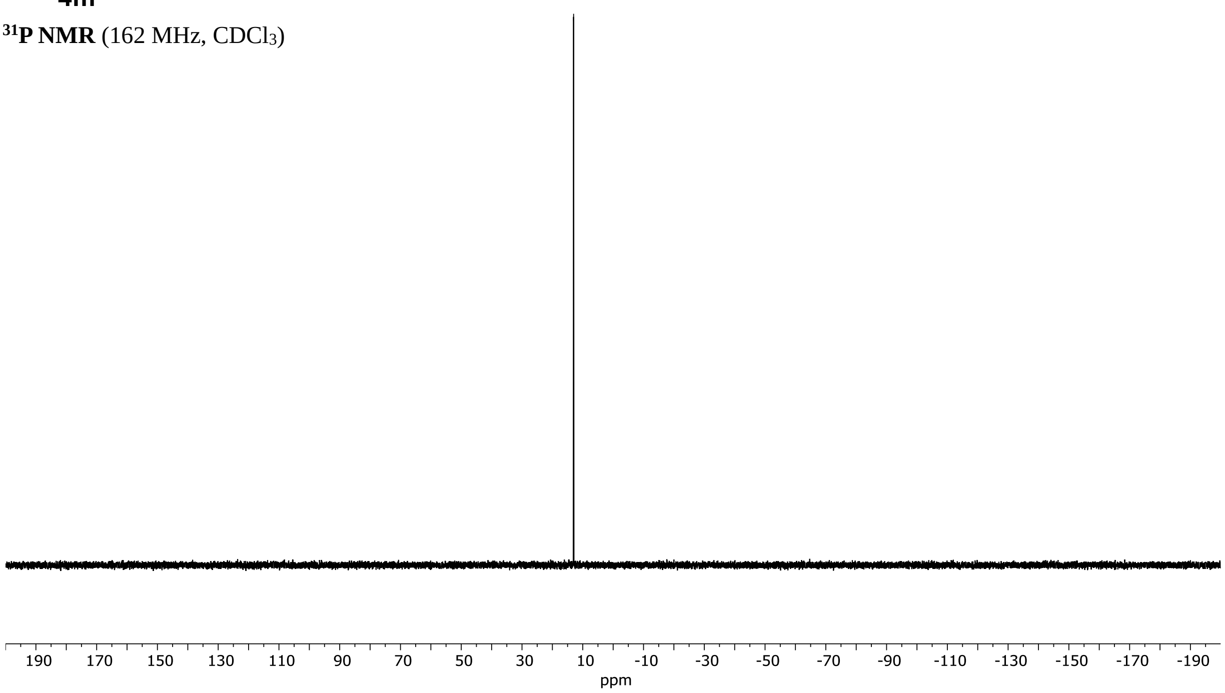

Diisopropyl (4-(trifluoromethyl)phenyl)phosphonate 4n

Crude ^{19}F NMR (377 MHz, $CDCl_3$)

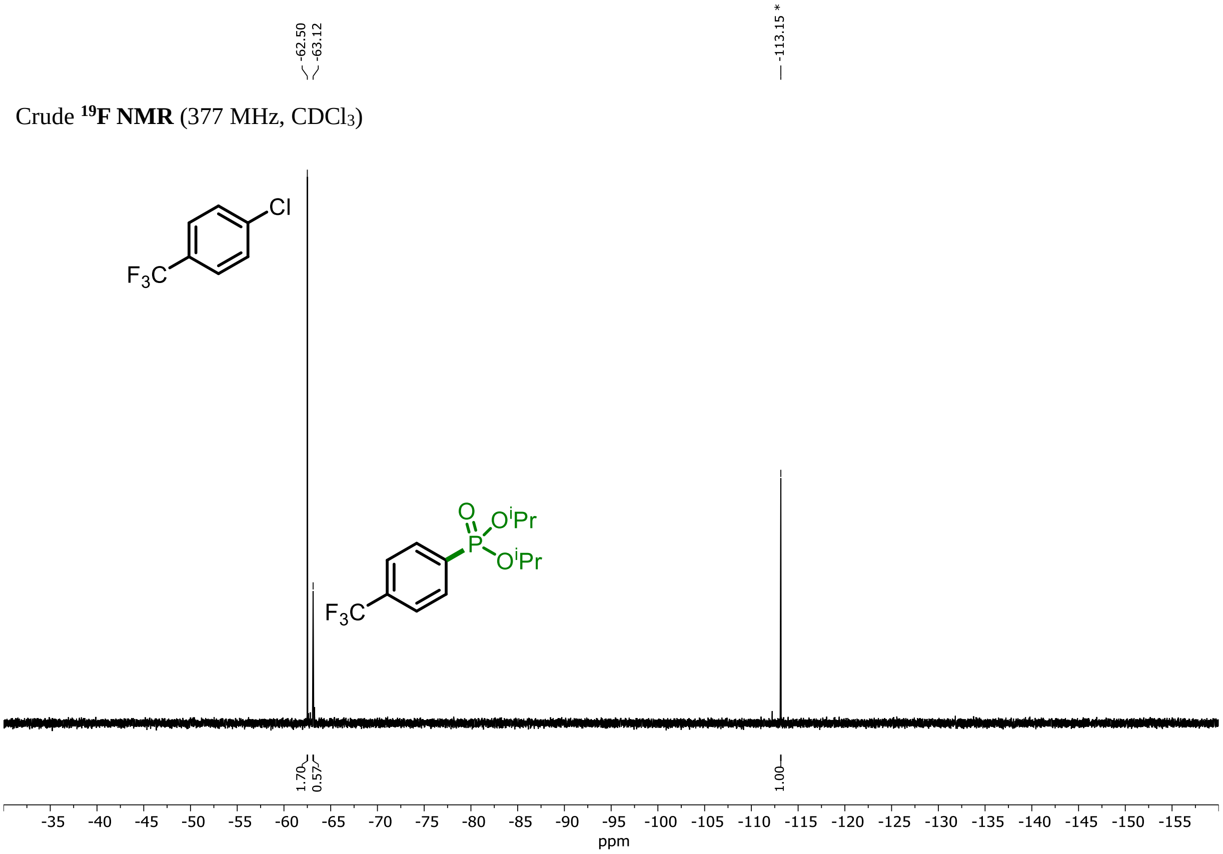

Diphenyl (2-(trifluoromethyl)phenyl)phosphonate 4o

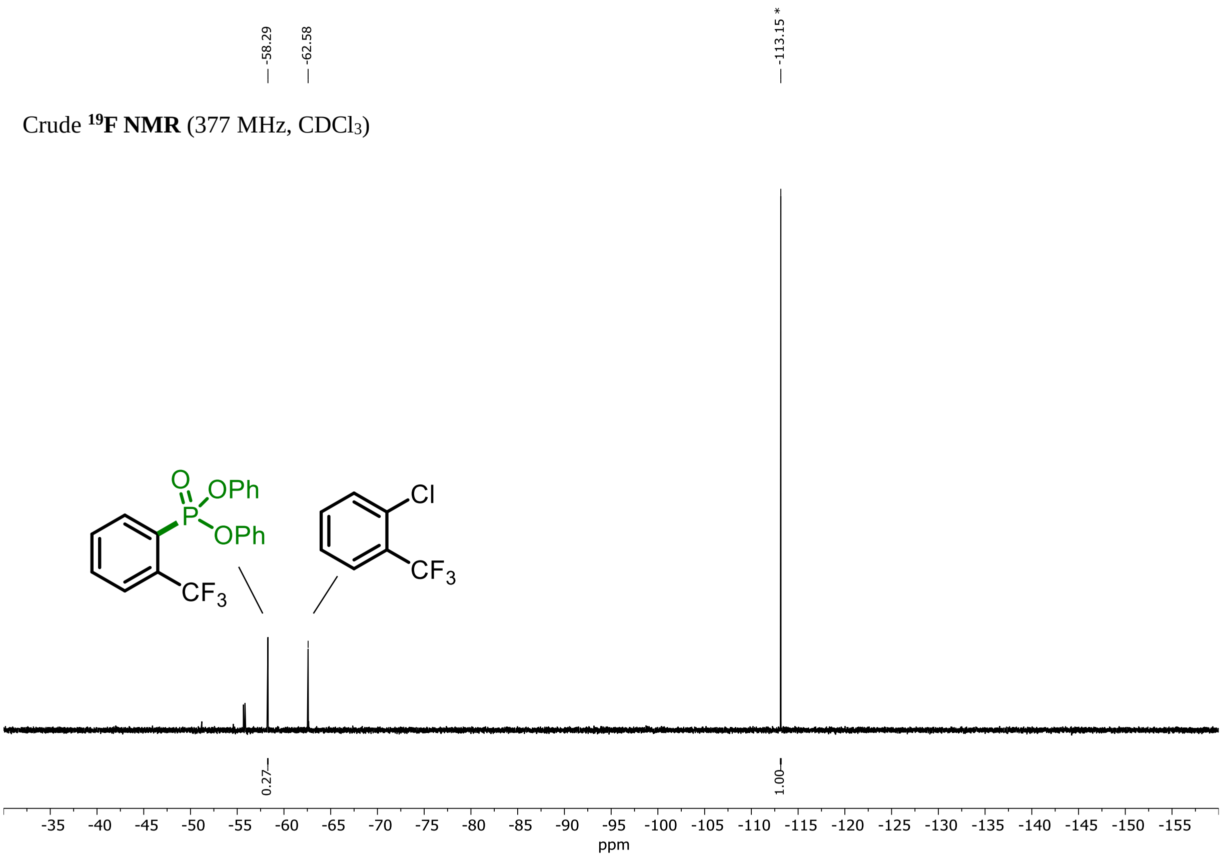

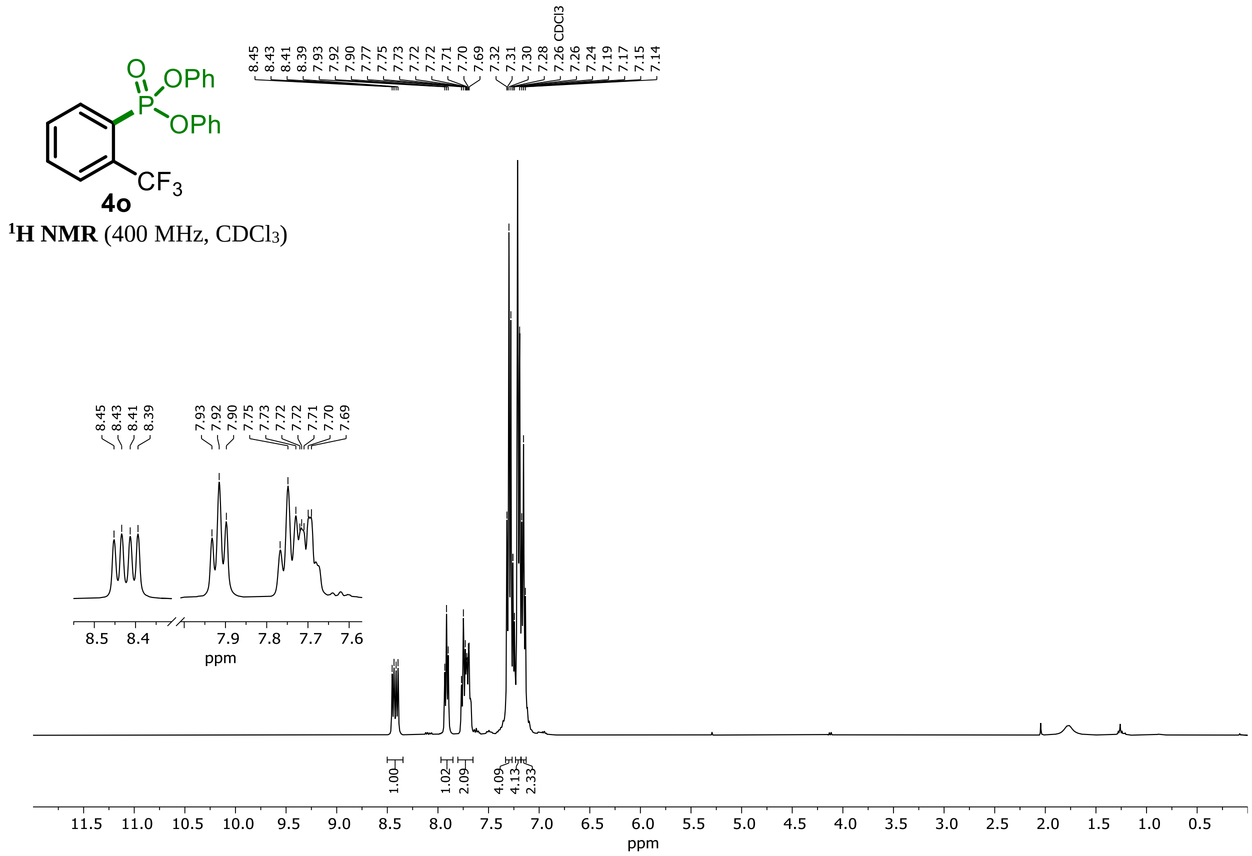

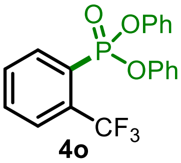

^{13}C NMR (101 MHz, $CDCl_3$)

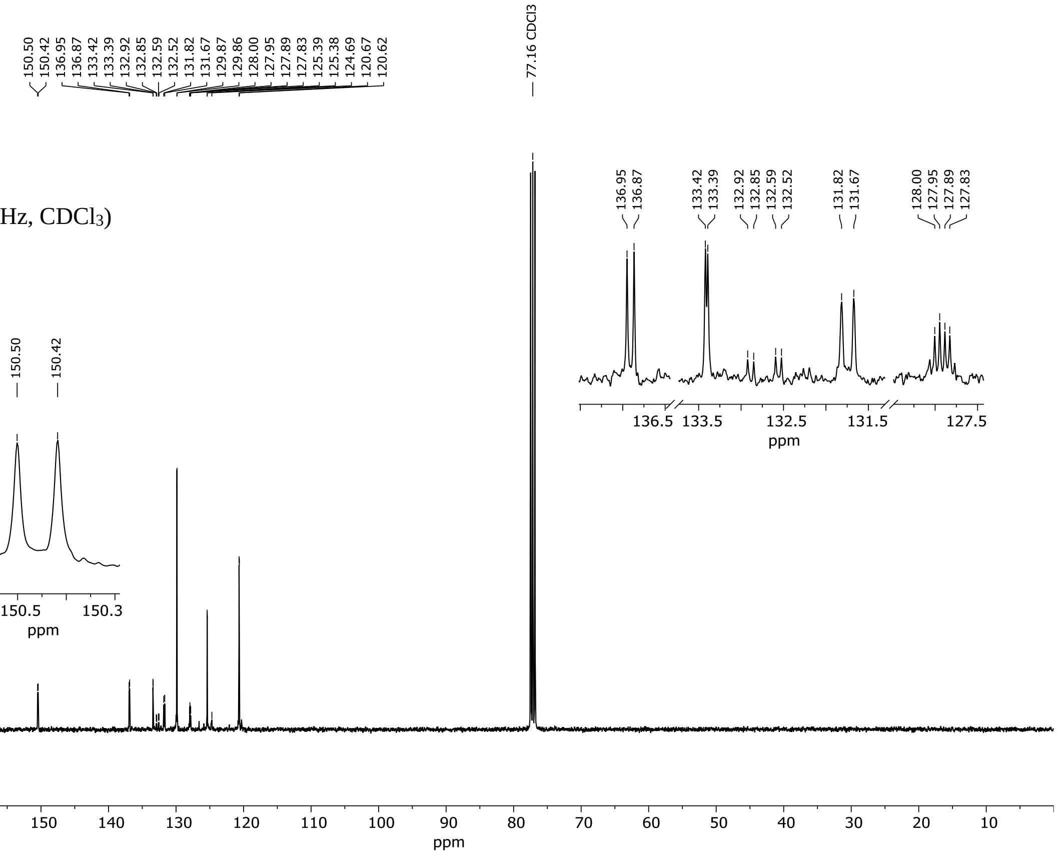

^{19}F NMR (377 MHz, $CDCl_3$)

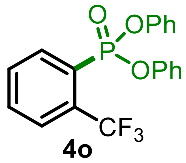

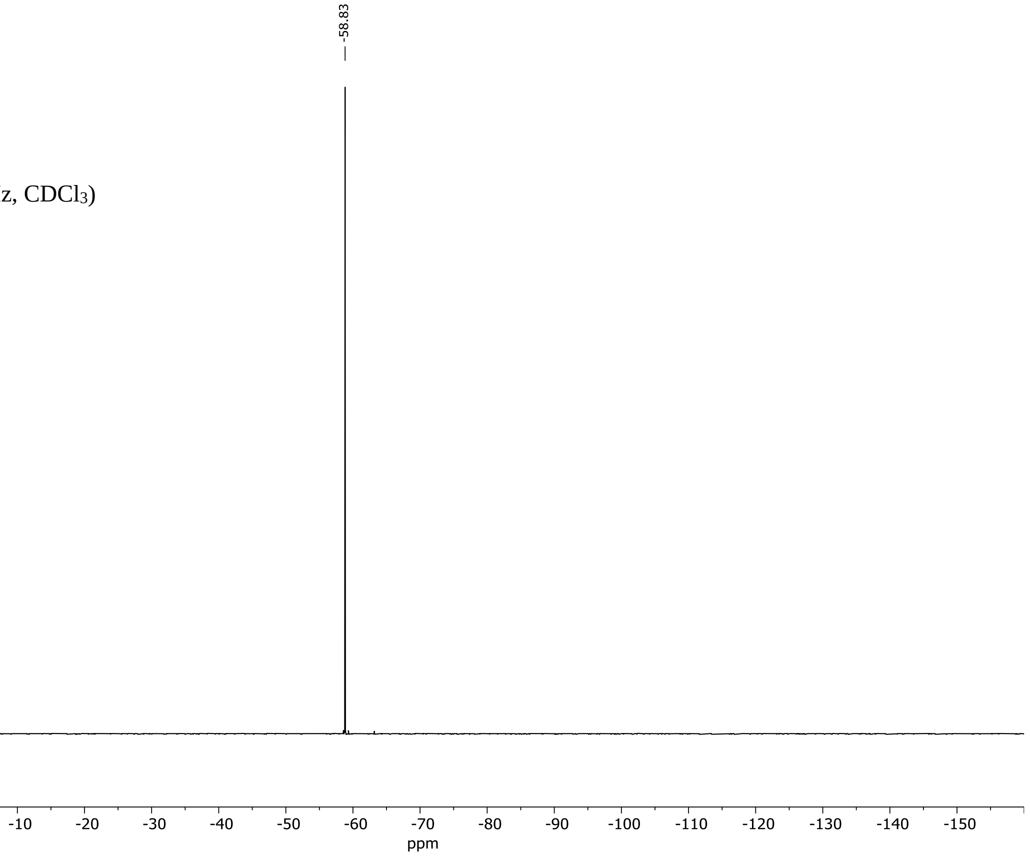

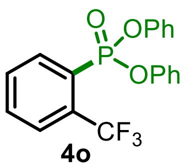

^{31}P NMR (162 MHz, $CDCl_3$)

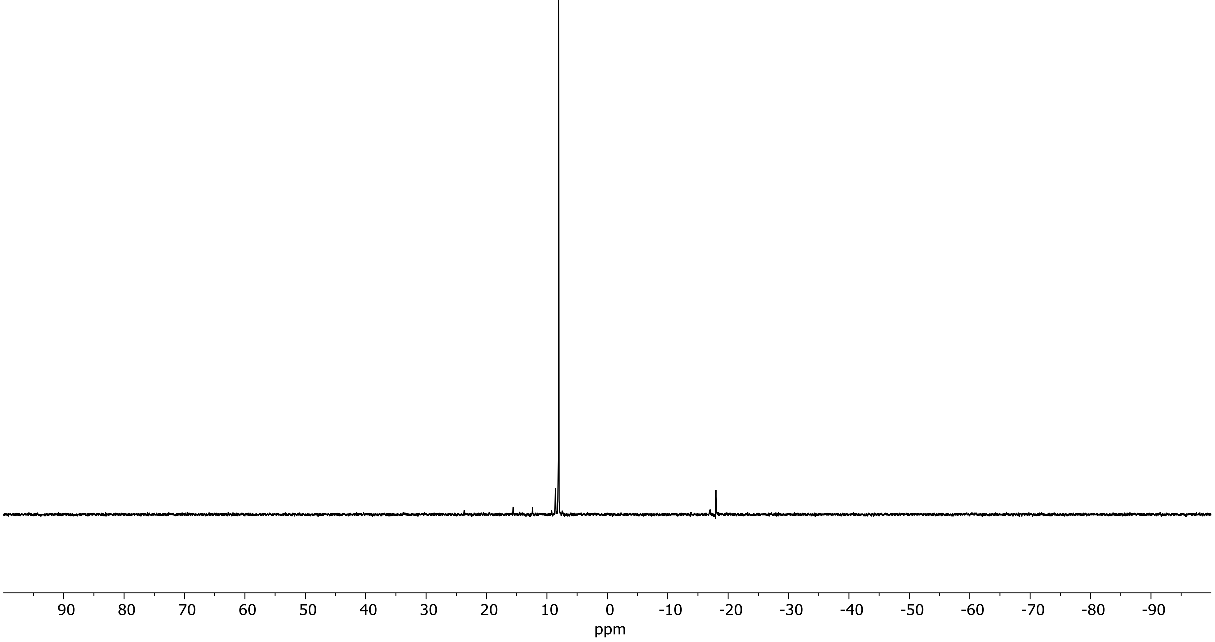

Diethyl (4-methoxyphenyl)phosphonate 4p

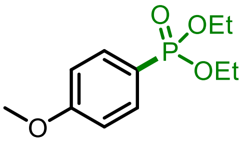

Crude ^{31}**P NMR** (162 MHz, $CDCl_3$)

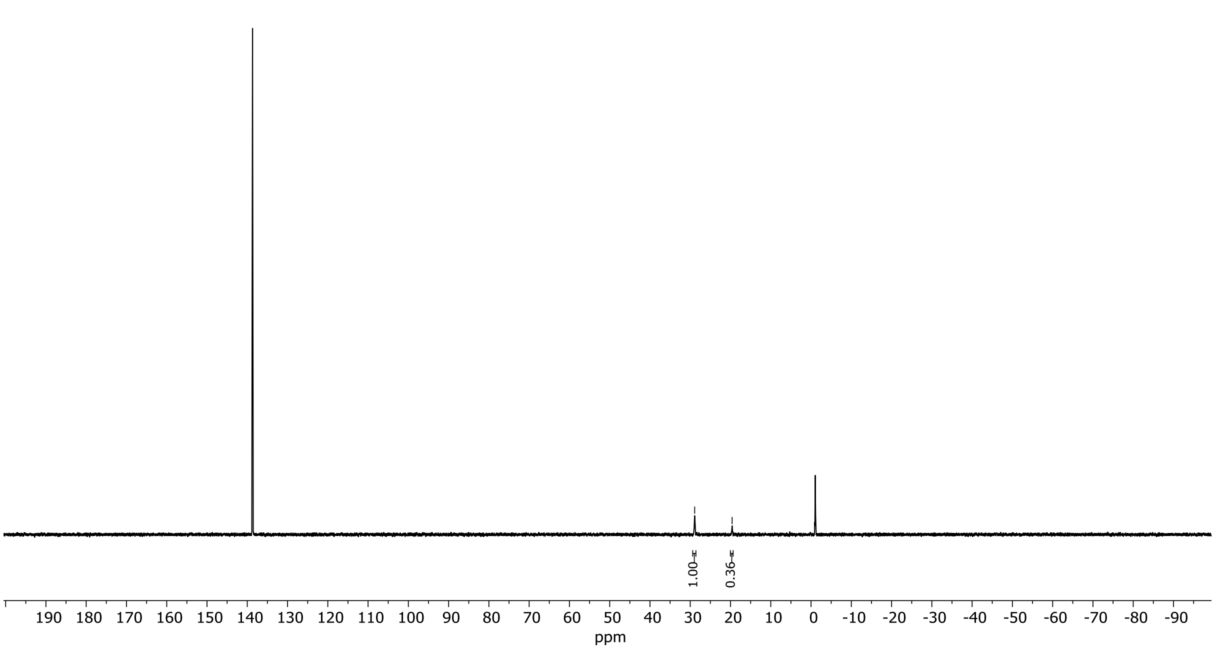

Tetraisopropyl (4-(trifluoromethyl)-1,3-phenylene)bis(phosphonate) and Diisopropyl (5-chloro-2-(trifluoromethyl)phenyl)phosphonate 4q

Crude ^{19}F NMR (376 MHz, $CDCl_3$)

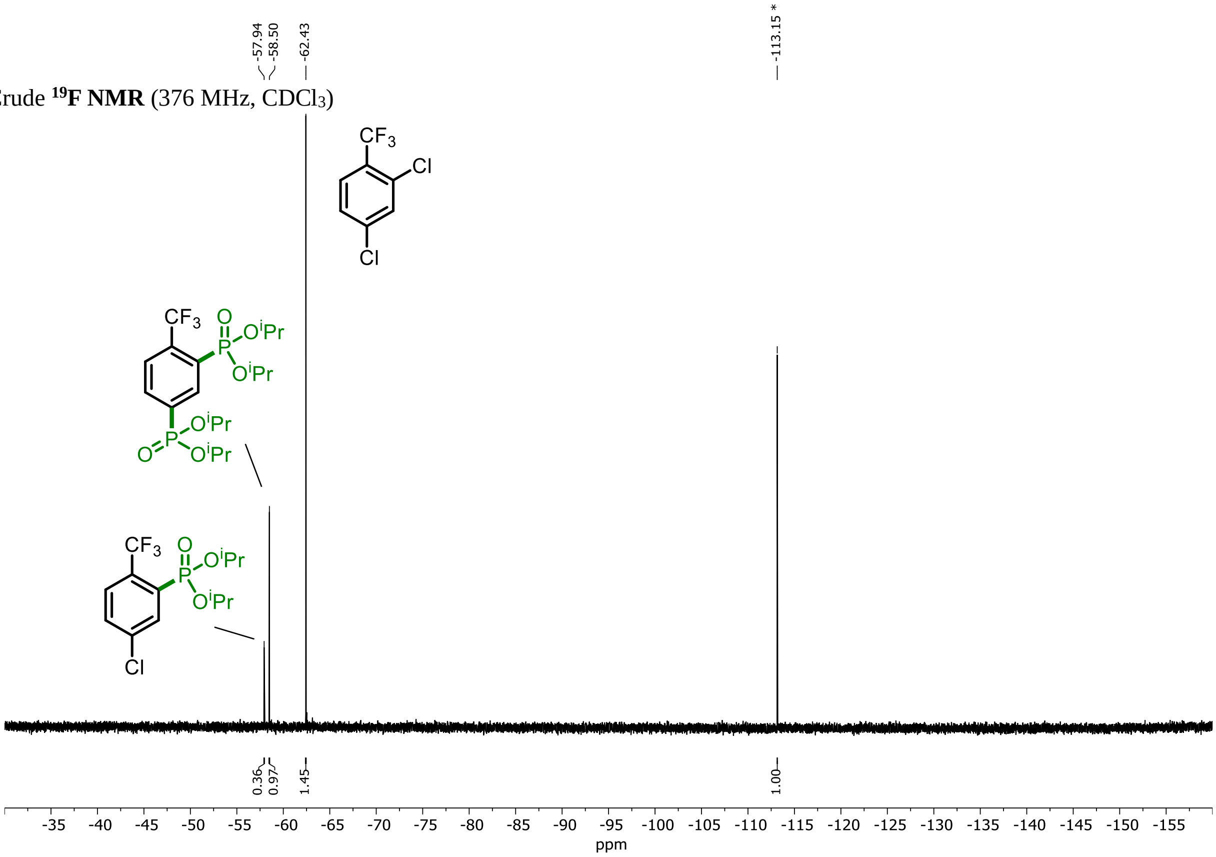

4q-o

^{1}H NMR (400 MHz, $CDCl_3$)

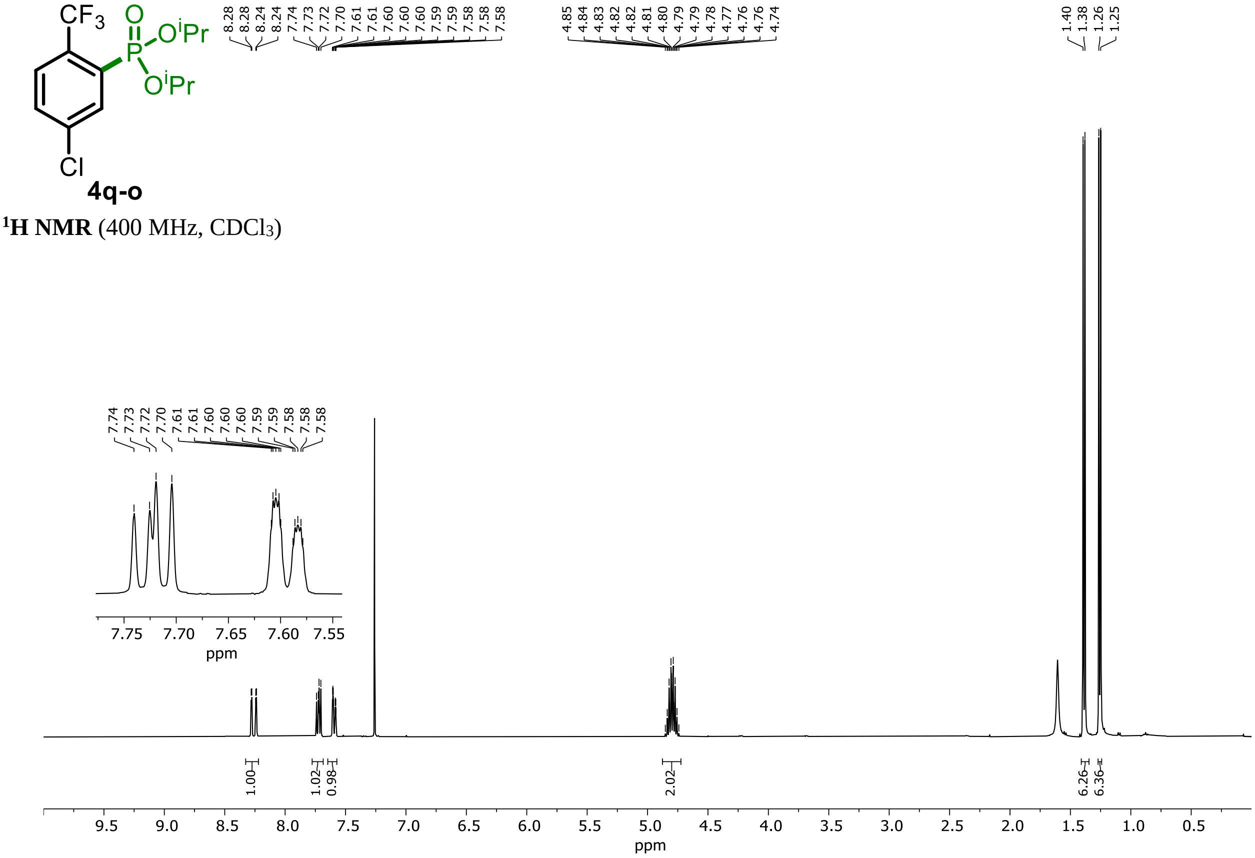

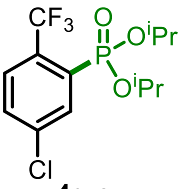

4q-o

^{13}**C NMR** (101 MHz, $CDCl_3$)

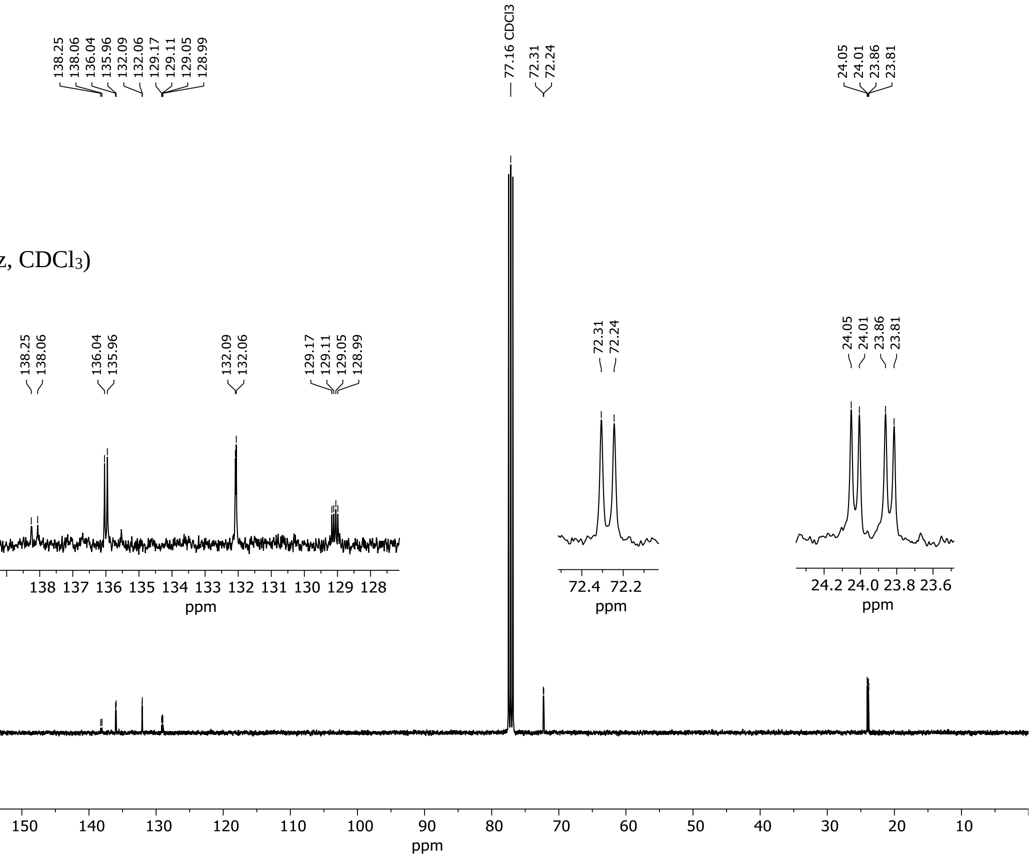

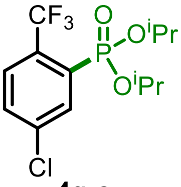

4q-o

^{19}**F NMR** (377 MHz, $CDCl_3$)

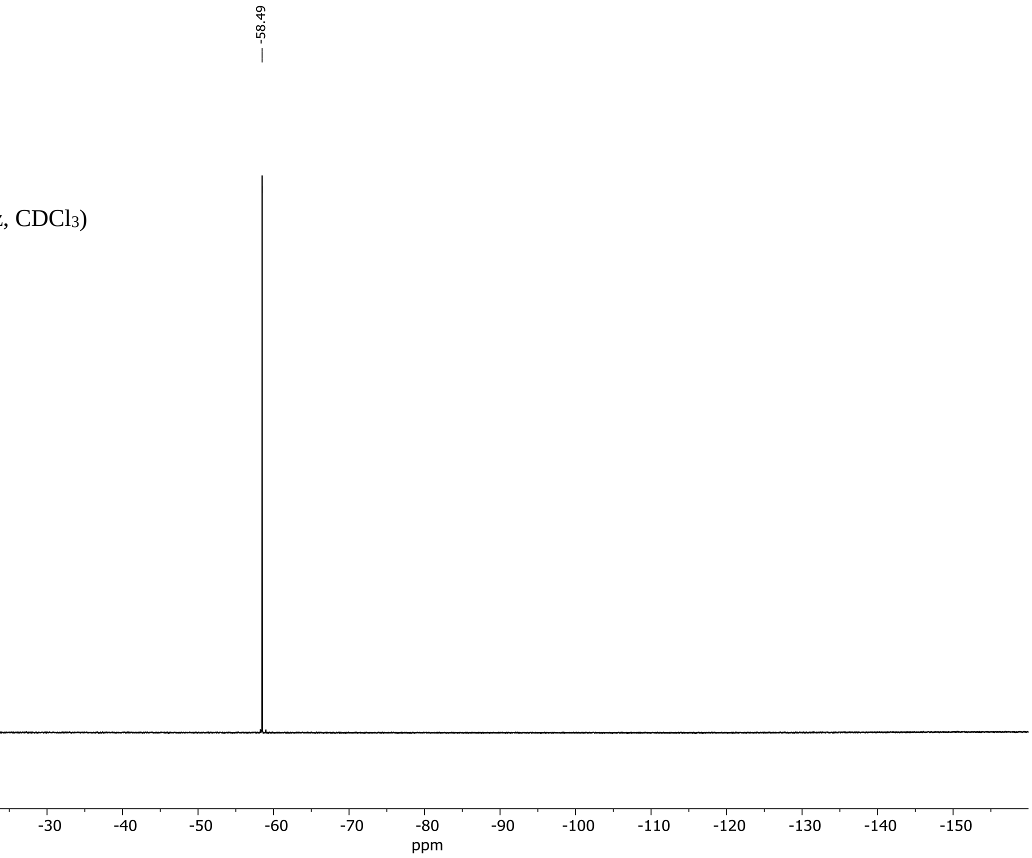

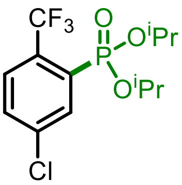

4q-o

^{31}P NMR (166 MHz, CDCl$_3$)

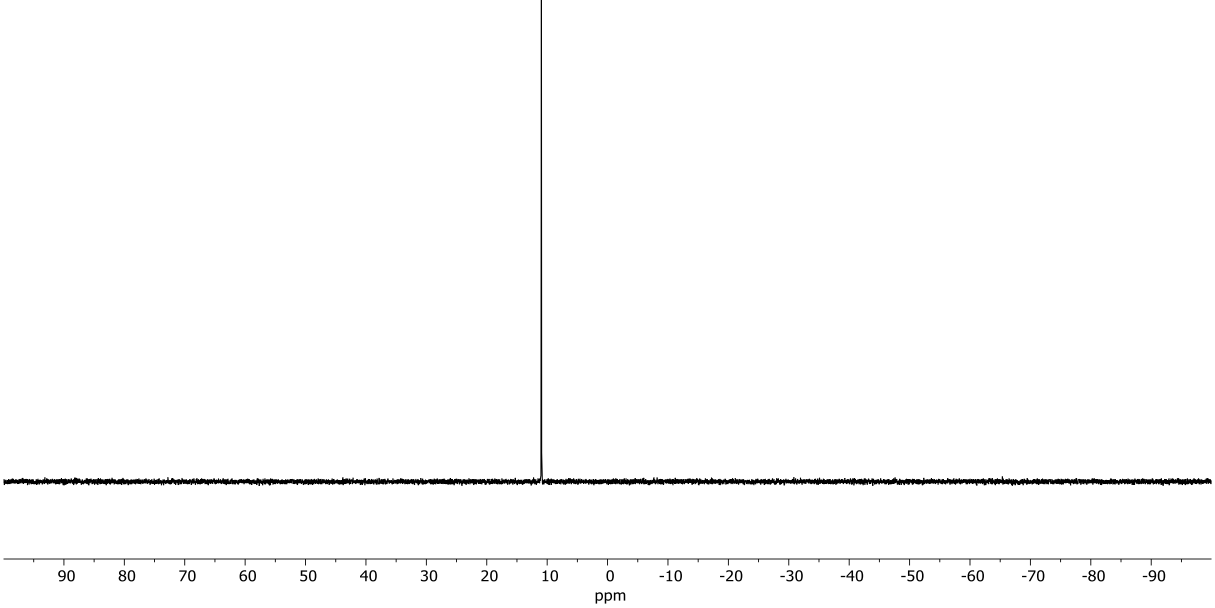

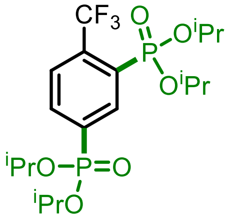

4q-o,p

^{1}H NMR (400 MHz, CDCl$_3$)

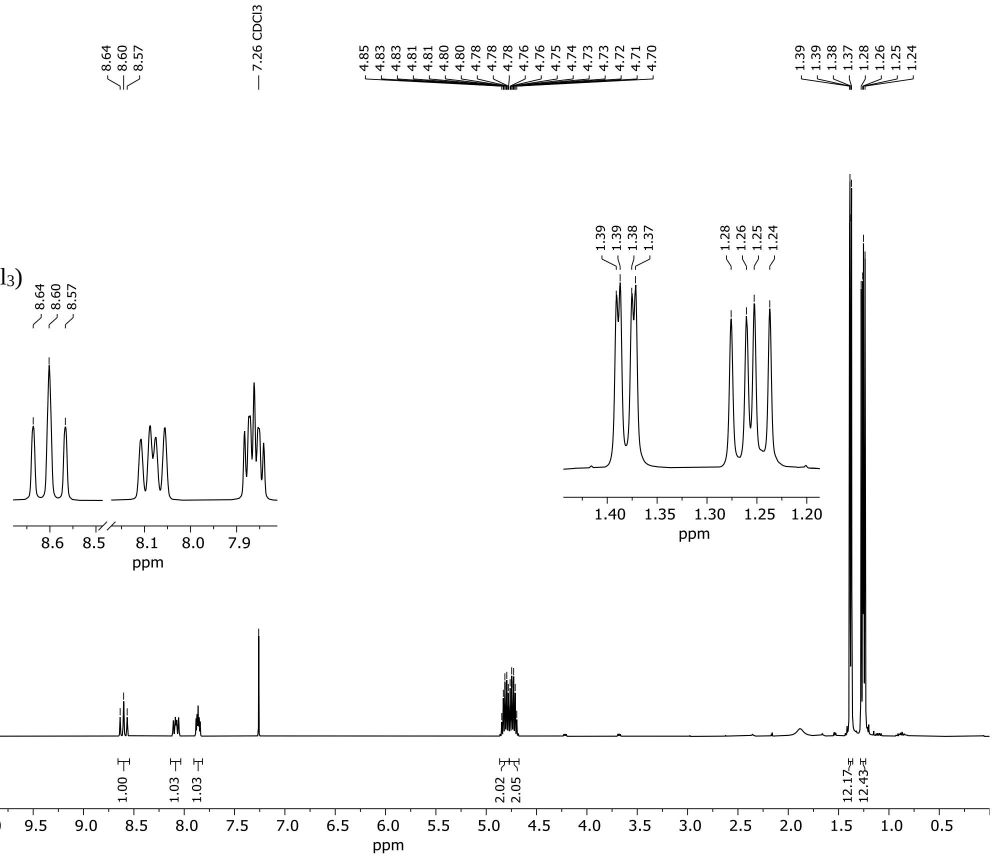

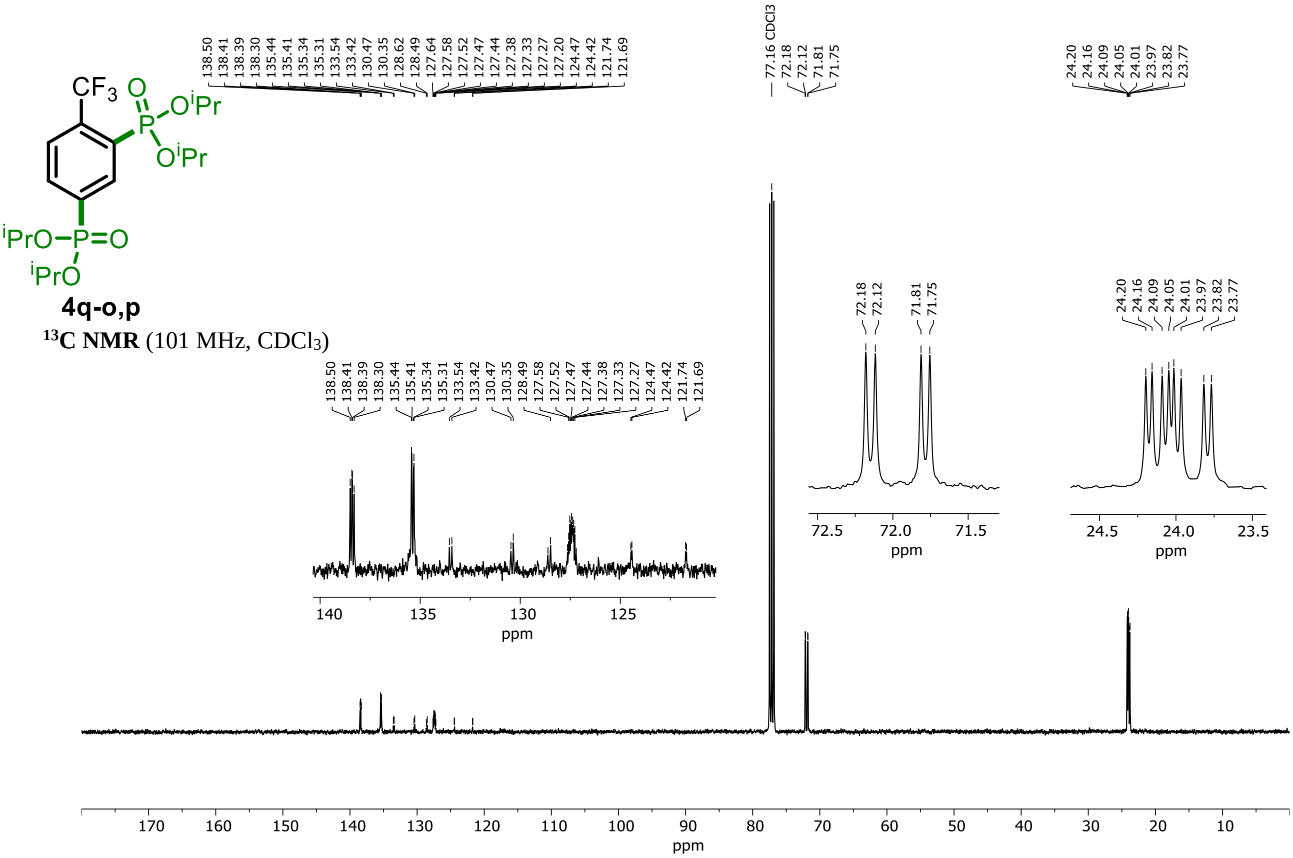

4q-o,p

^{13}C NMR (101 MHz, $CDCl_3$)

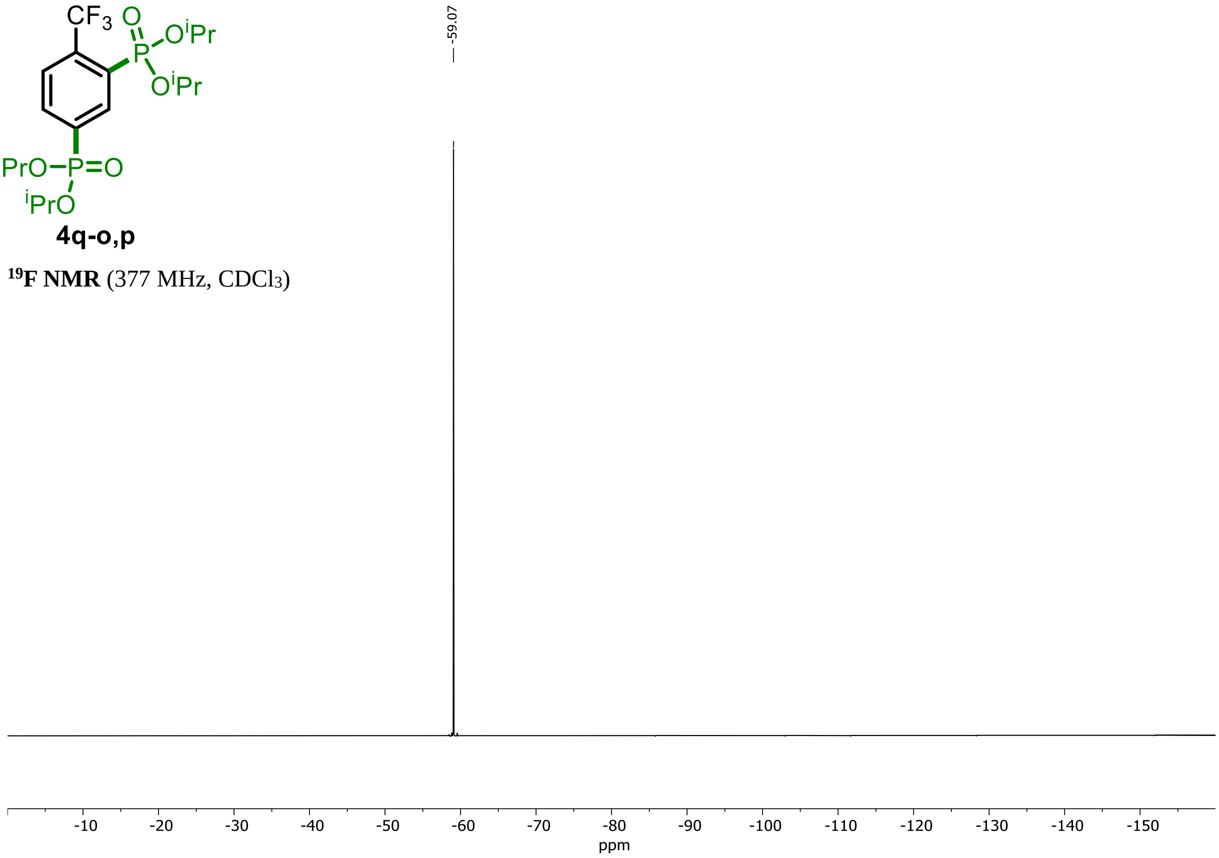

4q-o,p

^{19}F NMR (377 MHz, $CDCl_3$)

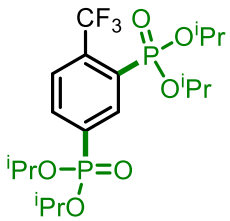

4q-o,p

^{31}P NMR (162 MHz, $CDCl_3$)

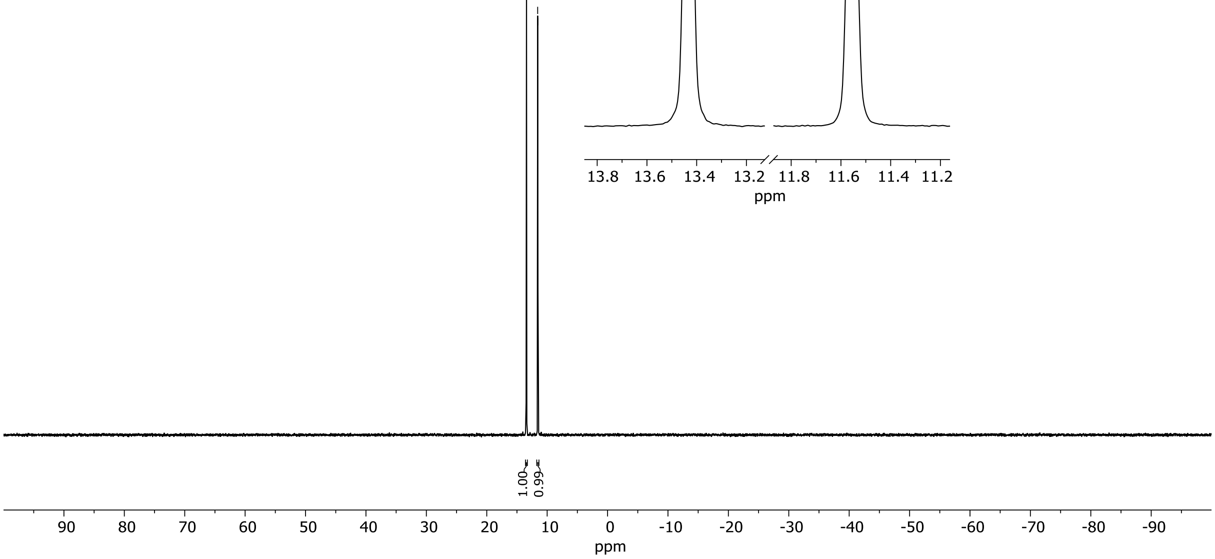

2-(benzo[b]thiophen-2-yl)propan-2-ol 5a

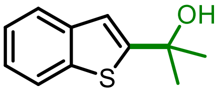

5a

^{1}H NMR (400 MHz, CD_2Cl_2)

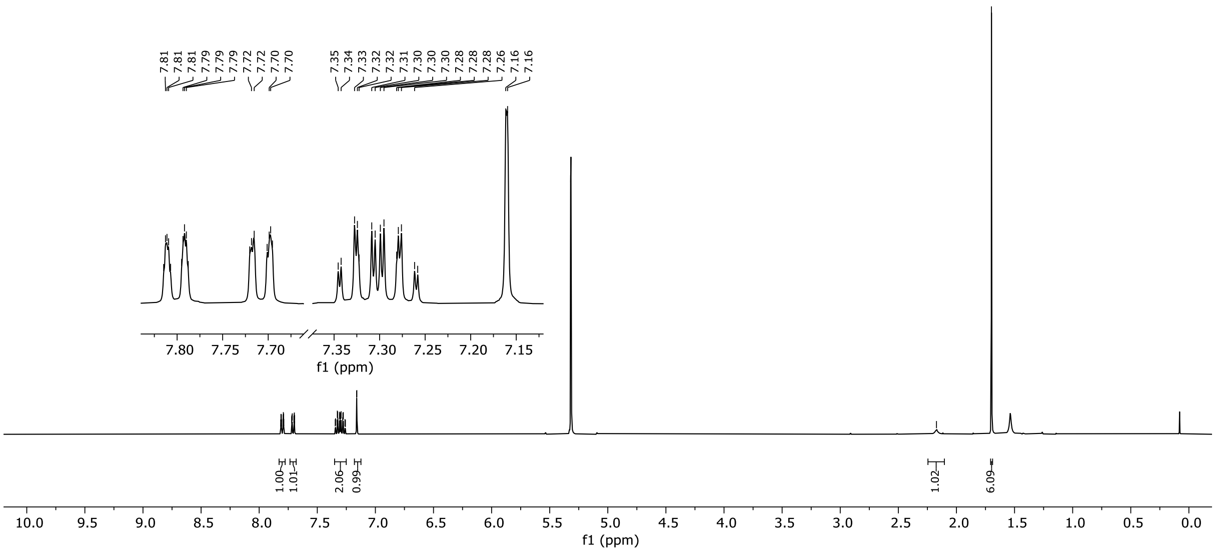

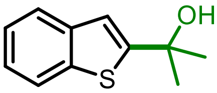

5a

^{13}C NMR (101 MHz, CD_2Cl_2)

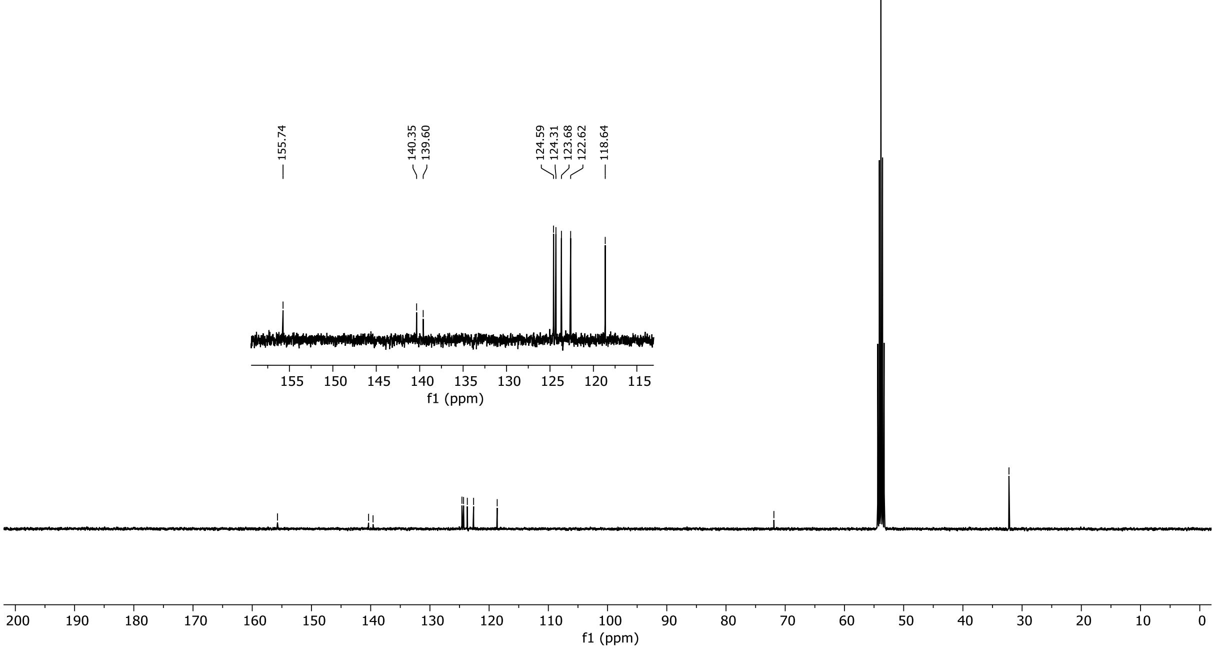

1-(benzo[b]thiophen-2-yl)cyclohexan-1-ol 5b

5b

^{1}H NMR (400 MHz, $CDCl_3$)

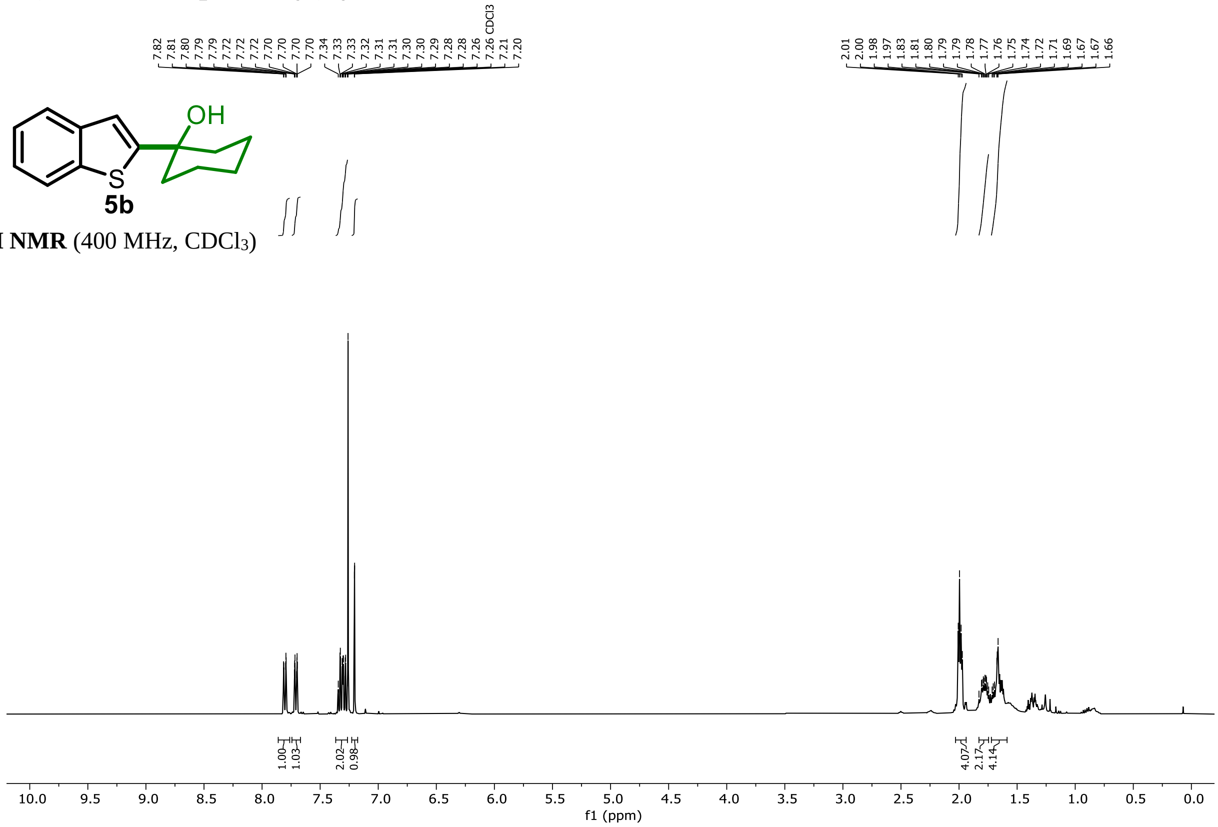

5b

^{13}C NMR (101 MHz, $CDCl_3$)

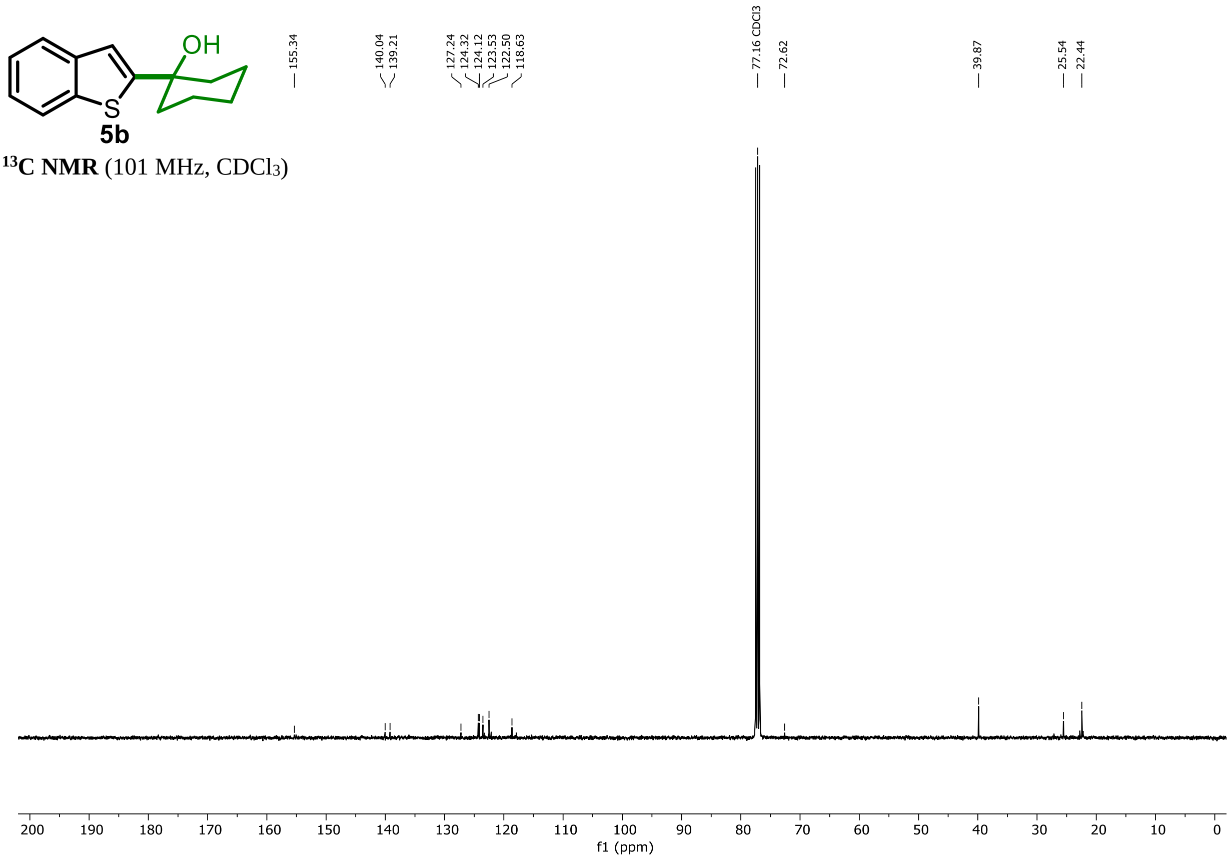

1-(benzo[b]thiophen-2-yl)cyclobutan-1-ol 5c

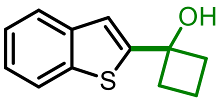

5c

^{1}H NMR (400 MHz, $CDCl_3$)

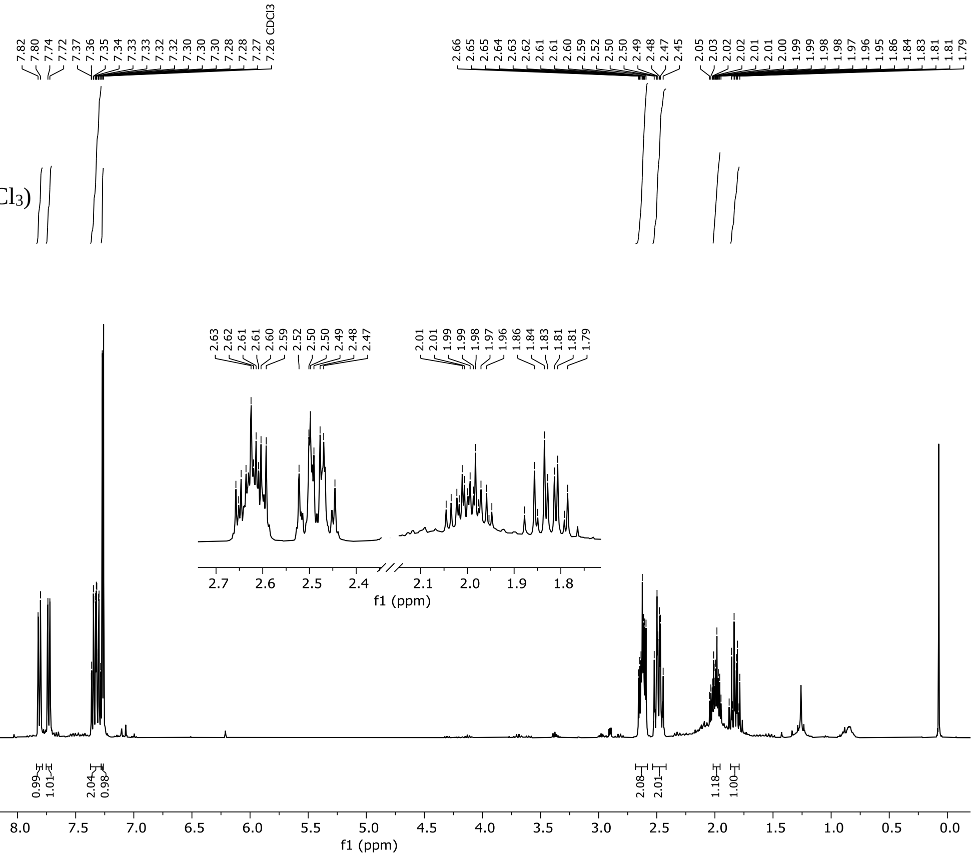

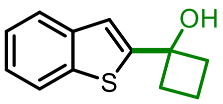

5c

^{13}C NMR (101 MHz, $CDCl_3$)

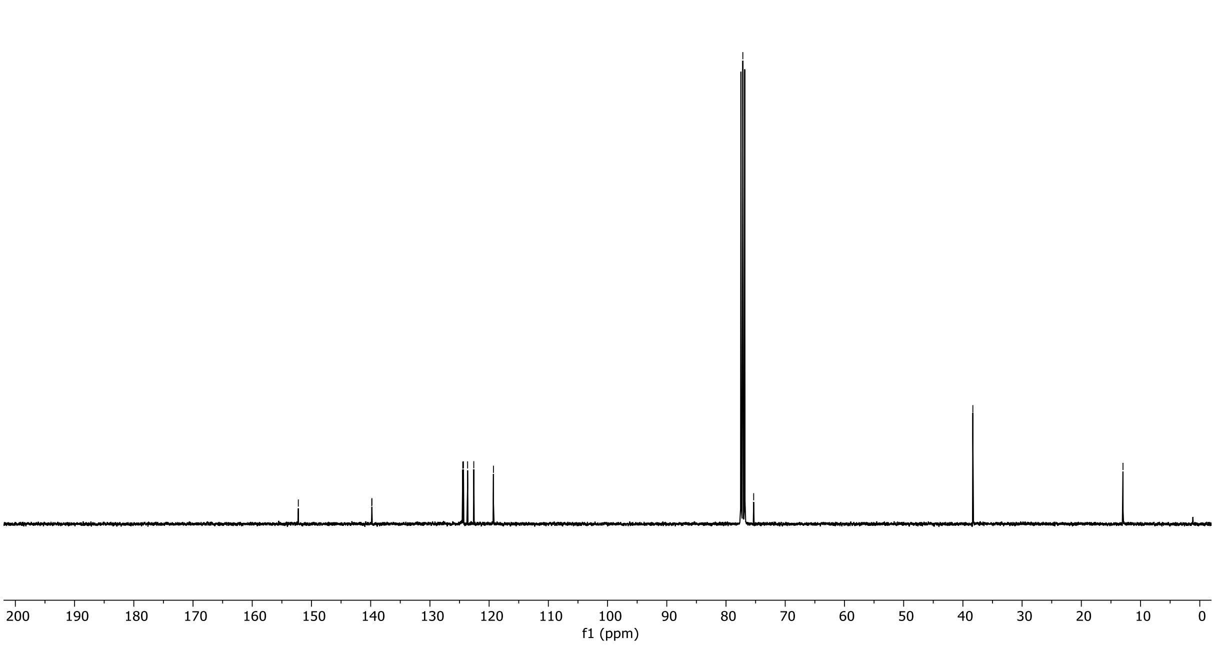

2-(cyclopent-1-en-1-yl)benzo[b]thiophene 5d

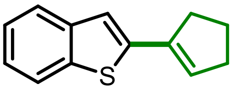

5d

^{1}H NMR (400 MHz, $CDCl_3$)

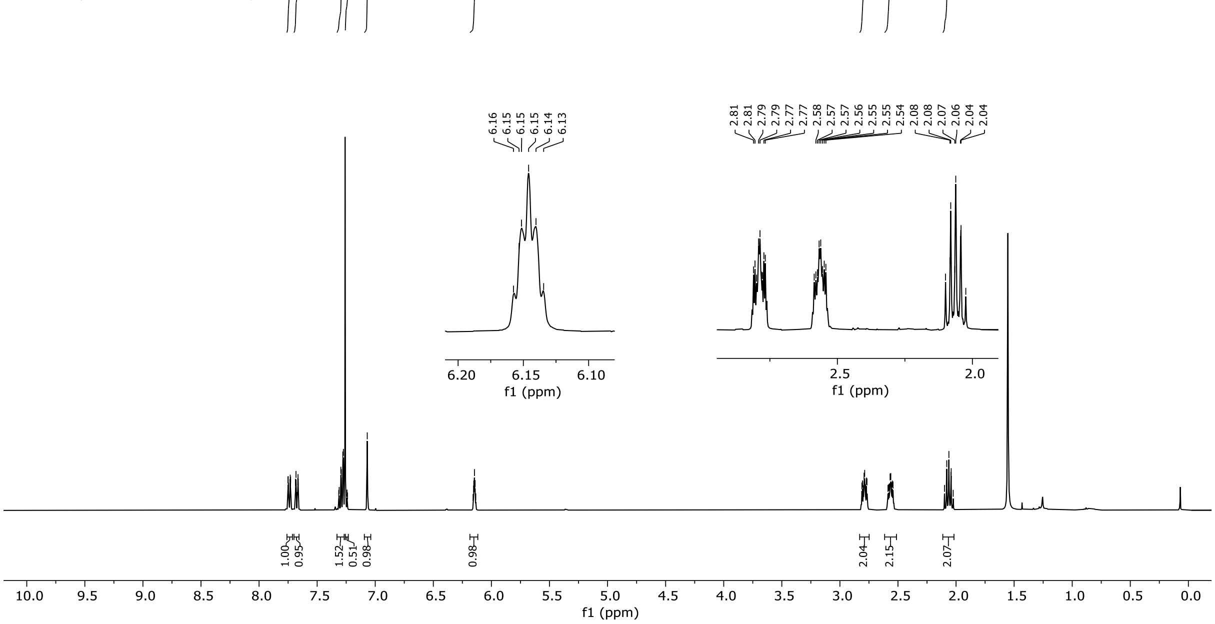

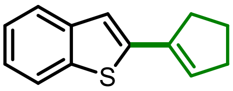

5d

^{13}C NMR (101 MHz, $CDCl_3$)

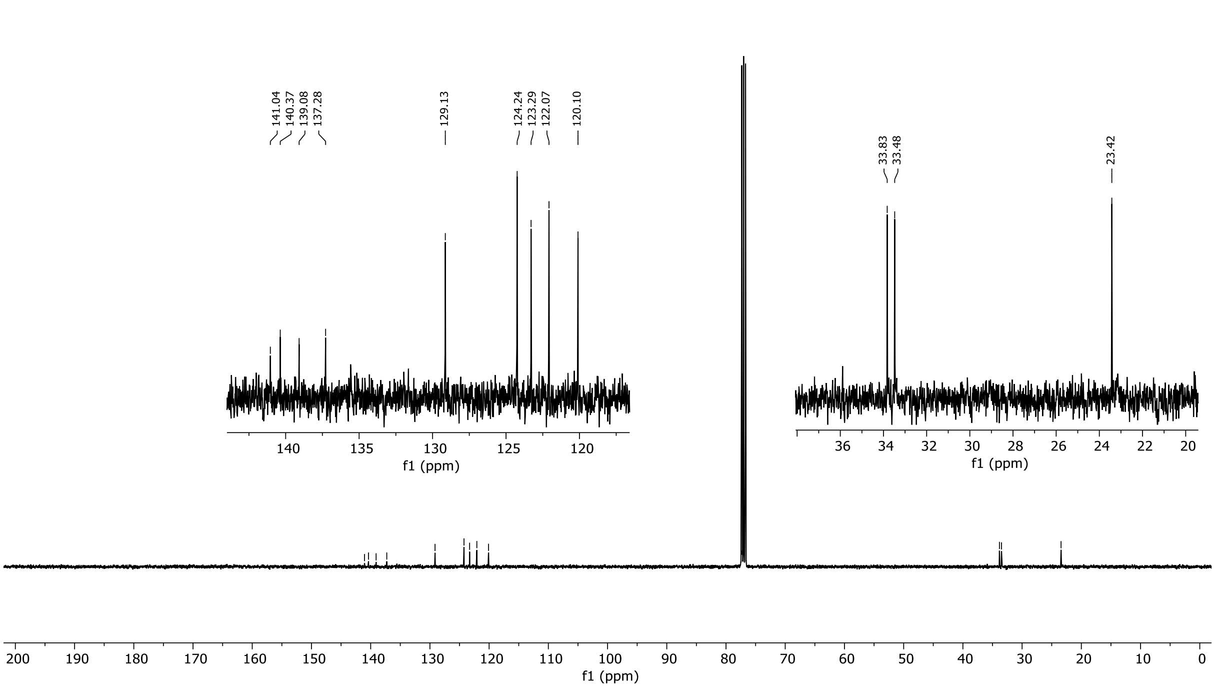

benzo[b]thiophene-2-carboxylic acid 5e

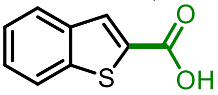

5e

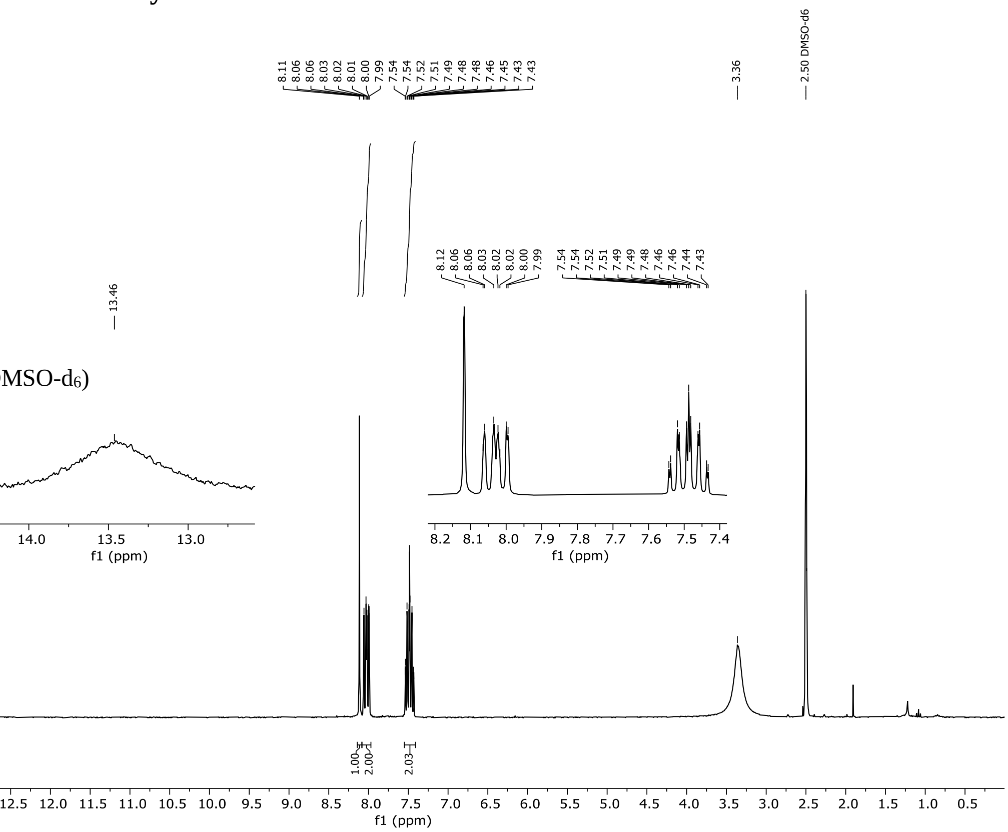

^{1}**H NMR** (300 MHz, DMSO-d$_6$)

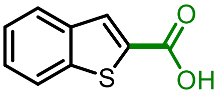

5e

^{13}**C NMR** (75 MHz, DMSO-d$_6$)

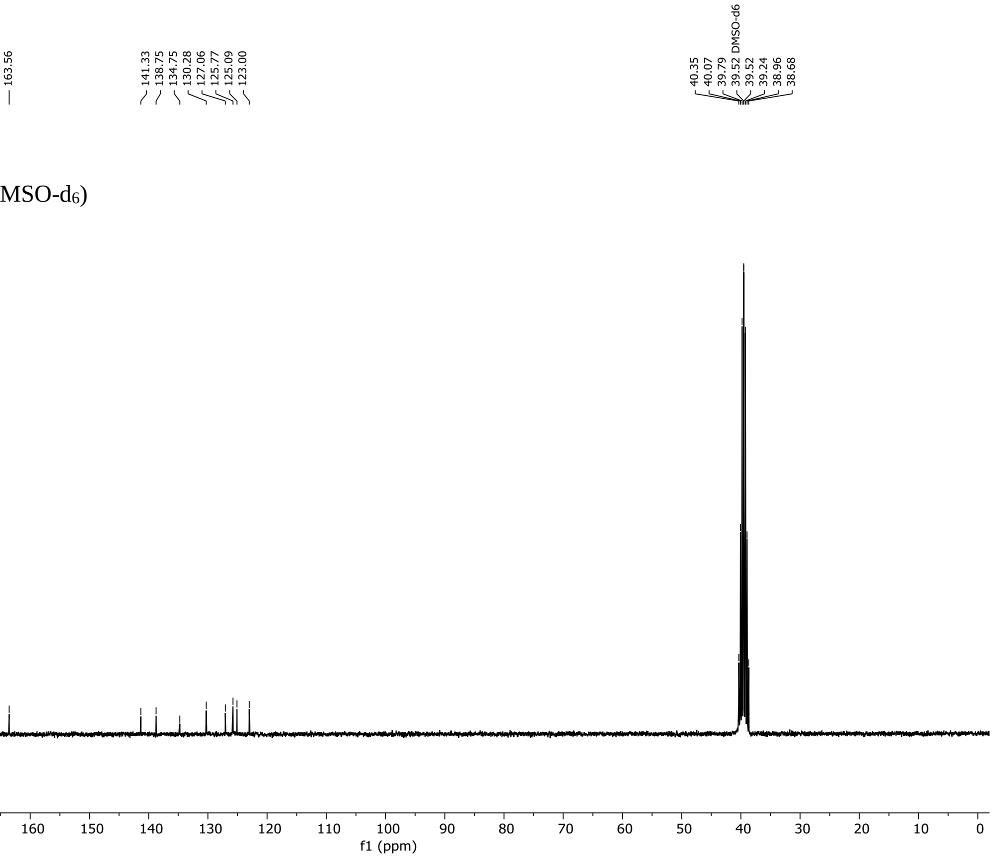

Diisopropyl (4-methoxy-3-(trifluoromethyl)phenyl)phosphonate 6a

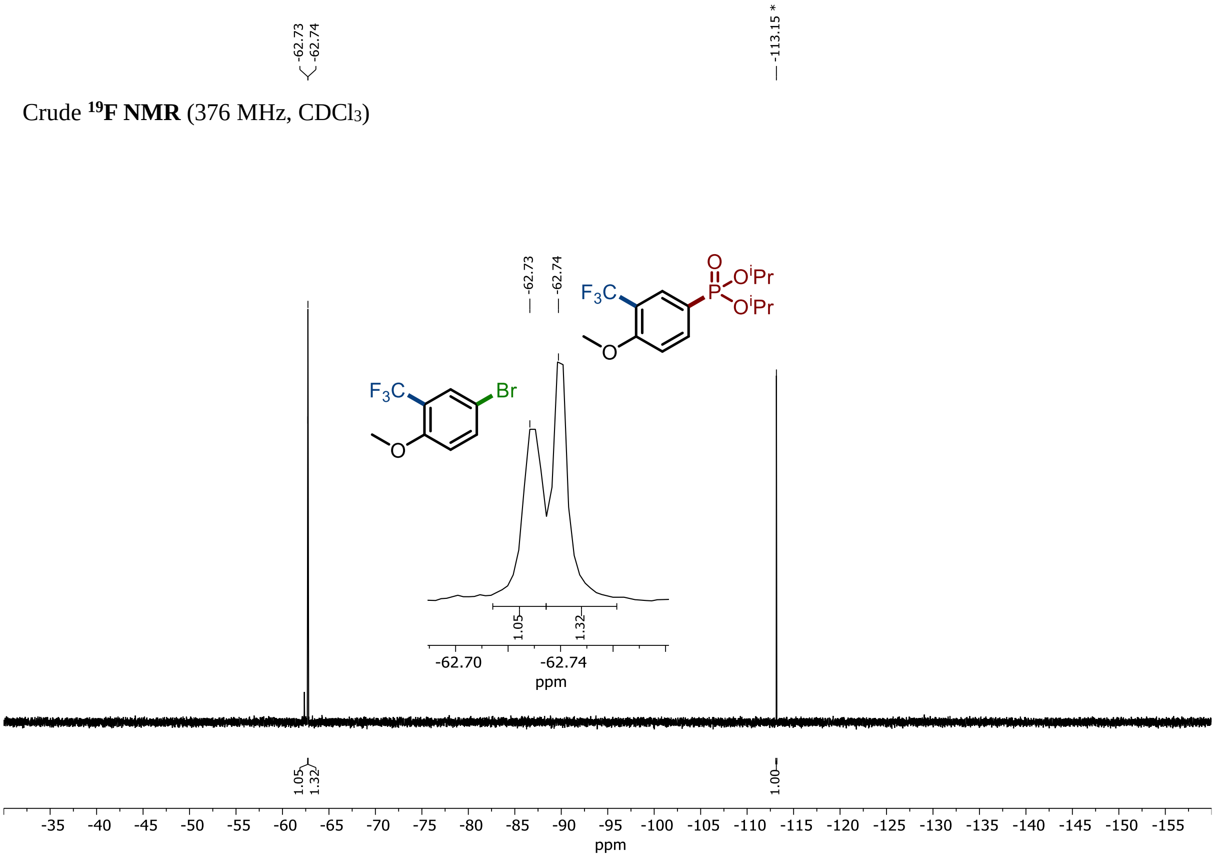

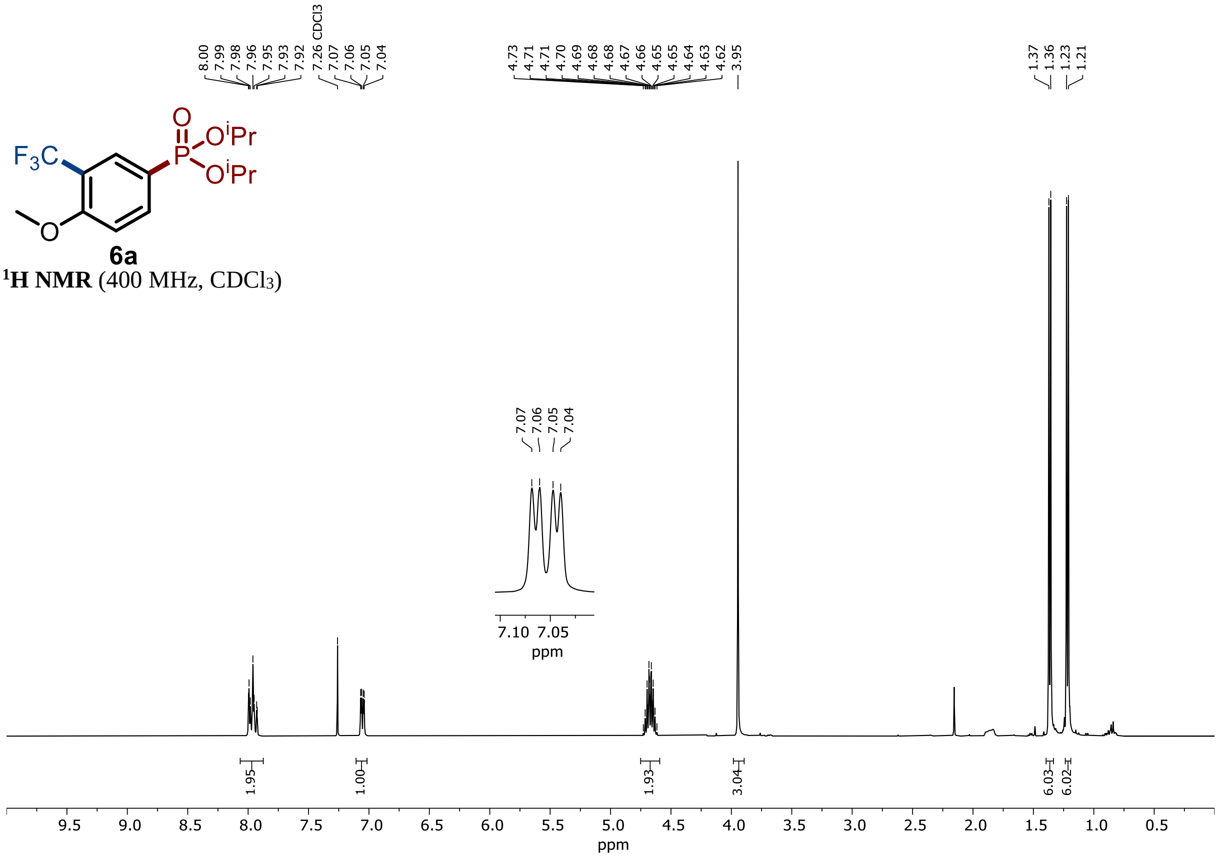

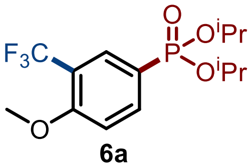

6a

^{13}C NMR (101 MHz, $CDCl_3$)

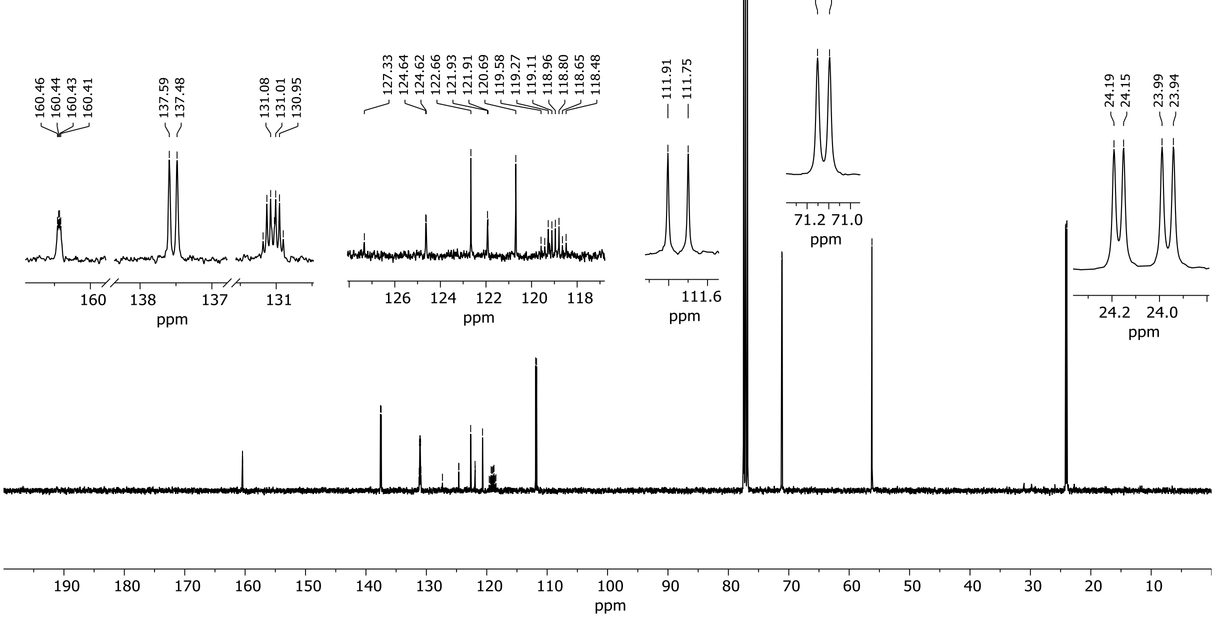

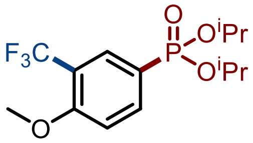

6a

^{19}F NMR (376 MHz, $CDCl_3$)

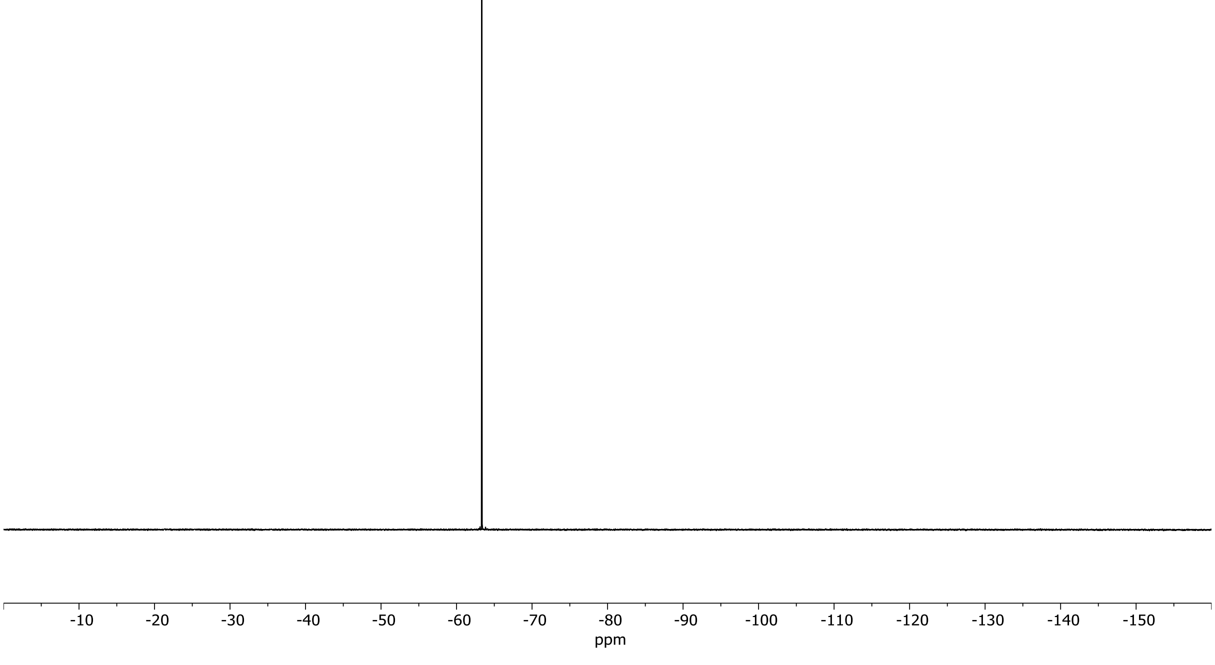

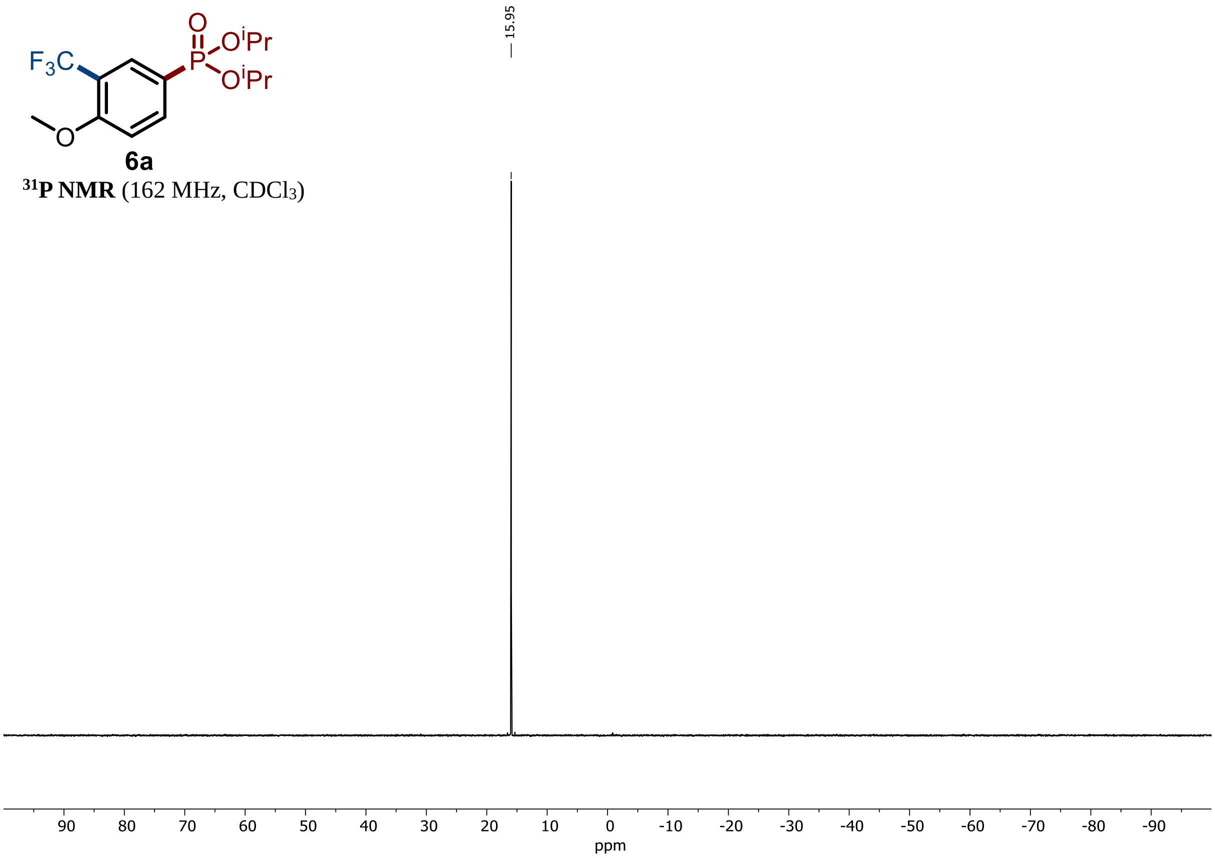

6a

^{31}P NMR (162 MHz, $CDCl_3$)

Dimethyl (4-methoxy-3-(trifluoromethyl)phenyl)phosphonate 6b

Crude **^{19}F NMR** (376 MHz, CDCl3)

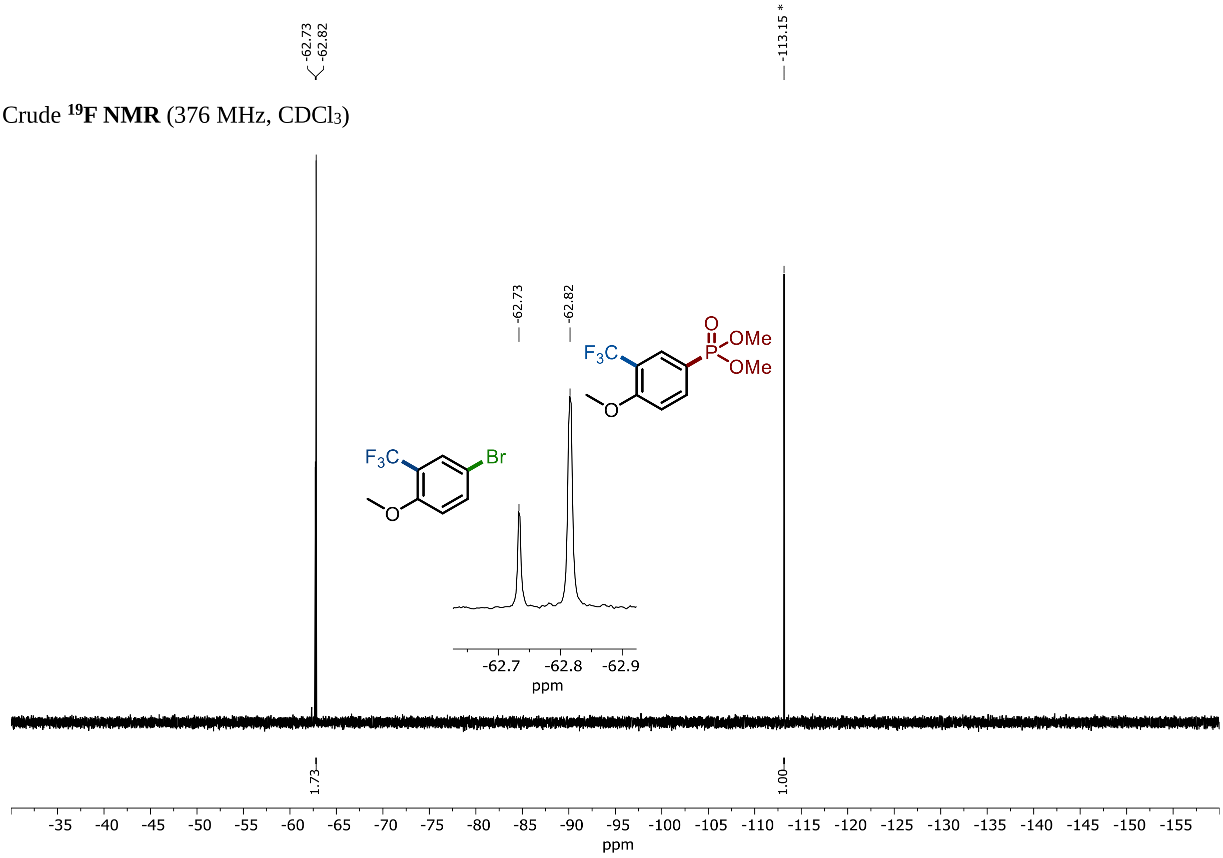

^{1}H NMR (400 MHz, CDCl3)

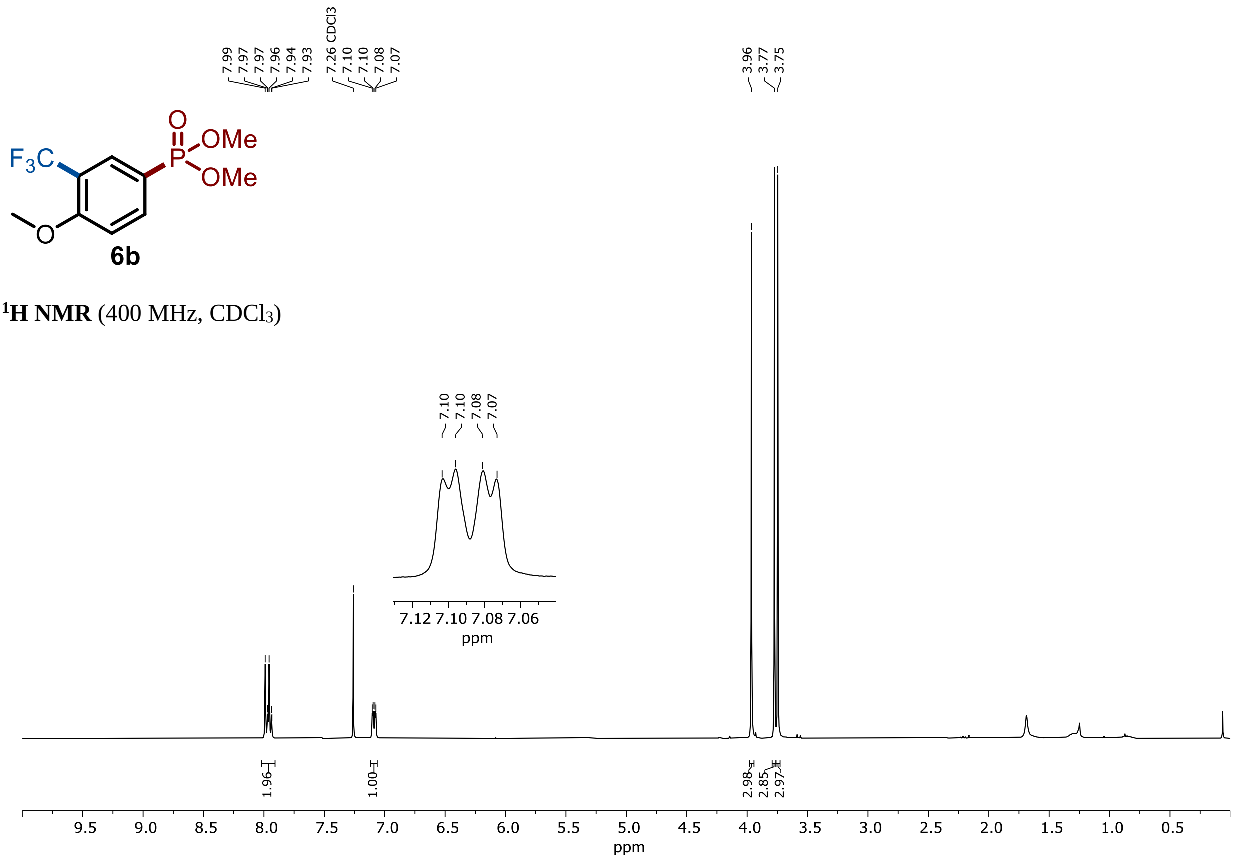

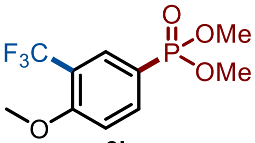

6b

^{13}C NMR (101 MHz, CDCl3)

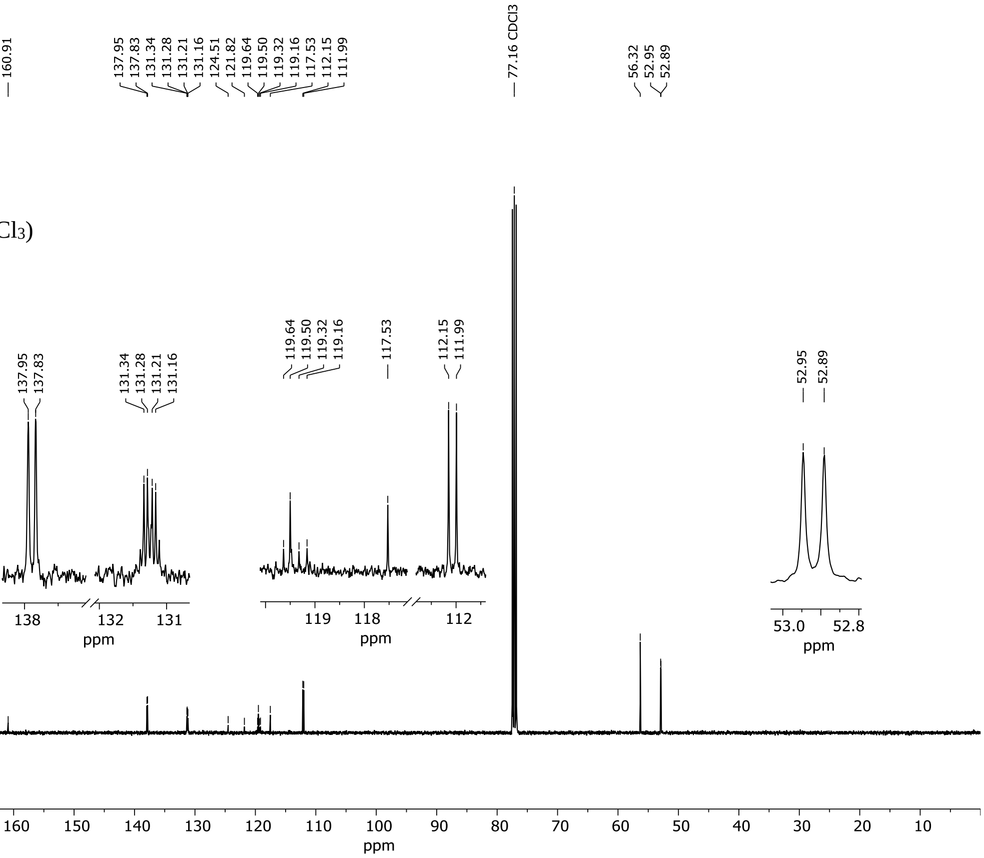

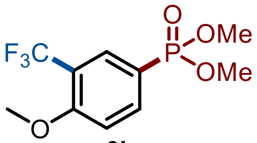

6b

^{19}F NMR (376 MHz, CDCl3)

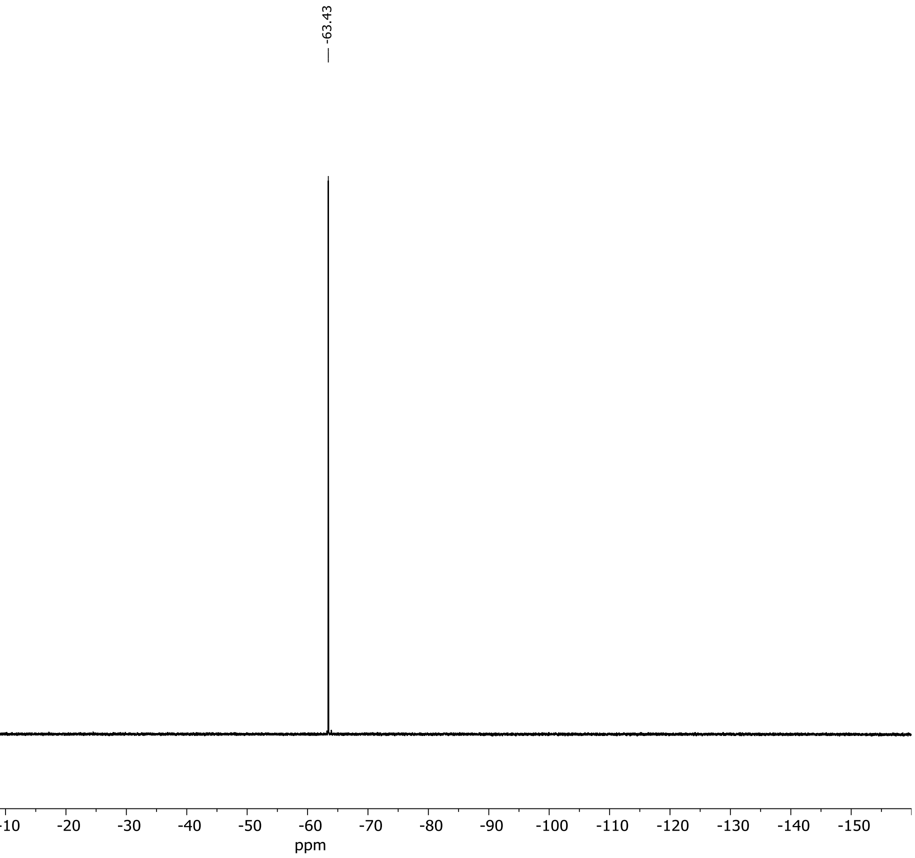

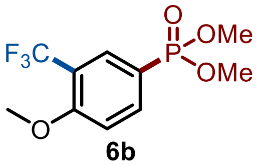

6b

^{31}P NMR (162 MHz, $CDCl_3$)

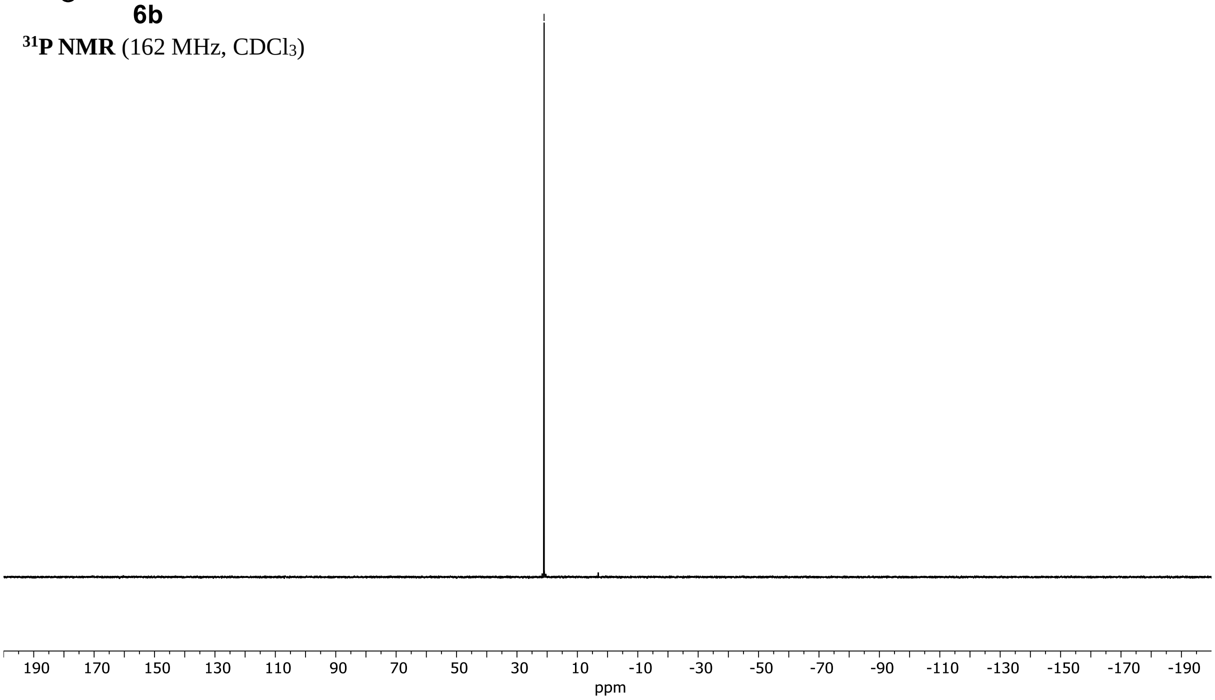

2-(4-methoxy-3-(trifluoromethyl)phenyl)-1-methyl-1H-pyrrole 6c

Crude ^{19}F NMR (376 MHz, $CDCl_3$)

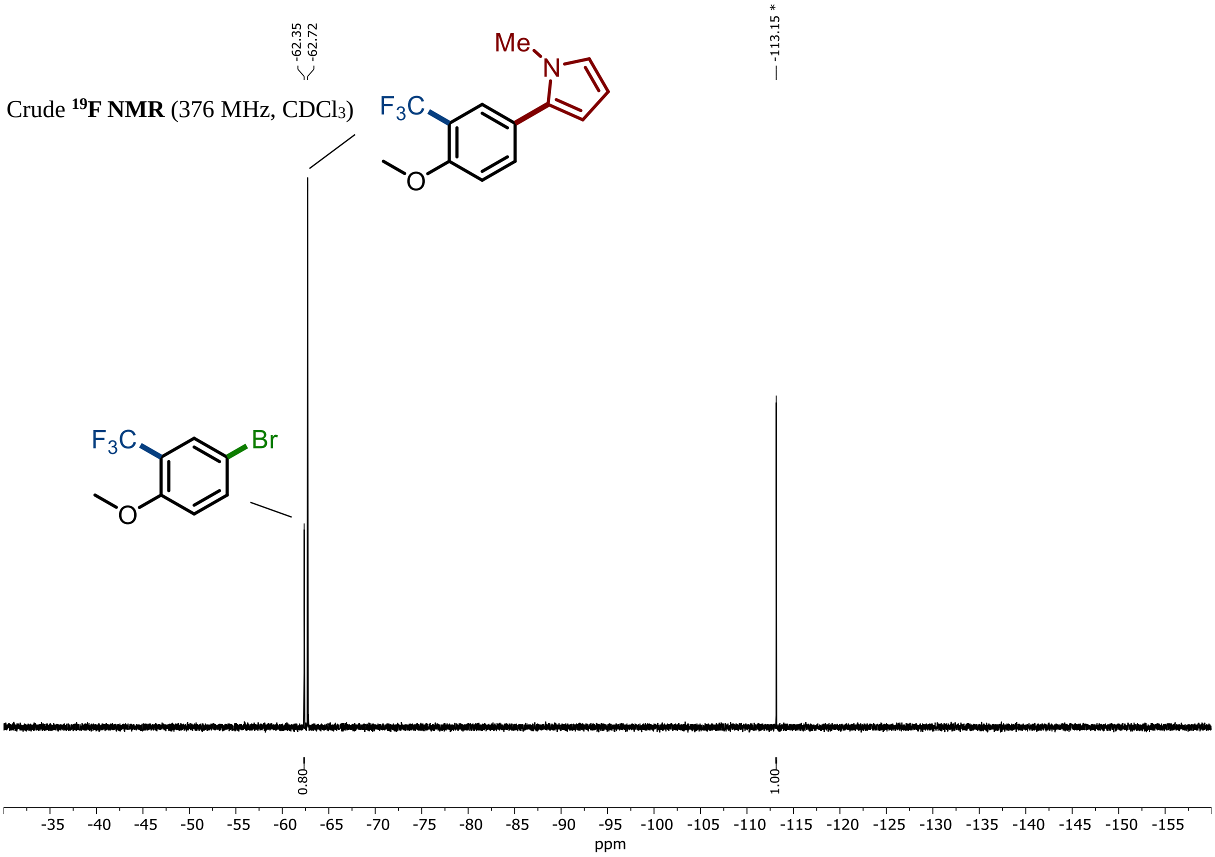

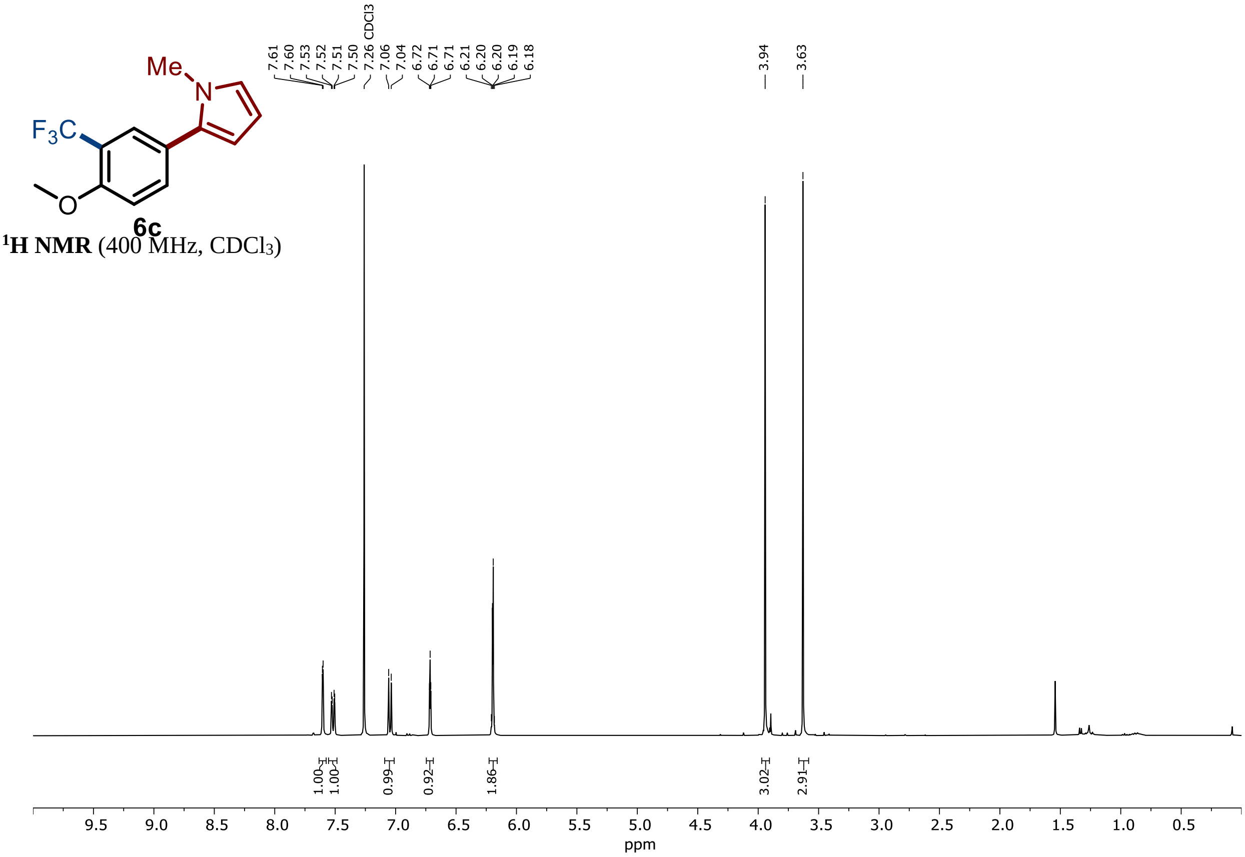

^{1}H NMR (400 MHz, $CDCl_3$)

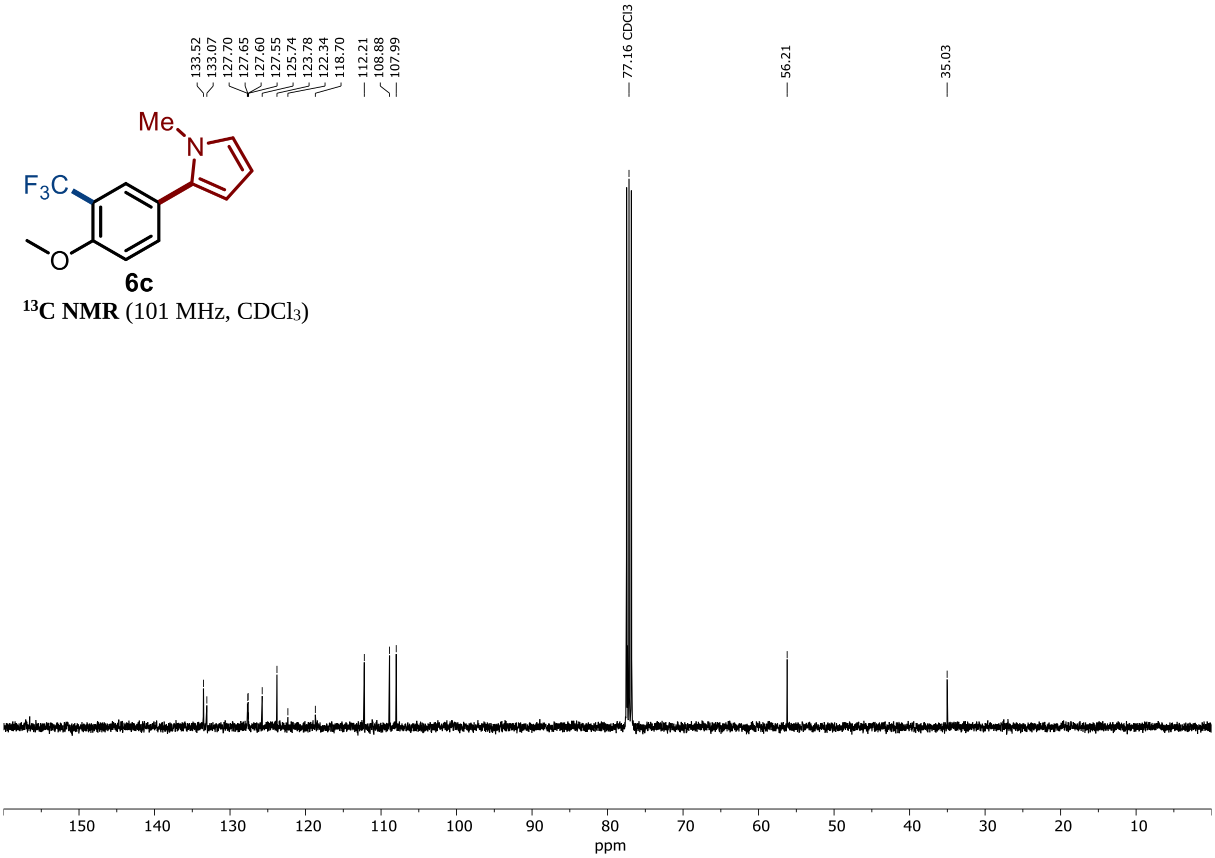

^{13}C NMR (101 MHz, $CDCl_3$)

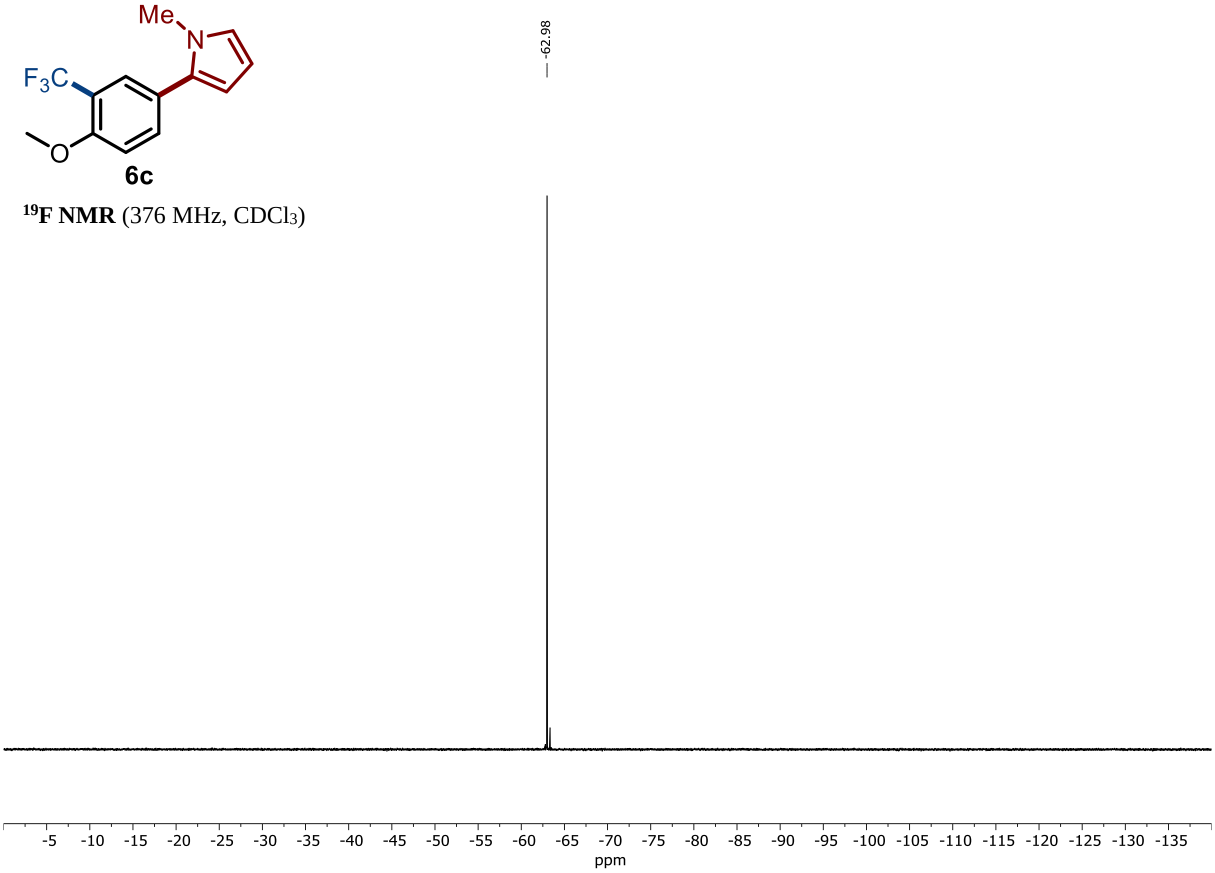

^{19}F NMR (376 MHz, $CDCl_3$)

10. References

[1] D. B. G. Williams, M. Lawton, *J. Org. Chem.* **2010**, *75*, 8351.
[2] R. J. Kutta, *Dissertation*, University of Regensburg, Regensburg, **2013**.
[3] Y. Suhara, M. Watanabe, S. Motoyoshi, K. Nakagawa, A. Wada, K. Takeda, K. Takahashi, H. Tokiwa, T. Okano, *J. Med. Chem.* **2011**, *54*, 4918.
[4] S. Himori, K. Maruyama, T. Sakurai, JP2009185165, **2008**.
[5] S. M. Husain, M. A. Schätzle, S. Lüdeke, M. Müller, *Angew. Chem. Int. Ed.* **2014**, *53*, 9806.
[6] M. Redaelli, C. Mucignat-Caretta, A. A. Isse, A. Gennaro, R. Pezzani, R. Pasquale, V. Pavan, M. Crisma, G. Ribaudo, G. Zagotto, *Eur. J. Med. Chem.* **2015**, *96*, 458.
[7] I. Ghosh, J. Khamrai, A. Savateev, N. Shlapakov, M. Antonietti, B. König, *Science* **2019**, *365*, 360.
[8] U. Megerle, R. Lechner, B. König, E. Riedle, *Photochem. Photobiol. Sci.* **2010**, *9*, 1400.
[9] V. V. Pavlishchuk, A. W. Addison, *Inorganica Chim. Acta* **2000**, *298*, 97.
[10] L. Li, X. Mu, W. Liu, Y. Wang, Z. Mi, C.-J. Li, *J. Am. Chem. Soc.* **2016**, *138*, 5809.
[11] Y. Ye, S. H. Lee, M. S. Sanford, *Org. Lett.* **2011**, *13*, 5464.
[12] D. A. Nagib, D. W. C. MacMillan, *Nature* **2011**, *480*, 224.
[13] J. M. Quibell, G. J. P. Perry, D. M. Cannas, I. Larrosa, *Chem. Sci.* **2018**, *9*, 3860.
[14] B. M. Campbell, J. B. Gordon, E. R. Raguram, M. I. Gonzalez, K. G. Reynolds, M. Nava, D. G. Nocera, *Science* **2024**, *383*, 279.
[15] E. Mejía, A. Togni, *ACS Catal.* **2012**, *2*, 521.
[16] Y. Ji, T. Brueckl, R. D. Baxter, Y. Fujiwara, I. B. Seiple, S. Su, D. G. Blackmond, P. S. Baran, *PNAS* **2011**, *108*, 14411.
[17] L. Skora, W. Jahnke, *ACS Med. Chem. Lett.* **2017**, *8*, 632.
[18] J. R. Box, M. E. Avanthay, D. L. Poole, A. J. J. Lennox, *Angew. Chem. Int. Ed.* **2023**, *62*, e202218195.
[19] R. Sakamoto, H. Kashiwagi, K. Maruoka, *Org. Lett.* **2017**, *19*, 5126.
[20] R. S. Shaikh, I. Ghosh, B. König, *Chem. Eur. J.* **2017**, *23*, 12120.
[21] L. Niu, J. Liu, H. Yi, S. Wang, X.-A. Liang, A. K. Singh, C.-W. Chiang, A. Lei, *ACS Catal.* **2017**, *7*, 7412.
[22] M. Koohgard, M. Hosseini-Sarvari, *Org. Biomol. Chem.* **2021**, *19*, 5905.
[23] Y. Shimada, K. Hattori, N. Tada, T. Miura, A. Itoh, *Synthesis* **2013**, *45*, 2684.
[24] A. Bouziane, B. Carboni, C. Bruneau, F. Carreaux, J.-L. Renaud, *Tetrahedron* **2008**, *64*, 11745.
[25] B. L. Ryland, S. D. McCann, T. C. Brunold, S. S. Stahl, *J. Am. Chem. Soc.* **2014**, *136*, 12166.
[26] S. Musa, I. Shaposhnikov, S. Cohen, D. Gelman, *Angewandte Chemie (International ed. in English)* **2011**, *50*, 3533.
[27] A. Iben Ayad, C. Belda Marín, E. Colaco, C. Lefevre, C. Méthivier, A. Ould Driss, J. Landoulsi, E. Guénin, *Green Chem.* **2019**, *21*, 6646.
[28] K. Matsuo, M. Shindo, *Org. Lett.* **2010**, *12*, 5346.
[29] K. Nguyen, van Nguyen, H. Tran, P. Pham, *RSC Adv.* **2023**, *13*, 7168.
[30] M. Zhang, Y. Zhai, S. Ru, D. Zang, S. Han, H. Yu, Y. Wei, *Chem. Commun.* **2018**, *54*, 10164.
[31] G. Laudadio, S. Govaerts, Y. Wang, D. Ravelli, H. F. Koolman, M. Fagnoni, S. W. Djuric, T. Noël, *Angew. Chem. Int. Ed.* **2018**, *57*, 4078.
[32] F. Bilodeau, M.-C. Brochu, N. Guimond, K. H. Thesen, P. Forgione, *J. Org. Chem.* **2010**, *75*, 1550.
[33] T. Shi, X. Wang, G. Yin, Z. Wang, *Org. Chem. Front.* **2022**, *9*, 1599.
[34] J. Wen, S. Qin, L.-F. Ma, L. Dong, J. Zhang, S.-S. Liu, Y.-S. Duan, S.-Y. Chen, C.-W. Hu, X.-Q. Yu, *Org. Lett.* **2010**, *12*, 2694.
[35] I. Ghosh, B. König, *Angew. Chem. Int. Ed.* **2016**, *55*, 7676.
[36] T. Yamamoto, T. Morita, J. Takagi, T. Yamakawa, *Org. Lett.* **2011**, *13*, 5766.
[37] S. Sengmany, A. Ollivier, E. Le Gall, E. Léonel, *Org. Biomol. Chem.* **2018**, *16*, 4495.
[38] Y. Kuninobu, H. Ida, M. Nishi, M. Kanai, *Nat. Chem.* **2015**, *7*, 712.

[39] R. S. Shaikh, S. J. S. Düsel, B. König, *ACS Catal.* **2016**, *6*, 8410.
[40] Y. Belabassi, S. Alzghari, J.-L. Montchamp, *J. Organomet. Chem* **2008**, *693*, 3171.
[41] M. Schmalzbauer, T. D. Svejstrup, F. Fricke, P. Brandt, M. J. Johansson, G. Bergonzini, B. König, *Chem* **2020**, *6*, 2658.
[42] C. Eberle, B. S. Lauber, D. Fankhauser, M. Kaiser, R. Brun, R. L. Krauth-Siegel, F. Diederich, *ChemMedChem* **2011**, *6*, 292.
[43] D. Meyer, P. Renaud, *Angew. Chem. Int. Ed.* **2017**, *56*, 10858.
